# *Myxococcus xanthus* DK1622 Coordinates Expressions of the Duplicate *groEL* and Single *groES* Genes for Synergistic Functions of GroELs and GroES

**DOI:** 10.3389/fmicb.2017.00733

**Published:** 2017-04-27

**Authors:** Li Zhuo, Yan Wang, Zheng Zhang, Jian Li, Xiao-Hua Zhang, Yue-zhong Li

**Affiliations:** ^1^State Key Laboratory of Microbial Technology, School of Life Science, Shandong UniversityJinan, China; ^2^College of Marine Life Sciences, Ocean University of ChinaQingdao, China

**Keywords:** *Myxococcus xanthus*, chaperonins, interdependence, duplicate *groEL*s, single *groES*, coordinated expression, self-regulation

## Abstract

Chaperonin GroEL (Cpn60) requires cofactor GroES (Cpn10) for protein refolding in bacteria that possess single *groEL* and *groES* genes in a bicistronic *groESL* operon. Among 4,861 completely-sequenced prokaryotic genomes, 884 possess duplicate *groEL* genes and 770 possess *groEL* genes with no neighboring *groES*. It is unclear whether stand-alone *groEL* requires *groES* in order to function and, if required, how duplicate *groEL* genes and unequal *groES* genes balance their expressions. In *Myxococcus xanthus* DK1622, we determined that, while duplicate *groEL*s were alternatively deletable, the single *groES* that clusters with *groEL1* was essential for cell survival. Either GroEL1 or GroEL2 required interactions with GroES for *in vitro* and *in vivo* functions. Deletion of *groEL1* or *groEL2* resulted in decreased expressions of both *groEL* and *groES*; and ectopic complementation of *groEL* recovered not only the *groEL* but also *groES* expressions. The addition of an extra *groES* gene upstream *groEL2* to form a bicistronic operon had almost no influence on *groES* expression and the cell survival rate, whereas over-expression of *groES* using a self-replicating plasmid simultaneously increased the *groEL* expressions. The results indicated that *M. xanthus* DK1622 cells coordinate expressions of the duplicate *groEL* and single *groES* genes for synergistic functions of GroELs and GroES. We proposed a potential regulation mechanism for the expression coordination.

## Introduction

GroEL proteins belong to the chaperonin of the chaperonin-60 family (Cpn60) that assists in protein folding, assembly, transport, and degradation (Ranson et al., 1998; Lund, 2001), and are essential for many physiological processes in cells (Houry et al., 1999). GroEL is usually a major heat-shock protein that is over-produced under non-permissive temperatures and aids in proper refolding of the proteins denatured by heat shock (Vanbogelen et al., 1987; Fayet et al., 1989; Kerner et al., 2005). The GroEL protein is a Type I chaperonin and is typically characterized by the formation of a 14-mer homopolymer arranged as two back-to-back/stacked rings, each of which comprises seven subunits (Horwich et al., 2006). In bacteria with single *groEL* genes, the co-chaperonin GroES, belonging to the chaperonin of the chaperonin-10 family (Cpn10) and encoded by the bicistronic *groESL* operon, forms a heptamer to bind to the GroEL homopolymer in the presence of ATP, forming a large central cavity that encapsulates substrate proteins and enables correct folding through multiple cycles of binding and release (Saibil and Ranson, 2002).

While most bacteria contain single copies of *groEL*, some possess two or more highly conserved *groEL* genes in their genomes (Craig et al., 1993; Kong et al., 1993; Karlin and Brocchieri, 2000; Gould et al., 2007; Jiang et al., 2008; Wang et al., 2013, 2014). In bacteria possessing single *groEL* genes, *groES* is always present upstream *groEL* in a bicistronic operon (*groESL*); in species with duplicate *groEL* genes, not all *groELs* are preceded by a *groES* gene (Lund, 2009). There are two possibilities for functioning manner of the stand-alone *groEL*: the encoded protein has evolved into a GroES-independent chaperonin or kept to function in a GroES-dependent manner. Goyal et al. suggested that, *groES* might be selectively lost from the operon after duplication of *groESL*, resulting in stand-alone *groEL* genes that then evolved to function in a *groES*-independent manner (Goyal et al., 2006). In *Anabaena* L-31, there are a *groESL* operon and a single *groEL* gene. Potnis et al. (2016) found that *Anabaena* GroEL1 formed stable higher oligomer (>12-mer) and prevented thermal aggregation of malate dehydrogenase (MDH), a substrate that requires a chaperonin for refolding following denaturation (Kumar et al., 2015). The refolding activity of *Anabaena* GroEL1 was lower than that of *Escherichia coli* GroEL, but independent of both GroES and ATP; and the presence of GroES enhanced the ATPase activity of *Anabaena* GroEL (Potnis et al., 2016). Qamra et al. also determined that the cpn60.2 (a GroEL homolog) proteins of *Mycobacterium tuberculosis* were able to polymerize into smaller oligomers, which alone retained the ability to suppress the aggregation of substrate proteins (Qamra et al., 2004). Interestingly, Kong et al. observed an independent expression of the *groES* and *groEL* genes in an operon in *M. tuberculosis* (Kong et al., 1993). However, it is still largely unknown whether stand-alone *groEL* genes require *groES* in order to function and, if required, how duplicate *groEL*s and a single *groES* balance their expressions.

Myxobacteria are phylogenetically located in the delta division of Proteobacteria. These bacteria are distinguished by their complex multicellular behaviors, such as moving in swarms on solid surfaces, cooperative feeding on macromolecules or other microbial cells, and the development of multicellular fruiting bodies embodied with numerous adversity-resistant myxospores (Shimkets, 1990; Dworkin and Kaiser, 1993). *Myxococcus xanthus* DK1622 is a model strain of myxobacteria. The bacterium contains two *groEL* genes; *groEL1* appears in a *groESL* operon, while *groEL2* has no neighboring *groES* (Goldman et al., 2006; Li et al., 2010). The two *groEL* genes are alternatively deletable for cell growth, but function divergently in specific cellular processes: *groEL1* is essential for the development, while *groEL2* is required for cell predation and the biosynthesis of secondary metabolite myxovirescin (Li et al., 2010; Wang et al., 2013, 2014). The expression of *groEL2* is usually less than half of *groEL1* in either the growth or the developmental stage. However, in the mutant with the deletion of *groEL1* (YL0301), the expression of the *groEL2* gene increased more than two-fold of that in wild-type strain DK1622; whereas the expression of *groEL1* in the *groEL2* deletion mutant (YL0302) was similar to that in the wild-type strain DK1622 (Li et al., 2010). In response to heat shock, the expression of the *groEL* genes was regulated in a complex manner in *groEL* deletion and complementary mutants, which led to the total expressions of *groEL* to approach the expression level in the wild type strain (Wang et al., 2013). Seemly, there is a balance of the expressions of *groEL* genes in *Myxococcus* cells for their cellular functions. In this study, comparative genomics analysis revealed that stand-alone *groEL* gene often appears in bacteria possessing two or more *groEL* genes. Using *M. xanthus* DK1622 as a model, we determined that the single *groES* gene was essential for cell survival, and both GroEL1 and GroEL2 required GroES in order to function. The duplicate *groEL* genes and the single *groES* gene are expressed in a coordinated manner for their synergetic functions in *M. xanthus* cells. A self-regulatory mechanism was proposed to explain how a single *groES* gene simultaneously meets the requirements of two endogenous *groEL* genes.

## Materials and methods

### Cultures, plasmids, and growth conditions

The strains, plasmids and primers employed in this study are listed in Supplementary Tables 1, 2. The *M. xanthus* strains were cultivated in Casitone-based rich-nutrient CTT medium (Hodgkin and Kaiser, 1977) for growth assays. *E. coli* strains were routinely grown on Luria-Bertani (LB) agar or in LB broth. The incubation temperatures were 37°C and 30°C for *E. coli* cells and *M. xanthus* cells, respectively. When required, final concentrations of 40 μg/ml kanamycin, 100 μg/ml ampicillin, 34 μg/ml chloramphenicol, and 10 μg/ml tetracycline were added to the media.

### Bioinformatics analysis of the occurrence of *groEL* and *groES* genes in prokaryotic genomes

The two *groEL* genes and one *groES* gene in the *M. xanthus* DK1622 genome are *MXAN_RS23765* (*groEL1*), *MXAN_RS21695* (*groEL2*), and *MXAN_RS23760* (*groES*). The NCBI accession numbers for the GroEL1, GroEL2, and GroES protein sequences of *M. xanthus* DK1622 are WP_011554876.1, WP_011554465.1, and WP_002640434.1, respectively. The conserved domain information of GroEL and GroES was obtained from the CDD protein family (cdd239460 for GroEL and cdd238197 for GroES of *M. xanthus* DK1622, respectively; Marchler-Bauer et al., 2017). Based on the sequence similarity of the conserved domains, we performed RPS-BLAST (Reverse Position-Specific BLAST), which searches against the NCBI genome database (http://www.ncbi.nlm.nih.gov/genome/), and retrieved the superset of all the GroEL and GroES protein sequences in prokaryotic genomes. The *E*-value for the search was set to be equal to or higher than the default cutoff of 0.01.

A whole-genome-based myxobacterial phylogenetic tree was constructed online utilizing the CVTree3 program and a composition vector approach (Zuo and Hao, 2015).

### Deletion of *groES*

Deletion of *groES* was carried out in *M. xanthus* DK1622 using the positive-negative KG cassettes (Ueki et al., 1996). Genomic DNA from DK1622 served as the template for PCR amplification of the upstream and downstream homologous arms utilizing Pfu DNA polymerase. The arms were fused at the BamHI site to create internal deletion fragments, which were cloned into SmaI-digested pBJ113, forming the deletion plasmid pBJ-*groES*. The deletion plasmid was transferred via electroporation into *M. xanthus* DK1622 cells as previously described (Kashefi and Hartzell, 1995). Individual Km-resistant colonies were selected and inoculated onto CTT agar plates supplemented with 1% galactose for a second round of screening. Deletion mutants were identified based on their galactose resistance and kanamycin sensitivity phenotypes, as well as by PCR and sequencing verification (Li et al., 2010).

### Complementary inactivation experiments to knock out *groES*

The *groES* gene was fused to the *pilA* promoter (1 kb), and the fused sequence was digested with XbaI and EcoRI and ligated into the site-specific integration plasmid pSWU30, which had been digested with XbaI and EcoRI, to obtain the plasmid pSWU-*groES*. This plasmid was transferred via electroporation into *M. xanthus* DK1622 cells as previously described (Kashefi and Hartzell, 1995). Individual tetracycline-resistant colonies were selected, and the selected mutant was designated YL0308. The mutant was further employed to delete the local *groES* gene via electroporation with pBJ-*groES*. The km^r^ and ter^r^ mutants were selected, and proper integration of the fragment was confirmed by PCR and sequencing (Lobedanz and Sogaard-Andersen, 2003).

### Insertion of *groES* before *groEL2*

The *groES* gene, including the *groES-groEL1* intergenic region, was fused to the promoter (500 bp) of *groEL2*, which was then fused upstream of *groEL2*. The fused fragments were digested with XbaI and EcoRI and ligated into the site-specific integration plasmid pSWU30, which was digested with XbaI and EcoRI to obtain the plasmid pSWU-*P*_*groEL*2_+*groES*+*groEL2*. This plasmid was transferred via electroporation into *groEL2* deletion mutant YL0302 cells. Individual colonies with tetracycline-resistance were selected, and the mutant was designated YL1101. All primers mentioned above are listed in Supplementary Table 2.

### Construction of *groES* over-expression mutants via the pZJY41 plasmid

The promoter of either *groEL2* or *groEL1* was fused to the *groES* gene, and the fused fragments were digested with BamHI and EcoRI and ligated into the *Myxococcus-E. coli* shuttle plasmid pZJY41 (which had also been digested with BamHI and EcoRI) to obtain the plasmids pZJY-*P*_*groEL*2_+*groES* and pZJY-*P*_*groEL*1_+*groES*. The constructed plasmids were then transferred via electroporation into *M. xanthus* DK1622 cells, and the mutants were designated YL1102 and YL1103, respectively (Supplementary Table 1).

### Protein purification

The *M. xanthus groEL* and *groES* genes were separately cloned into pET22b and over–expressed in *E. coli* BL21(DE3) cells. The heterogeneously expressed GroELs and GroES were purified as described (Klunker et al., 2003), with some modifications. Briefly, BL21 cells were grown at 37°C in LB medium with 100 μg/ml ampicillin to an OD600 of 0.7 and induced with 1 mM IPTG. Cells were resuspended in lysis buffer (25 mM Tris-HCl, pH 8.0, 300 mM NaCl, 2 mM dithiothreitol) and lysed via sonication. After gentle ultrasonication, the mixtures were centrifuged at 4°C for 30 min (12,000 rpm). The supernatant containing His-tagged GroES was applied to a Ni^2+^-NTA column and purified by Superdex 200 chromatography. His-tag sequences were not added to the GroEL proteins to maintain target protein activity. The supernatants containing GroEL were concentrated using tubes with a 100-kDa cut-off and were further purified by chromatography (Superdex 200).

### ATPase assay

An ATPase assay was performed as previously described (Figueiredo et al., 2004) with minor modifications. Briefly, 1 μM GroEL1 and GroEL2 were individually added to a reaction buffer containing 20 mM MOPS, pH 7.5, 100 mM KCl, 5 mM MgCl_2_, and 0.5 M ammonium sulfate with or without 2 μM GroES at 37°C. The reaction was initiated by adding 2 mM ATP and stopped with 20 mM CDTA after 10 min. Liberated inorganic phosphate was quantified by the malachite green assay (Adenosinetriphosphatase assay kit from Nanjing Jiancheng Bioengineering Institute, China) after incubation at 25°C for 30 min. The absorption was measured at 640 nm. Three independent replicates were performed for each experiment.

### Refolding assay

Twenty micromolars malate dehydrogenase (MDH) was denatured in 1 M guanidinium HCl for 60 min, and the reaction system was diluted 100-fold with buffer A (50 mM Tris-HCl, pH 7.0, 0.5 M ammonium sulfate, and 2 mM ATP). Next, 1 μM GroEL1 and GroEL2 were separately added into buffer A with or without 2 μM GroES. The concentration of added chaperonin was determined by NanoDrop 2000 Spectrophotometer. The reaction mixture was incubated at room temperature for 60 min (Figueiredo et al., 2004). Fifty millimolars CDTA was added to stop the reaction. The enzymatic activity of MDH was measured every 10 min at 340 nm using buffer B, containing 50 mM Tris-HCl, pH 7.0, and 0.5 M ammonium sulfate, 1 mg/ml bovine serum albumin, 0.22 mM NADH, 0.55 mM oxaloacetate, 1 mM CDTA. For citrate synthase (CS) refolding assay, after a similar process, the enzymatic activity of CS was determined every 10 min at 412 nm using the Citrate Synthase Activity Assay Kit (Sigma-Alorich). Three independent replicates were performed for each experiment.

### Protein pull-down assay

Proteins were purified as described above. Excess amount of GroEL1 (20 mg) or GroEL2 (20 mg) proteins was mixed with 1–4 mg His-tagged GroES in suspension buffer (25 mM Tris-HCl, pH 8.0, 300 mM NaCl, 2 mM dithiothreitol, and 2 mM ATP) for 3 h. The mixtures were eluted through nickel columns. Then the columns were washed three times using washing buffer, and the bound proteins were eluted from the columns with 500 mM imidazole and detected by SDS-PAGE.

### Native PAGE

Hetero-expression of the duplicate GroELs and GroES was performed using pET22b-GroEL1-NoHis, pET22b-GroEL2-NoHis, and pET28a-GroES-NoHis respectively (Supplementary Table 1), in *E. coli* BL21(DE3) cells. BL21 cells were treated as described as above. After gentle ultrasonic treatment, cells were centrifuged at 4°C for 30 min (12,000 rpm) for the native PAGE assay. Compared to normal sodium dodecyl sulfate (SDS)-PAGE, the protein buffer and gel for native PAGE contained no SDS or mercaptoethanol. Electrophoresis was performed in a mixture of ice and water to avoid the depolymerization of multimer proteins.

### *In vivo* refolding assay

To investigate the *in vivo* refolding activities of GroEL1/2 proteins with or without the presence of GroES, we constructed the plasmids pET22b-HrcA, pBAD33-GroESL1/2, and pBAD33-GroEL1/2 (Supplementary Figure 1). The compatible plasmids pET22b-HrcA and pBAD33-GroESL1/2 (or pBAD33-GroEL1/2) were transformed into *E. coli* Top 10F' cells (Supplementary Table 1), and transformants were selected under the double-resistance conditions of 34 μg/ml chloramphenicol and 100 μg/ml ampicillin. When the culture OD600 reached 0.7, 100 mM IPTG was added for the induction. Then, cells were harvested, resuspended in lysis buffer containing 25 mM Tris-HCl, pH 8.0, 300 mM NaCl, 2 mM dithiothreitol, and lysed via sonication. After gentle ultrasonication, the mixtures were centrifuged at 4°C for 30 min (12,000 rpm), which divided the mixture into supernatants and sediments. The samples were tested the expression and solubility of HrcA protein by SDS-PAGE.

### Quantitative real-time PCR analysis

*M. xanthus* DK1622 cells and *groEL* mutants were inoculated at final concentration of 1 × 10^7^ cells/ml in CTT medium and cultured for 36 h to the exponential growth stage, respectively. Then 0.5 ml of each of the cultures was transferred into 50 ml fresh CTT medium for further cultivation. The cultures were harvested at periodic time points, and the RNA was extracted immediately using a bacterial RNA extraction kit (Thermo Fisher) according to the manufacturer's instructions. The purified RNA extracts were reverse-transcribed to cDNA. Quantitative real-time PCR was performed in a total reaction volume of 20 μl containing 1 μl of 250 nM primers, 10 μl of SYBR Green PCR master mix, 8.5 μl of RNase-free water, and 0.5 μl of a 10-fold-diluted cDNA template. 16S rRNA gene was used as the reference. The primers used for real-time PCR are listed in Supplementary Table 2.

### Heat-shock assays

Mid-log-phase cultures of *M. xanthus* cells were harvested as described above. After heat-shock treatment at 42°C for 30 min or 60 min, the cells were immediately serially diluted and plated on 1.5 and 0.3% CTT agar. After 6 days, colony-forming unit (CFU) numbers were calculated, using untreated cells as a control. The survival rate was calculated as CFU divided by the cell number before heat shock (5 × 10^9^; Li et al., 2010).

### Statistical analysis

The difference significance was analyzed statistically by using IBM SPSS Statistics for independent-samples *T*-test.

## Results

### Stand-alone *groEL* gene often appears in bacteria possessing duplicate *groEL* genes

We surveyed the occurrence of *groEL* and *groES* genes by RPS-BLAST searching based on sequence similarity, and retrieved a total of 5,658 *groEL* and 5,253 *groES* genes in 4,861 completely sequenced prokaryotic genomes (Supplementary Table 3). The number of *groEL* genes in a genome range from zero to seven, and 93.5% of these genomes contain one or more *groEL* genes (Table 1). Consistent with the previous report (Williams and Fares, 2010), Mycoplasma often lack the *groEL* genes (67 of the 105 sequenced genomes). In addition, some Spiroplasma, Ureaplasma, and unclassified bacteria have no *groEL* gene. In Archaea, strains from Euryarchaeota and TACK group also lack *groEL*. In the genomes with single *groEL*s, the *groEL* gene is always neighbored with a *groES* gene, forming a bicistronic *groESL* operon. There are 884 bacterial genomes possessing two or more *groEL* genes (19.5% of the 4,861 sequenced genomes). Of the total 5,658 *groEL* genes, 4,824 were found closely downstream a *groES* gene in a *groESL* operon, while the other 834 (14.7% of *groEL* genes) have no neighboring *groES* gene. The stand-alone *groEL* genes are distributed in 770 genomes; thus, some genomes contain two or more stand-alone *groEL* genes. This is consistent with a previous survey of 669 bacterial genomes in which the majority of *groEL* genes co-occurred with a neighboring *groES* gene, while others stood alone (Lund, 2009). Notably, of the total 5,253 *groES* genes, 435 stand alone with no neighboring *groEL* gene; these genes are distributed across 419 genomes.

**Table 1:** **Copy numbers of ***groEL*** and ***groES*** genes in sequenced prokaryotic genomes**.

**Copy numbers**	**0**	**1**	**2**	**3**	**4**	**5**	**6**	**7**
*groEL*	314	3,663	731	108	28	8	6	3
*groES*	304	4,027	402	100	19	8	1	0

**Figure 1:**
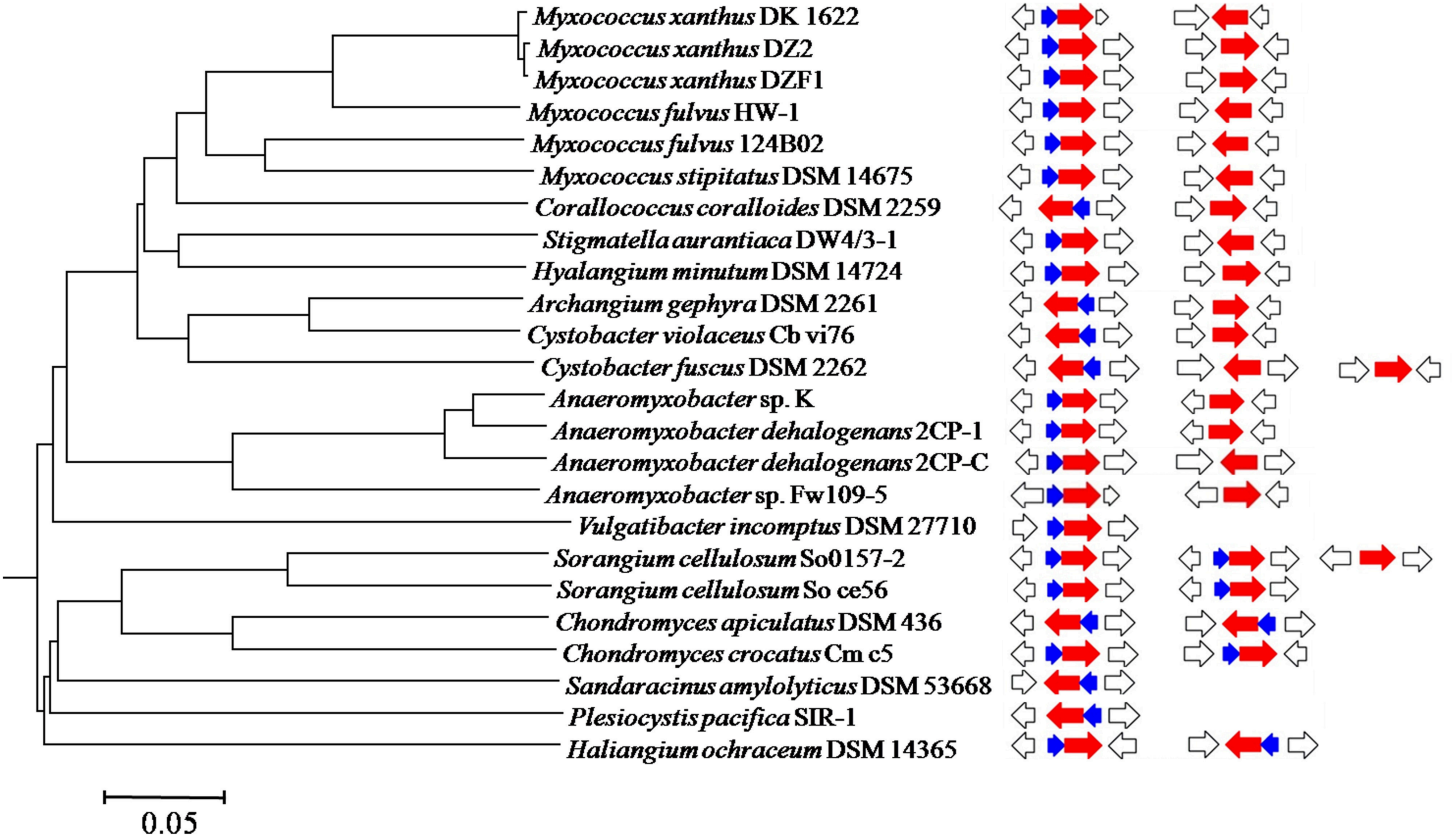
**A phylogenomic tree constructed using 24 sequenced myxobacterial genomes**. The bar equals to 0.05 of the phylogenetic distance. The occurrences of *groEL* and *groES* genes in each genome were correspondingly integrated into the tree. Blue, red, and white arrows indicate *groES, groEL* and flanking genes, respectively.

There are 24 sequenced myxobacterial genomes available in the GenBank database. Among them, 19 genomes contain two *groEL* genes, two contain three *groEL* genes, and three contain single *groEL* genes. Figure 1 is a diagrammatic sketch of the occurrence of *groEL* and *groES* genes in myxobacteria (details referred to Supplementary Table 4). All myxobacterial genomes possess at least one complete bicistronic *groESL* operon. Interestingly, the loss of a neighboring *groES* is rather a characteristic of duplicate *groEL* genes in the Cystobacterineae suborder of myxobacteria, while the four sequenced genomes of *Sorangium* and *Chondromyces* in the Sorangineae suborder and the marine halophilic strain *Haliangium ochraceum* DSM14365 contain a second *groESL* operon. Of the two genomes possessing three copies of *groEL*, the Sorangineae strain (*S. cellulosum* So0157-2) has two complete *groESL* operons and a stand-alone *groEL*, while the Cystobacterineae strain (*C. fuscus* DSM2262) has a complete *groESL* operon and two stand-alone *groEL* genes. The three myxobacteria with single *groEL* genes are newly identified myxobacterial strains that are phylogenetically distant from “the classical myxobacteria.” Although normally coinciding at a high taxonomic level, the occurrence of the *groES* and *groEL* genes may vary at the level of genus or even the species. For example, while one of the two *groEL* genes in *Cystobacter violaceus* Cb vi76 neighbors *groES* and one does not, the *C. fuscus* DSM 2262 strain has three *groEL* genes, of which one neighbors a *groES* gene and two stand alone. *S. cellulosum* So ce56 possesses two complete *groESL* clusters, but *S. cellulosum* So0157-2 contains two *groESL* clusters and a third stand-alone *groEL* gene.

### The *groES* gene is essential for the cell survival of *M. xanthus* DK1622

*M. xanthus* DK1622 possesses two *groEL* genes that share 79% similarity of their amino acid sequences (Goldman et al., 2006; Li et al., 2010). Based on our previous studies, a single *groEL1* or *groEL2* gene is capable of individually supporting the growth of DK1622 cells, but these two genes function divergently in predation and development processes (Li et al., 2010; Wang et al., 2013). Another significant characteristic of the two paralogous *groEL* genes is their organization differences in genome. The *groEL1* gene (*MXAN_RS23765*) lies downstream of *groES*, forming a complete bicistronic *groESL* operon, verified using RT-PCR (referred to Supplementary Figure 2), while *groEL2* (*MXAN_RS21695*) does not neighbor with a *groES* gene.

**Figure 2:**
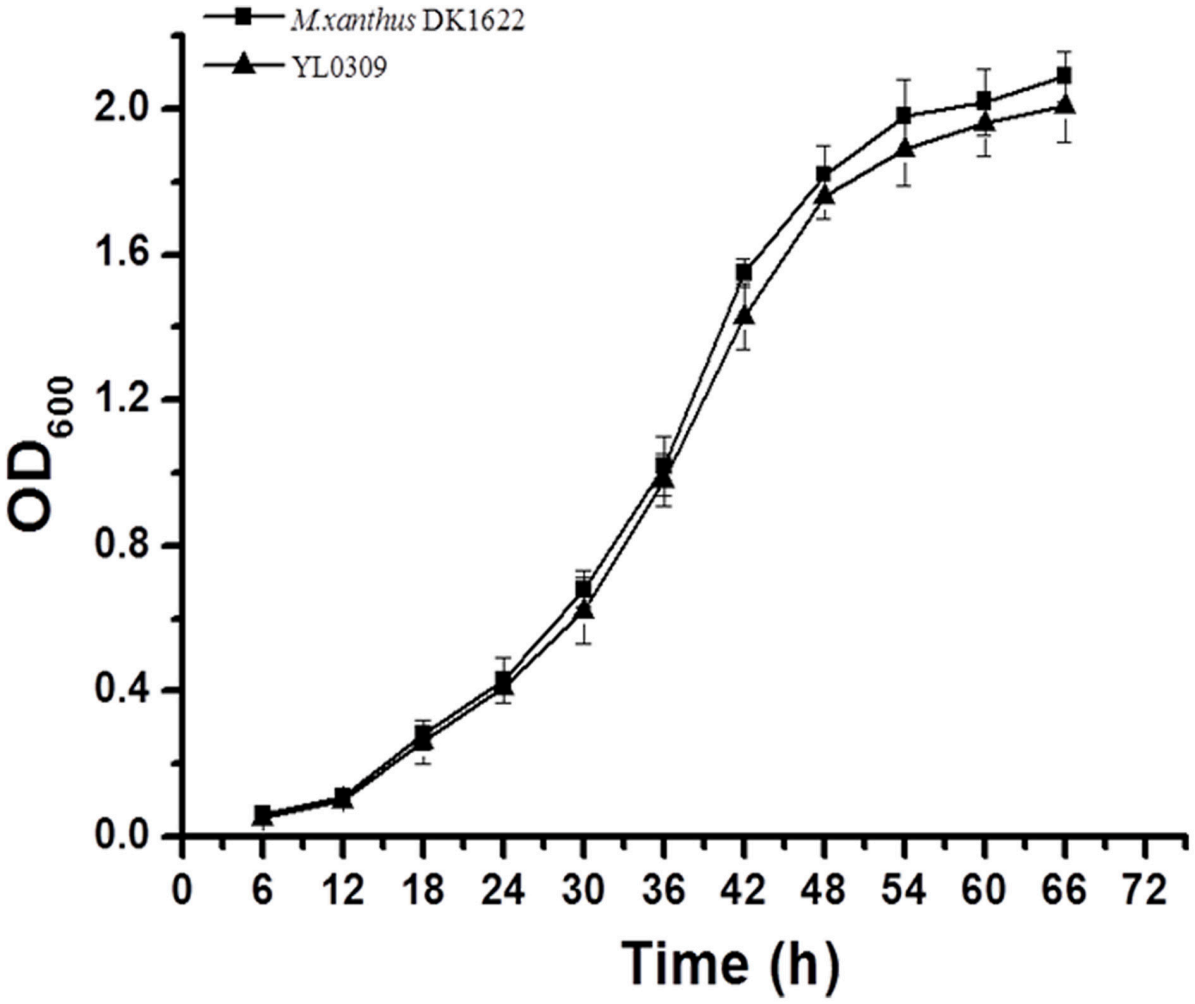
**Growth curves of the wild-type strain DK1622 and the ***groES***-deletion mutant YL0309 complemented with an ectopic ***groES*** gene**. Error bars represent standard deviation of three time repeats.

We attempted to knock out the *groES* gene but failed, suggesting that inactivation of the *groES* gene is fatal to DK1622 cells. To confirm the essentiality of *groES*, we complemented this gene at the Mx8 *attB* site in the DK1622 genome using pSWU30 to form a mutant with two copies of the *groES* gene (strain YL0308). We further attempted to delete the original *groES* from YL0308, and, as expected, obtained a viable mutant (YL0309), having an ectopically expressed *groES* gene and the stand-alone genes *groEL1* and *groEL2*. The YL0309 mutant had a nearly identical growth curve to the wild-type strain DK1622 (*P* > 0.05 for each detected time point; Figure 2). Thus, the *groES* gene is essential for the cell survival of *M. xanthus* DK1622. The *groEL1* and *groEL2* genes were alternatively deletable for cell survival, which suggested that GroEL2 also required GroES in order to function. *M. xanthus* cells were likely able to self-regulate *groEL* and *groES* gene expression to meet cellular requirements for these proteins.

### GroES is able to bind to either GroEL1 or GroEL2 and is required for GroEL refolding activities *In vitro*

**Figure 3:**
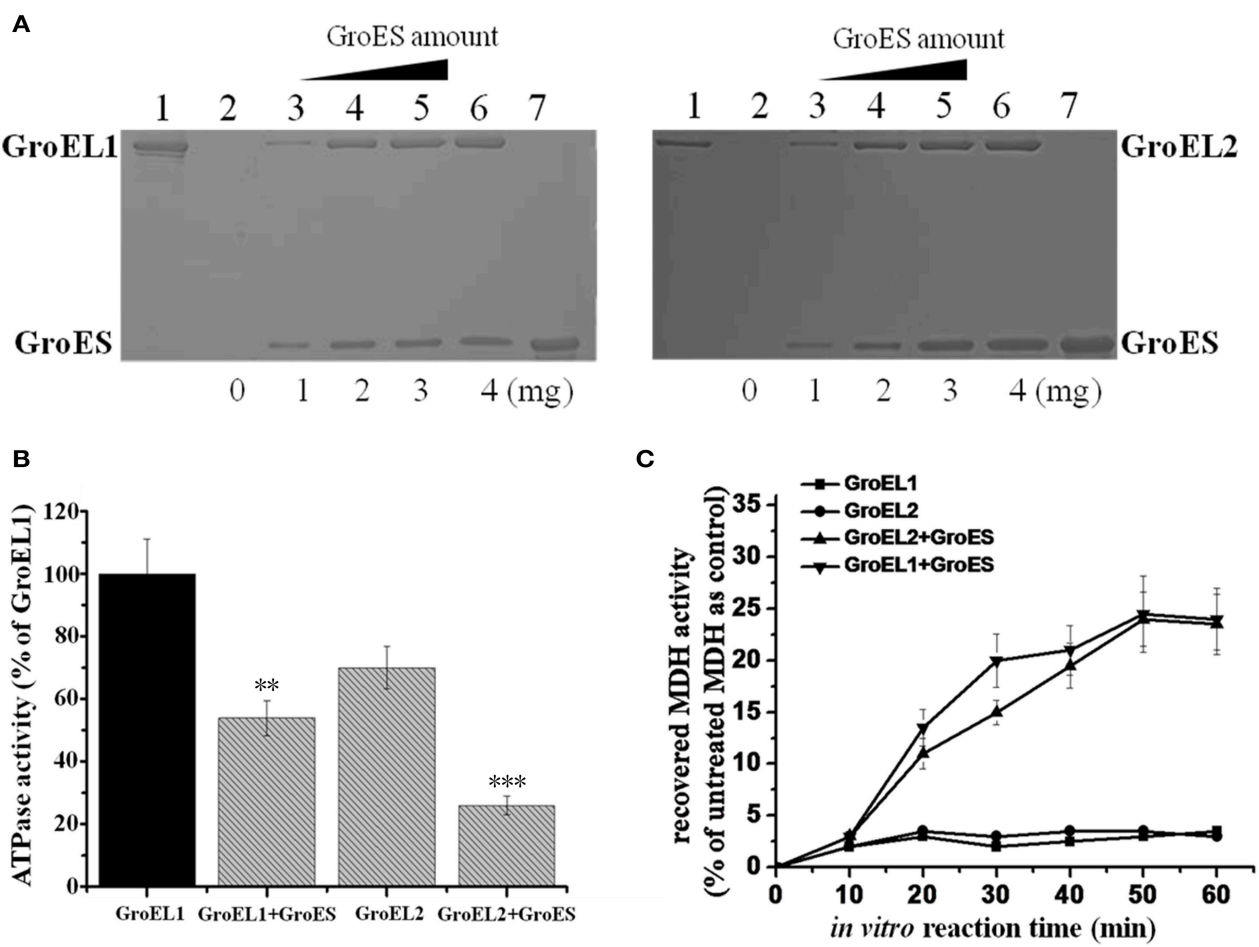
*****In vitro*** binding and refolding activities of GroELs and GroES from ***M. xanthus*** DK1622. (A)** GroEL1 (left) and GroEL2 (right) proteins bound by the His-tag GroES-containing nickel column. Bound GroEL-GroES complexes were eluted from the column with 500 mM imidazole and assayed by SDS-PAGE. Line 1, GroEL1 and GroEL2 proteins that passed through the His-tag GroES column; lines 2–6, increased His-tag GroES bound by the nickel column (0-4 mg) with the same amounts of GroEL proteins for each line; line 7, GroES control with no GroEL. **(B)** ATPase activities of GroELs in the absence or presence of GroES. For statistical analysis, ^***^P < 0.001 and ^**^P < 0.01, respectively. **(C)** MDH renaturation activities of GroELs in the absence or presence of GroES. Error bars in the pictures represent standard deviation of three time repeats.

To ascertain the relationship between the two GroELs and the single GroES protein of *M. xanthus* DK1622, we hetero-expressed these genes in *E. coli* cells and purified the target proteins. The *in vitro* binding activities of GroES and GroEL1/GroEL2 were assayed using two methods. We designed GroES with a His-tag at the C-terminus, which made the protein to be able to bind to a nickel column. The GroEL proteins had no His-tag and were unable to bind to the nickel column by themselves. When added to a GroES-containing column, either GroEL1 or GroEL2 was able to retain on the column (Figure 3A). As the concentration of the column-bound GroES increased, the recovery of GroEL proteins by the column also increased. Furthermore, GroEL is an ATPase, and binding to GroES reduces the ATPase activity of GroEL proteins (Martin et al., 1991; Viitanen et al., 1991). After the addition of GroES, the ATPase activities of the GroEL1 and GroEL2 proteins decreased to a similar extent (*P* < 0.001 and 0.001 < *P* < 0.01 for GroEL1 and GroEL2, respectively; Figure 3B). The above results confirmed the binding activities of both GroEL1 and GroEL2 to GroES *in vitro*. Based on the results in Figure 3B, GroEL1 showed higher *in vitro* ATPase activity than GroEL2 in the absence or presence of GroES, suggesting their disparate refolding activities in cells.

We assayed the *in vitro* refolding activities of the two GroELs with the malate dehydrogenase (MDH) protein. In the presence of GroEL1 or GroEL2, but without GroES, denatured MDH exhibited slight renaturation. When GroES was added, the MDH-folding activity of GroEL1 had a slightly higher rate than that of GroEL2 after 30 min (*P* < 0.05), but the final refolding activities were nearly identical (~25% re-natured MDH) for both GroEL1 and GroEL2 (Figure 3C). In addition, we also performed the refolding assay on citrate synthase (CS), which showed similar result as the MDH refolding (Supplementary Figure 3).

### GroES forms polymer complexes with either GroEL1 or GroEL2 and aids GroEL refolding functions *In vivo*

**Figure 4:**
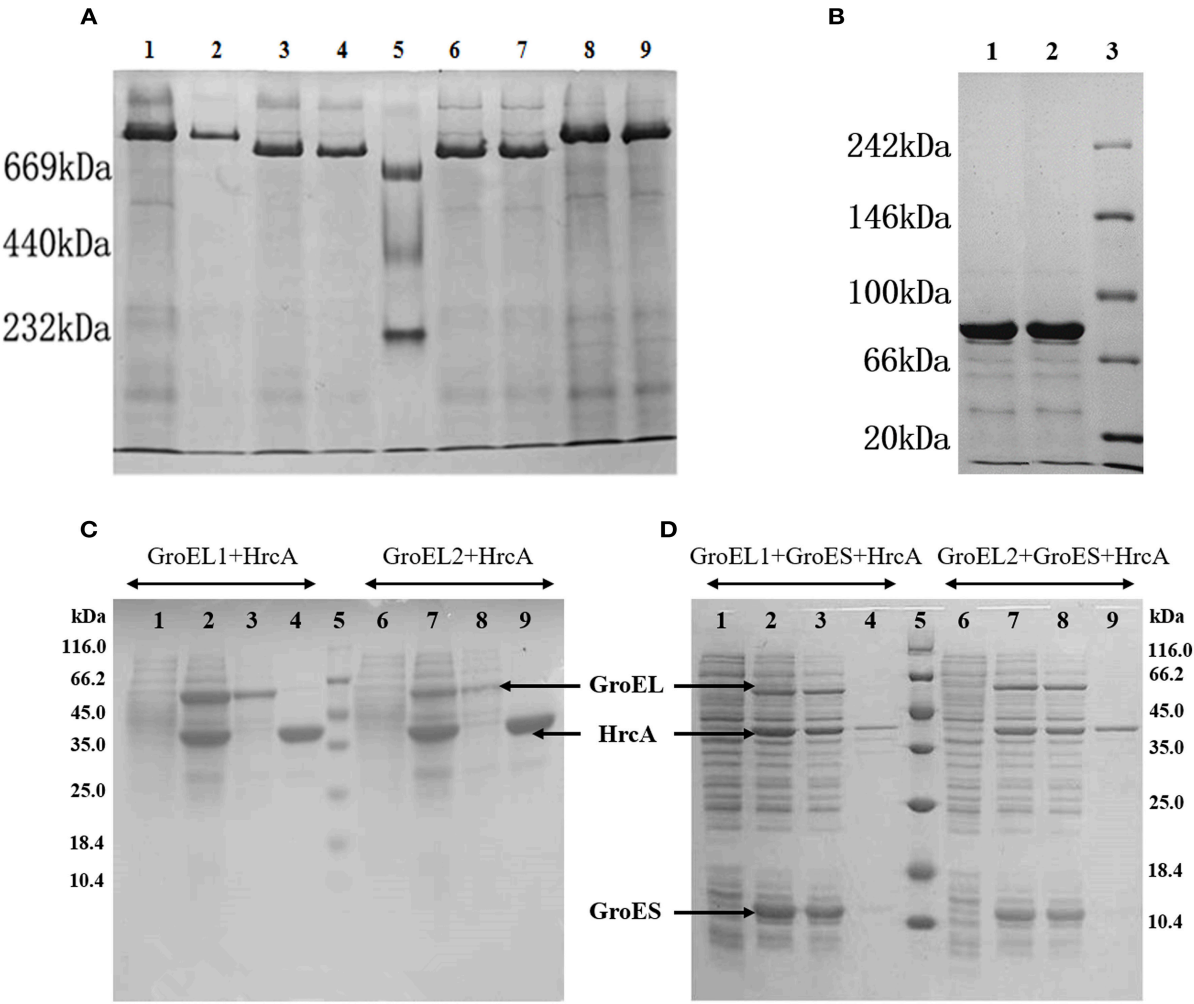
*****In vivo*** forms and refolding activities of GroELs and GroES proteins from ***M. xanthus*** DK1622 in ***E. coli*** cells. (A,B)** Native-PAGE analysis of extracts from *E. coli* cells expressing various arrays of DK1622 *groEL* and *groES* genes. **(A)** Lanes 1–2, co-expression of GroEL1 and GroES; lanes 3–4, expression of GroEL1 alone; lane 5, native protein markers; lanes 6–7, expression of GroEL2 alone; lanes 8–9, co-expression of GroEL2 and GroES. **(B)** Lanes 1–2, expression of GroES alone; lane 3, native protein markers. Each sample was repeated twice in gel for reliability of the Native-PAGE results. **(C,D)** SDS-PAGE analysis of co-expression of *hrcA* with *groEL1* (left) or *groEL2* (right) in the absence and presence of *groES* in *E. coli* cells, respectively. Lane 1, 6, before induction with IPTG; lane 2, 7, after induction; lane 3, 8, supernatants; lane 4, 9, sediments; lane 5, Mw markers. Ten microlitres were loaded for each lane.

We estimated the *in vivo* polymerization of the *Myxococcus* GroEL1 and GroEL2 proteins in *E. coli* cells in the presence or absence of the GroES. As the native-PAGE shown, the alone-expressed GroEL1 and GroEL2 proteins had a molecular weight (Mw) of ~800 kDa, respectively (the lanes 3–4 and 6–7 in Figure 4A). The molecular weight is about 14-times of that of the GroEL proteins (the Mws of GroEL1 and GroEL2 are 57.9 and 58.1 kDa, respectively, and their 14-mer polymers are 810.6 and 813.4 kDa, respectively). Similarly, the only-expressed GroES proteins formed a 7-mer complex (lanes 1–2 in Figure 4B; the Mw of GroES is 10.7 kDa, and the Mws of 7-mer GroES are 74.9 kDa). Co-polymers of GroEL and GroES were also observed in *E. coli* cells containing co-expressed *groEL1*/*groEL2* and *groES* genes of *M. xanthus* DK1622. The GroEL1-GroES and GroEL2-GroES complexes formed retarded bands with a similar molecular size (lanes 1–2 and 8–9 in Figure 4A; the Mws of 7-mer GroES plus 14-mer GroEL1 or 14-mer GroEL2 are 885.5 or 888.3 kDa, respectively). These results suggested that the individual GroEL1 and GroEL2 proteins both existed as 14-mer homopolymers, each of which was able to bind the 7-mer of GroES *in vivo*.

HrcA is a negative regulatory protein that is able to bind to the CIRCE (Controlling Inverted Repeat of Chaperone Expression) regions in front of the *groESL* operons and thus reduce the expression of *groEL* and *groES* genes (Wilson et al., 2005). The HrcA protein relies on GroEL to complete refolding and is a potential GroEL substrate. We assayed the *in vivo* refolding functions of the two GroEL proteins with or without GroES by expressing the *groEL, groES* and *hrcA* genes from *M. xanthus* in different combinations in *E. coli* cells. SDS-PAGE result indicated that in the absence of the DK1622 *groES* gene, HrcA primarily existed in the form of inclusion bodies in cells when co-expressed with only the *groEL1* or *groEL2* gene from *M. xanthus* (Figure 4C). However, when the DK1622 *groES* gene was further introduced into in the *E. coli* cells containing the *hrcA* and *groEL1*/*groEL2* genes from *M. xanthus*, the HrcA protein was then mostly soluble (Figure 4D). These results suggested that both GroEL1 and GroEL2 proteins from *M. xanthus* were able to aid the refolding of the HrcA proteins in the presence of the *M. xanthus* GroES in *E. coli* cells.

### *M. xanthus* cells coordinate expressions of single *groES* and duplicate *groEL*s

**Figure 5:**
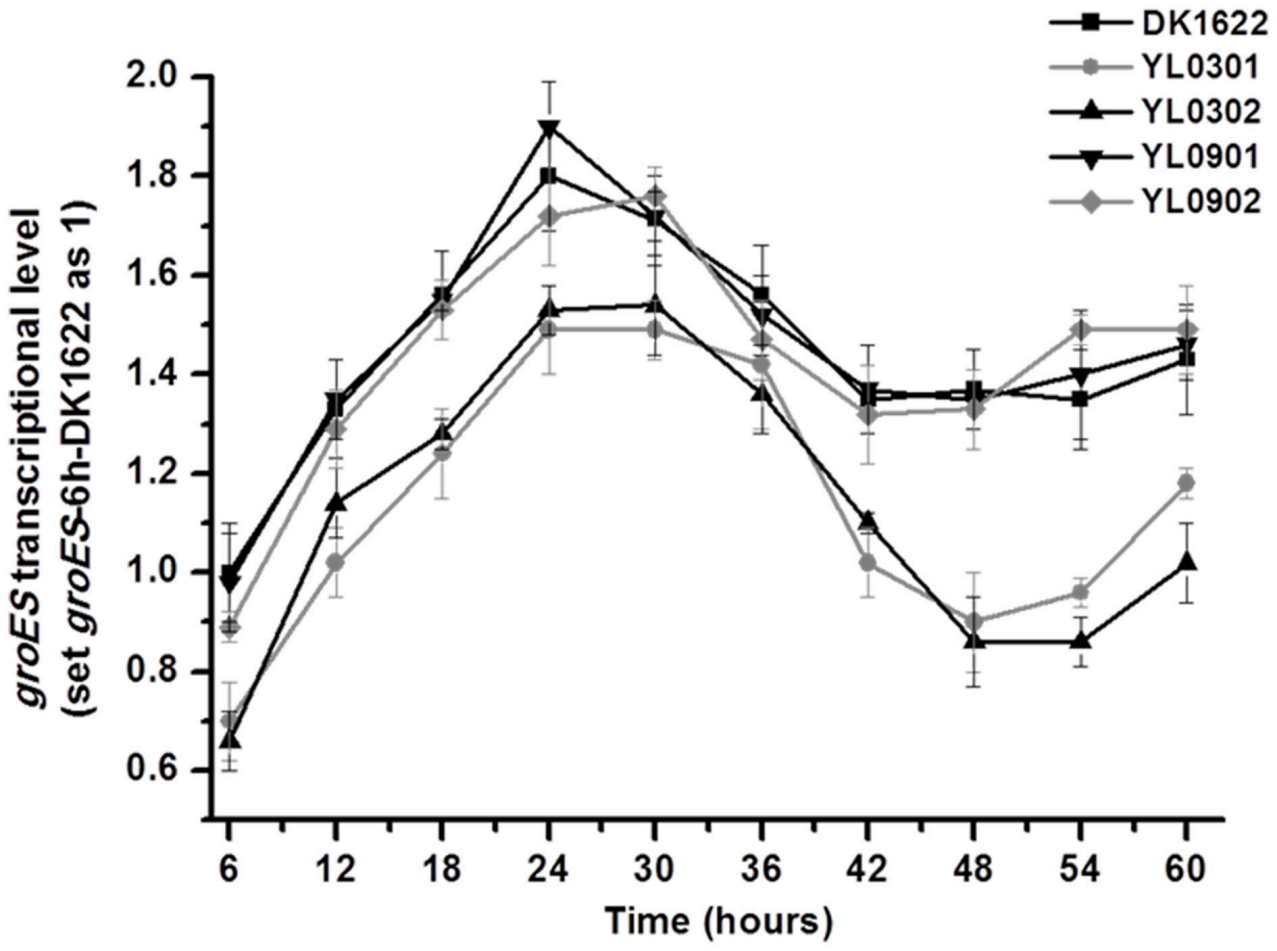
**Quantitative PCR analysis of ***groES*** gene expression levels in ***groEL*** mutants (YL0301 and YL0302), complementary mutants (YL0901 and YL0902), and the wild-type strain DK1622**. *groES* expression in DK1622 after 6 h incubation was set to one. Error bars represent standard deviation of three time repeats. DK1622, wild-type strain; YL0301, *groEL1*-deletion mutant; YL0302, *groEL2*-deletion mutant; YL0901, YL0301 complemented with *groEL1*; YL0902, YL0301 complemented with *groEL2*.

The above results indicated that either GroEL1 or GroEL2 requires GroES in order to function in *M. xanthus*. The two *groEL*s and the single *groES* had to coordinate their expressions for synergy. Our previous studies showed that the two *groEL*s had a balance of their expressions in *M. xanthus* cells (Li et al., 2010; Wang et al., 2013). In this study, we further assayed expressions of the *groES* gene in the wild type strain DK1622, the *groEL1/2* deletion mutants (YL0301 and YL0302) and their corresponding complementary strains (YL0901 and YL0902) by using quantitative PCR. In the wild type strain DK1622, *groES* gene expression increased and reached its maximum during the first 24 h of incubation in CTT medium. Then, the expression decreased and reached its lowest level after ~42–48 h of incubation (Figure 5). There was a slight increase in *groES* expression in the late stationary growth stage. This *groES* expression curve was highly consistent with that of the *groEL* genes in DK1622 (Li et al., 2010). Deletion of the *groEL2* gene, which has no neighboring *groES* gene, resulted in the decreased expression of the *groES* gene at each time point. This result is similar to those obtained following the deletion of the *groEL1* gene (Figure 5). When *groEL1* or *groEL2* with its own promoter was complemented ectopically at the *attB* site in the genome of the *groEL1* deletion mutant YL0301 (YL0901 and YL0902), the mutants recovered the total *groEL* expression levels comparable to that in *M. xanthus* DK1622 (Wang et al., 2013). Interestingly, the *groES* expression levels in YL0901 and YL0902 were also recovered to that in the wild type strain (Figure 5). These results suggested that there was a self-regulation mechanism to balance the expressions of the *groEL* and *groES* genes for their synergetic functions in *M. xanthus* cells.

### Over-expression of *groES* simultaneously raises both the *groEL1* and *groEL2* expressions

**Figure 6:**
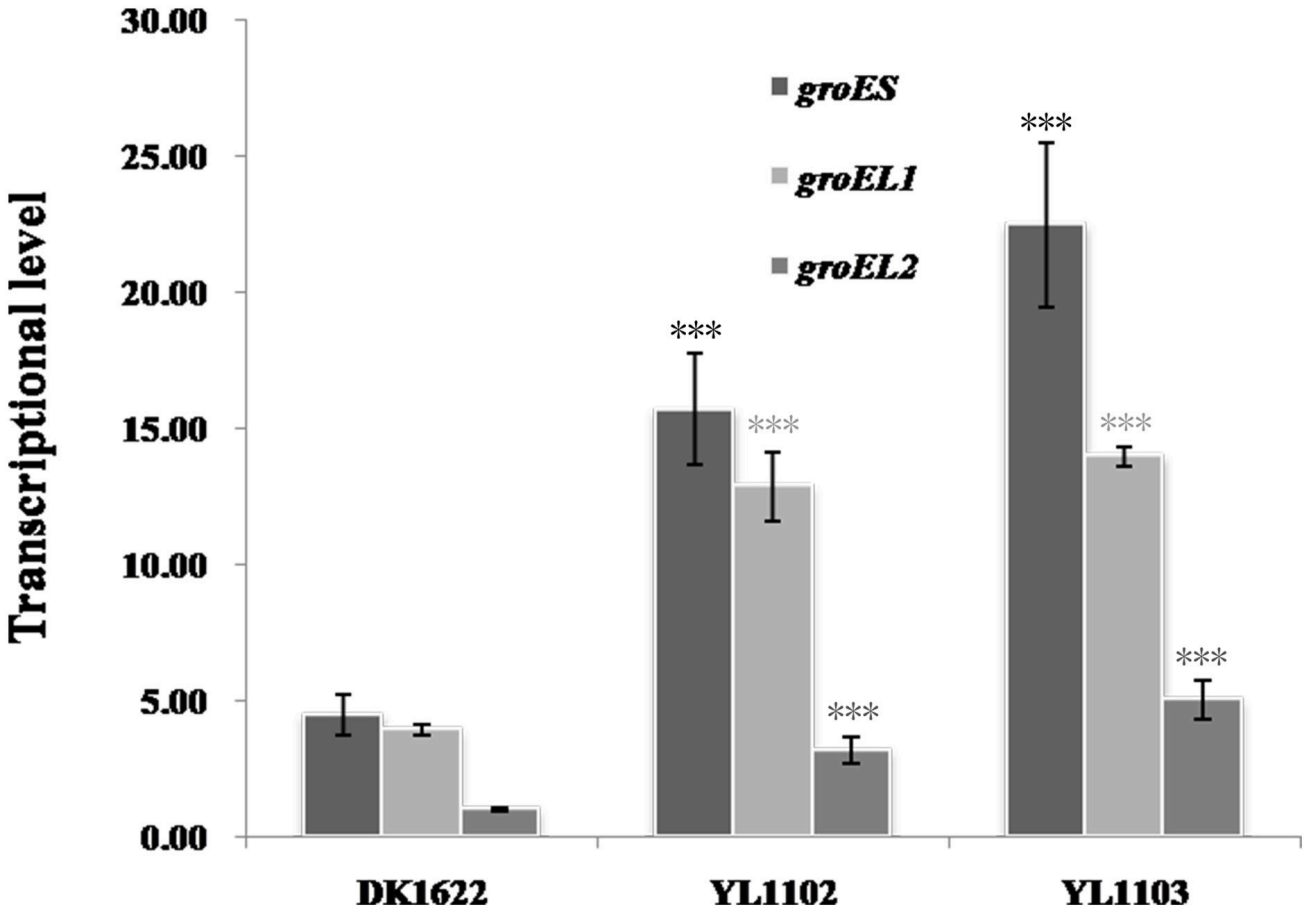
*****groES***, ***groEL1***, and ***groEL2*** transcription levels in the wild-type strain DK1622 and the ***groES*** over–expression mutants YL1102 and YL1103**. *groEL2* expression in DK1622 was set to one. Error bars represent standard deviation of three time repeats. For statistical analysis, ^***^P < 0.001. DK1622, wild-type strain; YL1102, over-expression mutant inserted the plasmid pZJY-*P*_*groEL*2_+*groES*; YL1103, over-expression mutant inserted the plasmid pZJY-*P*_*groEL*1_+*groES*.

In *M. xanthus* DK1622, the *groES* and *groEL1* genes are in an operon under the same promoter, while *groEL2* stands with a different promoter (Supplementary Figure 4 demonstrates their construction in genome). To confirm the possible coordinated expression and self-regulation of isolated *groEL* and *groES* genes, we further introduced an additional *groES* gene into DK1622 using the shuttle plasmid pZJY41 under the *groEL2* or *groEL1* promoter (referred to Supplementary Figure 4), forming the mutants YL1102 and YL1103, respectively. Compared with that in the wild-type strain DK1622, the expressions of *groES* markedly increased in both the YL1102 and YL1103 mutants (*P* < 0.001) and the *groEL1* promoter is stronger than the *groEL2* promoter (Figure 6). Interestingly, the expressions of either the *groEL1* or the *groEL2* genes also increased correspondingly (*P* < 0.001). The *groES* expression levels were comparable to the total expression changes of *groEL1* and *groEL2* in the two mutants. The results further demonstrated that the expression levels of the *groEL* and *groES* genes appeared to be self-regulated in *M. xanthus*, even when distributed in different places in the genome.

### Artificial *groESL2* operon has almost no effect on *groES* and *groEL* expressions

**Figure 7:**
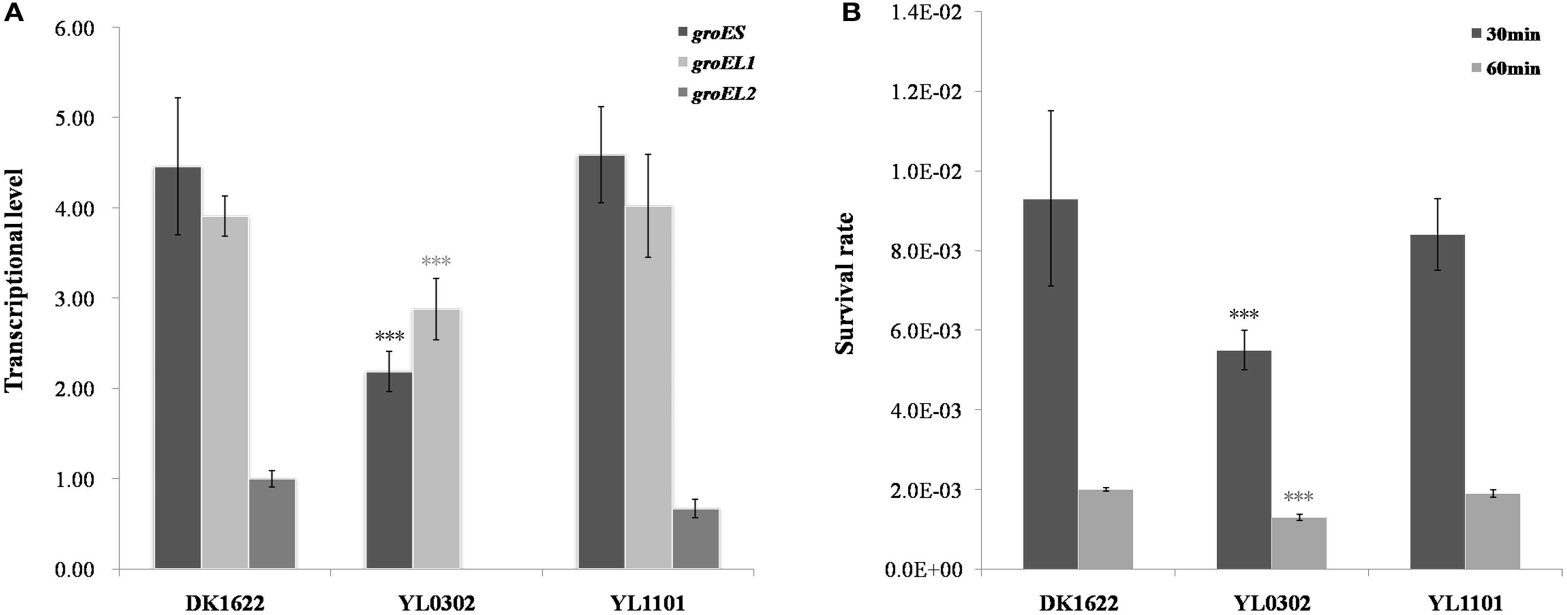
**Comparison of the wild-type strain DK1622 and the mutant containing an artificial ***groESL2*** operon (YL1101). (A)** *groES* and *groEL* transcriptional levels after 24 h of incubation in CTT liquid growth medium, with the *groEL2*-deletion mutant YL0302 as a control. *groEL2* expression in DK1622 was set to one. **(B)** Survival rates of DK1622 and YL1101 after heat-shock treatment at 42°C for 30 or 60 min, with the *groEL2*-deletion mutant YL0302 as a control. Error bars represent standard deviation of three time repeats. For statistical analysis, ^***^P < 0.001.

Stand-alone *groELs* are quite common in bacteria. In myxobacteria, the second *groEL* gene exists alone in the Cystobacterineae suborder but forms a complete *groESL* operon in the Sorangineae suborder (Figure 1). Based on our results, the stand-alone *groEL* gene functioned in a *groES*-dependent manner, at least in *M. xanthus* DK1622. We thus sought to determine whether an artificial *groESL2* operon was able to change the coordinated expression of *groEL* and *groES* genes in *M. xanthus*. We inserted a second copy of the *groES* gene in front of *groEL2* in DK1622, following the native *groEL2* promoter (Supplementary Figure 4) to form a complete bicistronic *groESL2* operon (mutant YL1101). The co-expression of *groEL2* and the inserted *groES* in the artificial *groESL* operon was verified by RT-PCR amplification (Supplementary Figure 2). However, the total expression levels of *groES*, as well as that of *groEL*s, in this mutant were still similar to those of DK1622 (*P* > 0.05); whereas the *groES* and *groEL1* expressions in the *groEL2* deletion mutant (YL0302) were both significantly decreased (*P* < 0.001; Figure 7A). The co-chaperonin GroES, in combination with the chaperonin GroEL, helps bacteria resist heat-shock conditions (Moparthi et al., 2013). We further assayed the heat-shock response of the *M. xanthus* cells containing two complete *groESL* operons. The survival rate of the YL1101 mutant after heat-shock treatment showed almost no differences from that of the wild-type strain DK1622 (*P* > 0.05); whereas the *groEL2* deletion mutant decreased the survival rate upon heat shock (*P* < 0.001; Figure 7B). The results demonstrated that the single *groES* gene was molecularly equivalent for the expressions and functions of the duplicate *groEL* genes in *M. xanthus* cells.

## Discussion

Classically, the genes encoding the chaperonin GroEL and its co-factor GroES are clustered in a bicistronic *groESL* operon in bacterial genomes. In bacteria possessing single *groEL* genes, *groES* is always present upstream *groEL* to form an operon, thus probably ensuring balanced expression for their synergetic functions. However, in genomes with two or more *groEL* genes, the duplicated *groEL* genes have been observed to stand alone with no neighboring *groES* gene. The occurrence of stand-alone *groEL* genes, which are often encountered in bacterial genomes, appeared to be characteristic of certain taxonomic units, such as the Cystobacterineae suborder of myxobacteria. Based on the genetic and biochemical analyses presented in this study, the single *groES* gene was indispensable for *M. xanthus* cells, and the stand-alone *groEL* gene still required *groES* in order to function. Generally, each of the GroEL1 and GroEL2 proteins was able to form a 14-mer complex, which further bound to a 7-mer polymer of the GroES protein *in vivo*, consistent with that in the bacteria possessing single *groEL* and *groES* genes (Weissman et al., 1995; Liu et al., 2009). In the absence of GroES, the DK1622 GroEL proteins were unable to refold denatured proteins correctly *in vitro* and *in vivo*. Thus, similar to GroEL1, GroEL2 depends on the presence of GroES to carry out its functions, even though the *groEL2* gene has lost its neighboring *groES* gene. However, as shown by our bioinformatics analysis, the presence of *groEL* and *groES* genes varies widely across prokaryotic genomes (Table 1). Although we determined that both GroEL proteins require the presence of GroES to carry out refolding in *M. xanthus* DK1622, it is unknown whether there are GroES-independent GroELs.

The results present in this paper showed that the *M. xanthus* cells are able to self-regulate expressions of the *groEL* and *groES* genes to meet the commensurable requirements for these proteins. For example, the deletion of *groEL1* or *groEL2* decreased not only *groEL* expression but also *groES* expression, while ectopic complementation of *groEL* recovered both *groEL* and *groES* expression in *M. xanthus* cells. Notably, the addition of an extra *groES* gene in front of *groEL2* to form an artificial *groESL2* operon had almost no effect on the expressions of *groES* and *groEL*s, but the over-expression of *groES* using the self-replicating plasmid pZJY41 increased *groEL* expressions considerably. This analysis indicated that the duplicate *groEL* and single *groES* genes have evolved to express in a coordinated manner for their synergistic functions in *M. xanthus* cells. The dependence of stand-alone *groEL* genes on non-neighbored *groES* is easily understandable in the context of the functional mechanisms of GroEL and its co-factor GroES. However, it is puzzled how the single *groES* gene coordinate its expression to meet the requirements of two endogenous *groEL* genes.

It is known that the HrcA protein is able to bind to the CIRCE regions in front of the *groESL* operons to negatively regulate expressions of the *groEL* and *groES* genes (Wilson et al., 2005). Wilson et al. reported that the GroEL protein, probably as well as the GroES protein was able to auto-regulate its own expression in *Chlamydia trachomatis* through direct interactions with the HrcA repressor protein (Wilson et al., 2005). Kong et al. observed an unbalanced expression of the operon-organized *groES* and *groEL* genes in *M. tuberculosis* (Kong et al., 1993). In *Salmonella*, differential expressions of bicistronic *groES* and *groEL* genes in a *groESL* operon were reported to be due to the mediation of an RNA thermometer in the *groES*-5′UTR, which regulated translation of *groES*, but not of *groEL* upon heat-shock (Cimdins et al., 2013). In addition, in *E. coli* cells, there is an imperfect transcriptional terminator in the intergenic region of the other major chaperonin gene operon *dnaKJ*, which was able to regulate the *dnaK* and *dnaJ* to express differentially (Bardwell et al., 1986). Herein, we proposed that the products of the *groEL*/*groES* genes involved in auto-regulation of expressions, and there were GroEL/GroES- dependent modulating sequences within or before the *groESL1* and *groEL2* operons for the coordinated expressions of the GroELs and GroES chaperonins in *M. xanthus*. The removal of *groES* from the duplicated ancient *groESL2* operon is likely an important evolutionary development. Thus, controlling the expressions of duplicate *groEL* and single *groES* genes under an integrated mechanism might be easier and more efficient for cells to adapt to the changing environment. The exact regulation mechanisms for the coordinated expressions of the *groES* and *groEL1*/*groEL2* genes in *M. xanthus* are undergoing investigation in our laboratory.

## Conclusion

In general, the genes encoding the chaperonins GroEL and GroES form a *groESL* operon. However, stand-alone *groEL* genes also exist broadly in the bacteria containing multiple *groEL* genes. The stand-alone *groEL*s may play functions independently or still dependently on *groES*. Here we prove that the stand-alone *groEL2* gene strictly relies on *groES* to function in *M. xanthus* DK1622. The duplicate *groEL* and the single *groES* genes were expressed and functioned interdependently in coordination. Adding an excess *groES* before *groEL2* had almost no influences on *groES* expression and the cell survival rate, and over-expression of *groES* increased the *groEL* expressions commensurably. The duplicated *groEL* and single *groES* genes thus have evolved an accurate self-regulation pattern for their cellular functions.

## Author contributions

Conceived and designed the experiments: YL, LZ, YW. Performed the experiments: LZ, YW, ZZ, JL. Analyzed the data: YL, LZ, YW, ZZ, XZ. Wrote the paper: YL, LZ, YW.

## Funding

This work was financially supported by the National Natural Science Foundation of China (NSFC) (No. 31471183 & No. 31670076) and the NSFC Key Program (No. 31130004) awarded to YL.

### Conflict of interest statement

The authors declare that the research was conducted in the absence of any commercial or financial relationships that could be construed as a potential conflict of interest.

## Supplementary material

Supplementary Figure 1 **The diagrammatic sketches for the construction of the plasmids of pET22b-HrcA (A)**, pBAD33-GroESL1/2, and pBAD33-GroEL1/2 **(B)**. The processing plasmids of pET22b-GroESL1/2 and pET22b-GroEL1/2 were constructed using the similar progress as pET22b-HrcA.

Supplementary Figure 2 **RT-PCR detection on the operon-organization of the native ***groES -groEL1*** locus and the artificial ***groES-groEL2*** locus in ***M. xanthus***. (A)** Lines 1, 4, PCR amplification of the *groES-groEL1* locus using the cDNA from DK1622 and YL1101 as template, respectively; Line 7, PCR amplification of the *groES-groEL2* locus using YL1101 cDNA as template; Lines 2, 5, 8, positive controls using the total DNA extracted from DK1622 or YL1101 as the template; Lines 3, 6, 9, negative controls in which no reverse transcriptase was added. M, Trans 2K Plus II markers. The cDNA templates were obtained by reverse transcription of the total RNA extracted from *M. xanthus* DK1622 or the mutant YL1101 using random primers. The strains were grown in CTT medium at 30°C for 24 h. **(B)** PCR amplification verification of the existence of *groES, groEL1* and *groEL2* genes in the cDNA samples from DK1622 and YL1101. Lines 1, 3, PCR amplification of *groES*. Lines 2, 4, PCR amplification of *groEL1*. Line 5, PCR amplification of *groEL2*. M, Trans 2K Plus II markers. The primers used for amplification are listed in Supplementary Table 2.

Supplementary Figure 3 **CS renaturation activities of GroELs in the absence or presence of GroES**. Error bars in the pictures represent standard deviation of three time repeats.

Supplementary Figure 4 **A diagrammatic sketch of the construction of the ***groES-groEL1*** operon and the ***groEL2*** gene in ***M. xanthus*** DK1622**. The promoters that were used to construct the YL1102 and YL1103 mutants containing excess *groES* genes are shown in the figure. The insertion of *groES* in the artificial *groES*-*groEL2* operon of YL1101 was between the *groEL2*-promoter and the *groEL2* gene.

Supplementary Table 1 **Bacterial strains and plasmids used in this study**.

Supplementary Table 2 **List of primers used in this study**.

Supplementary Table 3 **Copy numbers of ***groEL*** and ***groES*** in sequenced prokaryotic genomes**.

Supplementary Table 4 **The occurrence of ***groEL*** and ***groES*** genes in 24 sequenced myxobacterial genomes**.

## References

[B1] Bardwell J. C. Tilly K. Craig E. King J. Zylicz M. Georgopoulos C. (1986). The nucleotide sequence of the *Escherichia coli* K12 *dnaJ*+ gene. A gene that encodes a heat shock protein. J. Biol. Chem. 261, 1782–1785. 3003085

[B2] Cimdins A. Roßmanith J. Langklotz S. Bandow J. E. Narberhaus F. (2013). Differential control of Salmonella heat shock operons by structured mRNAs. Mol. Microbiol. 89, 715–731. 10.1111/mmi.12308 23802546

[B3] Craig E. A. Gambill B. D. Nelson R. J. (1993). Heat shock proteins: molecular chaperones of protein biogenesis. Microbiol. Rev. 57, 402–414. 8336673 10.1128/mr.57.2.402-414.1993 PMC372916

[B4] Dworkin M. Kaiser D. (1993). Myxobacteria II. Washington, DC: American Society for Microbiology.

[B5] Fayet O. Ziegelhoffer T. Georgopoulos C. (1989). The *groES* and *groEL* heat shock gene products of *Escherichia coli* are essential for bacterial growth at all temperatures. J. Bacteriol. 171, 1379–1385. 10.1128/jb.171.3.1379-1385.1989 2563997 PMC209756

[B6] Figueiredo L. Klunker D. Ang D. Naylor D. J. Kerner M. J. Georgopoulos C.. (2004). Functional characterization of an archaeal GroEL/GroES chaperonin system: significance of substrate encapsulation. J. Biol. Chem. 279, 1090–1099. 10.1074/jbc.M310914200 14576149

[B7] Goldman B. S. Nierman W. C. Kaiser D. Slater S. C. Durkin A. S. Eisen J. A.. (2006). Evolution of sensory complexity recorded in a myxobacterial genome. Proc. Natl. Acad. Sci. U.S.A. 103, 15200–15205. 10.1073/pnas.0607335103 17015832 PMC1622800

[B8] Gould P. S. Burgar H. R. Lund P. A. (2007). Homologous *cpn60* genes in *Rhizobium leguminosarum* are not functionally equivalent. Cell Stress Chaperones 12, 123–131. 10.1379/CSC-227R.1 17688191 PMC1949324

[B9] Goyal K. Qamra R. Mande S. C. (2006). Multiple gene duplication and rapid evolution in the *groEL* gene: functional implications. J. Mol. Evol. 63, 781–787. 10.1007/s00239-006-0037-7 17103057

[B10] Hodgkin J. Kaiser D. (1977). Cell-to-cell stimulation of movement in nonmotile mutants of *Myxococcus*. Proc. Natl. Acad. Sci. U.S.A. 74, 2938–2942. 10.1073/pnas.74.7.2938 16592422 PMC431354

[B11] Horwich A. L. Farr G. W. Fenton W. A. (2006). GroEL-GroES-mediated protein folding. Chem. Rev. 106, 1917–1930. 10.1021/cr040435v 16683761

[B12] Houry W. A. Frishman D. Eckerskorn C. Lottspeich F. Hartl F. U. (1999). Identification of *in vivo* substrates of the chaperonin GroEL. Nature 402, 147–154. 10.1038/45977 10647006

[B13] Jiang D. M. Zhao L. Zhang C. Y. Li J. Xia Z. J. Wang J.. (2008). Taxonomic analysis of Sorangium strains based on HSP60 and 16S rRNA gene sequences and morphology. Int. J. Syst. Evol. Microbiol. 58, 2654–2659. 10.1099/ijs.0.65806-0 18984709

[B14] Karlin S. Brocchieri L. (2000). Heat shock protein 60 sequence comparisons: duplications, lateral transfer, and mitochondrial evolution. Proc. Natl. Acad. Sci. U.S.A. 97, 11348–11353. 10.1073/pnas.97.21.11348 11027334 PMC17203

[B15] Kashefi K. Hartzell P. L. (1995). Genetic suppression and phenotypic masking of a *Myxococcus xanthus frzF-* defect. Mol. Microbiol. 15, 483–494. 10.1111/j.1365-2958.1995.tb02262.x 7783619

[B16] Kerner M. J. Naylor D. J. Ishihama Y. Maier T. Chang H. C. Stines A. P.. (2005). Proteome-wide analysis of chaperonin-dependent protein folding in *Escherichia coli*. Cell 122, 209–220. 10.1016/j.cell.2005.05.028 16051146

[B17] Klunker D. Haas B. Hirtreiter A. Figueiredo L. Naylor D. J. Pfeifer G.. (2003). Coexistence of group I and group II chaperonins in the archaeon *Methanosarcina mazei*. J. Biol. Chem. 278, 33256–33267. 10.1074/jbc.M302018200 12796498

[B18] Kong T. H. Coates A. R. Butcher P. D. Hickman C. J. Shinnick T. M. (1993). *Mycobacterium tuberculosis* expresses two chaperonin-60 homologs. Proc. Natl. Acad. Sci. U.S.A. 90, 2608–2612. 10.1073/pnas.90.7.2608 7681982 PMC46144

[B19] Kumar C. M. Mande S. C. Mahajan G. (2015). Multiple chaperonins in bacteria–novel functions and non-canonical behaviors. Cell Stress Chaperones 20, 555–574. 10.1007/s12192-015-0598-8 25986150 PMC4463927

[B20] Li J. Wang Y. Zhang C. Y. Zhang W. Y. Jiang D. M. Wu Z. H.. (2010). *Myxococcus xanthus* viability depends on *groEL* supplied by either of two genes, but the paralogs have different functions during heat shock, predation, and development. J. Bacteriol. 192, 1875–1881. 10.1128/JB.01458-09 20139189 PMC2838048

[B21] Liu H. Kovács E. Lund P. A. (2009). Characterisation of mutations in GroES that allow GroEL to function as a single ring. FEBS Lett. 583, 2365–2371. 10.1016/j.febslet.2009.06.027 19545569

[B22] Lobedanz S. Sogaard-Andersen L. (2003). Identification of the C-signal, a contact-dependent morphogen coordinating multiple developmental responses in *Myxococcus xanthus*. Genes Dev. 17, 2151–2161. 10.1101/gad.274203 12923062 PMC196456

[B23] Lund P. A. (2001). Microbial molecular chaperones. Adv. Microb. Physiol. 44, 93–140. 10.1016/S0065-2911(01)44012-4 11407116

[B24] Lund P. A. (2009). Multiple chaperonins in bacteria–why so many? FEMS Microbiol. Rev. 33, 785–800. 10.1111/j.1574-6976.2009.00178.x 19416363

[B25] Marchler-Bauer A. Bo Y. Han L. He J. Lanczycki C. J. Lu S.. (2017). CDD/SPARCLE: functional classification of proteins via subfamily domain architectures. Nucleic Acids Res. 45, D200–D203. 10.1093/nar/gkw1129 27899674 PMC5210587

[B26] Martin J. Langer T. Boteva R. Schramel A. Horwich A. L. Hartl F. U. (1991). Chaperonin-mediated protein folding at the surface of *groEL* through a “molten globule”-like intermediate. Nature 352, 36–42. 10.1038/352036a0 1676490

[B27] Moparthi S. B. Sjölander D. Villebeck L. Jonsson B. H. Hammarström P. Carlsson U. (2013). Transient conformational remodeling of folding proteins by GroES-individually and in concert with GroEL. J. Chem. Biol. 7, 1–15. 10.1007/s12154-013-0106-5 24386013 PMC3877409

[B28] Potnis A. A. Rajaram H. Apte S. K. (2016). GroEL of the nitrogen-fixing cyanobacterium *Anabaena* sp. strain L-31 exhibits GroES and ATP-independent refolding activity. J. Biochem. 159, 295–304. 10.1093/jb/mvv100 26449235

[B29] Qamra R. Srinivas V. Mande S. C. (2004). *Mycobacterium tuberculosis* GroEL homologues unusually exist as lower oligomers and retain the ability to suppress aggregation of substrate proteins. J. Mol. Biol. 342, 605–617. 10.1016/j.jmb.2004.07.066 15327959

[B30] Ranson N. A. White H. E. Saibil H. R. (1998). Chaperonins. Biochem. J. 333 (Pt 2), 233–242. 10.1042/bj3330233 9657960 PMC1219577

[B31] Saibil H. R. Ranson N. A. (2002). The chaperonin folding machine. Trends Biochem. Sci. 27, 627–632. 10.1016/S0968-0004(02)02211-9 12468232

[B32] Shimkets L. J. (1990). Social and developmental biology of the myxobacteria. Microbiol. Rev. 54, 473–501. 1708086 10.1128/mr.54.4.473-501.1990 PMC372790

[B33] Ueki T. Inouye S. Inouye M. (1996). Positive-negative KG cassettes for construction of multi-gene deletions using a single drug marker. Gene 183, 153–157. 10.1016/S0378-1119(96)00546-X 8996101

[B34] Vanbogelen R. A. Acton M. A. Neidhardt F. C. (1987). Induction of the heat shock regulon does not produce thermotolerance in *Escherichia coli*. Genes Dev. 1, 525–531. 10.1101/gad.1.6.525 3315852

[B35] Viitanen P. V. Donaldson G. K. Lorimer G. H. Lubben T. H. Gatenby A. A. (1991). Complex interactions between the chaperonin 60 molecular chaperone and dihydrofolate reductase. Biochemistry 30, 9716–9723. 10.1021/bi00104a021 1680394

[B36] Wang Y. Li X. Zhang W. Zhou X. Li Y. Z. (2014). The *groEL2* gene, but not *groEL1*, is required for biosynthesis of the secondary metabolite myxovirescin in *Myxococcus xanthus* DK1622. Microbiology 160, 488–495. 10.1099/mic.0.065862-0 24425771

[B37] Wang Y. Zhang W. Y. Zhang Z. Li J. Li Z. F. Tan Z. G.. (2013). Mechanisms involved in the functional divergence of duplicated GroEL chaperonins in *Myxococcus xanthus* DK1622. PLoS Genet. 9:e1003306. 10.1371/journal.pgen.1003306 23437010 PMC3578752

[B38] Weissman J. S. Hohl C. M. Kovalenko O. Kashi Y. Chen S. Braig K.. (1995). Mechanism of GroEL action: productive release of polypeptide from a sequestered position under GroES. Cell 83, 577–587. 10.1016/0092-8674(95)90098-5 7585961

[B39] Williams T. A. Fares M. A. (2010). The effect of chaperonin buffering on protein evolution. Genome Biol. Evol. 2, 609–619. 10.1093/gbe/evq045 20660109 PMC3296372

[B40] Wilson A. C. Wu C. C. Yates J. R. III. Tan M. (2005). Chlamydial GroEL autoregulates its own expression through direct interactions with the HrcA repressor protein. J. Bacteriol. 187, 7535–7542. 10.1128/JB.187.21.7535-7542.2005 16237037 PMC1272993

[B41] Zuo G. Hao B. (2015). CVTree3 Web Server for whole-genome-based and alignment-free prokaryotic phylogeny and taxonomy. Genomics Proteomics Bioinformatics 13, 321–331. 10.1016/j.gpb.2015.08.004 26563468 PMC4678791

