# Supplementary figures and images for: Myxococcus xanthus DK1622 Coordinates Expressions of the Duplicate groEL and Single groES Genes for Synergistic Functions of GroELs and GroES
Source: Front Microbiol. 2017 Apr 27;8:733. doi: 10.3389/fmicb.2017.00733 (PMC5406781; doi:10.3389/fmicb.2017.00733)

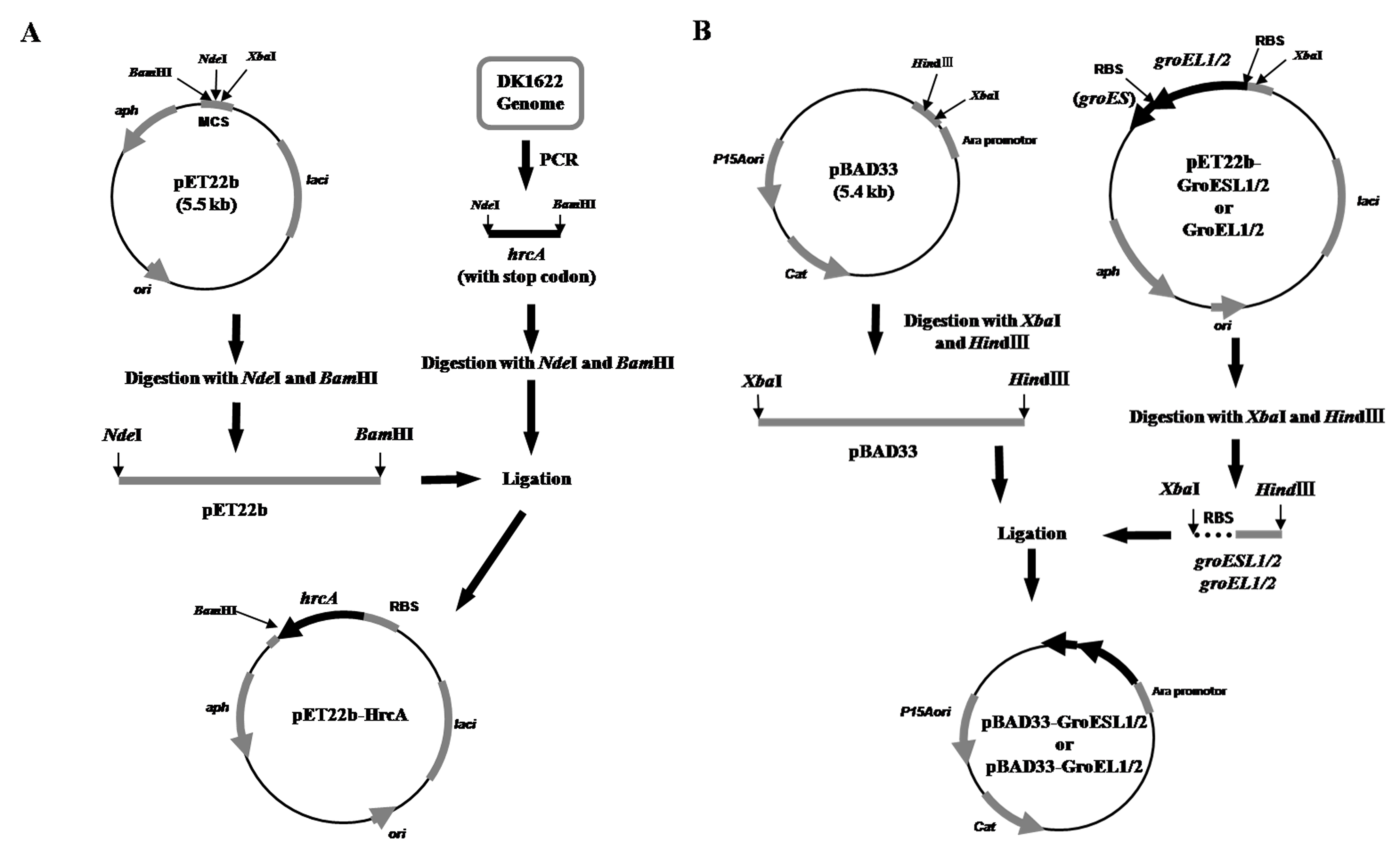

Supplement: Supplementary Figure 1 — The diagrammatic sketches for the construction of the plasmids of pET22b-HrcA (A), pBAD33-GroESL1/2, and pBAD33-GroEL1/2 (B). The processing plasmids of pET22b-GroESL1/2 and pET22b-GroEL1/2 were constructed using the similar progress as pET22b-HrcA. [file Image1.TIF]

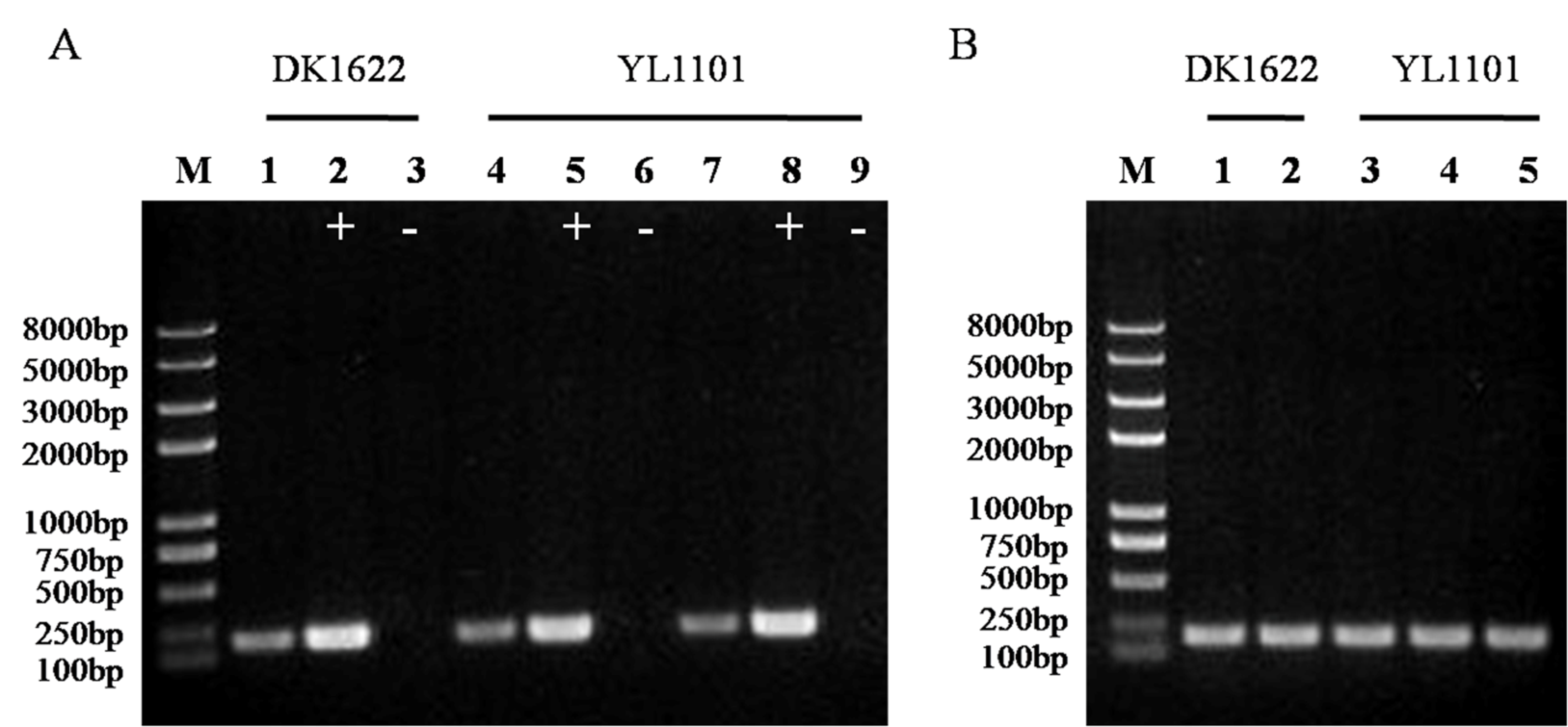

Supplement: Supplementary Figure 2 — RT-PCR detection on the operon-organization of the native groES -groEL1 locus and the artificial groES-groEL2 locus in M. xanthus. (A) Lines 1, 4, PCR amplification of the groES-groEL1 locus using the cDNA from DK1622 and YL1101 as template, respectively; Line 7, PCR amplification of the groES-groEL2 locus using YL1101 cDNA as template; Lines 2, 5, 8, positive controls using the total DNA extracted from DK1622 or YL1101 as the template; Lines 3, 6, 9, negative controls in which no reverse transcriptase was added. M, Trans 2K Plus II markers. The cDNA templates were obtained by reverse transcription of the total RNA extracted from M. xanthus DK1622 or the mutant YL1101 using random primers. The strains were grown in CTT medium at 30°C for 24 h. (B) PCR amplification verification of the existence of groES, groEL1 and groEL2 genes in the cDNA samples from DK1622 and YL1101. Lines 1, 3, PCR amplification of groES. Lines 2, 4, PCR amplification of groEL1. Line 5, PCR amplification of groEL2. M, Trans 2K Plus II markers. The primers used for amplification are listed in Supplementary Table 2. [file Image2.tif]

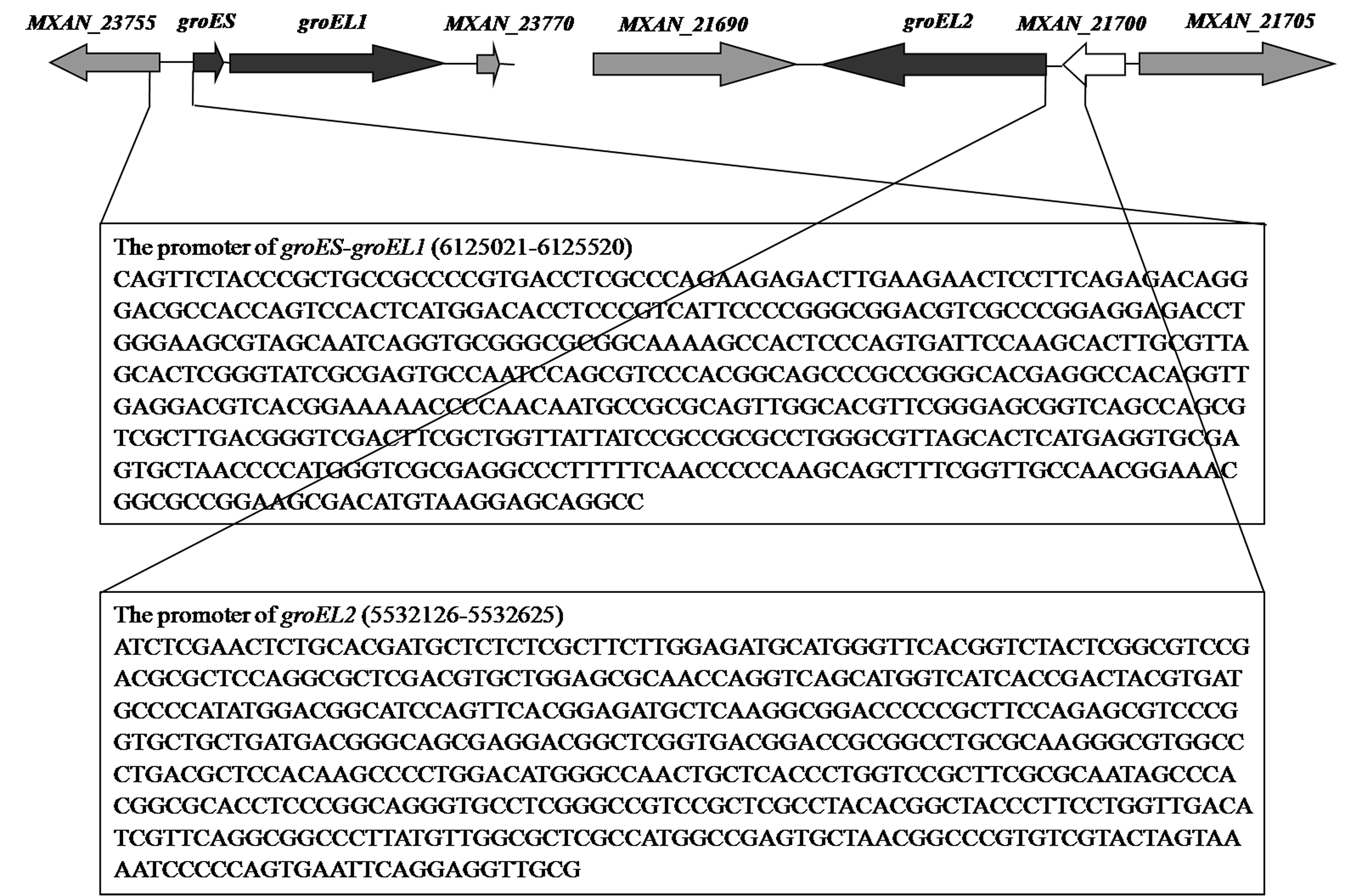

Supplement: Supplementary Figure 4 — A diagrammatic sketch of the construction of the groES-groEL1 operon and the groEL2 gene in M. xanthus DK1622. The promoters that were used to construct the YL1102 and YL1103 mutants containing excess groES genes are shown in the figure. The insertion of groES in the artificial groES-groEL2 operon of YL1101 was between the groEL2-promoter and the groEL2 gene. [file Image4.TIF]
