# Supplementary material for: Myxococcus xanthus DK1622 Coordinates Expressions of the Duplicate groEL and Single groES Genes for Synergistic Functions of GroELs and GroES
Source: Front Microbiol. 2017 Apr 27;8:733. doi: 10.3389/fmicb.2017.00733 (PMC5406781; doi:10.3389/fmicb.2017.00733)
Supplement: Supplementary Table 1 — Bacterial strains and plasmids used in this study. [file Table1.PDF]

Supplementary Table S1. Bacterial strains and plasmids used in this study.

Strain and plasmid	Genotype or description	Source or reference
Strains		
M. xanthus		
DK1622	Wild-type strain	D.Kaiser University of Standford (D Kaiser, 1979)
YL0308	DK1622:: pSWU-*groES*	This study
YL0309	DK1622::pSWU-*groES*Δ*MXAN_RS23760*	This study
YL1101	YL0302:: pSWU-P_{groEL2}+*groES*+*groEL2*	This study
YL1102	DK1622:: pZJY-P_{groEL2}+*groES*	This study
YL1103	DK1622:: pZJY-P_{groEL1}+*groES*	This study
YL0901	YL0301::pSWU-*groEL1*(*groEL1*integrated at *attB* site with its own promoter)	Wang et al., 2013
YL0902	YL0301::pSWU-*groEL2*(*groEL2*integrated at *attB* site with its own promoter)	Wang et al., 2013
YL0301	DK1622*ΔMXAN_RS23765*(*groEL1* deletion)	Li et al., 2010
YL0302	DK1622Δ*MXAN_RS21695*(*groEL*2 deletion)	Li et al., 2010
E. coli		
BL21(DE3)	F^- *ompT hsdS*$_B$($r_B^-m_B^-$)*gal dcm* (DE3)	Stratagene
XL1-Blue MR	Δ(*mcrA*)183Δ(*mcrCB-hsdSMR-mrr*)*173 endA1 supE44 thi-1 recA1 gyrA96 relA1 lac*	Stratagene
Top 10F’	F'[*lacIq Tn10*(*tetR*)] *mcrA* Δ(*mrr-hsdRMS-mcrBC*) φ80*lacZ* Δ*M15*Δ*lacX74 deoR nupG recAl araD139* Δ(*ara-leu*)*7697 galU galK rpsL*(*StrR*) *endAl λ-*	Stratagene
Plasmids		
pBJ113	Gene replacement vector with KG cassette; Km^r	Laboratory collection
pBJ-*groES*	Upstream and downstream homologous arms of DK1622 *MXAN_RS23760*(*groES*) and inserted into SmaI of pBJ113,Km^r	This study

pBJ-*groESL1*	Upstream and downstream homologous arms of DK1622 *MXAN_RS23760* - *MXAN_RS23765*(*groESL1*) and inserted into SmaI of pBJ113,Km^r	This study
pSWU30	Site-specific integration vector with *Mx8 attB* integration site; Tet^r	Tâm Mignot, CNRS(Centre national de la recherche scientifique)
pSWU-*groES*	300bp fragment of *MXAN_RS23760*（*groES*）with its upstream 1kb *pilA* promotor sequence and inserted into XbaI/EcoRI of pSWU30, Tet^r	This study
pSWU-P_{groEL2}+*groES*+*groEL2*	500bp promoter of *MXAN_RS21695*（*groEL2*）and *MXAN_RS23760*（*groES*） with *MXAN_RS21695*（*groEL2*）, inserted into XbaI/EcoRI of pSWU30, Tet^r	This study
pZJY41	Amp^r, Km^r, the stable *Myxococcus-E. coli* shuttle plasmid	Zhao et al., 2008
pZJY- P_{groEL2}+*groES*	500bp promoter of *MXAN_RS21695*（*groEL2*）with *MXAN_RS23760*（*groES*） sequence and inserted into BamHI/EcoRI of pZJY41, Amp^r, Km^r	This study
pZJY-P_{groEL1}+*groES*	500bp promoter of *MXAN_RS23765*（*groEL1*）with *MXAN_RS23760*（*groES*） sequence and inserted into BamHI/EcoRI of pZJY41, Amp^r, Km^r	This study
pET22b	Expression vector, Amp^r, C-His·tag, T7 promotor	Qi Q.S. Shandong university
pET22b-GroEL1-NoHis	No His tagged *groEL1* insertion in pET22b	This study
pET22b-GroEL2-NoHis	No His tagged *groEL2* insertion in pET22b	This study
pET22b-GroES	C-His tagged *groES* insertion in pET22b	This study
pET22b-HrcA	C-His tagged *hrcA* insertion in pET22b	This study
pET28a	Expression vector, Km^r, N-His·tag, C-	Qi Q.S. Shandong

	His·tag, T7 promotor	university
pET28a-GroES-NoHis	No His tagged *groES* insertion in pET28a	This study
pBAD33	Cloning vector，Cm^r，Ara promotor，p15A *ori*	Zhang C.C. Marseille University
pBAD33-GroESL1	No His tagged *groESL1* insertion in pBAD33	This study
pBAD33-GroESL2	No His tagged *groESL2* insertion in pBAD33	This study
pBAD33-GroEL1	No His tagged *groEL1* insertion in pBAD33	This study
pBAD33-GroEL2	No His tagged *groEL2* insertion in pBAD33	This study

References:

D Kaiser. (1979). Social gliding is correlated with the presence of pili in *Myxococcus xanthus*. *Proc Natl Acad Sci.* 76, 5952-5956.

Li, J., Wang, Y., Zhang, C.Y., Zhang, W.Y., Jiang, D.M., Wu, Z.H., Liu, H., and Li, Y.Z. (2010). *Myxococcus xanthus* viability depends on *groEL* supplied by either of two genes, but the paralogs have different functions during heat shock, predation, and development. *J Bacteriol* 192**,** 1875-1881.

Wang, Y., Zhang, W.Y., Zhang, Z., Li, J., Li, Z.F., Tan, Z.G., Zhang, T.T., Wu, Z.H., Liu, H., and Li, Y.Z. (2013). Mechanisms involved in the functional divergence of duplicated GroEL chaperonins in *Myxococcus xanthus* DK1622. *PLoS Genet* 9**,** e1003306.

Zhao, J.Y., Zhong, L., Shen, M.J., Xia, Z.J., Cheng, Q.X., Sun, X., Zhao, G.P., Li, Y.Z., and Qin, Z.J. (2008). Discovery of the autonomously replicating plasmid pMF1 from *Myxococcus fulvus* and development of a gene cloning system in *Myxococcus xanthus*. *Appl Environ Microbiol* 74**,** 1980-1987.
