# Supplementary material for: Myxococcus xanthus DK1622 Coordinates Expressions of the Duplicate groEL and Single groES Genes for Synergistic Functions of GroELs and GroES
Source: Front Microbiol. 2017 Apr 27;8:733. doi: 10.3389/fmicb.2017.00733 (PMC5406781; doi:10.3389/fmicb.2017.00733)
Supplement: Supplementary Table 2 — List of primers used in this study. [file Table2.PDF]

Supplementary Table S2. List of primers used in this study.

Primer	Sequence(5-3)*	Use
groES-KO-1-1 -for	CGCACGTGAAGCCGTAGATG	Amplification of upstream homologous arm for deletion of *groES*
groES-KO-1-1-rev	CGGGATCCGGCCTGCTCCTTACA TGTCG	
groES-KO-1-2-for	CGGGATCCTCCGGCTTCCCCCAC TCTTC	Amplification of downstream homologous arm for deletion of *groES*
groES-KO-1-2-rev	CGGTCGTAGTCGCTGGAGGTCT	
groES-KO-2-1-for	TCGTAGGGCTTGAGCCTGTCG	Amplification of upstream homologous arm for deletion of *groES*
groES-KO-2-1-rev	CGCGGATCCAATCTTCATGGCCT GCTCCTTAC	
groES-KO-2-2-for	CGCGGATCCGAGGATGTGCTCG GCGTGAT	Amplification of downstream homologous arm for deletion of *groES*
groES-KO-2-2-rev	CGGTCGTAGTCGCTGGAGGT	
groES-E-for	GGGAATTCCATATGAAGATTC GTCCCCTGCAG	PCR amplification of *groES*
groES-E-rev	CGCGGATCCTTACTTCTCGATC ACGCCGAGC	
groES-CKO -for	CAACCTTCTCTGAGGACCCCCA TGAAGATTCGTCCCCTGCA	PCR amplification of *groES* for fusion with promotor of *pilA*
groES-CKO -rev	CGGAATTCTTACTTCTCGATCA CGCCGA	
pliA-P-for	CTAGTCTAGAGCGGCGTTGAA CGAGGGG	PCR amplification of promotor of *pilA* for expression of *groES*
pliA-P-rev	GGGGGTCCTCAGAGAAGGTTG C	
groES-ex-up	TCGCCGAGGAGAACAA	Transcriptional level detection of *groES*
groES-ex-down	GATCACGCCGAGCACA	
groEL1-ex-up	CAAGGACGGTGTGACGGTCG	Transcriptional level detection of *groEL1*
groEL1-ex-down	CCTTGTCGATGCCGCGCTTG	
groEL2 -ex-up	GAAGAGCTTCGGCTCCCCCA	Transcriptional level detection of *groEL2*
groEL2-ex-down	TTGAGGTCCATCGGGCTGTG	
16s-ex-up	CGGCGTGACAAGTCGGGTGTG AAAG	Reference gene for transcriptional level detection
16s-ex-down	CGTCTCAGCGTCAGTTACCGTC CAG	
groESL1-up	GGCAAGGTGCAGGAGGACG	PCR amplification of *groES*-

groESL1-down	CGCACGTCGAAAAGAATGTCC	*groEL1* locus
groESL2-up	GGCAAGGTGCAGGAGGACG	PCR amplification of *groES*-*groEL2* locus in YL1101
groESL2-down	GATGGAAGAAAATTTCCTTCGC	
