# Supplementary material for: Myxococcus xanthus DK1622 Coordinates Expressions of the Duplicate groEL and Single groES Genes for Synergistic Functions of GroELs and GroES
Source: Front Microbiol. 2017 Apr 27;8:733. doi: 10.3389/fmicb.2017.00733 (PMC5406781; doi:10.3389/fmicb.2017.00733)
Supplement: Supplementary Table 3 — Copy numbers of groEL and groES in sequenced prokaryotic genomes. [file Table3.PDF]

Supplementary Table S3. Copy numbers of *groEL* and *groES* in sequenced prokaryotic genomes.

GroEL	GroES	Group	Organism name
1	1	Acidobacteria	*Acidobacterium capsulatum ATCC 51196*
1	1	Acidobacteria	*Candidatus Koribacter versatilis Ellin345*
1	2	Acidobacteria	*Candidatus Solibacter usitatus Ellin6076*
1	1	Acidobacteria	*Chloracidobacterium thermophilum B*
1	1	Acidobacteria	*Granulicella mallensis MP5ACTX8*
1	1	Acidobacteria	*Granulicella tundricola MP5ACTX9*
1	2	Acidobacteria	*Terriglobus roseus DSM 18391*
1	1	Acidobacteria	*Terriglobus saanensis SP1PR4*
1	1	Aquificae	*Aquifex aeolicus VF5*
1	1	Aquificae	*Desulfurobacterium thermolithotrophum DSM 11699*
1	1	Aquificae	*Hydrogenobacter thermophilus TK-6*
1	1	Aquificae	*Hydrogenobacter thermophilus TK-6*
1	1	Aquificae	*Hydrogenobaculum sp. 3684*
1	1	Aquificae	*Hydrogenobaculum sp. HO*
1	1	Aquificae	*Hydrogenobaculum sp. SHO*
1	1	Aquificae	*Hydrogenobaculum sp. SN*
1	1	Aquificae	*Hydrogenobaculum sp. Y04AAS1*
1	2	Aquificae	*Persephonella marina EX-H1*
1	1	Aquificae	*Sulfurihydrogenibium azorense Az-Fu1*
1	1	Aquificae	*Sulfurihydrogenibium sp. YO3AOP1*
1	1	Aquificae	*Thermocrinis albus DSM 14484*
1	1	Aquificae	*Thermocrinis ruber*
1	1	Aquificae	*Thermovibrio ammonificans HB-1*
1	1	Caldiserica	*Caldisericum exile AZM16c01*
1	1	Chrysiogenetes	*Desulfurispirillum indicum S5*
1	1	Deferribacteres	*Calditerrivibrio nitroreducens DSM 19672*

1	1	Deferribacteres	*Deferribacter desulfuricans SSM1*
1	1	Deferribacteres	*Denitrovibrio acetiphilus DSM 12809*
1	1	Deferribacteres	*Flexistipes sinusarabici DSM 4947*
1	2	Dictyoglomi	*Dictyoglomus thermophilum H-6-12*
1	2	Dictyoglomi	*Dictyoglomus turgidum DSM 6724*
1	1	Elusimicrobia	*Elusimicrobium minutum Pei191*
1	1	Elusimicrobia	*Endomicrobium proavitum*
1	1	Elusimicrobia	*uncultured Termite group 1 bacterium phylotype Rs-D17*
0	0	Euryarchaeota	*Aciduliprofundum boonei T469*
0	0	Euryarchaeota	*Aciduliprofundum sp. MAR08-339*
0	0	Euryarchaeota	*Archaeoglobus fulgidus DSM 4304*
0	0	Euryarchaeota	*Archaeoglobus fulgidus DSM 8774*
0	0	Euryarchaeota	*Archaeoglobus profundus DSM 5631*
0	0	Euryarchaeota	*Archaeoglobus sulfaticallidus PM70-1*
0	0	Euryarchaeota	*Archaeoglobus veneficus SNP6*
0	0	Euryarchaeota	*Candidatus Methanomassiliicoccus intestinalis Issoire-Mx1*
0	0	Euryarchaeota	*Candidatus Methanomethylophilus alvus Mx1201*
0	0	Euryarchaeota	*Candidatus Methanoplasma termitum*
0	0	Euryarchaeota	*Ferroglobus placidus DSM 10642*
0	0	Euryarchaeota	*Ferroplasma acidarmanus fer1*
0	0	Euryarchaeota	*Geoglobus acetivorans*
0	0	Euryarchaeota	*Geoglobus ahangari*
0	0	Euryarchaeota	*Halalkalicoccus jeotgali B3*
0	0	Euryarchaeota	*Halanaeroarchaeum sulfurireducens*
0	0	Euryarchaeota	*Halanaeroarchaeum sulfurireducens*
0	0	Euryarchaeota	*Haloarcula hispanica ATCC 33960*
0	0	Euryarchaeota	*Haloarcula hispanica N601*
0	0	Euryarchaeota	*Haloarcula marismortui ATCC 43049*
0	0	Euryarchaeota	*Haloarcula sp. CBA1115*

0	0	Euryarchaeota	*Halobacterium hubeiense*
0	0	Euryarchaeota	*Halobacterium salinarum NRC-1*
0	0	Euryarchaeota	*Halobacterium salinarum R1*
0	0	Euryarchaeota	*Halobacterium sp. DL1*
0	0	Euryarchaeota	*Haloferax gibbonsii*
0	0	Euryarchaeota	*Haloferax mediterranei ATCC 33500*
0	0	Euryarchaeota	*Haloferax volcanii DS2*
0	0	Euryarchaeota	*Halogeometricum borinquense DSM 11551*
0	0	Euryarchaeota	*Halomicrobium mukohataei DSM 12286*
0	0	Euryarchaeota	*Halopiger xanaduensis SH-6*
0	0	Euryarchaeota	*Haloquadratum walsbyi C23*
0	0	Euryarchaeota	*Haloquadratum walsbyi DSM 16790*
0	0	Euryarchaeota	*Halorhabdus tiamatea SARL4B*
0	0	Euryarchaeota	*Halorhabdus utahensis DSM 12940*
0	0	Euryarchaeota	*Halorubrum lacusprofundi ATCC 49239*
0	0	Euryarchaeota	*Halostagnicola larsenii XH-48*
0	0	Euryarchaeota	*Haloterrigena turkmenica DSM 5511*
0	0	Euryarchaeota	*Methanobacterium formicicum*
0	0	Euryarchaeota	*Methanobacterium formicicum*
0	0	Euryarchaeota	*Methanobacterium formicicum*
0	0	Euryarchaeota	*Methanobacterium lacus*
0	0	Euryarchaeota	*Methanobacterium paludis*
0	0	Euryarchaeota	*Methanobacterium sp. MB1*
0	0	Euryarchaeota	*Methanobrevibacter millerae*
0	0	Euryarchaeota	*Methanobrevibacter ruminantium M1*
0	0	Euryarchaeota	*Methanobrevibacter smithii ATCC 35061*
0	0	Euryarchaeota	*Methanobrevibacter sp. AbM4*
0	0	Euryarchaeota	*Methanocaldococcus bathoardescens*
0	0	Euryarchaeota	*Methanocaldococcus fervens AG86*

0	0	Euryarchaeota	*Methanocaldococcus infernus ME*
0	0	Euryarchaeota	*Methanocaldococcus sp. FS406-22*
0	0	Euryarchaeota	*Methanocaldococcus vulcanius M7*
0	0	Euryarchaeota	*Methanocella arvoryzae MRE50*
0	0	Euryarchaeota	*Methanocella conradii HZ254*
0	0	Euryarchaeota	*Methanococcoides burtonii DSM 6242*
0	0	Euryarchaeota	*Methanococcoides methylutens MM1*
0	0	Euryarchaeota	*Methanococcus aeolicus Nankai-3*
0	0	Euryarchaeota	*Methanococcus maripaludis C5*
0	0	Euryarchaeota	*Methanococcus maripaludis C6*
0	0	Euryarchaeota	*Methanococcus maripaludis C7*
0	0	Euryarchaeota	*Methanococcus maripaludis S2*
0	0	Euryarchaeota	*Methanococcus maripaludis X1*
0	0	Euryarchaeota	*Methanococcus vannielii SB*
0	0	Euryarchaeota	*Methanococcus voltae A3*
0	0	Euryarchaeota	*Methanocorpusculum labreanum Z*
0	0	Euryarchaeota	*Methanoculleus bourgensis MS2*
0	0	Euryarchaeota	*Methanoculleus marisnigri JR1*
0	0	Euryarchaeota	*Methanoculleus sp. MAB1*
0	0	Euryarchaeota	*Methanohalobium evestigatum Z-7303*
0	0	Euryarchaeota	*Methanohalophilus mahii DSM 5219*
0	0	Euryarchaeota	*Methanolacinia petrolearia DSM 11571*
0	0	Euryarchaeota	*Methanolinea tarda NOBI-1*
0	0	Euryarchaeota	*Methanolobus psychrophilus R15*
0	0	Euryarchaeota	*Methanomethylovorans hollandica DSM 15978*
0	0	Euryarchaeota	*Methanoregula boonei 6A8*
0	0	Euryarchaeota	*Methanoregula formicica SMSP*
0	0	Euryarchaeota	*Methanosaeta concilii GP6*
0	0	Euryarchaeota	*Methanosaeta harundinacea 6Ac*

0	0	Euryarchaeota	*Methanosalsum zhilinae DSM 4017*
0	0	Euryarchaeota	*Methanosarcina acetivorans C2A*
0	0	Euryarchaeota	*Methanosarcina barkeri 227*
0	0	Euryarchaeota	*Methanosarcina barkeri 3*
0	0	Euryarchaeota	*Methanosarcina barkeri CM1*
0	0	Euryarchaeota	*Methanosarcina barkeri MS*
0	0	Euryarchaeota	*Methanosarcina barkeri str. Fusaro*
0	0	Euryarchaeota	*Methanosarcina barkeri str. Wiesmoor*
0	0	Euryarchaeota	*Methanosarcina horonobensis HB-1 = JCM 15518*
0	0	Euryarchaeota	*Methanosarcina lacustris Z-7289*
0	0	Euryarchaeota	*Methanosarcina mazei C16*
0	0	Euryarchaeota	*Methanosarcina mazei Go1*
0	0	Euryarchaeota	*Methanosarcina mazei LYC*
0	0	Euryarchaeota	*Methanosarcina mazei S-6*
0	0	Euryarchaeota	*Methanosarcina mazei SarPi*
0	0	Euryarchaeota	*Methanosarcina mazei WWM610*
0	0	Euryarchaeota	*Methanosarcina siciliae C2J*
0	0	Euryarchaeota	*Methanosarcina siciliae T4/M*
0	0	Euryarchaeota	*Methanosarcina sp. Kolksee*
0	0	Euryarchaeota	*Methanosarcina sp. MTP4*
0	0	Euryarchaeota	*Methanosarcina sp. WH1*
0	0	Euryarchaeota	*Methanosarcina sp. WWM596*
0	0	Euryarchaeota	*Methanosarcina thermophila TM-1*
0	0	Euryarchaeota	*Methanosarcina vacuolata Z-761*
0	0	Euryarchaeota	*Methanosphaera stadtmanae DSM 3091*
0	0	Euryarchaeota	*Methanosphaerula palustris E1-9c*
0	0	Euryarchaeota	*Methanospirillum hungatei JF-1*
0	0	Euryarchaeota	*Methanothermobacter marburgensis str. Marburg*
0	0	Euryarchaeota	*Methanothermobacter sp. CaT2*

0	0	Euryarchaeota	*Methanothermobacter thermautotrophicus str. Delta H*
0	0	Euryarchaeota	*Methanothermococcus okinawensis IH1*
0	0	Euryarchaeota	*Methanothermus fervidus DSM 2088*
0	0	Euryarchaeota	*Methanotorris igneus Kol 5*
0	0	Euryarchaeota	*Natrialba magadii ATCC 43099*
0	0	Euryarchaeota	*Natrinema pellirubrum DSM 15624*
0	0	Euryarchaeota	*Natrinema sp. J7-2*
0	0	Euryarchaeota	*Natronobacterium gregoryi SP2*
0	0	Euryarchaeota	*Natronococcus occultus SP4*
0	0	Euryarchaeota	*Natronomonas moolapensis 8.8.11*
0	0	Euryarchaeota	*Natronomonas pharaonis DSM 2160*
0	0	Euryarchaeota	*Palaeococcus pacificus DY20341*
0	0	Euryarchaeota	*Picrophilus torridus DSM 9790*
0	0	Euryarchaeota	*Pyrococcus furiosus COM1*
0	0	Euryarchaeota	*Pyrococcus furiosus DSM 3638*
0	0	Euryarchaeota	*Pyrococcus horikoshii OT3*
0	0	Euryarchaeota	*Pyrococcus sp. NA2*
0	0	Euryarchaeota	*Pyrococcus sp. ST04*
0	0	Euryarchaeota	*Pyrococcus yayanosii CH1*
0	0	Euryarchaeota	*Salinarchaeum sp. Harcht-Bsk1*
0	0	Euryarchaeota	*Thermococcus barophilus*
0	0	Euryarchaeota	*Thermococcus barophilus MP*
0	0	Euryarchaeota	*Thermococcus cleftensis*
0	0	Euryarchaeota	*Thermococcus eurythermalis*
0	0	Euryarchaeota	*Thermococcus gammatolerans EJ3*
0	0	Euryarchaeota	*Thermococcus guaymasensis DSM 11113*
0	0	Euryarchaeota	*Thermococcus kodakarensis KOD1*
0	0	Euryarchaeota	*Thermococcus litoralis DSM 5473*
0	0	Euryarchaeota	*Thermococcus nautili*

0	0	Euryarchaeota	*Thermococcus onnurineus NA1*
0	0	Euryarchaeota	*Thermococcus paralvinellae*
0	0	Euryarchaeota	*Thermococcus peptonophilus*
0	0	Euryarchaeota	*Thermococcus sibiricus MM 739*
0	0	Euryarchaeota	*Thermococcus sp. 2319x1*
0	0	Euryarchaeota	*Thermococcus sp. 4557*
0	0	Euryarchaeota	*Thermococcus sp. AM4*
0	0	Euryarchaeota	*Thermoplasma volcanium GSS1*
0	0	Euryarchaeota	*Thermoplasmatales archaeon BRNA1*
1	1	FCB group	*Aequorivita sublithincola DSM 14238*
1	1	FCB group	*Algibacter sp. HZ22*
1	2	FCB group	*Algoriphagus machipongonensis*
1	1	FCB group	*Alistipes finegoldii DSM 17242*
1	1	FCB group	*Bacteroidales bacterium CF*
1	1	FCB group	*Bacteroides cellulosilyticus*
1	1	FCB group	*Bacteroides fragilis*
1	1	FCB group	*Bacteroides fragilis*
1	1	FCB group	*Bacteroides fragilis 638R*
1	1	FCB group	*Bacteroides fragilis NCTC 9343*
1	1	FCB group	*Bacteroides fragilis YCH46*
1	1	FCB group	*Bacteroides helcogenes P 36-108*
1	1	FCB group	*Bacteroides ovatus*
1	1	FCB group	*Bacteroides salanitronis DSM 18170*
1	1	FCB group	*Bacteroides thetaiotaomicron*
1	1	FCB group	*Bacteroides thetaiotaomicron VPI-5482*
1	1	FCB group	*Bacteroides vulgatus ATCC 8482*
1	1	FCB group	*Barnesiella viscericola DSM 18177*
1	1	FCB group	*Belliella baltica DSM 15883*
1	1	FCB group	*Blattabacterium sp. (Blaberus giganteus)*

1	1	FCB group	*Blattabacterium sp. (Blatta orientalis) str. Tarazona*
1	1	FCB group	*Blattabacterium sp. (Blattella germanica) str. Bge*
1	1	FCB group	*Blattabacterium sp. (Cryptocercus punctulatus) str. Cpu*
1	1	FCB group	*Blattabacterium sp. (Mastotermes darwiniensis) str. MADAR*
1	1	FCB group	*Blattabacterium sp. (Nauphoeta cinerea)*
1	1	FCB group	*Blattabacterium sp. (Panesthia angustipennis spadica) str. BPAA*
1	1	FCB group	*Blattabacterium sp. (Periplaneta americana) str. BPLAN*
1	1	FCB group	*Candidatus Amoebophilus asiaticus 5a2*
1	1	FCB group	*Candidatus Azobacteroides pseudotrichonymphae genomovar. CFP2*
1	1	FCB group	*Candidatus Cloacimonas acidaminovorans str. Evry*
1	1	FCB group	*Capnocytophaga canimorsus Cc5*
1	1	FCB group	*Capnocytophaga ochracea DSM 7271*
1	1	FCB group	*Capnocytophaga sp. oral taxon 323*
1	1	FCB group	*Cardinium endosymbiont cEper1 of Encarsia pergandiella*
1	1	FCB group	*Cellulophaga algicola DSM 14237*
1	1	FCB group	*Cellulophaga baltica 18*
1	1	FCB group	*Cellulophaga baltica NN016038*
1	1	FCB group	*Cellulophaga lytica*
1	1	FCB group	*Cellulophaga lytica DSM 7489*
1	2	FCB group	*Chitinophaga pinensis DSM 2588*
1	2	FCB group	*Chlorobaculum parvum NCIB 8327*
1	2	FCB group	*Chlorobium chlorochromatii CaD3*
1	2	FCB group	*Chlorobium limicola DSM 245*
1	2	FCB group	*Chlorobium luteolum DSM 273*
1	2	FCB group	*Chlorobium phaeobacteroides BS1*
1	2	FCB group	*Chlorobium phaeobacteroides DSM 266*

1	2	FCB group	*Chlorobium phaeovibrioides DSM 265*
1	2	FCB group	*Chlorobium tepidum TLS*
1	2	FCB group	*Chloroherpeton thalassium ATCC 35110*
1	1	FCB group	*Chryseobacterium gallinarum*
1	1	FCB group	*Chryseobacterium sp. IHB B 17019*
1	1	FCB group	*Chryseobacterium sp. StRB126*
1	1	FCB group	*Croceibacter atlanticus HTCC2559*
1	2	FCB group	*Cyclobacterium amurskyense*
1	2	FCB group	*Cyclobacterium marinum DSM 745*
1	1	FCB group	*Cytophaga hutchinsonii ATCC 33406*
1	1	FCB group	*Dokdonia sp. 4H-3-7-5*
1	1	FCB group	*Dokdonia sp. MED134*
1	2	FCB group	*Draconibacterium orientale*
1	2	FCB group	*Dyadobacter fermentans DSM 18053*
1	2	FCB group	*Echinicola vietnamensis DSM 17526*
1	1	FCB group	*Elizabethkingia anophelis*
1	1	FCB group	*Elizabethkingia anophelis NUHP1*
1	1	FCB group	*Elizabethkingia meningoseptica FMS-007*
1	1	FCB group	*Elizabethkingia sp. BM10*
1	2	FCB group	*Emticicia oligotrophica DSM 17448*
1	1	FCB group	*endosymbiont of Llaveia axin axin*
1	1	FCB group	*Fermentimonas caenicola*
1	1	FCB group	*Fibrobacter succinogenes subsp. succinogenes S85*
1	1	FCB group	*Fibrobacter succinogenes subsp. succinogenes S85*
1	2	FCB group	*Flammeovirgaceae bacterium 311*
1	1	FCB group	*Flavobacteriaceae bacterium 3519-10*
1	1	FCB group	*Flavobacterium branchiophilum FL-15*
1	1	FCB group	*Flavobacterium columnare*
1	1	FCB group	*Flavobacterium columnare ATCC 49512*

1	1	FCB group	*Flavobacterium indicum GPTSA100-9 = DSM 17447*
1	1	FCB group	*Flavobacterium johnsoniae UW101*
1	1	FCB group	*Flavobacterium psychrophilum*
1	1	FCB group	*Flavobacterium psychrophilum*
1	1	FCB group	*Flavobacterium psychrophilum*
1	1	FCB group	*Flavobacterium psychrophilum*
1	1	FCB group	*Flavobacterium psychrophilum*
1	1	FCB group	*Flavobacterium psychrophilum*
1	1	FCB group	*Flavobacterium psychrophilum FPG101*
1	1	FCB group	*Flavobacterium psychrophilum FPG3*
1	1	FCB group	*Flavobacterium psychrophilum JIP02/86*
1	1	FCB group	*Flexibacter litoralis DSM 6794*
1	1	FCB group	*Fluviicola taffensis DSM 16823*
1	2	FCB group	*Gemmatimonas aurantiaca T-27*
1	1	FCB group	*Gramella forsetii KT0803*
2	1	FCB group	*Haliscomenobacter hydrossis DSM 1100*
1	1	FCB group	*Hymenobacter sp. APR13*
1	1	FCB group	*Hymenobacter sp. DG25A*
1	1	FCB group	*Hymenobacter sp. DG25B*
1	1	FCB group	*Hymenobacter swuensis DY53*
1	3	FCB group	*Ignavibacterium album JCM 16511*
1	1	FCB group	*Lacinutrix sp. 5H-3-7-4*
1	2	FCB group	*Leadbetterella byssophila DSM 17132*
1	1	FCB group	*Maribacter sp. HTCC2170*
1	1	FCB group	*Marivirga tractuosa DSM 4126*
1	3	FCB group	*Melioribacter roseus P3M-2*
1	1	FCB group	*Mucinivorans hirudinis*
1	1	FCB group	*Muricauda lutaonensis*
1	1	FCB group	*Muricauda ruestringensis DSM 13258*

1	1	FCB group	*Myroides odoratimimus*
1	1	FCB group	*Myroides profundi*
1	1	FCB group	*Myroides sp. A21*
1	2	FCB group	*Niabella soli DSM 19437*
3	3	FCB group	*Niastella koreensis GR20-10*
1	1	FCB group	*Nonlabens dokdonensis DSW-6*
1	1	FCB group	*Nonlabens sp. MIC269*
1	1	FCB group	*Odoribacter splanchnicus DSM 20712*
1	2	FCB group	*Ornithobacterium rhinotracheale DSM 15997*
1	2	FCB group	*Ornithobacterium rhinotracheale ORT-UMN 88*
1	1	FCB group	*Owenweeksia hongkongensis DSM 17368*
1	1	FCB group	*Paludibacter propionicigenes WB4*
1	2	FCB group	*Parabacteroides distasonis ATCC 8503*
1	1	FCB group	*Pedobacter heparinus DSM 2366*
1	1	FCB group	*Pedobacter sp. PACM 27299*
1	2	FCB group	*Pelodictyon phaeoclathratiforme BU-1*
1	1	FCB group	*Persicobacter sp. JZB09*
1	1	FCB group	*Polaribacter sp. MED152*
1	2	FCB group	*Pontibacter korlensis*
1	1	FCB group	*Porphyromonas asaccharolytica DSM 20707*
1	1	FCB group	*Porphyromonas gingivalis*
1	1	FCB group	*Porphyromonas gingivalis 381*
1	1	FCB group	*Porphyromonas gingivalis A7A1-28*
1	1	FCB group	*Porphyromonas gingivalis AJW4*
1	1	FCB group	*Porphyromonas gingivalis ATCC 33277*
1	1	FCB group	*Porphyromonas gingivalis TDC60*
1	1	FCB group	*Porphyromonas gingivalis W83*
1	1	FCB group	*Prevotella dentalis DSM 3688*
1	1	FCB group	*Prevotella denticola F0289*

1	1	FCB group	*Prevotella enoeca*
1	1	FCB group	*Prevotella fusca JCM 17724*
1	1	FCB group	*Prevotella intermedia*
1	1	FCB group	*Prevotella intermedia 17*
1	1	FCB group	*Prevotella melaninogenica ATCC 25845*
1	1	FCB group	*Prevotella ruminicola 23*
1	1	FCB group	*Prevotella sp. oral taxon 299 str. F0039*
1	2	FCB group	*Prosthecochloris aestuarii DSM 271*
1	1	FCB group	*Pseudopedobacter saltans DSM 12145*
1	1	FCB group	*Psychroflexus torquis ATCC 700755*
1	2	FCB group	*Rhodothermus marinus DSM 4252*
1	2	FCB group	*Rhodothermus marinus SG0.5JP17-172*
0	1	FCB group	*Riemerella anatipestifer*
1	1	FCB group	*Riemerella anatipestifer*
1	1	FCB group	*Riemerella anatipestifer ATCC 11845 = DSM 15868*
1	1	FCB group	*Riemerella anatipestifer ATCC 11845 = DSM 15868*
1	1	FCB group	*Riemerella anatipestifer CH3*
1	1	FCB group	*Riemerella anatipestifer RA-CH-1*
1	1	FCB group	*Riemerella anatipestifer RA-CH-2*
1	1	FCB group	*Riemerella anatipestifer RA-GD*
1	1	FCB group	*Riemerella anatipestifer Yb2*
1	1	FCB group	*Robiginitalea biformata HTCC2501*
1	1	FCB group	*Rufibacter sp. DG31D*
1	2	FCB group	*Rufibacter tibetensis*
1	2	FCB group	*Runella slithyformis DSM 19594*
2	2	FCB group	*Salinibacter ruber DSM 13855*
2	2	FCB group	*Salinibacter ruber M8*
1	1	FCB group	*Saprospira grandis str. Lewin*
1	1	FCB group	*Sediminicola sp. YIK13*

1	1	FCB group	*Siansivirga zeaxanthinifaciens CC-SAMT-1*
1	1	FCB group	*Solitalea canadensis DSM 3403*
1	2	FCB group	*Sphingobacterium sp. 21*
1	2	FCB group	*Sphingobacterium sp. ML3W*
1	2	FCB group	*Spirosoma radiotolerans*
1	2	FCB group	*Tannerella forsythia 3313*
1	2	FCB group	*Tannerella forsythia 92A2*
1	2	FCB group	*Tannerella forsythia KS16*
1	1	FCB group	*Tenacibaculum dicentrarchi*
1	1	FCB group	*Weeksella virosa DSM 16922*
1	1	FCB group	*Winogradskyella sp. PG-2*
1	1	FCB group	*Zobellia galactanivorans*
1	1	FCB group	*Zunongwangia profunda SM-A87*
1	1	Fusobacteria	*Fusobacterium hwasookii ChDC F174*
1	1	Fusobacteria	*Fusobacterium hwasookii ChDC F206*
1	1	Fusobacteria	*Fusobacterium hwasookii ChDC F300*
1	1	Fusobacteria	*Fusobacterium nucleatum subsp. animalis*
1	1	Fusobacteria	*Fusobacterium nucleatum subsp. animalis*
1	1	Fusobacteria	*Fusobacterium nucleatum subsp. animalis 4_8*
1	1	Fusobacteria	*Fusobacterium nucleatum subsp. animalis 7_1*
1	1	Fusobacteria	*Fusobacterium nucleatum subsp. nucleatum ATCC 25586*
1	1	Fusobacteria	*Fusobacterium nucleatum subsp. nucleatum ChDC F316*
1	1	Fusobacteria	*Fusobacterium nucleatum subsp. polymorphum*
1	1	Fusobacteria	*Fusobacterium nucleatum subsp. polymorphum*
1	1	Fusobacteria	*Fusobacterium nucleatum subsp. vincentii 3_1_27*
1	1	Fusobacteria	*Fusobacterium nucleatum subsp. vincentii 3_1_36A2*
1	1	Fusobacteria	*Ilyobacter polytropus DSM 2926*
1	1	Fusobacteria	*Leptotrichia buccalis C-1013-b*
1	1	Fusobacteria	*Leptotrichia sp. oral taxon 212*

1	1	Fusobacteria	*Sebaldella termitidis ATCC 33386*
1	1	Fusobacteria	*Sneathia amnii*
1	1	Fusobacteria	*Streptobacillus moniliformis DSM 12112*
1	1	Nitrospirae	*Candidatus Nitrospira inopinata*
1	1	Nitrospirae	*Leptospirillum ferriphilum ML-04*
1	1	Nitrospirae	*Leptospirillum ferriphilum YSK*
1	1	Nitrospirae	*Leptospirillum ferrooxidans C2-3*
1	1	Nitrospirae	*Leptospirillum sp. Group II 'CF-1'*
1	1	Nitrospirae	*Nitrospira defluvii*
1	1	Nitrospirae	*Nitrospira moscoviensis*
1	2	Nitrospirae	*Thermodesulfovibrio yellowstonii DSM 11347*
1	1	Proteobacteria	*[Haemophilus] parasuis*
1	1	Proteobacteria	*[Haemophilus] parasuis*
1	1	Proteobacteria	*[Polyangium] brachysporum*
1	1	Proteobacteria	*[Pseudomonas syringae] pv. tomato str. DC3000*
1	1	Proteobacteria	*Acetobacter pasteurianus*
1	1	Proteobacteria	*Acetobacter pasteurianus 386B*
1	1	Proteobacteria	*Acetobacter pasteurianus IFO 3283-01*
1	1	Proteobacteria	*Acetobacter pasteurianus IFO 3283-01-42C*
1	1	Proteobacteria	*Acetobacter pasteurianus IFO 3283-03*
1	1	Proteobacteria	*Acetobacter pasteurianus IFO 3283-07*
1	1	Proteobacteria	*Acetobacter pasteurianus IFO 3283-12*
1	1	Proteobacteria	*Acetobacter pasteurianus IFO 3283-22*
1	1	Proteobacteria	*Acetobacter pasteurianus IFO 3283-26*
1	1	Proteobacteria	*Acetobacter pasteurianus IFO 3283-32*
1	1	Proteobacteria	*Acetobacter pasteurianus NBRC 101655*
1	1	Proteobacteria	*Acetobacter senegalensis*
2	2	Proteobacteria	*Achromobacter denitrificans*
1	1	Proteobacteria	*Achromobacter xylosoxidans*

2	2	Proteobacteria	*Achromobacter xylosoxidans*
1	1	Proteobacteria	*Achromobacter xylosoxidans A8*
2	2	Proteobacteria	*Achromobacter xylosoxidans C54*
2	1	Proteobacteria	*Acidiphilium cryptum JF-5*
2	1	Proteobacteria	*Acidiphilium multivorum AIU301*
1	1	Proteobacteria	*Acidithiobacillus caldus ATCC 51756*
1	1	Proteobacteria	*Acidithiobacillus caldus SM-1*
1	1	Proteobacteria	*Acidithiobacillus ferrivorans SS3*
1	1	Proteobacteria	*Acidithiobacillus ferrooxidans ATCC 23270*
1	1	Proteobacteria	*Acidithiobacillus ferrooxidans ATCC 53993*
1	1	Proteobacteria	*Acidovorax avenae subsp. avenae ATCC 19860*
1	1	Proteobacteria	*Acidovorax citrulli AAC00-1*
1	1	Proteobacteria	*Acidovorax ebreus TPSY*
1	1	Proteobacteria	*Acidovorax sp. JS42*
1	1	Proteobacteria	*Acidovorax sp. KKS102*
1	1	Proteobacteria	*Acinetobacter baumannii*
1	1	Proteobacteria	*Acinetobacter baumannii*
1	1	Proteobacteria	*Acinetobacter baumannii*
1	1	Proteobacteria	*Acinetobacter baumannii*
1	1	Proteobacteria	*Acinetobacter baumannii*
1	1	Proteobacteria	*Acinetobacter baumannii*
1	1	Proteobacteria	*Acinetobacter baumannii*
1	1	Proteobacteria	*Acinetobacter baumannii*
1	1	Proteobacteria	*Acinetobacter baumannii*
1	1	Proteobacteria	*Acinetobacter baumannii*
1	1	Proteobacteria	*Acinetobacter baumannii*
1	1	Proteobacteria	*Acinetobacter baumannii*
1	1	Proteobacteria	*Acinetobacter baumannii*
1	1	Proteobacteria	*Acinetobacter baumannii*

1	1	Proteobacteria	*Acinetobacter baumannii*
1	1	Proteobacteria	*Acinetobacter baumannii*
1	1	Proteobacteria	*Acinetobacter baumannii*
1	1	Proteobacteria	*Acinetobacter baumannii*
2	2	Proteobacteria	*Acinetobacter baumannii*
2	2	Proteobacteria	*Acinetobacter baumannii*
2	2	Proteobacteria	*Acinetobacter baumannii*
2	2	Proteobacteria	*Acinetobacter baumannii*
1	1	Proteobacteria	*Acinetobacter baumannii 1656-2*
1	1	Proteobacteria	*Acinetobacter baumannii AB0057*
1	1	Proteobacteria	*Acinetobacter baumannii AB307-0294*
1	1	Proteobacteria	*Acinetobacter baumannii ACICU*
1	1	Proteobacteria	*Acinetobacter baumannii AYE*
1	1	Proteobacteria	*Acinetobacter baumannii BJAB07104*
1	1	Proteobacteria	*Acinetobacter baumannii BJAB0715*
1	1	Proteobacteria	*Acinetobacter baumannii BJAB0868*
1	1	Proteobacteria	*Acinetobacter baumannii D1279779*
1	1	Proteobacteria	*Acinetobacter baumannii LAC-4*
1	1	Proteobacteria	*Acinetobacter baumannii MDR-TJ*
1	1	Proteobacteria	*Acinetobacter baumannii MDR-ZJ06*
1	1	Proteobacteria	*Acinetobacter baumannii NCGM 237*
1	1	Proteobacteria	*Acinetobacter baumannii TYTH-1*
2	2	Proteobacteria	*Acinetobacter baumannii ZW85-1*
1	1	Proteobacteria	*Acinetobacter equi*
1	1	Proteobacteria	*Acinetobacter johnsonii XBB1*
2	2	Proteobacteria	*Acinetobacter nosocomialis*
1	1	Proteobacteria	*Acinetobacter oleivorans DR1*
2	2	Proteobacteria	*Acinetobacter pittii*
1	1	Proteobacteria	*Acinetobacter pittii PHEA-2*

1	1	Proteobacteria	*Acinetobacter sp. ADP1*
1	1	Proteobacteria	*Acinetobacter sp. TTH0-4*
1	1	Proteobacteria	*Actinobacillus equuli subsp. equuli*
1	1	Proteobacteria	*Actinobacillus pleuropneumoniae serovar 3 str. JL03*
1	1	Proteobacteria	*Actinobacillus pleuropneumoniae serovar 5b str. L20*
1	1	Proteobacteria	*Actinobacillus pleuropneumoniae serovar 7 str. AP76*
1	1	Proteobacteria	*Actinobacillus pleuropneumoniae serovar 8*
1	1	Proteobacteria	*Actinobacillus succinogenes 130Z*
1	1	Proteobacteria	*Actinobacillus suis ATCC 33415*
1	1	Proteobacteria	*Advenella kashmirensis WT001*
1	1	Proteobacteria	*Advenella mimigardefordensis DPN7*
1	1	Proteobacteria	*Aeromonas hydrophila*
1	1	Proteobacteria	*Aeromonas hydrophila*
1	1	Proteobacteria	*Aeromonas hydrophila*
1	1	Proteobacteria	*Aeromonas hydrophila*
1	1	Proteobacteria	*Aeromonas hydrophila 4AK4*
1	1	Proteobacteria	*Aeromonas hydrophila AL09-71*
1	1	Proteobacteria	*Aeromonas hydrophila J-1*
1	1	Proteobacteria	*Aeromonas hydrophila ML09-119*
1	1	Proteobacteria	*Aeromonas hydrophila pc104A*
1	1	Proteobacteria	*Aeromonas hydrophila subsp. hydrophila ATCC 7966*
1	1	Proteobacteria	*Aeromonas hydrophila YL17*
1	1	Proteobacteria	*Aeromonas media WS*
1	1	Proteobacteria	*Aeromonas salmonicida subsp. salmonicida A449*
1	1	Proteobacteria	*Aeromonas schubertii*
1	1	Proteobacteria	*Aeromonas veronii B565*
1	1	Proteobacteria	*Agarivorans gilvus*
1	1	Proteobacteria	*Aggregatibacter actinomycetemcomitans*
1	1	Proteobacteria	*Aggregatibacter actinomycetemcomitans*

1	1	Proteobacteria	*Aggregatibacter actinomycetemcomitans ANH9381*
1	1	Proteobacteria	*Aggregatibacter actinomycetemcomitans D7S-1*
1	1	Proteobacteria	*Aggregatibacter actinomycetemcomitans HK1651*
1	1	Proteobacteria	*Aggregatibacter actinomycetemcomitans HK1651*
1	1	Proteobacteria	*Aggregatibacter actinomycetemcomitans NUM4039*
1	1	Proteobacteria	*Aggregatibacter aphrophilus*
1	1	Proteobacteria	*Aggregatibacter aphrophilus NJ8700*
1	1	Proteobacteria	*Aggregatibacter aphrophilus NJ8700*
1	1	Proteobacteria	*Agrobacterium fabrum str. C58*
3	3	Proteobacteria	*Agrobacterium radiobacter K84*
2	2	Proteobacteria	*Agrobacterium sp. H13-3*
1	1	Proteobacteria	*Agrobacterium tumefaciens*
1	1	Proteobacteria	*Agrobacterium tumefaciens*
1	1	Proteobacteria	*Agrobacterium vitis S4*
1	1	Proteobacteria	*Alcaligenes faecalis*
1	1	Proteobacteria	*Alcanivorax borkumensis SK2*
1	1	Proteobacteria	*Alcanivorax dieselolei B5*
1	1	Proteobacteria	*Alcanivorax pacificus W11-5*
1	1	Proteobacteria	*Alcanivorax sp. NBRC 101098*
1	1	Proteobacteria	*Alicycliphilus denitrificans BC*
1	1	Proteobacteria	*Alicycliphilus denitrificans K601*
1	1	Proteobacteria	*Aliivibrio salmonicida LFI1238*
1	1	Proteobacteria	*Aliivibrio wodanis*
1	1	Proteobacteria	*Alkalilimnicola ehrlichii MLHE-1*
2	2	Proteobacteria	*Allochromatium vinosum DSM 180*
1	1	Proteobacteria	*alpha proteobacterium HIMB5*
1	1	Proteobacteria	*alpha proteobacterium HIMB59*
2	2	Proteobacteria	*Altererythrobacter atlanticus*
1	1	Proteobacteria	*Altererythrobacter epoxidivorans*

1	1	Proteobacteria	*Altererythrobacter marensis*
1	1	Proteobacteria	*Alteromonas addita*
0	0	Proteobacteria	*Alteromonas australica*
1	1	Proteobacteria	*Alteromonas australica*
1	1	Proteobacteria	*Alteromonas macleodii*
1	1	Proteobacteria	*Alteromonas macleodii*
1	1	Proteobacteria	*Alteromonas macleodii ATCC 27126*
1	1	Proteobacteria	*Alteromonas macleodii str. 'Balearic Sea AD45'*
1	1	Proteobacteria	*Alteromonas macleodii str. 'Black Sea 11'*
1	1	Proteobacteria	*Alteromonas macleodii str. 'English Channel 673'*
1	1	Proteobacteria	*Alteromonas mediterranea*
1	1	Proteobacteria	*Alteromonas mediterranea*
1	1	Proteobacteria	*Alteromonas mediterranea DE1*
1	1	Proteobacteria	*Alteromonas mediterranea MED64*
1	1	Proteobacteria	*Alteromonas mediterranea U7*
1	1	Proteobacteria	*Alteromonas mediterranea U8*
1	1	Proteobacteria	*Alteromonas mediterranea UM4b*
1	1	Proteobacteria	*Alteromonas mediterranea UM7*
1	1	Proteobacteria	*Alteromonas sp. SN2*
1	1	Proteobacteria	*Alteromonas stellipolaris*
1	1	Proteobacteria	*Alteromonas stellipolaris LMG 21856*
2	1	Proteobacteria	*Anaeromyxobacter dehalogenans 2CP-1*
2	1	Proteobacteria	*Anaeromyxobacter dehalogenans 2CP-C*
2	1	Proteobacteria	*Anaeromyxobacter sp. Fw109-5*
2	1	Proteobacteria	*Anaeromyxobacter sp. K*
1	1	Proteobacteria	*Anaplasma centrale str. Israel*
1	1	Proteobacteria	*Anaplasma marginale str. Florida*
1	1	Proteobacteria	*Anaplasma marginale str. St. Maries*
1	1	Proteobacteria	*Anaplasma phagocytophilum str. HZ*

1	1	Proteobacteria	*Anaplasma phagocytophilum str. HZ2*
1	1	Proteobacteria	*Anaplasma phagocytophilum str. JM*
2	1	Proteobacteria	*Archangium gephyra*
1	1	Proteobacteria	*Arcobacter butzleri 7h1h*
1	1	Proteobacteria	*Arcobacter butzleri ED-1*
1	1	Proteobacteria	*Arcobacter butzleri RM4018*
1	1	Proteobacteria	*Arcobacter nitrofigilis DSM 7299*
1	1	Proteobacteria	*Arcobacter sp. L*
1	1	Proteobacteria	*Aromatoleum aromaticum EbN1*
1	1	Proteobacteria	*Asaia bogorensis NBRC 16594*
1	1	Proteobacteria	*Asticcacaulis excentricus CB 48*
1	1	Proteobacteria	*Aureimonas sp. AU20*
3	2	Proteobacteria	*Azoarcus sp. BH72*
1	1	Proteobacteria	*Azoarcus sp. CIB*
2	1	Proteobacteria	*Azoarcus sp. KH32C*
2	2	Proteobacteria	*Azorhizobium caulinodans ORS 571*
1	1	Proteobacteria	*Azospirillum brasilense*
3	3	Proteobacteria	*Azospirillum brasilense*
3	2	Proteobacteria	*Azospirillum lipoferum 4B*
3	2	Proteobacteria	*Azospirillum sp. B510*
2	1	Proteobacteria	*Azospirillum thiophilum*
2	1	Proteobacteria	*Azotobacter chroococcum NCIMB 8003*
2	1	Proteobacteria	*Azotobacter vinelandii CA*
2	1	Proteobacteria	*Azotobacter vinelandii CA6*
2	1	Proteobacteria	*Azotobacter vinelandii DJ*
1	1	Proteobacteria	*Bartonella australis Aust/NH1*
1	1	Proteobacteria	*Bartonella bacilliformis*
1	1	Proteobacteria	*Bartonella bacilliformis KC583*
1	1	Proteobacteria	*Bartonella clarridgeiae 73*

1	1	Proteobacteria	*Bartonella grahamii as4aup*
1	1	Proteobacteria	*Bartonella henselae*
1	1	Proteobacteria	*Bartonella henselae*
1	1	Proteobacteria	*Bartonella henselae*
1	1	Proteobacteria	*Bartonella henselae str. Houston-1*
1	1	Proteobacteria	*Bartonella quintana RM-11*
1	1	Proteobacteria	*Bartonella quintana str. Toulouse*
1	1	Proteobacteria	*Bartonella tribocorum*
1	1	Proteobacteria	*Bartonella tribocorum CIP 105476*
1	1	Proteobacteria	*Bartonella vinsonii subsp. berkhoffii str. Winnie*
1	1	Proteobacteria	*Basilea psittacipulmonis DSM 24701*
1	1	Proteobacteria	*Baumannia cicadellinicola str. Hc (Homalodisca coagulata)*
1	2	Proteobacteria	*Bdellovibrio bacteriovorus*
1	1	Proteobacteria	*Bdellovibrio bacteriovorus str. Tiberius*
1	2	Proteobacteria	*Bdellovibrio bacteriovorus W*
1	2	Proteobacteria	*Bdellovibrio exovorus JSS*
2	2	Proteobacteria	*Beggiatoa leptomitiformis*
2	2	Proteobacteria	*Beijerinckia indica subsp. indica ATCC 9039*
1	1	Proteobacteria	*beta proteobacterium CB*
1	1	Proteobacteria	*Bibersteinia trehalosi USDA-ARS-USMARC-188*
1	1	Proteobacteria	*Bibersteinia trehalosi USDA-ARS-USMARC-189*
1	1	Proteobacteria	*Bibersteinia trehalosi USDA-ARS-USMARC-190*
1	1	Proteobacteria	*Bibersteinia trehalosi USDA-ARS-USMARC-192*
2	2	Proteobacteria	*Blastochloris viridis*
2	2	Proteobacteria	*Blastochloris viridis*
2	2	Proteobacteria	*Blastochloris viridis*
1	1	Proteobacteria	*Blochmannia endosymbiont of Camponotus (Colobopsis) obliquus*
1	1	Proteobacteria	*Blochmannia endosymbiont of Polyrhachis (Hedomyrma)*

			turneri
1	1	Proteobacteria	*Bordetella avium 197N*
1	1	Proteobacteria	*Bordetella bronchiseptica*
1	1	Proteobacteria	*Bordetella bronchiseptica 253*
1	1	Proteobacteria	*Bordetella bronchiseptica MO149*
2	2	Proteobacteria	*Bordetella hinzii*
2	2	Proteobacteria	*Bordetella hinzii*
1	0	Proteobacteria	*Bordetella holmesii 44057*
1	1	Proteobacteria	*Bordetella holmesii ATCC 51541*
1	1	Proteobacteria	*Bordetella holmesii F627*
1	1	Proteobacteria	*Bordetella holmesii H558*
1	1	Proteobacteria	*Bordetella parapertussis Bpp5*
1	1	Proteobacteria	*Bordetella pertussis*
1	1	Proteobacteria	*Bordetella pertussis*
1	1	Proteobacteria	*Bordetella pertussis*
1	1	Proteobacteria	*Bordetella pertussis*
1	1	Proteobacteria	*Bordetella pertussis*
1	1	Proteobacteria	*Bordetella pertussis*
1	1	Proteobacteria	*Bordetella pertussis*
1	1	Proteobacteria	*Bordetella pertussis*
1	1	Proteobacteria	*Bordetella pertussis*
1	1	Proteobacteria	*Bordetella pertussis*
1	1	Proteobacteria	*Bordetella pertussis*
1	1	Proteobacteria	*Bordetella pertussis*
1	1	Proteobacteria	*Bordetella pertussis*
1	1	Proteobacteria	*Bordetella pertussis*
1	1	Proteobacteria	*Bordetella pertussis*
1	1	Proteobacteria	*Bordetella pertussis*
1	1	Proteobacteria	*Bordetella pertussis*

1	1	Proteobacteria	*Bordetella pertussis*
1	1	Proteobacteria	*Bordetella pertussis*
1	1	Proteobacteria	*Bordetella pertussis*
1	1	Proteobacteria	*Bordetella pertussis*
1	1	Proteobacteria	*Bordetella pertussis*
1	1	Proteobacteria	*Bordetella pertussis*
1	1	Proteobacteria	*Bordetella pertussis*
1	1	Proteobacteria	*Bordetella pertussis*
1	1	Proteobacteria	*Bordetella pertussis*
1	1	Proteobacteria	*Bordetella pertussis*
1	1	Proteobacteria	*Bordetella pertussis*
1	1	Proteobacteria	*Bordetella pertussis*
1	1	Proteobacteria	*Bordetella pertussis*
1	1	Proteobacteria	*Bordetella pertussis*
1	1	Proteobacteria	*Bordetella pertussis*
1	1	Proteobacteria	*Bordetella pertussis*
1	1	Proteobacteria	*Bordetella pertussis*
1	1	Proteobacteria	*Bordetella pertussis*
1	1	Proteobacteria	*Bordetella pertussis*
1	1	Proteobacteria	*Bordetella pertussis*
1	1	Proteobacteria	*Bordetella pertussis*
1	1	Proteobacteria	*Bordetella pertussis*
1	1	Proteobacteria	*Bordetella pertussis*
1	1	Proteobacteria	*Bordetella pertussis*
1	1	Proteobacteria	*Bordetella pertussis*
1	1	Proteobacteria	*Bordetella pertussis*
1	1	Proteobacteria	*Bordetella pertussis*
1	1	Proteobacteria	*Bordetella pertussis*
1	1	Proteobacteria	*Bordetella pertussis*

1	1	Proteobacteria	*Bordetella pertussis 137*
1	1	Proteobacteria	*Bordetella pertussis 18323*
1	1	Proteobacteria	*Bordetella pertussis B1917*
1	1	Proteobacteria	*Bordetella pertussis CS*
1	1	Proteobacteria	*Bordetella petrii*
4	4	Proteobacteria	*Bradyrhizobium diazoefficiens*
7	5	Proteobacteria	*Bradyrhizobium diazoefficiens USDA 110*
6	5	Proteobacteria	*Bradyrhizobium japonicum*
6	5	Proteobacteria	*Bradyrhizobium japonicum USDA 6*
3	2	Proteobacteria	*Bradyrhizobium oligotrophicum S58*
5	4	Proteobacteria	*Bradyrhizobium sp. BTAi1*
4	4	Proteobacteria	*Bradyrhizobium sp. CCGE-LA001*
4	3	Proteobacteria	*Bradyrhizobium sp. ORS 278*
3	3	Proteobacteria	*Bradyrhizobium sp. S23321*
1	1	Proteobacteria	*Brevundimonas sp. DS20*
1	1	Proteobacteria	*Brevundimonas subvibrioides ATCC 15264*
1	1	Proteobacteria	*Brucella abortus*
1	1	Proteobacteria	*Brucella abortus*
1	1	Proteobacteria	*Brucella abortus*
1	1	Proteobacteria	*Brucella abortus*
1	1	Proteobacteria	*Brucella abortus*
1	1	Proteobacteria	*Brucella abortus*
1	1	Proteobacteria	*Brucella abortus 104M*
1	1	Proteobacteria	*Brucella abortus 2308*
1	1	Proteobacteria	*Brucella abortus A13334*
1	1	Proteobacteria	*Brucella abortus bv. 1 str. 9-941*
1	1	Proteobacteria	*Brucella abortus bv. 2 str. 86/8/59*
1	1	Proteobacteria	*Brucella abortus bv. 6 str. 870*
1	1	Proteobacteria	*Brucella abortus bv. 9 str. C68*

1	1	Proteobacteria	*Brucella abortus S19*
1	1	Proteobacteria	*Brucella canis*
1	1	Proteobacteria	*Brucella canis*
1	1	Proteobacteria	*Brucella canis ATCC 23365*
1	1	Proteobacteria	*Brucella canis HSK A52141*
0	1	Proteobacteria	*Brucella ceti TE10759-12*
1	1	Proteobacteria	*Brucella melitensis*
1	1	Proteobacteria	*Brucella melitensis ATCC 23457*
1	1	Proteobacteria	*Brucella melitensis bv. 1 str. 16M*
1	1	Proteobacteria	*Brucella melitensis bv. 1 str. 16M*
1	1	Proteobacteria	*Brucella melitensis bv. 3 str. Ether*
1	1	Proteobacteria	*Brucella melitensis M28*
1	1	Proteobacteria	*Brucella melitensis M5-90*
1	1	Proteobacteria	*Brucella melitensis NI*
1	1	Proteobacteria	*Brucella microti CCM 4915*
1	1	Proteobacteria	*Brucella ovis ATCC 25840*
1	1	Proteobacteria	*Brucella pinnipedialis*
1	1	Proteobacteria	*Brucella pinnipedialis B2/94*
1	1	Proteobacteria	*Brucella suis*
1	1	Proteobacteria	*Brucella suis*
1	1	Proteobacteria	*Brucella suis*
2	2	Proteobacteria	*Brucella suis*
2	2	Proteobacteria	*Brucella suis*
1	1	Proteobacteria	*Brucella suis 1330*
1	1	Proteobacteria	*Brucella suis 1330*
1	1	Proteobacteria	*Brucella suis ATCC 23445*
1	1	Proteobacteria	*Brucella suis bv. 1 str. S2*
1	1	Proteobacteria	*Brucella suis bv. 2*
1	1	Proteobacteria	*Brucella suis bv. 2*

1	1	Proteobacteria	*Brucella suis bv. 2*
1	1	Proteobacteria	*Brucella suis bv. 2*
1	1	Proteobacteria	*Brucella suis bv. 2*
1	1	Proteobacteria	*Brucella suis bv. 3 str. 686*
1	1	Proteobacteria	*Brucella suis VBI22*
1	1	Proteobacteria	*Brucella vulpis*
1	1	Proteobacteria	*Buchnera aphidicola (Aphis glycines)*
1	1	Proteobacteria	*Buchnera aphidicola (Cinara tujafilina)*
1	1	Proteobacteria	*Buchnera aphidicola BCc*
1	1	Proteobacteria	*Buchnera aphidicola str. 5A (Acyrthosiphon pisum)*
1	1	Proteobacteria	*Buchnera aphidicola str. Ak (Acyrthosiphon kondoi)*
1	1	Proteobacteria	*Buchnera aphidicola str. APS (Acyrthosiphon pisum)*
1	1	Proteobacteria	*Buchnera aphidicola str. Bp (Baizongia pistaciae)*
1	1	Proteobacteria	*Buchnera aphidicola str. F009 (Myzus persicae)*
1	1	Proteobacteria	*Buchnera aphidicola str. G002 (Myzus persicae)*
1	1	Proteobacteria	*Buchnera aphidicola str. JF98 (Acyrthosiphon pisum)*
1	1	Proteobacteria	*Buchnera aphidicola str. JF99 (Acyrthosiphon pisum)*
1	1	Proteobacteria	*Buchnera aphidicola str. LL01 (Acyrthosiphon pisum)*
1	1	Proteobacteria	*Buchnera aphidicola str. LSR1 (Acyrthosiphon pisum)*
1	1	Proteobacteria	*Buchnera aphidicola str. Sg (Schizaphis graminum)*
1	1	Proteobacteria	*Buchnera aphidicola str. TLW03 (Acyrthosiphon pisum)*
1	1	Proteobacteria	*Buchnera aphidicola str. Tuc7 (Acyrthosiphon pisum)*
1	1	Proteobacteria	*Buchnera aphidicola str. Ua (Uroleucon ambrosiae)*
1	1	Proteobacteria	*Buchnera aphidicola str. USDA (Myzus persicae)*
1	1	Proteobacteria	*Buchnera aphidicola str. W106 (Myzus persicae)*
3	3	Proteobacteria	*Burkholderia ambifaria AMMD*
3	3	Proteobacteria	*Burkholderia ambifaria AMMD*
3	3	Proteobacteria	*Burkholderia ambifaria MC40-6*
3	3	Proteobacteria	*Burkholderia cenocepacia*

3	3	Proteobacteria	*Burkholderia cenocepacia*
3	3	Proteobacteria	*Burkholderia cenocepacia*
2	2	Proteobacteria	*Burkholderia cenocepacia AU 1054*
3	3	Proteobacteria	*Burkholderia cenocepacia H111*
3	3	Proteobacteria	*Burkholderia cenocepacia HI2424*
3	3	Proteobacteria	*Burkholderia cenocepacia J2315*
3	3	Proteobacteria	*Burkholderia cenocepacia MC0-3*
2	2	Proteobacteria	*Burkholderia cepacia*
4	3	Proteobacteria	*Burkholderia cepacia*
2	2	Proteobacteria	*Burkholderia cepacia ATCC 25416*
2	2	Proteobacteria	*Burkholderia cepacia GG4*
2	2	Proteobacteria	*Burkholderia cepacia JBK9*
2	2	Proteobacteria	*Burkholderia contaminans*
2	2	Proteobacteria	*Burkholderia dolosa AU0158*
3	3	Proteobacteria	*Burkholderia gladioli*
3	3	Proteobacteria	*Burkholderia gladioli BSR3*
2	2	Proteobacteria	*Burkholderia glumae BGR1*
2	2	Proteobacteria	*Burkholderia glumae LMG 2196 = ATCC 33617*
3	3	Proteobacteria	*Burkholderia glumae PG1*
2	2	Proteobacteria	*Burkholderia lata*
1	2	Proteobacteria	*Burkholderia mallei*
1	2	Proteobacteria	*Burkholderia mallei*
1	2	Proteobacteria	*Burkholderia mallei*
1	2	Proteobacteria	*Burkholderia mallei*
1	2	Proteobacteria	*Burkholderia mallei*
1	2	Proteobacteria	*Burkholderia mallei*
1	2	Proteobacteria	*Burkholderia mallei*
1	2	Proteobacteria	*Burkholderia mallei*
1	2	Proteobacteria	*Burkholderia mallei*

1	2	Proteobacteria	*Burkholderia mallei*
1	2	Proteobacteria	*Burkholderia mallei ATCC 23344*
1	2	Proteobacteria	*Burkholderia mallei NCTC 10229*
1	2	Proteobacteria	*Burkholderia mallei NCTC 10247*
1	2	Proteobacteria	*Burkholderia mallei NCTC 10247*
1	2	Proteobacteria	*Burkholderia mallei SAVP1*
2	2	Proteobacteria	*Burkholderia multivorans*
2	2	Proteobacteria	*Burkholderia multivorans ATCC 17616*
2	2	Proteobacteria	*Burkholderia multivorans ATCC 17616*
2	2	Proteobacteria	*Burkholderia multivorans ATCC BAA-247*
2	2	Proteobacteria	*Burkholderia oklahomensis*
2	2	Proteobacteria	*Burkholderia oklahomensis C6786*
3	3	Proteobacteria	*Burkholderia plantarii*
2	3	Proteobacteria	*Burkholderia pseudomallei*
2	3	Proteobacteria	*Burkholderia pseudomallei*
2	3	Proteobacteria	*Burkholderia pseudomallei*
2	3	Proteobacteria	*Burkholderia pseudomallei*
2	3	Proteobacteria	*Burkholderia pseudomallei*
2	3	Proteobacteria	*Burkholderia pseudomallei*
2	3	Proteobacteria	*Burkholderia pseudomallei*
2	3	Proteobacteria	*Burkholderia pseudomallei*
2	3	Proteobacteria	*Burkholderia pseudomallei*
2	3	Proteobacteria	*Burkholderia pseudomallei*
2	3	Proteobacteria	*Burkholderia pseudomallei*
2	3	Proteobacteria	*Burkholderia pseudomallei*
2	3	Proteobacteria	*Burkholderia pseudomallei*
2	3	Proteobacteria	*Burkholderia pseudomallei*
3	3	Proteobacteria	*Burkholderia pseudomallei*
2	3	Proteobacteria	*Burkholderia pseudomallei 1026b*

2	3	Proteobacteria	*Burkholderia pseudomallei 1026b*
2	3	Proteobacteria	*Burkholderia pseudomallei 1106a*
2	3	Proteobacteria	*Burkholderia pseudomallei 1710b*
2	3	Proteobacteria	*Burkholderia pseudomallei 406e*
2	3	Proteobacteria	*Burkholderia pseudomallei 576*
2	3	Proteobacteria	*Burkholderia pseudomallei 668*
2	3	Proteobacteria	*Burkholderia pseudomallei 7894*
2	3	Proteobacteria	*Burkholderia pseudomallei A79A*
2	3	Proteobacteria	*Burkholderia pseudomallei B03*
2	3	Proteobacteria	*Burkholderia pseudomallei BPC006*
2	3	Proteobacteria	*Burkholderia pseudomallei HBPUB10134a*
2	3	Proteobacteria	*Burkholderia pseudomallei HBPUB10303a*
2	3	Proteobacteria	*Burkholderia pseudomallei K42*
2	3	Proteobacteria	*Burkholderia pseudomallei K96243*
2	3	Proteobacteria	*Burkholderia pseudomallei K96243*
2	3	Proteobacteria	*Burkholderia pseudomallei MSHR146*
3	3	Proteobacteria	*Burkholderia pseudomallei MSHR305*
3	3	Proteobacteria	*Burkholderia pseudomallei MSHR520*
2	3	Proteobacteria	*Burkholderia pseudomallei MSHR5848*
2	3	Proteobacteria	*Burkholderia pseudomallei MSHR5855*
2	3	Proteobacteria	*Burkholderia pseudomallei MSHR5858*
3	3	Proteobacteria	*Burkholderia pseudomallei MSHR62*
2	3	Proteobacteria	*Burkholderia pseudomallei NAU20B-16*
3	3	Proteobacteria	*Burkholderia pseudomallei NAU35A-3*
2	3	Proteobacteria	*Burkholderia pseudomallei NCTC 13178*
2	3	Proteobacteria	*Burkholderia pseudomallei NCTC 13179*
2	3	Proteobacteria	*Burkholderia pseudomallei Pasteur 52237*
2	3	Proteobacteria	*Burkholderia pseudomallei PB08298010*
2	3	Proteobacteria	*Burkholderia pseudomallei TSV 48*

2	2	Proteobacteria	*Burkholderia pyrrocinia*
2	2	Proteobacteria	*Burkholderia sp. 2002721687*
2	2	Proteobacteria	*Burkholderia sp. Bp5365*
1	1	Proteobacteria	*Burkholderia sp. CCGE1001*
3	3	Proteobacteria	*Burkholderia sp. CCGE1002*
3	2	Proteobacteria	*Burkholderia sp. CCGE1003*
3	3	Proteobacteria	*Burkholderia sp. HB1*
3	3	Proteobacteria	*Burkholderia sp. KJ006*
1	1	Proteobacteria	*Burkholderia sp. PAMC 26561*
1	1	Proteobacteria	*Burkholderia sp. PAMC 28687*
1	1	Proteobacteria	*Burkholderia sp. RPE64*
2	2	Proteobacteria	*Burkholderia sp. RPE67*
2	2	Proteobacteria	*Burkholderia sp. YI23*
3	2	Proteobacteria	*Burkholderia thailandensis*
3	2	Proteobacteria	*Burkholderia thailandensis 2002721643*
3	2	Proteobacteria	*Burkholderia thailandensis 2002721723*
3	2	Proteobacteria	*Burkholderia thailandensis 34*
3	2	Proteobacteria	*Burkholderia thailandensis E254*
3	2	Proteobacteria	*Burkholderia thailandensis E264*
3	2	Proteobacteria	*Burkholderia thailandensis E444*
3	2	Proteobacteria	*Burkholderia thailandensis H0587*
2	2	Proteobacteria	*Burkholderia thailandensis MSMB121*
3	2	Proteobacteria	*Burkholderia thailandensis MSMB59*
3	2	Proteobacteria	*Burkholderia thailandensis USAMRU Malaysia #20*
4	4	Proteobacteria	*Burkholderia ubonensis MSMB22*
2	2	Proteobacteria	*Burkholderia vietnamiensis G4*
2	2	Proteobacteria	*Burkholderia vietnamiensis LMG 10929*
1	1	Proteobacteria	*Burkholderiales bacterium GJ-E10*
1	1	Proteobacteria	*Campylobacter coli*

1	1	Proteobacteria	*Campylobacter coli*
1	1	Proteobacteria	*Campylobacter coli*
1	1	Proteobacteria	*Campylobacter coli*
1	1	Proteobacteria	*Campylobacter coli 15-537360*
1	1	Proteobacteria	*Campylobacter coli CVM N29710*
1	1	Proteobacteria	*Campylobacter coli RM1875*
1	1	Proteobacteria	*Campylobacter coli RM4661*
1	1	Proteobacteria	*Campylobacter coli RM5611*
1	1	Proteobacteria	*Campylobacter concisus*
1	1	Proteobacteria	*Campylobacter concisus 13826*
1	1	Proteobacteria	*Campylobacter curvus 525.92*
1	1	Proteobacteria	*Campylobacter fetus subsp. fetus 04/554*
1	1	Proteobacteria	*Campylobacter fetus subsp. fetus 82-40*
1	1	Proteobacteria	*Campylobacter fetus subsp. testudinum*
1	1	Proteobacteria	*Campylobacter fetus subsp. testudinum 03-427*
1	1	Proteobacteria	*Campylobacter fetus subsp. testudinum Sp3*
1	1	Proteobacteria	*Campylobacter fetus subsp. venerealis 97/608*
1	1	Proteobacteria	*Campylobacter fetus subsp. venerealis cfvi03/293*
1	1	Proteobacteria	*Campylobacter fetus subsp. venerealis str. 84-112*
1	1	Proteobacteria	*Campylobacter gracilis*
1	1	Proteobacteria	*Campylobacter hominis ATCC BAA-381*
1	1	Proteobacteria	*Campylobacter iguaniorum*
1	1	Proteobacteria	*Campylobacter iguaniorum*
1	1	Proteobacteria	*Campylobacter insulaenigrae NCTC 12927*
1	1	Proteobacteria	*Campylobacter jejuni*
1	1	Proteobacteria	*Campylobacter jejuni*
1	1	Proteobacteria	*Campylobacter jejuni*
1	1	Proteobacteria	*Campylobacter jejuni*
1	1	Proteobacteria	*Campylobacter jejuni*

1	1	Proteobacteria	*Campylobacter jejuni*
1	1	Proteobacteria	*Campylobacter jejuni*
1	1	Proteobacteria	*Campylobacter jejuni*
1	1	Proteobacteria	*Campylobacter jejuni*
1	1	Proteobacteria	*Campylobacter jejuni*
1	1	Proteobacteria	*Campylobacter jejuni*
1	1	Proteobacteria	*Campylobacter jejuni*
1	1	Proteobacteria	*Campylobacter jejuni*
1	1	Proteobacteria	*Campylobacter jejuni*
1	1	Proteobacteria	*Campylobacter jejuni*
1	1	Proteobacteria	*Campylobacter jejuni*
1	1	Proteobacteria	*Campylobacter jejuni*
1	1	Proteobacteria	*Campylobacter jejuni*
1	1	Proteobacteria	*Campylobacter jejuni*
1	1	Proteobacteria	*Campylobacter jejuni*
1	1	Proteobacteria	*Campylobacter jejuni*
1	1	Proteobacteria	*Campylobacter jejuni*
1	1	Proteobacteria	*Campylobacter jejuni*
1	1	Proteobacteria	*Campylobacter jejuni*
1	1	Proteobacteria	*Campylobacter jejuni*
1	1	Proteobacteria	*Campylobacter jejuni*
1	1	Proteobacteria	*Campylobacter jejuni*
1	1	Proteobacteria	*Campylobacter jejuni*
1	1	Proteobacteria	*Campylobacter jejuni*
1	1	Proteobacteria	*Campylobacter jejuni*
1	1	Proteobacteria	*Campylobacter jejuni*
1	1	Proteobacteria	*Campylobacter jejuni*
1	1	Proteobacteria	*Campylobacter jejuni*
1	1	Proteobacteria	*Campylobacter jejuni*

1	1	Proteobacteria	*Campylobacter jejuni*
1	1	Proteobacteria	*Campylobacter jejuni*
1	1	Proteobacteria	*Campylobacter jejuni*
1	1	Proteobacteria	*Campylobacter jejuni*
1	1	Proteobacteria	*Campylobacter jejuni*
1	1	Proteobacteria	*Campylobacter jejuni*
1	1	Proteobacteria	*Campylobacter jejuni*
1	1	Proteobacteria	*Campylobacter jejuni*
1	1	Proteobacteria	*Campylobacter jejuni*
1	1	Proteobacteria	*Campylobacter jejuni*
1	1	Proteobacteria	*Campylobacter jejuni*
1	1	Proteobacteria	*Campylobacter jejuni*
1	1	Proteobacteria	*Campylobacter jejuni*
1	1	Proteobacteria	*Campylobacter jejuni*
1	1	Proteobacteria	*Campylobacter jejuni*
1	1	Proteobacteria	*Campylobacter jejuni*
1	1	Proteobacteria	*Campylobacter jejuni*
1	1	Proteobacteria	*Campylobacter jejuni*
1	1	Proteobacteria	*Campylobacter jejuni*
1	1	Proteobacteria	*Campylobacter jejuni*
1	1	Proteobacteria	*Campylobacter jejuni*
1	1	Proteobacteria	*Campylobacter jejuni*
1	1	Proteobacteria	*Campylobacter jejuni*
1	1	Proteobacteria	*Campylobacter jejuni*
1	1	Proteobacteria	*Campylobacter jejuni*
1	1	Proteobacteria	*Campylobacter jejuni*
1	1	Proteobacteria	*Campylobacter jejuni 32488*
1	1	Proteobacteria	*Campylobacter jejuni 4031*
1	1	Proteobacteria	*Campylobacter jejuni RM1221*

1	1	Proteobacteria	*Campylobacter jejuni subsp. doylei 269.97*
1	1	Proteobacteria	*Campylobacter jejuni subsp. jejuni*
1	1	Proteobacteria	*Campylobacter jejuni subsp. jejuni*
1	1	Proteobacteria	*Campylobacter jejuni subsp. jejuni*
1	1	Proteobacteria	*Campylobacter jejuni subsp. jejuni*
1	1	Proteobacteria	*Campylobacter jejuni subsp. jejuni*
1	1	Proteobacteria	*Campylobacter jejuni subsp. jejuni*
1	1	Proteobacteria	*Campylobacter jejuni subsp. jejuni*
1	1	Proteobacteria	*Campylobacter jejuni subsp. jejuni*
1	1	Proteobacteria	*Campylobacter jejuni subsp. jejuni*
1	1	Proteobacteria	*Campylobacter jejuni subsp. jejuni 00-2425*
1	1	Proteobacteria	*Campylobacter jejuni subsp. jejuni 00-2426*
1	1	Proteobacteria	*Campylobacter jejuni subsp. jejuni 00-2538*
1	1	Proteobacteria	*Campylobacter jejuni subsp. jejuni 00-2544*
1	1	Proteobacteria	*Campylobacter jejuni subsp. jejuni 81116*
1	1	Proteobacteria	*Campylobacter jejuni subsp. jejuni 81-176*
1	1	Proteobacteria	*Campylobacter jejuni subsp. jejuni 81-176*
1	1	Proteobacteria	*Campylobacter jejuni subsp. jejuni CG8421*
1	1	Proteobacteria	*Campylobacter jejuni subsp. jejuni F38011*
1	1	Proteobacteria	*Campylobacter jejuni subsp. jejuni IA3902*
1	1	Proteobacteria	*Campylobacter jejuni subsp. jejuni M1*
1	1	Proteobacteria	*Campylobacter jejuni subsp. jejuni NCTC 11168 = ATCC 700819*
1	1	Proteobacteria	*Campylobacter jejuni subsp. jejuni NCTC 11168-BN148*
1	1	Proteobacteria	*Campylobacter jejuni subsp. jejuni NCTC 11168-GSv*
1	1	Proteobacteria	*Campylobacter jejuni subsp. jejuni NCTC 11168-K12E5*
1	1	Proteobacteria	*Campylobacter jejuni subsp. jejuni NCTC 11168-Kf1*
1	1	Proteobacteria	*Campylobacter jejuni subsp. jejuni NCTC 11168-mcK12E5*
1	1	Proteobacteria	*Campylobacter jejuni subsp. jejuni NCTC 11168-mfK12E5*

1	1	Proteobacteria	*Campylobacter jejuni subsp. jejuni PT14*
1	1	Proteobacteria	*Campylobacter jejuni subsp. jejuni R14*
1	1	Proteobacteria	*Campylobacter jejuni subsp. jejuni S3*
1	1	Proteobacteria	*Campylobacter lari*
1	1	Proteobacteria	*Campylobacter lari CCUG 22395*
1	1	Proteobacteria	*Campylobacter lari NCTC 11845*
1	1	Proteobacteria	*Campylobacter lari RM16701*
1	1	Proteobacteria	*Campylobacter lari RM16712*
1	1	Proteobacteria	*Campylobacter lari RM2100*
1	1	Proteobacteria	*Campylobacter lari subsp. concheus LMG 11760*
1	1	Proteobacteria	*Campylobacter peloridis LMG 23910*
1	1	Proteobacteria	*Campylobacter sp. RM16704*
1	1	Proteobacteria	*Campylobacter subantarcticus LMG 24374*
1	1	Proteobacteria	*Campylobacter subantarcticus LMG 24377*
1	1	Proteobacteria	*Campylobacter ureolyticus RIGS 9880*
1	1	Proteobacteria	*Campylobacter volucris LMG 24379*
1	1	Proteobacteria	*Candidatus Accumulibacter phosphatis clade IIA str. UW-1*
1	1	Proteobacteria	*Candidatus Babela massiliensis*
1	1	Proteobacteria	*Candidatus Bartonella ancashi*
1	1	Proteobacteria	*Candidatus Baumannia cicadellinicola*
1	1	Proteobacteria	*Candidatus Baumannia cicadellinicola*
1	1	Proteobacteria	*Candidatus Blochmannia chromaiodes str. 640*
1	1	Proteobacteria	*Candidatus Blochmannia floridanus*
1	1	Proteobacteria	*Candidatus Blochmannia pennsylvanicus str. BPEN*
1	1	Proteobacteria	*Candidatus Blochmannia vafer str. BVAF*
1	1	Proteobacteria	*Candidatus Carsonella ruddii HT isolate Thao2000*
1	1	Proteobacteria	*Candidatus Endolissoclinum faulkneri L2*
1	1	Proteobacteria	*Candidatus Endolissoclinum faulkneri L5*
1	1	Proteobacteria	*Candidatus Evansia muelleri*

2	2	Proteobacteria	*Candidatus Filomicrobium marinum*
2	2	Proteobacteria	*Candidatus Filomicrobium marinum*
1	1	Proteobacteria	*Candidatus Ishikawaella capsulata Mpkobe*
1	1	Proteobacteria	*Candidatus Kinetoplastibacterium blastocrithidii (ex Strigomonas culicis)*
1	1	Proteobacteria	*Candidatus Kinetoplastibacterium blastocrithidii TCC012E*
1	1	Proteobacteria	*Candidatus Kinetoplastibacterium crithidii (ex Angomonas deanei ATCC 30255)*
1	1	Proteobacteria	*Candidatus Kinetoplastibacterium crithidii TCC036E*
1	1	Proteobacteria	*Candidatus Kinetoplastibacterium desouzaii TCC079E*
1	1	Proteobacteria	*Candidatus Kinetoplastibacterium galatii TCC219*
1	1	Proteobacteria	*Candidatus Kinetoplastibacterium oncopeltii TCC290E*
1	1	Proteobacteria	*Candidatus Liberibacter africanus PTSAPSY*
1	1	Proteobacteria	*Candidatus Liberibacter americanus str. Sao Paulo*
1	1	Proteobacteria	*Candidatus Liberibacter asiaticus*
1	1	Proteobacteria	*Candidatus Liberibacter asiaticus str. gxpsy*
1	1	Proteobacteria	*Candidatus Liberibacter asiaticus str. Ishi-1*
1	1	Proteobacteria	*Candidatus Liberibacter asiaticus str. psy62*
1	1	Proteobacteria	*Candidatus Liberibacter solanacearum CLso-ZC1*
1	1	Proteobacteria	*Candidatus Methylopumilus planktonicus*
1	1	Proteobacteria	*Candidatus Methylopumilus turicensis*
0	1	Proteobacteria	*Candidatus Midichloria mitochondrii IricVA*
1	1	Proteobacteria	*Candidatus Moranella endobia PCIT*
1	1	Proteobacteria	*Candidatus Moranella endobia PCVAL*
1	1	Proteobacteria	*Candidatus Pantoea carbekii*
1	1	Proteobacteria	*Candidatus Pantoea carbekii*
1	1	Proteobacteria	*Candidatus Paracaedibacter acanthamoebae*
1	1	Proteobacteria	*Candidatus Pelagibacter sp. IMCC9063*
1	1	Proteobacteria	*Candidatus Pelagibacter ubique HTCC1062*

1	1	Proteobacteria	*Candidatus Portiera aleyrodidarum BT-B-HRs*
1	1	Proteobacteria	*Candidatus Portiera aleyrodidarum BT-B-HRs*
1	1	Proteobacteria	*Candidatus Portiera aleyrodidarum BT-QVLC*
1	1	Proteobacteria	*Candidatus Portiera aleyrodidarum BT-QVLC*
1	1	Proteobacteria	*Candidatus Portiera aleyrodidarum MED (Bemisia tabaci)*
1	1	Proteobacteria	*Candidatus Profftella armatura*
1	1	Proteobacteria	*Candidatus Profftella armatura*
1	1	Proteobacteria	*Candidatus Puniceispirillum marinum IMCC1322*
1	1	Proteobacteria	*Candidatus Rickettsia amblyommii*
1	1	Proteobacteria	*Candidatus Rickettsia amblyommii str. GAT-30V*
1	1	Proteobacteria	*Candidatus Riesia pediculicola USDA*
1	1	Proteobacteria	*Candidatus Ruthia magnifica str. Cm (Calyptogena magnifica)*
2	2	Proteobacteria	*Candidatus Sodalis pierantonius str. SOPE*
1	1	Proteobacteria	*Candidatus Symbiobacter mobilis CR*
1	1	Proteobacteria	*Candidatus Tachikawaea gelatinosa*
1	1	Proteobacteria	*Candidatus Thioglobus autotrophica*
1	1	Proteobacteria	*Candidatus Thioglobus singularis PS1*
1	1	Proteobacteria	*Candidatus Vesicomyosocius okutanii HA*
2	2	Proteobacteria	*Castellaniella defragrans 65Phen*
1	1	Proteobacteria	*Caulobacter crescentus CB15*
1	1	Proteobacteria	*Caulobacter crescentus NA1000*
1	1	Proteobacteria	*Caulobacter henricii*
1	1	Proteobacteria	*Caulobacter segnis ATCC 21756*
1	1	Proteobacteria	*Caulobacter sp. K31*
1	1	Proteobacteria	*Cedecea neteri*
1	1	Proteobacteria	*Cedecea neteri*
1	1	Proteobacteria	*Cedecea neteri*
1	1	Proteobacteria	*Cedecea neteri*

1	1	Proteobacteria	*Cedecea neteri*
1	1	Proteobacteria	*Celeribacter indicus*
1	1	Proteobacteria	*Celeribacter marinus*
1	1	Proteobacteria	*Cellvibrio japonicus Ueda107*
1	1	Proteobacteria	*Chania multitudinisentens RB-25*
2	2	Proteobacteria	*Chelativorans sp. BNC1*
2	2	Proteobacteria	*Chelatococcus sp. CO-6*
2	2	Proteobacteria	*Chondromyces crocatus*
2	2	Proteobacteria	*Chromobacterium violaceum ATCC 12472*
1	1	Proteobacteria	*Chromohalobacter salexigens DSM 3043*
1	1	Proteobacteria	*Citrobacter amalonaticus*
1	1	Proteobacteria	*Citrobacter amalonaticus*
1	1	Proteobacteria	*Citrobacter amalonaticus Y19*
1	1	Proteobacteria	*Citrobacter freundii*
1	1	Proteobacteria	*Citrobacter freundii*
1	1	Proteobacteria	*Citrobacter freundii*
1	1	Proteobacteria	*Citrobacter freundii CFNIH1*
1	1	Proteobacteria	*Citrobacter koseri ATCC BAA-895*
1	1	Proteobacteria	*Citrobacter rodentium ICC168*
1	1	Proteobacteria	*Citrobacter sp. FDAARGOS_156*
1	1	Proteobacteria	*Citromicrobium sp. JL477*
1	1	Proteobacteria	*Collimonas arenae*
1	1	Proteobacteria	*Collimonas arenae*
1	1	Proteobacteria	*Collimonas arenae*
1	1	Proteobacteria	*Collimonas fungivorans*
1	1	Proteobacteria	*Collimonas fungivorans Ter331*
1	1	Proteobacteria	*Collimonas pratensis*
1	1	Proteobacteria	*Collimonas pratensis*
1	1	Proteobacteria	*Colwellia psychrerythraea 34H*

1	1	Proteobacteria	*Colwellia sp. MT41*
1	1	Proteobacteria	*Comamonadaceae bacterium A1*
1	1	Proteobacteria	*Comamonadaceae bacterium B1*
1	1	Proteobacteria	*Comamonas testosteroni CNB-2*
1	1	Proteobacteria	*Comamonas testosteroni P19*
1	1	Proteobacteria	*Comamonas testosteroni TK102*
1	1	Proteobacteria	*Confluentimicrobium sp. EMB200-NS6*
2	1	Proteobacteria	*Corallococcus coralloides DSM 2259*
1	1	Proteobacteria	*Coxiella burnetii*
1	1	Proteobacteria	*Coxiella burnetii CbuG_Q212*
1	1	Proteobacteria	*Coxiella burnetii CbuK_Q154*
1	1	Proteobacteria	*Coxiella burnetii Dugway 5J108-111*
1	1	Proteobacteria	*Coxiella burnetii RSA 331*
1	1	Proteobacteria	*Coxiella burnetii RSA 493*
1	1	Proteobacteria	*Coxiella burnetii Z3055*
1	1	Proteobacteria	*Coxiella endosymbiont of Amblyomma americanum*
1	1	Proteobacteria	*Coxiella-like endosymbiont*
2	2	Proteobacteria	*Croceicoccus naphthovorans*
1	1	Proteobacteria	*Cronobacter condimenti 1330*
1	1	Proteobacteria	*Cronobacter dublinensis subsp. dublinensis LMG 23823*
1	1	Proteobacteria	*Cronobacter malonaticus LMG 23826*
1	1	Proteobacteria	*Cronobacter muytjensii ATCC 51329*
1	1	Proteobacteria	*Cronobacter sakazakii*
1	1	Proteobacteria	*Cronobacter sakazakii*
1	1	Proteobacteria	*Cronobacter sakazakii ATCC BAA-894*
1	1	Proteobacteria	*Cronobacter sakazakii CMCC 45402*
1	1	Proteobacteria	*Cronobacter sakazakii ES15*
1	1	Proteobacteria	*Cronobacter sakazakii SP291*
1	1	Proteobacteria	*Cronobacter turicensis z3032*

1	1	Proteobacteria	*Cronobacter universalis NCTC 9529*
1	1	Proteobacteria	*Cupriavidus basilensis*
1	1	Proteobacteria	*Cupriavidus gilardii CR3*
1	1	Proteobacteria	*Cupriavidus metallidurans CH34*
1	1	Proteobacteria	*Cupriavidus necator N-1*
1	1	Proteobacteria	*Cycloclasticus sp. P1*
1	1	Proteobacteria	*Cycloclasticus zancles 78-ME*
1	1	Proteobacteria	*Dechloromonas aromatica RCB*
1	1	Proteobacteria	*Dechlorosoma suillum PS*
1	1	Proteobacteria	*Delftia acidovorans SPH-1*
1	1	Proteobacteria	*Delftia sp. Cs1-4*
1	1	Proteobacteria	*Desulfarculus baarsii DSM 2075*
1	1	Proteobacteria	*Desulfatibacillum alkenivorans AK-01*
1	1	Proteobacteria	*Desulfobacca acetoxidans DSM 11109*
1	1	Proteobacteria	*Desulfobacterium autotrophicum HRM2*
1	1	Proteobacteria	*Desulfobacula toluolica Tol2*
1	1	Proteobacteria	*Desulfobulbus propionicus DSM 2032*
1	1	Proteobacteria	*Desulfocapsa sulfexigens DSM 10523*
1	1	Proteobacteria	*Desulfococcus oleovorans Hxd3*
1	1	Proteobacteria	*Desulfohalobium retbaense DSM 5692*
1	1	Proteobacteria	*Desulfomicrobium baculatum DSM 4028*
1	1	Proteobacteria	*Desulfomonile tiedjei DSM 6799*
1	1	Proteobacteria	*Desulfotalea psychrophila LSv54*
2	2	Proteobacteria	*Desulfovibrio aespoeensis Aspo-2*
1	1	Proteobacteria	*Desulfovibrio africanus str. Walvis Bay*
1	1	Proteobacteria	*Desulfovibrio alaskensis G20*
2	2	Proteobacteria	*Desulfovibrio desulfuricans ND132*
1	1	Proteobacteria	*Desulfovibrio desulfuricans subsp. desulfuricans str. ATCC 27774*

1	1	Proteobacteria	*Desulfovibrio fairfieldensis*
1	1	Proteobacteria	*Desulfovibrio magneticus RS-1*
2	2	Proteobacteria	*Desulfovibrio salexigens DSM 2638*
1	1	Proteobacteria	*Desulfovibrio vulgaris DP4*
1	1	Proteobacteria	*Desulfovibrio vulgaris RCH1*
1	1	Proteobacteria	*Desulfovibrio vulgaris str. Hildenborough*
1	1	Proteobacteria	*Desulfovibrio vulgaris str. 'Miyazaki F'*
1	1	Proteobacteria	*Desulfurella acetivorans A63*
1	1	Proteobacteria	*Desulfurivibrio alkaliphilus AHT 2*
1	1	Proteobacteria	*Desulfuromonas sp. WTL*
3	2	Proteobacteria	*Devosia sp. A16*
1	1	Proteobacteria	*Devosia sp. H5989*
1	1	Proteobacteria	*Dichelobacter nodosus VCS1703A*
1	1	Proteobacteria	*Dickeya dadantii 3937*
1	1	Proteobacteria	*Dickeya zeae EC1*
1	1	Proteobacteria	*Dickeya zeae Ech586*
1	1	Proteobacteria	*Dinoroseobacter shibae DFL 12 = DSM 16493*
1	1	Proteobacteria	*Dyella japonica A8*
1	1	Proteobacteria	*Dyella jiangningensis*
1	1	Proteobacteria	*Edwardsiella anguillarum ET080813*
1	1	Proteobacteria	*Edwardsiella ictaluri 93-146*
1	1	Proteobacteria	*Edwardsiella piscicida C07-087*
1	1	Proteobacteria	*Edwardsiella sp. EA181011*
1	1	Proteobacteria	*Edwardsiella tarda*
1	1	Proteobacteria	*Edwardsiella tarda EIB202*
1	1	Proteobacteria	*Edwardsiella tarda FL6-60*
1	1	Proteobacteria	*Ehrlichia canis str. Jake*
1	1	Proteobacteria	*Ehrlichia chaffeensis str. Arkansas*
1	1	Proteobacteria	*Ehrlichia chaffeensis str. Heartland*

1	1	Proteobacteria	*Ehrlichia chaffeensis str. Jax*
1	1	Proteobacteria	*Ehrlichia chaffeensis str. Liberty*
1	1	Proteobacteria	*Ehrlichia chaffeensis str. Osceola*
1	1	Proteobacteria	*Ehrlichia chaffeensis str. Saint Vincent*
1	1	Proteobacteria	*Ehrlichia chaffeensis str. Wakulla*
1	1	Proteobacteria	*Ehrlichia chaffeensis str. West Paces*
1	1	Proteobacteria	*Ehrlichia muris AS145*
1	1	Proteobacteria	*Ehrlichia ruminantium str. Gardel*
1	1	Proteobacteria	*Ehrlichia ruminantium str. Welgevonden*
1	1	Proteobacteria	*Ehrlichia ruminantium str. Welgevonden*
1	1	Proteobacteria	*Ehrlichia sp. HF*
1	1	Proteobacteria	*endosymbiont of Acanthamoeba sp. UWC8*
2	2	Proteobacteria	*endosymbiont of unidentified scaly snail isolate Monju*
1	1	Proteobacteria	*Enterobacter aerogenes*
1	1	Proteobacteria	*Enterobacter aerogenes*
1	1	Proteobacteria	*Enterobacter aerogenes*
1	1	Proteobacteria	*Enterobacter aerogenes*
1	1	Proteobacteria	*Enterobacter aerogenes EA1509E*
1	1	Proteobacteria	*Enterobacter aerogenes KCTC 2190*
1	1	Proteobacteria	*Enterobacter asburiae*
1	1	Proteobacteria	*Enterobacter asburiae*
1	1	Proteobacteria	*Enterobacter asburiae*
1	1	Proteobacteria	*Enterobacter asburiae L1*
1	1	Proteobacteria	*Enterobacter asburiae LF7a*
1	1	Proteobacteria	*Enterobacter cloacae*
1	1	Proteobacteria	*Enterobacter cloacae*
1	1	Proteobacteria	*Enterobacter cloacae*
1	1	Proteobacteria	*Enterobacter cloacae*
1	1	Proteobacteria	*Enterobacter cloacae*

1	1	Proteobacteria	*Enterobacter cloacae*
1	1	Proteobacteria	*Enterobacter cloacae*
1	1	Proteobacteria	*Enterobacter cloacae*
1	1	Proteobacteria	*Enterobacter cloacae*
1	1	Proteobacteria	*Enterobacter cloacae*
1	1	Proteobacteria	*Enterobacter cloacae*
1	1	Proteobacteria	*Enterobacter cloacae*
2	2	Proteobacteria	*Enterobacter cloacae*
1	1	Proteobacteria	*Enterobacter cloacae ECNIH2*
1	1	Proteobacteria	*Enterobacter cloacae ECNIH3*
1	1	Proteobacteria	*Enterobacter cloacae ECR091*
1	1	Proteobacteria	*Enterobacter cloacae EcWSU1*
1	1	Proteobacteria	*Enterobacter cloacae P101*
1	1	Proteobacteria	*Enterobacter cloacae subsp. cloacae ATCC 13047*
1	1	Proteobacteria	*Enterobacter cloacae subsp. cloacae ENHKU01*
1	1	Proteobacteria	*Enterobacter cloacae subsp. dissolvens SDM*
1	1	Proteobacteria	*Enterobacter lignolyticus SCF1*
1	1	Proteobacteria	*Enterobacter sp. 638*
1	1	Proteobacteria	*Enterobacter sp. E20*
1	1	Proteobacteria	*Enterobacter sp. FY-07*
1	1	Proteobacteria	*Enterobacter sp. R4-368*
1	1	Proteobacteria	*Enterobacteriaceae bacterium strain FGI 57*
1	1	Proteobacteria	*Erwinia amylovora ATCC 49946*
1	1	Proteobacteria	*Erwinia amylovora CFBP1430*
1	1	Proteobacteria	*Erwinia amylovora LA635*
1	1	Proteobacteria	*Erwinia amylovora LA636*
1	1	Proteobacteria	*Erwinia amylovora LA637*
1	1	Proteobacteria	*Erwinia billingiae Eb661*
1	1	Proteobacteria	*Erwinia pyrifoliae Ep1/96*

1	1	Proteobacteria	*Erwinia sp. Ejp617*
1	1	Proteobacteria	*Erwinia tasmaniensis Et1/99*
2	2	Proteobacteria	*Erythrobacter litoralis HTCC2594*
1	1	Proteobacteria	*Erythrobacter sp. s21-N3*
1	1	Proteobacteria	*Escherichia albertii*
1	1	Proteobacteria	*Escherichia albertii KF1*
1	1	Proteobacteria	*Escherichia coli*
1	1	Proteobacteria	*Escherichia coli*
1	1	Proteobacteria	*Escherichia coli*
1	1	Proteobacteria	*Escherichia coli*
1	1	Proteobacteria	*Escherichia coli*
1	1	Proteobacteria	*Escherichia coli*
1	1	Proteobacteria	*Escherichia coli*
1	1	Proteobacteria	*Escherichia coli*
1	1	Proteobacteria	*Escherichia coli*
1	1	Proteobacteria	*Escherichia coli*
1	1	Proteobacteria	*Escherichia coli*
1	1	Proteobacteria	*Escherichia coli*
1	1	Proteobacteria	*Escherichia coli*
1	1	Proteobacteria	*Escherichia coli*
1	1	Proteobacteria	*Escherichia coli*
1	1	Proteobacteria	*Escherichia coli*
1	1	Proteobacteria	*Escherichia coli*
1	1	Proteobacteria	*Escherichia coli*
1	1	Proteobacteria	*Escherichia coli*
1	1	Proteobacteria	*Escherichia coli*
1	1	Proteobacteria	*Escherichia coli*
1	1	Proteobacteria	*Escherichia coli*
1	1	Proteobacteria	*Escherichia coli*

1	1	Proteobacteria	*Escherichia coli*
1	1	Proteobacteria	*Escherichia coli*
1	1	Proteobacteria	*Escherichia coli*
1	1	Proteobacteria	*Escherichia coli*
1	1	Proteobacteria	*Escherichia coli*
1	1	Proteobacteria	*Escherichia coli*
1	1	Proteobacteria	*Escherichia coli*
1	1	Proteobacteria	*Escherichia coli*
1	1	Proteobacteria	*Escherichia coli*
1	1	Proteobacteria	*Escherichia coli*
1	1	Proteobacteria	*Escherichia coli*
1	1	Proteobacteria	*Escherichia coli*
1	1	Proteobacteria	*Escherichia coli*
1	1	Proteobacteria	*Escherichia coli*
1	1	Proteobacteria	*Escherichia coli*
1	1	Proteobacteria	*Escherichia coli*
1	1	Proteobacteria	*Escherichia coli*
1	1	Proteobacteria	*Escherichia coli*
1	1	Proteobacteria	*Escherichia coli*
1	1	Proteobacteria	*Escherichia coli*
1	1	Proteobacteria	*Escherichia coli*
1	1	Proteobacteria	*Escherichia coli*
1	1	Proteobacteria	*Escherichia coli*
1	1	Proteobacteria	*Escherichia coli*
1	1	Proteobacteria	*Escherichia coli*
1	1	Proteobacteria	*Escherichia coli*
3	4	Proteobacteria	*Escherichia coli*
1	1	Proteobacteria	*Escherichia coli 042*
1	1	Proteobacteria	*Escherichia coli 1303*

1	1	Proteobacteria	*Escherichia coli 536*
1	1	Proteobacteria	*Escherichia coli 55989*
1	1	Proteobacteria	*Escherichia coli ABU 83972*
1	1	Proteobacteria	*Escherichia coli ACN001*
1	1	Proteobacteria	*Escherichia coli APEC IMT5155*
2	2	Proteobacteria	*Escherichia coli APEC O1*
1	1	Proteobacteria	*Escherichia coli APEC O78*
1	1	Proteobacteria	*Escherichia coli ATCC 25922*
1	1	Proteobacteria	*Escherichia coli ATCC 8739*
1	1	Proteobacteria	*Escherichia coli B*
1	1	Proteobacteria	*Escherichia coli B*
1	1	Proteobacteria	*Escherichia coli B str. REL606*
1	1	Proteobacteria	*Escherichia coli BL21(DE3)*
1	1	Proteobacteria	*Escherichia coli BL21(DE3)*
1	1	Proteobacteria	*Escherichia coli 'BL21-Gold(DE3)pLysS AG'*
1	1	Proteobacteria	*Escherichia coli BW25113*
1	1	Proteobacteria	*Escherichia coli BW2952*
1	1	Proteobacteria	*Escherichia coli CFT073*
1	1	Proteobacteria	*Escherichia coli DH1*
1	1	Proteobacteria	*Escherichia coli DH1*
1	1	Proteobacteria	*Escherichia coli ECC-1470*
1	1	Proteobacteria	*Escherichia coli ER2796*
1	1	Proteobacteria	*Escherichia coli ETEC H10407*
1	1	Proteobacteria	*Escherichia coli HS*
1	1	Proteobacteria	*Escherichia coli IAI1*
1	1	Proteobacteria	*Escherichia coli IAI39*
1	1	Proteobacteria	*Escherichia coli IHE3034*
1	1	Proteobacteria	*Escherichia coli JJ1886*
1	1	Proteobacteria	*Escherichia coli JJ1887*

1	1	Proteobacteria	*Escherichia coli K-12*
1	1	Proteobacteria	*Escherichia coli K-12*
1	1	Proteobacteria	*Escherichia coli K-12*
1	1	Proteobacteria	*Escherichia coli K-12*
1	1	Proteobacteria	*Escherichia coli K-12*
1	1	Proteobacteria	*Escherichia coli K-12*
1	1	Proteobacteria	*Escherichia coli K-12*
1	1	Proteobacteria	*Escherichia coli K-12*
1	1	Proteobacteria	*Escherichia coli K-12*
1	1	Proteobacteria	*Escherichia coli K-12*
1	1	Proteobacteria	*Escherichia coli K-12*
1	1	Proteobacteria	*Escherichia coli K-12*
1	1	Proteobacteria	*Escherichia coli K-12*
1	1	Proteobacteria	*Escherichia coli K-12*
1	1	Proteobacteria	*Escherichia coli KLY*
1	1	Proteobacteria	*Escherichia coli KO11FL*
1	1	Proteobacteria	*Escherichia coli KO11FL*
1	1	Proteobacteria	*Escherichia coli LF82*
1	1	Proteobacteria	*Escherichia coli LY180*
1	1	Proteobacteria	*Escherichia coli NA114*
1	1	Proteobacteria	*Escherichia coli Nissle 1917*
1	1	Proteobacteria	*Escherichia coli O103:H2 str. 12009*
1	1	Proteobacteria	*Escherichia coli O104:H4 str. 2009EL-2050*
1	1	Proteobacteria	*Escherichia coli O104:H4 str. 2009EL-2071*
1	1	Proteobacteria	*Escherichia coli O104:H4 str. 2011C-3493*
1	1	Proteobacteria	*Escherichia coli O104:H4 str. C227-11*
1	1	Proteobacteria	*Escherichia coli O111:H- str. 11128*
1	1	Proteobacteria	*Escherichia coli O127:H6 str. E2348/69*
1	1	Proteobacteria	*Escherichia coli O139:H28 str. E24377A*

1	1	Proteobacteria	*Escherichia coli O145:H28 str. RM12581*
1	1	Proteobacteria	*Escherichia coli O145:H28 str. RM12761*
1	1	Proteobacteria	*Escherichia coli O145:H28 str. RM13514*
1	1	Proteobacteria	*Escherichia coli O145:H28 str. RM13516*
1	1	Proteobacteria	*Escherichia coli O157:H16*
1	1	Proteobacteria	*Escherichia coli O157:H7*
1	1	Proteobacteria	*Escherichia coli O157:H7 str. EC4115*
1	1	Proteobacteria	*Escherichia coli O157:H7 str. EDL933*
1	1	Proteobacteria	*Escherichia coli O157:H7 str. Sakai*
1	1	Proteobacteria	*Escherichia coli O157:H7 str. SS17*
1	1	Proteobacteria	*Escherichia coli O157:H7 str. SS52*
1	1	Proteobacteria	*Escherichia coli O157:H7 str. TW14359*
1	1	Proteobacteria	*Escherichia coli O25b:H4-ST131*
1	1	Proteobacteria	*Escherichia coli O26:H11 str. 11368*
1	1	Proteobacteria	*Escherichia coli O55:H7 str. CB9615*
1	1	Proteobacteria	*Escherichia coli O55:H7 str. RM12579*
1	1	Proteobacteria	*Escherichia coli O7:K1 str. CE10*
1	1	Proteobacteria	*Escherichia coli O83:H1 str. NRG 857C*
1	1	Proteobacteria	*Escherichia coli P12b*
1	1	Proteobacteria	*Escherichia coli PCN033*
1	1	Proteobacteria	*Escherichia coli PCN061*
1	1	Proteobacteria	*Escherichia coli PMV-1*
1	1	Proteobacteria	*Escherichia coli RS218*
1	1	Proteobacteria	*Escherichia coli SE11*
1	1	Proteobacteria	*Escherichia coli SE15*
1	1	Proteobacteria	*Escherichia coli SMS-3-5*
1	1	Proteobacteria	*Escherichia coli str. 'clone D i14'*
1	1	Proteobacteria	*Escherichia coli str. 'clone D i2'*
1	1	Proteobacteria	*Escherichia coli str. K-12 substr. DH10B*

1	1	Proteobacteria	*Escherichia coli str. K-12 substr. MC4100*
1	1	Proteobacteria	*Escherichia coli str. K-12 substr. MDS42*
1	1	Proteobacteria	*Escherichia coli str. K-12 substr. MG1655*
1	1	Proteobacteria	*Escherichia coli str. K-12 substr. MG1655*
1	1	Proteobacteria	*Escherichia coli str. K-12 substr. MG1655*
1	1	Proteobacteria	*Escherichia coli str. K-12 substr. MG1655*
1	1	Proteobacteria	*Escherichia coli str. K-12 substr. MG1655*
1	1	Proteobacteria	*Escherichia coli str. K-12 substr. MG1655*
1	1	Proteobacteria	*Escherichia coli str. K-12 substr. MG1655*
1	1	Proteobacteria	*Escherichia coli str. K-12 substr. W3110*
1	1	Proteobacteria	*Escherichia coli UM146*
1	1	Proteobacteria	*Escherichia coli UMNF18*
1	1	Proteobacteria	*Escherichia coli UMNK88*
1	1	Proteobacteria	*Escherichia coli UTI89*
1	1	Proteobacteria	*Escherichia coli VR50*
1	1	Proteobacteria	*Escherichia coli W*
1	1	Proteobacteria	*Escherichia coli W*
1	1	Proteobacteria	*Escherichia coli Xuzhou21*
1	1	Proteobacteria	*Escherichia fergusonii ATCC 35469*
1	1	Proteobacteria	*Ferrimonas balearica DSM 9799*
1	1	Proteobacteria	*Francisella cf. novicida Fx1*
1	1	Proteobacteria	*Francisella cf. tularensis subsp. novicida 3523*
1	1	Proteobacteria	*Francisella guangzhouensis*
1	1	Proteobacteria	*Francisella noatunensis subsp. orientalis*
1	1	Proteobacteria	*Francisella noatunensis subsp. orientalis*
1	1	Proteobacteria	*Francisella noatunensis subsp. orientalis FNO12*
1	1	Proteobacteria	*Francisella noatunensis subsp. orientalis FNO24*
1	1	Proteobacteria	*Francisella noatunensis subsp. orientalis LADL--07-285A*
1	1	Proteobacteria	*Francisella noatunensis subsp. orientalis str. Toba 04*

1	1	Proteobacteria	*Francisella philomiragia*
1	1	Proteobacteria	*Francisella philomiragia*
1	1	Proteobacteria	*Francisella philomiragia*
1	1	Proteobacteria	*Francisella philomiragia*
1	1	Proteobacteria	*Francisella philomiragia*
1	1	Proteobacteria	*Francisella philomiragia subsp. philomiragia ATCC 25015*
1	1	Proteobacteria	*Francisella philomiragia subsp. philomiragia ATCC 25017*
1	1	Proteobacteria	*Francisella sp. FSC1006*
1	1	Proteobacteria	*Francisella sp. TX077308*
1	1	Proteobacteria	*Francisella tularensis subsp. holarctica*
1	1	Proteobacteria	*Francisella tularensis subsp. holarctica*
1	1	Proteobacteria	*Francisella tularensis subsp. holarctica*
1	1	Proteobacteria	*Francisella tularensis subsp. holarctica F92*
1	1	Proteobacteria	*Francisella tularensis subsp. holarctica FSC200*
1	1	Proteobacteria	*Francisella tularensis subsp. holarctica FTNF002-00*
1	1	Proteobacteria	*Francisella tularensis subsp. holarctica LVS*
1	1	Proteobacteria	*Francisella tularensis subsp. holarctica LVS*
1	1	Proteobacteria	*Francisella tularensis subsp. holarctica OSU18*
1	1	Proteobacteria	*Francisella tularensis subsp. holarctica OSU18*
0	1	Proteobacteria	*Francisella tularensis subsp. holarctica PHIT-FT049*
1	1	Proteobacteria	*Francisella tularensis subsp. mediasiatica FSC147*
1	1	Proteobacteria	*Francisella tularensis subsp. novicida D9876*
1	1	Proteobacteria	*Francisella tularensis subsp. novicida F6168*
1	1	Proteobacteria	*Francisella tularensis subsp. novicida U112*
1	1	Proteobacteria	*Francisella tularensis subsp. novicida U112*
1	1	Proteobacteria	*Francisella tularensis subsp. tularensis*
1	1	Proteobacteria	*Francisella tularensis subsp. tularensis*
1	1	Proteobacteria	*Francisella tularensis subsp. tularensis*
1	1	Proteobacteria	*Francisella tularensis subsp. tularensis FSC198*

1	1	Proteobacteria	*Francisella tularensis subsp. tularensis MA00-2987*
1	1	Proteobacteria	*Francisella tularensis subsp. tularensis NE061598*
1	1	Proteobacteria	*Francisella tularensis subsp. tularensis SCHU S4*
1	1	Proteobacteria	*Francisella tularensis subsp. tularensis SCHU S4*
1	1	Proteobacteria	*Francisella tularensis subsp. tularensis str. SCHU S4 substr. NR-28534*
1	1	Proteobacteria	*Francisella tularensis subsp. tularensis TI0902*
1	1	Proteobacteria	*Francisella tularensis subsp. tularensis TIGB03*
1	1	Proteobacteria	*Francisella tularensis subsp. tularensis WY-00W4114*
1	1	Proteobacteria	*Francisella tularensis subsp. tularensis WY96-3418*
1	1	Proteobacteria	*Frateuria aurantia DSM 6220*
1	1	Proteobacteria	*Frischella perrara*
1	1	Proteobacteria	*Gallibacterium anatis UMN179*
1	1	Proteobacteria	*Gallionella capsiferriformans ES-2*
1	1	Proteobacteria	*gamma proteobacterium HdN1*
1	1	Proteobacteria	*Geoalkalibacter subterraneus*
1	1	Proteobacteria	*Geobacter bemidjiensis Bem*
1	1	Proteobacteria	*Geobacter daltonii FRC-32*
1	1	Proteobacteria	*Geobacter lovleyi SZ*
1	1	Proteobacteria	*Geobacter metallireducens GS-15*
1	1	Proteobacteria	*Geobacter pickeringii*
1	1	Proteobacteria	*Geobacter sp. M18*
1	1	Proteobacteria	*Geobacter sp. M21*
1	1	Proteobacteria	*Geobacter sulfurreducens KN400*
1	1	Proteobacteria	*Geobacter sulfurreducens PCA*
1	1	Proteobacteria	*Geobacter uraniireducens Rf4*
1	1	Proteobacteria	*Gilliamella apicola*
1	1	Proteobacteria	*Glaciecola nitratireducens FR1064*
1	1	Proteobacteria	*Glaciecola sp. 4H-3-7+YE-5*

2	2	Proteobacteria	*Gluconacetobacter diazotrophicus PA1 5*
2	4	Proteobacteria	*Gluconacetobacter diazotrophicus PA1 5*
1	1	Proteobacteria	*Gluconobacter oxydans 621H*
1	1	Proteobacteria	*Gluconobacter oxydans DSM 3504*
1	1	Proteobacteria	*Gluconobacter oxydans H24*
1	1	Proteobacteria	*Granulibacter bethesdensis CGDNIH1*
1	1	Proteobacteria	*Granulibacter bethesdensis CGDNIH2*
1	1	Proteobacteria	*Granulibacter bethesdensis CGDNIH3*
1	1	Proteobacteria	*Granulibacter bethesdensis CGDNIH4*
2	1	Proteobacteria	*Grimontia hollisae*
1	1	Proteobacteria	*Gynuella sunshinyii YC6258*
1	1	Proteobacteria	*Haemophilus ducreyi 35000HP*
1	1	Proteobacteria	*Haemophilus influenzae*
1	1	Proteobacteria	*Haemophilus influenzae*
1	1	Proteobacteria	*Haemophilus influenzae*
1	1	Proteobacteria	*Haemophilus influenzae*
1	1	Proteobacteria	*Haemophilus influenzae*
1	1	Proteobacteria	*Haemophilus influenzae 10810*
1	1	Proteobacteria	*Haemophilus influenzae 2019*
1	1	Proteobacteria	*Haemophilus influenzae 86-028NP*
1	1	Proteobacteria	*Haemophilus influenzae CGSHiCZ412602*
1	1	Proteobacteria	*Haemophilus influenzae F3031*
1	1	Proteobacteria	*Haemophilus influenzae F3047*
1	1	Proteobacteria	*Haemophilus influenzae KR494*
1	1	Proteobacteria	*Haemophilus influenzae PittEE*
1	1	Proteobacteria	*Haemophilus influenzae R2846*
1	1	Proteobacteria	*Haemophilus influenzae R2866*
1	1	Proteobacteria	*Haemophilus influenzae Rd KW20*
1	1	Proteobacteria	*Haemophilus parainfluenzae T3T1*

1	1	Proteobacteria	*Haemophilus parasuis SH0165*
1	1	Proteobacteria	*Haemophilus somnus 129PT*
1	1	Proteobacteria	*Haemophilus somnus 2336*
1	1	Proteobacteria	*Hafnia alvei*
1	1	Proteobacteria	*Hafnia alvei FB1*
1	1	Proteobacteria	*Hahella chejuensis KCTC 2396*
2	3	Proteobacteria	*Haliangium ochraceum DSM 14365*
1	1	Proteobacteria	*Halobacteriovorax marinus SJ*
2	2	Proteobacteria	*Halocynthiibacter arcticus*
1	1	Proteobacteria	*Halomonas campaniensis*
1	1	Proteobacteria	*Halomonas elongata DSM 2581*
1	1	Proteobacteria	*Halomonas huangheensis*
1	1	Proteobacteria	*Halomonas sp. KO116*
1	1	Proteobacteria	*Halorhodospira halophila SL1*
1	1	Proteobacteria	*Halothiobacillus neapolitanus c2*
1	1	Proteobacteria	*Helicobacter acinonychis str. Sheeba*
1	1	Proteobacteria	*Helicobacter cetorum MIT 00-7128*
1	1	Proteobacteria	*Helicobacter cetorum MIT 99-5656*
1	1	Proteobacteria	*Helicobacter cinaedi CCUG 18818 = ATCC BAA-847*
1	1	Proteobacteria	*Helicobacter cinaedi PAGU611*
1	1	Proteobacteria	*Helicobacter felis ATCC 49179*
1	1	Proteobacteria	*Helicobacter hepaticus ATCC 51449*
1	1	Proteobacteria	*Helicobacter mustelae 12198*
1	1	Proteobacteria	*Helicobacter pylori*
1	1	Proteobacteria	*Helicobacter pylori*
1	1	Proteobacteria	*Helicobacter pylori*
1	1	Proteobacteria	*Helicobacter pylori*
1	1	Proteobacteria	*Helicobacter pylori*
1	1	Proteobacteria	*Helicobacter pylori*

1	1	Proteobacteria	*Helicobacter pylori*
1	1	Proteobacteria	*Helicobacter pylori*
1	1	Proteobacteria	*Helicobacter pylori*
1	1	Proteobacteria	*Helicobacter pylori 2017*
1	1	Proteobacteria	*Helicobacter pylori 2018*
1	1	Proteobacteria	*Helicobacter pylori 26695*
1	1	Proteobacteria	*Helicobacter pylori 26695*
1	1	Proteobacteria	*Helicobacter pylori 26695-1*
1	1	Proteobacteria	*Helicobacter pylori 26695-1*
1	1	Proteobacteria	*Helicobacter pylori 26695-1CH*
1	1	Proteobacteria	*Helicobacter pylori 26695-1CL*
1	1	Proteobacteria	*Helicobacter pylori 35A*
1	1	Proteobacteria	*Helicobacter pylori 51*
1	1	Proteobacteria	*Helicobacter pylori 52*
1	1	Proteobacteria	*Helicobacter pylori 83*
1	1	Proteobacteria	*Helicobacter pylori 908*
1	1	Proteobacteria	*Helicobacter pylori Aklavik117*
1	1	Proteobacteria	*Helicobacter pylori Aklavik86*
1	1	Proteobacteria	*Helicobacter pylori B38*
1	1	Proteobacteria	*Helicobacter pylori B8*
1	1	Proteobacteria	*Helicobacter pylori BM012A*
1	1	Proteobacteria	*Helicobacter pylori BM012S*
1	1	Proteobacteria	*Helicobacter pylori Cuz20*
1	1	Proteobacteria	*Helicobacter pylori ELS37*
1	1	Proteobacteria	*Helicobacter pylori F16*
1	1	Proteobacteria	*Helicobacter pylori F30*
1	1	Proteobacteria	*Helicobacter pylori F32*
1	1	Proteobacteria	*Helicobacter pylori F57*
1	1	Proteobacteria	*Helicobacter pylori G27*

1	1	Proteobacteria	*Helicobacter pylori Gambia94/24*
1	1	Proteobacteria	*Helicobacter pylori HPAG1*
1	1	Proteobacteria	*Helicobacter pylori HUP-B14*
1	1	Proteobacteria	*Helicobacter pylori India7*
1	1	Proteobacteria	*Helicobacter pylori J166*
1	1	Proteobacteria	*Helicobacter pylori J99*
1	1	Proteobacteria	*Helicobacter pylori J99*
1	1	Proteobacteria	*Helicobacter pylori Lithuania75*
1	1	Proteobacteria	*Helicobacter pylori NY40*
1	1	Proteobacteria	*Helicobacter pylori OK113*
1	1	Proteobacteria	*Helicobacter pylori OK310*
1	1	Proteobacteria	*Helicobacter pylori oki102*
1	1	Proteobacteria	*Helicobacter pylori oki112*
1	1	Proteobacteria	*Helicobacter pylori oki128*
1	1	Proteobacteria	*Helicobacter pylori oki154*
1	1	Proteobacteria	*Helicobacter pylori oki422*
1	1	Proteobacteria	*Helicobacter pylori oki673*
1	1	Proteobacteria	*Helicobacter pylori oki828*
1	1	Proteobacteria	*Helicobacter pylori oki898*
1	1	Proteobacteria	*Helicobacter pylori P12*
1	1	Proteobacteria	*Helicobacter pylori PeCan18*
1	1	Proteobacteria	*Helicobacter pylori PeCan4*
1	1	Proteobacteria	*Helicobacter pylori Puno120*
1	1	Proteobacteria	*Helicobacter pylori Puno135*
1	1	Proteobacteria	*Helicobacter pylori Rif1*
1	1	Proteobacteria	*Helicobacter pylori Rif2*
1	1	Proteobacteria	*Helicobacter pylori Sat464*
1	1	Proteobacteria	*Helicobacter pylori Shi112*
1	1	Proteobacteria	*Helicobacter pylori Shi169*

1	1	Proteobacteria	*Helicobacter pylori Shi417*
1	1	Proteobacteria	*Helicobacter pylori Shi470*
1	1	Proteobacteria	*Helicobacter pylori SJM180*
1	1	Proteobacteria	*Helicobacter pylori SNT49*
1	1	Proteobacteria	*Helicobacter pylori SouthAfrica20*
1	1	Proteobacteria	*Helicobacter pylori SouthAfrica7*
1	1	Proteobacteria	*Helicobacter pylori UM032*
1	1	Proteobacteria	*Helicobacter pylori UM037*
1	1	Proteobacteria	*Helicobacter pylori UM066*
1	1	Proteobacteria	*Helicobacter pylori UM298*
1	1	Proteobacteria	*Helicobacter pylori UM299*
1	1	Proteobacteria	*Helicobacter pylori v225d*
1	1	Proteobacteria	*Helicobacter pylori XZ274*
1	1	Proteobacteria	*Helicobacter typhlonius*
1	1	Proteobacteria	*Herbaspirillum hiltneri N3*
1	1	Proteobacteria	*Herbaspirillum rubrisubalbicans M1*
2	2	Proteobacteria	*Herbaspirillum seropedicae*
2	2	Proteobacteria	*Herbaspirillum seropedicae SmR1*
1	1	Proteobacteria	*Herminiimonas arsenicoxydans*
1	1	Proteobacteria	*Hippea maritima DSM 10411*
1	1	Proteobacteria	*Hirschia baltica ATCC 49814*
1	1	Proteobacteria	*Hoeflea sp. IMCC20628*
3	3	Proteobacteria	*Hyphomicrobium denitrificans 1NES1*
1	1	Proteobacteria	*Hyphomicrobium denitrificans ATCC 51888*
2	2	Proteobacteria	*Hyphomicrobium nitrativorans NL23*
1	1	Proteobacteria	*Hyphomicrobium sp. MC1*
1	1	Proteobacteria	*Hyphomonas neptunium ATCC 15444*
1	1	Proteobacteria	*Idiomarina loihiensis GSL 199*
1	1	Proteobacteria	*Idiomarina loihiensis L2TR*

1	1	Proteobacteria	*Jannaschia sp. CCS1*
1	1	Proteobacteria	*Janthinobacterium agaricidamnosum NBRC 102515 = DSM 9628*
1	1	Proteobacteria	*Janthinobacterium sp. B9-8*
1	1	Proteobacteria	*Janthinobacterium sp. Marseille*
1	1	Proteobacteria	*Kangiella geojedonensis*
1	1	Proteobacteria	*Kangiella koreensis DSM 16069*
1	1	Proteobacteria	*Ketogulonicigenium vulgare*
0	0	Proteobacteria	*Ketogulonicigenium vulgare WSH-001*
1	1	Proteobacteria	*Ketogulonicigenium vulgare Y25*
1	1	Proteobacteria	*Kingella kingae*
1	1	Proteobacteria	*Klebsiella michiganensis*
1	1	Proteobacteria	*Klebsiella oxytoca*
1	1	Proteobacteria	*Klebsiella oxytoca*
1	1	Proteobacteria	*Klebsiella oxytoca*
1	1	Proteobacteria	*Klebsiella oxytoca*
2	2	Proteobacteria	*Klebsiella oxytoca E718*
1	1	Proteobacteria	*Klebsiella oxytoca HKOPL1*
1	1	Proteobacteria	*Klebsiella oxytoca KCTC 1686*
1	1	Proteobacteria	*Klebsiella oxytoca KONIH1*
1	1	Proteobacteria	*Klebsiella pneumoniae*
1	1	Proteobacteria	*Klebsiella pneumoniae*
1	1	Proteobacteria	*Klebsiella pneumoniae*
1	1	Proteobacteria	*Klebsiella pneumoniae*
1	1	Proteobacteria	*Klebsiella pneumoniae*
1	1	Proteobacteria	*Klebsiella pneumoniae*
1	1	Proteobacteria	*Klebsiella pneumoniae*
1	1	Proteobacteria	*Klebsiella pneumoniae*
1	1	Proteobacteria	*Klebsiella pneumoniae*

1	1	Proteobacteria	*Klebsiella pneumoniae*
1	1	Proteobacteria	*Klebsiella pneumoniae*
1	1	Proteobacteria	*Klebsiella pneumoniae*
1	1	Proteobacteria	*Klebsiella pneumoniae*
1	1	Proteobacteria	*Klebsiella pneumoniae*
1	4	Proteobacteria	*Klebsiella pneumoniae*
2	2	Proteobacteria	*Klebsiella pneumoniae*
2	2	Proteobacteria	*Klebsiella pneumoniae*
1	1	Proteobacteria	*Klebsiella pneumoniae 30660/NJST258_1*
1	1	Proteobacteria	*Klebsiella pneumoniae 30684/NJST258_2*
1	1	Proteobacteria	*Klebsiella pneumoniae 342*
1	1	Proteobacteria	*Klebsiella pneumoniae 500_1420*
1	1	Proteobacteria	*Klebsiella pneumoniae CG43*
1	1	Proteobacteria	*Klebsiella pneumoniae DMC1097*
1	1	Proteobacteria	*Klebsiella pneumoniae HK787*
1	1	Proteobacteria	*Klebsiella pneumoniae JM45*
1	1	Proteobacteria	*Klebsiella pneumoniae KCTC 2242*
1	1	Proteobacteria	*Klebsiella pneumoniae KP-1*
1	1	Proteobacteria	*Klebsiella pneumoniae subsp. pneumoniae*
1	1	Proteobacteria	*Klebsiella pneumoniae subsp. pneumoniae*
1	1	Proteobacteria	*Klebsiella pneumoniae subsp. pneumoniae*
1	1	Proteobacteria	*Klebsiella pneumoniae subsp. pneumoniae*
1	1	Proteobacteria	*Klebsiella pneumoniae subsp. pneumoniae*
1	1	Proteobacteria	*Klebsiella pneumoniae subsp. pneumoniae*
1	1	Proteobacteria	*Klebsiella pneumoniae subsp. pneumoniae*
1	1	Proteobacteria	*Klebsiella pneumoniae subsp. pneumoniae*
1	1	Proteobacteria	*Klebsiella pneumoniae subsp. pneumoniae*
1	1	Proteobacteria	*Klebsiella pneumoniae subsp. pneumoniae*
1	1	Proteobacteria	*Klebsiella pneumoniae subsp. pneumoniae*

1	1	Proteobacteria	*Klebsiella pneumoniae subsp. pneumoniae 1084*
1	1	Proteobacteria	*Klebsiella pneumoniae subsp. pneumoniae 1158*
1	1	Proteobacteria	*Klebsiella pneumoniae subsp. pneumoniae HS11286*
1	1	Proteobacteria	*Klebsiella pneumoniae subsp. pneumoniae Kp13*
1	1	Proteobacteria	*Klebsiella pneumoniae subsp. pneumoniae KPNIH1*
1	1	Proteobacteria	*Klebsiella pneumoniae subsp. pneumoniae KPNIH10*
1	1	Proteobacteria	*Klebsiella pneumoniae subsp. pneumoniae KPNIH24*
1	1	Proteobacteria	*Klebsiella pneumoniae subsp. pneumoniae KPNIH27*
1	1	Proteobacteria	*Klebsiella pneumoniae subsp. pneumoniae KPR0928*
1	1	Proteobacteria	*Klebsiella pneumoniae subsp. pneumoniae MGH 78578*
1	1	Proteobacteria	*Klebsiella pneumoniae subsp. pneumoniae NTUH-K2044*
2	2	Proteobacteria	*Klebsiella pneumoniae subsp. pneumoniae PittNDM01*
1	1	Proteobacteria	*Klebsiella pneumoniae UHKPC07*
1	1	Proteobacteria	*Klebsiella pneumoniae UHKPC33*
1	1	Proteobacteria	*Klebsiella quasipneumoniae*
1	1	Proteobacteria	*Klebsiella sp. G5*
1	1	Proteobacteria	*Klebsiella variicola*
1	1	Proteobacteria	*Klebsiella variicola*
1	1	Proteobacteria	*Klebsiella variicola*
1	1	Proteobacteria	*Klebsiella variicola At-22*
1	1	Proteobacteria	*Kluyvera intermedia*
1	1	Proteobacteria	*Komagataeibacter medellinensis NBRC 3288*
1	1	Proteobacteria	*Komagataeibacter xylinus E25*
1	1	Proteobacteria	*Kosakonia sacchari SP1*
1	1	Proteobacteria	*Labrenzia sp. CP4*
1	1	Proteobacteria	*Lacimicrobium alkaliphilum*
2	2	Proteobacteria	*Laribacter hongkongensis HLHK9*
1	1	Proteobacteria	*Lawsonia intracellularis N343*
1	1	Proteobacteria	*Lawsonia intracellularis PHE/MN1-00*

1	1	Proteobacteria	*Leclercia adecarboxylata*
1	1	Proteobacteria	*Legionella fallonii LLAP-10*
1	1	Proteobacteria	*Legionella hackeliae*
1	1	Proteobacteria	*Legionella longbeachae NSW150*
1	1	Proteobacteria	*Legionella oakridgensis ATCC 33761 = DSM 21215*
1	1	Proteobacteria	*Legionella pneumophila 2300/99 Alcoy*
1	1	Proteobacteria	*Legionella pneumophila str. Corby*
1	1	Proteobacteria	*Legionella pneumophila str. Lens*
1	1	Proteobacteria	*Legionella pneumophila str. Paris*
1	1	Proteobacteria	*Legionella pneumophila subsp. pascullei*
1	1	Proteobacteria	*Legionella pneumophila subsp. pascullei*
1	1	Proteobacteria	*Legionella pneumophila subsp. pascullei*
1	1	Proteobacteria	*Legionella pneumophila subsp. pneumophila*
1	1	Proteobacteria	*Legionella pneumophila subsp. pneumophila*
1	1	Proteobacteria	*Legionella pneumophila subsp. pneumophila*
1	1	Proteobacteria	*Legionella pneumophila subsp. pneumophila ATCC 43290*
1	1	Proteobacteria	*Legionella pneumophila subsp. pneumophila LPE509*
1	1	Proteobacteria	*Legionella pneumophila subsp. pneumophila str. Hextuple_2q*
1	1	Proteobacteria	*Legionella pneumophila subsp. pneumophila str. Hextuple_3a*
1	1	Proteobacteria	*Legionella pneumophila subsp. pneumophila str. Philadelphia 1*
2	2	Proteobacteria	*Leisingera methylohalidivorans DSM 14336*
1	1	Proteobacteria	*Leptothrix cholodnii SP-6*
1	1	Proteobacteria	*Liberibacter crescens*
1	1	Proteobacteria	*Liberibacter crescens BT-1*
0	1	Proteobacteria	*Limnohabitans sp. 103DPR2*
1	1	Proteobacteria	*Limnohabitans sp. 63ED37-2*

1	1	Proteobacteria	*Listonella anguillarum M3*
1	1	Proteobacteria	*Lysobacter antibioticus*
1	1	Proteobacteria	*Lysobacter antibioticus*
1	1	Proteobacteria	*Lysobacter capsici*
1	1	Proteobacteria	*Lysobacter gummosus*
1	1	Proteobacteria	*Magnetococcus marinus MC-1*
1	1	Proteobacteria	*Magnetospira sp. QH-2*
1	1	Proteobacteria	*Magnetospirillum gryphiswaldense MSR-1 v2*
1	1	Proteobacteria	*Magnetospirillum magneticum AMB-1*
1	1	Proteobacteria	*Mannheimia haemolytica*
1	1	Proteobacteria	*Mannheimia haemolytica*
1	1	Proteobacteria	*Mannheimia haemolytica D153*
1	1	Proteobacteria	*Mannheimia haemolytica D171*
1	1	Proteobacteria	*Mannheimia haemolytica D174*
1	1	Proteobacteria	*Mannheimia haemolytica M42548*
1	1	Proteobacteria	*Mannheimia haemolytica USDA-ARS-USMARC-183*
1	1	Proteobacteria	*Mannheimia haemolytica USDA-ARS-USMARC-184*
1	1	Proteobacteria	*Mannheimia haemolytica USDA-ARS-USMARC-185*
1	1	Proteobacteria	*Mannheimia haemolytica USMARC_2286*
1	1	Proteobacteria	*Mannheimia succiniciproducens MBEL55E*
1	1	Proteobacteria	*Mannheimia varigena USDA-ARS-USMARC-1261*
1	1	Proteobacteria	*Mannheimia varigena USDA-ARS-USMARC-1296*
1	1	Proteobacteria	*Mannheimia varigena USDA-ARS-USMARC-1312*
1	1	Proteobacteria	*Mannheimia varigena USDA-ARS-USMARC-1388*
1	1	Proteobacteria	*Maricaulis maris MCS10*
1	1	Proteobacteria	*Marichromatium purpuratum 984*
1	1	Proteobacteria	*Marinobacter adhaerens HP15*
1	1	Proteobacteria	*Marinobacter hydrocarbonoclasticus ATCC 49840*
1	1	Proteobacteria	*Marinobacter hydrocarbonoclasticus VT8*

1	1	Proteobacteria	*Marinobacter psychrophilus*
0	1	Proteobacteria	*Marinobacter salarius*
1	1	Proteobacteria	*Marinobacter similis*
0	1	Proteobacteria	*Marinobacter sp. BSs20148*
1	1	Proteobacteria	*Marinobacter sp. CP1*
1	1	Proteobacteria	*Marinomonas mediterranea MMB-1*
1	1	Proteobacteria	*Marinomonas posidonica IVIA-Po-181*
1	1	Proteobacteria	*Marinomonas sp. MWYL1*
2	1	Proteobacteria	*Marinovum algicola DG 898*
1	1	Proteobacteria	*Martelella endophytica*
1	1	Proteobacteria	*Martelella sp. AD-3*
1	1	Proteobacteria	*Massilia sp. NR 4-1*
1	1	Proteobacteria	*Massilia sp. WG5*
3	3	Proteobacteria	*Mesorhizobium australicum WSM2073*
4	4	Proteobacteria	*Mesorhizobium ciceri biovar biserrulae WSM1271*
5	5	Proteobacteria	*Mesorhizobium loti MAFF303099*
4	4	Proteobacteria	*Mesorhizobium opportunistum WSM2075*
2	1	Proteobacteria	*Methylibium petroleiphilum PM1*
1	1	Proteobacteria	*Methylobacillus flagellatus KT*
1	1	Proteobacteria	*Methylobacterium aquaticum*
2	2	Proteobacteria	*Methylobacterium extorquens AM1*
2	2	Proteobacteria	*Methylobacterium extorquens CM4*
2	2	Proteobacteria	*Methylobacterium extorquens DM4*
3	3	Proteobacteria	*Methylobacterium extorquens PA1*
3	3	Proteobacteria	*Methylobacterium nodulans ORS 2060*
2	2	Proteobacteria	*Methylobacterium oryzae CBMB20*
1	1	Proteobacteria	*Methylobacterium populi BJ001*
1	1	Proteobacteria	*Methylobacterium radiotolerans JCM 2831*
3	3	Proteobacteria	*Methylobacterium sp. 4-46*

2	2	Proteobacteria	*Methylobacterium sp. AMS5*
2	2	Proteobacteria	*Methyloceanibacter caenitepidi*
3	1	Proteobacteria	*Methylocella silvestris BL2*
3	2	Proteobacteria	*Methylococcus capsulatus str. Bath*
1	1	Proteobacteria	*Methylocystis sp. SC2*
1	1	Proteobacteria	*Methylomicrobium alcaliphilum 20Z*
1	1	Proteobacteria	*Methylomonas denitrificans*
1	1	Proteobacteria	*Methylomonas methanica MC09*
1	1	Proteobacteria	*Methylophaga frappieri*
1	1	Proteobacteria	*Methylophaga nitratireducenticrescens*
2	2	Proteobacteria	*Methylophilus sp. TWE2*
1	1	Proteobacteria	*Methylotenera mobilis JLW8*
1	1	Proteobacteria	*Methylotenera versatilis 301*
2	2	Proteobacteria	*Methylovorus glucosetrophus SIP3-4*
2	2	Proteobacteria	*Methylovorus sp. MP688*
1	1	Proteobacteria	*Micavibrio aeruginosavorus ARL-13*
1	1	Proteobacteria	*Micavibrio aeruginosavorus EPB*
1	1	Proteobacteria	*Moraxella catarrhalis*
1	1	Proteobacteria	*Moraxella catarrhalis*
1	1	Proteobacteria	*Moraxella catarrhalis BBH18*
1	1	Proteobacteria	*Moraxella osloensis*
1	1	Proteobacteria	*Morganella morganii*
1	1	Proteobacteria	*Morganella morganii subsp. morganii KT*
1	1	Proteobacteria	*Moritella viscosa*
2	1	Proteobacteria	*Myxococcus fulvus 124B02*
2	1	Proteobacteria	*Myxococcus fulvus HW-1*
2	1	Proteobacteria	*Myxococcus hansupus*
2	1	Proteobacteria	*Myxococcus stipitatus DSM 14675*
2	1	Proteobacteria	*Myxococcus xanthus DK 1622*

1	1	Proteobacteria	*Nautilia profundicola AmH*
1	1	Proteobacteria	*Neisseria elongata subsp. glycolytica ATCC 29315*
1	1	Proteobacteria	*Neisseria gonorrhoeae*
1	1	Proteobacteria	*Neisseria gonorrhoeae*
1	1	Proteobacteria	*Neisseria gonorrhoeae*
1	1	Proteobacteria	*Neisseria gonorrhoeae FA 1090*
1	1	Proteobacteria	*Neisseria gonorrhoeae MS11*
1	1	Proteobacteria	*Neisseria gonorrhoeae NCCP11945*
1	1	Proteobacteria	*Neisseria lactamica 020-06*
1	1	Proteobacteria	*Neisseria meningitidis*
1	1	Proteobacteria	*Neisseria meningitidis*
1	1	Proteobacteria	*Neisseria meningitidis*
1	1	Proteobacteria	*Neisseria meningitidis*
1	1	Proteobacteria	*Neisseria meningitidis*
1	1	Proteobacteria	*Neisseria meningitidis*
1	1	Proteobacteria	*Neisseria meningitidis 053442*
1	1	Proteobacteria	*Neisseria meningitidis 8013*
1	1	Proteobacteria	*Neisseria meningitidis alpha14*
1	1	Proteobacteria	*Neisseria meningitidis alpha710*
1	1	Proteobacteria	*Neisseria meningitidis FAM18*
1	1	Proteobacteria	*Neisseria meningitidis G2136*
1	1	Proteobacteria	*Neisseria meningitidis H44/76*
1	1	Proteobacteria	*Neisseria meningitidis LNP21362*
1	1	Proteobacteria	*Neisseria meningitidis M01-240149*
1	1	Proteobacteria	*Neisseria meningitidis M01-240355*
1	1	Proteobacteria	*Neisseria meningitidis M04-240196*
1	1	Proteobacteria	*Neisseria meningitidis M0579*
1	1	Proteobacteria	*Neisseria meningitidis M7124*
1	1	Proteobacteria	*Neisseria meningitidis MC58*

1	1	Proteobacteria	*Neisseria meningitidis NZ-05/33*
1	1	Proteobacteria	*Neisseria meningitidis WUE 2594*
1	1	Proteobacteria	*Neisseria meningitidis Z2491*
3	3	Proteobacteria	*Neorhizobium galegae bv. officinalis bv. officinalis str. HAMBI 1141*
2	2	Proteobacteria	*Neorhizobium galegae bv. orientalis str. HAMBI 540*
1	1	Proteobacteria	*Neorickettsia helminthoeca str. Oregon*
1	1	Proteobacteria	*Neorickettsia risticii str. Illinois*
1	1	Proteobacteria	*Neorickettsia sennetsu str. Miyayama*
1	1	Proteobacteria	*Nitratifractor salsuginis DSM 16511*
1	1	Proteobacteria	*Nitratiruptor sp. SB155-2*
3	3	Proteobacteria	*Nitrobacter hamburgensis X14*
3	3	Proteobacteria	*Nitrobacter winogradskyi Nb-255*
2	1	Proteobacteria	*Nitrosococcus halophilus Nc 4*
1	1	Proteobacteria	*Nitrosococcus oceani ATCC 19707*
1	1	Proteobacteria	*Nitrosococcus watsonii C-113*
2	1	Proteobacteria	*Nitrosomonas communis*
1	1	Proteobacteria	*Nitrosomonas europaea ATCC 19718*
1	1	Proteobacteria	*Nitrosomonas eutropha C91*
1	1	Proteobacteria	*Nitrosomonas sp. AL212*
2	2	Proteobacteria	*Nitrosomonas sp. Is79A3*
1	1	Proteobacteria	*Nitrosomonas ureae*
1	1	Proteobacteria	*Nitrosospira briensis C-128*
1	1	Proteobacteria	*Nitrosospira multiformis ATCC 25196*
1	1	Proteobacteria	*Novosphingobium aromaticivorans DSM 12444*
2	2	Proteobacteria	*Novosphingobium pentaromativorans US6-1*
1	1	Proteobacteria	*Novosphingobium sp. PP1Y*
1	1	Proteobacteria	*Obesumbacterium proteus*
1	1	Proteobacteria	*Oceanimonas sp. GK1*

1	1	Proteobacteria	*Ochrobactrum anthropi*
1	1	Proteobacteria	*Ochrobactrum anthropi ATCC 49188*
1	1	Proteobacteria	*Octadecabacter antarcticus 307*
3	3	Proteobacteria	*Octadecabacter arcticus 238*
1	1	Proteobacteria	*Octadecabacter temperatus*
1	1	Proteobacteria	*Oleispira antarctica RB-8*
2	2	Proteobacteria	*Oligotropha carboxidovorans OM4*
2	2	Proteobacteria	*Oligotropha carboxidovorans OM5*
2	2	Proteobacteria	*Oligotropha carboxidovorans OM5*
1	1	Proteobacteria	*Orientia tsutsugamushi str. Boryong*
1	1	Proteobacteria	*Orientia tsutsugamushi str. Ikeda*
1	1	Proteobacteria	*Ottowia sp. oral taxon 894*
2	2	Proteobacteria	*Pandoraea apista*
2	2	Proteobacteria	*Pandoraea apista*
2	2	Proteobacteria	*Pandoraea apista*
1	1	Proteobacteria	*Pandoraea faecigallinarum*
2	2	Proteobacteria	*Pandoraea norimbergensis*
1	1	Proteobacteria	*Pandoraea oxalativorans*
1	1	Proteobacteria	*Pandoraea pnomenusa*
1	1	Proteobacteria	*Pandoraea pnomenusa*
1	1	Proteobacteria	*Pandoraea pnomenusa 3kgm*
1	1	Proteobacteria	*Pandoraea pulmonicola*
1	1	Proteobacteria	*Pandoraea sputorum*
1	1	Proteobacteria	*Pandoraea thiooxydans*
2	2	Proteobacteria	*Pandoraea vervacti*
2	2	Proteobacteria	*Pannonibacter phragmitetus*
1	1	Proteobacteria	*Pantoea agglomerans*
1	1	Proteobacteria	*Pantoea ananatis LMG 20103*
1	1	Proteobacteria	*Pantoea ananatis LMG 5342*

1	1	Proteobacteria	*Pantoea ananatis PA13*
1	1	Proteobacteria	*Pantoea rwandensis*
1	1	Proteobacteria	*Pantoea sp. At-9b*
1	1	Proteobacteria	*Pantoea sp. PSNIH1*
1	1	Proteobacteria	*Pantoea sp. PSNIH2*
1	1	Proteobacteria	*Pantoea vagans C9-1*
4	3	Proteobacteria	*Paraburkholderia caribensis*
3	2	Proteobacteria	*Paraburkholderia caribensis MBA4*
2	2	Proteobacteria	*Paraburkholderia fungorum*
3	3	Proteobacteria	*Paraburkholderia phenoliruptrix BR3459a*
1	1	Proteobacteria	*Paraburkholderia phymatum STM815*
1	1	Proteobacteria	*Paraburkholderia phytofirmans PsJN*
1	1	Proteobacteria	*Paraburkholderia rhizoxinica HKI 454*
4	4	Proteobacteria	*Paraburkholderia xenovorans LB400*
5	5	Proteobacteria	*Paraburkholderia xenovorans LB400*
2	1	Proteobacteria	*Paracoccus aminophilus JCM 7686*
2	2	Proteobacteria	*Paracoccus denitrificans PD1222*
1	1	Proteobacteria	*Paraglaciecola psychrophila 170*
1	1	Proteobacteria	*Pararhodospirillum photometricum DSM 122*
1	1	Proteobacteria	*Parvibaculum lavamentivorans DS-1*
1	1	Proteobacteria	*Parvularcula bermudensis HTCC2503*
1	1	Proteobacteria	*Pasteurella multocida*
1	1	Proteobacteria	*Pasteurella multocida 36950*
1	1	Proteobacteria	*Pasteurella multocida OH1905*
1	1	Proteobacteria	*Pasteurella multocida subsp. multocida OH4807*
1	1	Proteobacteria	*Pasteurella multocida subsp. multocida PMTB2.1*
1	1	Proteobacteria	*Pasteurella multocida subsp. multocida str. 3480*
1	1	Proteobacteria	*Pasteurella multocida subsp. multocida str. HB03*
1	1	Proteobacteria	*Pasteurella multocida subsp. multocida str. HN06*

1	1	Proteobacteria	*Pasteurella multocida subsp. multocida str. Pm70*
1	1	Proteobacteria	*Paucibacter sp. KCTC 42545*
1	1	Proteobacteria	*Pectobacterium atrosepticum*
1	1	Proteobacteria	*Pectobacterium atrosepticum*
1	1	Proteobacteria	*Pectobacterium atrosepticum SCRI1043*
1	1	Proteobacteria	*Pectobacterium carotovorum subsp. carotovorum PC1*
1	1	Proteobacteria	*Pectobacterium carotovorum subsp. carotovorum PCC21*
1	1	Proteobacteria	*Pectobacterium sp. SCC3193*
1	1	Proteobacteria	*Pectobacterium wasabiae WPP163*
1	1	Proteobacteria	*Pelagibacterium halotolerans B2*
1	1	Proteobacteria	*Pelobacter carbinolicus DSM 2380*
1	1	Proteobacteria	*Pelobacter propionicus DSM 2379*
1	1	Proteobacteria	*Phaeobacter gallaeciensis 2.10*
2	1	Proteobacteria	*Phaeobacter gallaeciensis DSM 26640*
1	1	Proteobacteria	*Phaeobacter inhibens DSM 17395*
2	2	Proteobacteria	*Phenylobacterium zucineum HLK1*
2	2	Proteobacteria	*Photobacterium gaetbulicola Gung47*
1	1	Proteobacteria	*Photorhabdus asymbiotica*
1	1	Proteobacteria	*Photorhabdus temperata subsp. thracensis*
1	1	Proteobacteria	*Piscirickettsia salmonis*
1	1	Proteobacteria	*Piscirickettsia salmonis*
1	1	Proteobacteria	*Piscirickettsia salmonis*
1	1	Proteobacteria	*Piscirickettsia salmonis*
1	1	Proteobacteria	*Piscirickettsia salmonis LF-89 = ATCC VR-1361*
1	1	Proteobacteria	*Planktomarina temperata RCA23*
1	1	Proteobacteria	*Plautia stali symbiont*
1	1	Proteobacteria	*Pluralibacter gergoviae*
2	1	Proteobacteria	*Polaromonas naphthalenivorans CJ2*
1	1	Proteobacteria	*Polaromonas sp. JS666*

1	1	Proteobacteria	*Polymorphum gilvum SL003B-26A1*
1	1	Proteobacteria	*Polynucleobacter necessarius subsp. asymbioticus*
1	1	Proteobacteria	*Polynucleobacter necessarius subsp. asymbioticus QLW-P1DMWA-1*
1	1	Proteobacteria	*Polynucleobacter necessarius subsp. necessarius STIR1*
1	1	Proteobacteria	*Pragia fontium*
1	1	Proteobacteria	*Proteus mirabilis*
1	1	Proteobacteria	*Proteus mirabilis BB2000*
1	1	Proteobacteria	*Proteus mirabilis HI4320*
1	1	Proteobacteria	*Proteus vulgaris*
1	1	Proteobacteria	*Providencia stuartii*
1	1	Proteobacteria	*Providencia stuartii*
1	1	Proteobacteria	*Providencia stuartii MRSN 2154*
1	1	Proteobacteria	*Pseudoalteromonas atlantica T6c*
1	1	Proteobacteria	*Pseudoalteromonas haloplanktis TAC125*
1	1	Proteobacteria	*Pseudoalteromonas issachenkonii*
1	1	Proteobacteria	*Pseudoalteromonas phenolica*
1	1	Proteobacteria	*Pseudoalteromonas rubra*
1	1	Proteobacteria	*Pseudoalteromonas sp. Bsw20308*
1	1	Proteobacteria	*Pseudoalteromonas sp. OCN003*
1	1	Proteobacteria	*Pseudoalteromonas sp. SM9913*
1	1	Proteobacteria	*Pseudoalteromonas translucida KMM 520*
1	1	Proteobacteria	*Pseudogulbenkiania sp. NH8B*
1	1	Proteobacteria	*Pseudohongiella spirulinae*
1	1	Proteobacteria	*Pseudomonadaceae bacterium B4199*
1	1	Proteobacteria	*Pseudomonadaceae bacterium C6819*
1	1	Proteobacteria	*Pseudomonadaceae bacterium C6918*
1	1	Proteobacteria	*Pseudomonadaceae bacterium D3318*
1	1	Proteobacteria	*Pseudomonadaceae bacterium E1086*

1	1	Proteobacteria	*Pseudomonadaceae bacterium E1148*
1	1	Proteobacteria	*Pseudomonas aeruginosa*
1	1	Proteobacteria	*Pseudomonas aeruginosa*
1	1	Proteobacteria	*Pseudomonas aeruginosa*
1	1	Proteobacteria	*Pseudomonas aeruginosa*
1	1	Proteobacteria	*Pseudomonas aeruginosa*
1	1	Proteobacteria	*Pseudomonas aeruginosa*
1	1	Proteobacteria	*Pseudomonas aeruginosa*
1	1	Proteobacteria	*Pseudomonas aeruginosa*
1	1	Proteobacteria	*Pseudomonas aeruginosa*
1	1	Proteobacteria	*Pseudomonas aeruginosa*
1	1	Proteobacteria	*Pseudomonas aeruginosa*
1	1	Proteobacteria	*Pseudomonas aeruginosa*
1	1	Proteobacteria	*Pseudomonas aeruginosa*
1	1	Proteobacteria	*Pseudomonas aeruginosa*
1	1	Proteobacteria	*Pseudomonas aeruginosa*
1	1	Proteobacteria	*Pseudomonas aeruginosa*
1	1	Proteobacteria	*Pseudomonas aeruginosa*
1	1	Proteobacteria	*Pseudomonas aeruginosa*
1	1	Proteobacteria	*Pseudomonas aeruginosa*
1	1	Proteobacteria	*Pseudomonas aeruginosa*
1	1	Proteobacteria	*Pseudomonas aeruginosa*
1	1	Proteobacteria	*Pseudomonas aeruginosa*
1	1	Proteobacteria	*Pseudomonas aeruginosa*
1	1	Proteobacteria	*Pseudomonas aeruginosa*
1	1	Proteobacteria	*Pseudomonas aeruginosa*
1	1	Proteobacteria	*Pseudomonas aeruginosa*
1	1	Proteobacteria	*Pseudomonas aeruginosa*
1	1	Proteobacteria	*Pseudomonas aeruginosa*

1	1	Proteobacteria	*Pseudomonas aeruginosa*
1	1	Proteobacteria	*Pseudomonas aeruginosa*
1	1	Proteobacteria	*Pseudomonas aeruginosa*
1	1	Proteobacteria	*Pseudomonas aeruginosa 19BR*
1	1	Proteobacteria	*Pseudomonas aeruginosa 213BR*
1	1	Proteobacteria	*Pseudomonas aeruginosa AES-1R*
1	1	Proteobacteria	*Pseudomonas aeruginosa B136-33*
1	1	Proteobacteria	*Pseudomonas aeruginosa DHS01*
1	1	Proteobacteria	*Pseudomonas aeruginosa DK2*
1	1	Proteobacteria	*Pseudomonas aeruginosa DSM 50071*
1	1	Proteobacteria	*Pseudomonas aeruginosa LES431*
1	1	Proteobacteria	*Pseudomonas aeruginosa LESB58*
1	1	Proteobacteria	*Pseudomonas aeruginosa M18*
1	1	Proteobacteria	*Pseudomonas aeruginosa MTB-1*
1	1	Proteobacteria	*Pseudomonas aeruginosa NCGM2.S1*
1	1	Proteobacteria	*Pseudomonas aeruginosa PA1*
1	1	Proteobacteria	*Pseudomonas aeruginosa PA1R*
1	1	Proteobacteria	*Pseudomonas aeruginosa PA7*
1	1	Proteobacteria	*Pseudomonas aeruginosa PACS2*
1	1	Proteobacteria	*Pseudomonas aeruginosa PAO1*
1	1	Proteobacteria	*Pseudomonas aeruginosa RP73*
1	1	Proteobacteria	*Pseudomonas aeruginosa SCV20265*
1	1	Proteobacteria	*Pseudomonas aeruginosa UCBPP-PA14*
0	1	Proteobacteria	*Pseudomonas aeruginosa VRFPA04*
1	1	Proteobacteria	*Pseudomonas aeruginosa YL84*
1	1	Proteobacteria	*Pseudomonas alcaligenes*
1	1	Proteobacteria	*Pseudomonas alkylphenolia*
1	1	Proteobacteria	*Pseudomonas azotoformans*
2	1	Proteobacteria	*Pseudomonas balearica DSM 6083*

1	1	Proteobacteria	*Pseudomonas brassicacearum*
1	1	Proteobacteria	*Pseudomonas brassicacearum*
1	1	Proteobacteria	*Pseudomonas brassicacearum subsp. brassicacearum NFM421*
1	1	Proteobacteria	*Pseudomonas chlororaphis*
1	1	Proteobacteria	*Pseudomonas chlororaphis*
1	1	Proteobacteria	*Pseudomonas chlororaphis*
2	1	Proteobacteria	*Pseudomonas chlororaphis*
1	1	Proteobacteria	*Pseudomonas chlororaphis subsp. aurantiaca*
1	1	Proteobacteria	*Pseudomonas cichorii JBC1*
1	1	Proteobacteria	*Pseudomonas citronellolis*
1	1	Proteobacteria	*Pseudomonas cremoricolorata*
1	1	Proteobacteria	*Pseudomonas denitrificans ATCC 13867*
1	1	Proteobacteria	*Pseudomonas entomophila L48*
1	1	Proteobacteria	*Pseudomonas fluorescens*
1	1	Proteobacteria	*Pseudomonas fluorescens*
1	1	Proteobacteria	*Pseudomonas fluorescens*
1	1	Proteobacteria	*Pseudomonas fluorescens*
2	1	Proteobacteria	*Pseudomonas fluorescens*
1	1	Proteobacteria	*Pseudomonas fluorescens A506*
1	1	Proteobacteria	*Pseudomonas fluorescens F113*
2	1	Proteobacteria	*Pseudomonas fluorescens NCIMB 11764*
1	1	Proteobacteria	*Pseudomonas fluorescens Pf0-1*
1	1	Proteobacteria	*Pseudomonas fluorescens PICF7*
1	1	Proteobacteria	*Pseudomonas fluorescens SBW25*
1	1	Proteobacteria	*Pseudomonas fragi*
1	1	Proteobacteria	*Pseudomonas fulva 12-X*
2	1	Proteobacteria	*Pseudomonas knackmussii B13*
2	1	Proteobacteria	*Pseudomonas mandelii JR-1*

1	1	Proteobacteria	*Pseudomonas mendocina NK-01*
1	1	Proteobacteria	*Pseudomonas mendocina S5.2*
1	1	Proteobacteria	*Pseudomonas mendocina ymp*
1	1	Proteobacteria	*Pseudomonas monteilii*
2	1	Proteobacteria	*Pseudomonas monteilii SB3078*
2	1	Proteobacteria	*Pseudomonas monteilii SB3101*
1	1	Proteobacteria	*Pseudomonas mosselii SJ10*
1	1	Proteobacteria	*Pseudomonas oryzihabitans*
1	1	Proteobacteria	*Pseudomonas parafulva*
2	1	Proteobacteria	*Pseudomonas plecoglossicida*
1	1	Proteobacteria	*Pseudomonas poae RE*1-1-14*
1	1	Proteobacteria	*Pseudomonas protegens Cab57*
1	1	Proteobacteria	*Pseudomonas protegens CHA0*
1	1	Proteobacteria	*Pseudomonas protegens Pf-5*
1	1	Proteobacteria	*Pseudomonas pseudoalcaligenes*
1	1	Proteobacteria	*Pseudomonas pseudoalcaligenes CECT 5344*
1	1	Proteobacteria	*Pseudomonas putida*
1	1	Proteobacteria	*Pseudomonas putida*
1	1	Proteobacteria	*Pseudomonas putida BIRD-1*
1	1	Proteobacteria	*Pseudomonas putida F1*
1	1	Proteobacteria	*Pseudomonas putida GB-1*
1	1	Proteobacteria	*Pseudomonas putida H8234*
1	1	Proteobacteria	*Pseudomonas putida HB3267*
1	1	Proteobacteria	*Pseudomonas putida KT2440*
1	1	Proteobacteria	*Pseudomonas putida NBRC 14164*
1	1	Proteobacteria	*Pseudomonas putida ND6*
1	1	Proteobacteria	*Pseudomonas putida S12*
1	1	Proteobacteria	*Pseudomonas putida S13.1.2*
1	1	Proteobacteria	*Pseudomonas putida S16*

1	1	Proteobacteria	*Pseudomonas putida W619*
1	1	Proteobacteria	*Pseudomonas resinovorans NBRC 106553*
1	1	Proteobacteria	*Pseudomonas rhizosphaerae*
1	1	Proteobacteria	*Pseudomonas savastanoi pv. phaseolicola 1448A*
1	1	Proteobacteria	*Pseudomonas sp. 20_BN*
1	1	Proteobacteria	*Pseudomonas sp. CCOS 191*
1	1	Proteobacteria	*Pseudomonas sp. FGI182*
1	1	Proteobacteria	*Pseudomonas sp. L10.10*
1	1	Proteobacteria	*Pseudomonas sp. Os17*
1	1	Proteobacteria	*Pseudomonas sp. St29*
1	1	Proteobacteria	*Pseudomonas sp. StFLB209*
2	1	Proteobacteria	*Pseudomonas sp. TKP*
1	1	Proteobacteria	*Pseudomonas sp. URMO17WK12:I11*
2	1	Proteobacteria	*Pseudomonas sp. URMO17WK12:I11*
1	1	Proteobacteria	*Pseudomonas sp. UW4*
2	1	Proteobacteria	*Pseudomonas sp. VLB120*
1	1	Proteobacteria	*Pseudomonas stutzeri*
1	1	Proteobacteria	*Pseudomonas stutzeri*
2	1	Proteobacteria	*Pseudomonas stutzeri*
1	1	Proteobacteria	*Pseudomonas stutzeri A1501*
1	1	Proteobacteria	*Pseudomonas stutzeri ATCC 17588 = LMG 11199*
1	1	Proteobacteria	*Pseudomonas stutzeri CCUG 29243*
1	1	Proteobacteria	*Pseudomonas stutzeri DSM 10701*
1	1	Proteobacteria	*Pseudomonas stutzeri DSM 4166*
1	1	Proteobacteria	*Pseudomonas stutzeri RCH2*
1	1	Proteobacteria	*Pseudomonas syringae CC1557*
1	1	Proteobacteria	*Pseudomonas syringae pv. actinidiae ICMP 18884*
1	1	Proteobacteria	*Pseudomonas syringae pv. lapsa*
1	1	Proteobacteria	*Pseudomonas syringae pv. syringae B301D*

1	1	Proteobacteria	*Pseudomonas syringae pv. syringae B728a*
1	1	Proteobacteria	*Pseudomonas syringae pv. syringae HS191*
1	1	Proteobacteria	*Pseudomonas syringae UMAF0158*
1	1	Proteobacteria	*Pseudomonas trivialis*
1	1	Proteobacteria	*Pseudoxanthomonas spadix BD-a59*
1	1	Proteobacteria	*Pseudoxanthomonas suwonensis*
1	1	Proteobacteria	*Pseudoxanthomonas suwonensis 11-1*
1	1	Proteobacteria	*Psychrobacter arcticus 273-4*
1	1	Proteobacteria	*Psychrobacter cryohalolentis K5*
1	1	Proteobacteria	*Psychrobacter sp. G*
1	1	Proteobacteria	*Psychrobacter sp. PRwf-1*
1	1	Proteobacteria	*Psychrobacter urativorans*
3	2	Proteobacteria	*Psychromonas ingrahamii 37*
1	1	Proteobacteria	*Psychromonas sp. CNPT3*
1	1	Proteobacteria	*Pusillimonas sp. T7-7*
1	1	Proteobacteria	*Rahnella aquatilis HX2*
1	1	Proteobacteria	*Rahnella sp. Y9602*
2	1	Proteobacteria	*Ralstonia eutropha H16*
2	1	Proteobacteria	*Ralstonia eutropha JMP134*
2	2	Proteobacteria	*Ralstonia mannitolilytica*
4	2	Proteobacteria	*Ralstonia pickettii 12D*
4	2	Proteobacteria	*Ralstonia pickettii 12J*
4	3	Proteobacteria	*Ralstonia pickettii DTP0602*
1	1	Proteobacteria	*Ralstonia solanacearum*
1	1	Proteobacteria	*Ralstonia solanacearum*
1	1	Proteobacteria	*Ralstonia solanacearum*
1	1	Proteobacteria	*Ralstonia solanacearum GMI1000*
1	1	Proteobacteria	*Ralstonia solanacearum Po82*
1	1	Proteobacteria	*Ralstonia solanacearum PSI07*

1	1	Proteobacteria	*Ramlibacter tataouinensis*
1	1	Proteobacteria	*Ramlibacter tataouinensis TTB310*
1	1	Proteobacteria	*Raoultella ornithinolytica*
1	1	Proteobacteria	*Raoultella ornithinolytica B6*
3	3	Proteobacteria	*Rhizobium etli bv. mimosae str. IE4771*
4	4	Proteobacteria	*Rhizobium etli bv. mimosae str. Mim1*
3	3	Proteobacteria	*Rhizobium etli bv. phaseoli str. IE4803*
4	4	Proteobacteria	*Rhizobium etli CFN 42*
3	3	Proteobacteria	*Rhizobium etli CIAT 652*
3	3	Proteobacteria	*Rhizobium gallicum bv. gallicum R602*
2	2	Proteobacteria	*Rhizobium leguminosarum bv. trifolii CB782*
4	4	Proteobacteria	*Rhizobium leguminosarum bv. trifolii WSM1325*
5	5	Proteobacteria	*Rhizobium leguminosarum bv. trifolii WSM1689*
1	1	Proteobacteria	*Rhizobium leguminosarum bv. trifolii WSM2304*
4	3	Proteobacteria	*Rhizobium leguminosarum bv. viciae 3841*
2	2	Proteobacteria	*Rhizobium sp. IRBG74*
1	1	Proteobacteria	*Rhizobium sp. LPU83*
3	2	Proteobacteria	*Rhizobium sp. NT-26*
1	1	Proteobacteria	*Rhizobium tropici CIAT 899*
1	1	Proteobacteria	*Rhodanobacter denitrificans*
1	1	Proteobacteria	*Rhodobacter capsulatus SB 1003*
3	1	Proteobacteria	*Rhodobacter sphaeroides*
3	1	Proteobacteria	*Rhodobacter sphaeroides 2.4.1*
1	1	Proteobacteria	*Rhodobacter sphaeroides ATCC 17025*
2	1	Proteobacteria	*Rhodobacter sphaeroides ATCC 17029*
3	1	Proteobacteria	*Rhodobacter sphaeroides KD131*
2	1	Proteobacteria	*Rhodobacter sphaeroides WS8N*
2	1	Proteobacteria	*Rhodoferax ferrireducens T118*
1	1	Proteobacteria	*Rhodomicrobium vannielii ATCC 17100*

2	2	Proteobacteria	*Rhodopseudomonas palustris BisA53*
2	2	Proteobacteria	*Rhodopseudomonas palustris BisB18*
2	2	Proteobacteria	*Rhodopseudomonas palustris BisB5*
2	2	Proteobacteria	*Rhodopseudomonas palustris DX-1*
2	2	Proteobacteria	*Rhodopseudomonas palustris HaA2*
2	2	Proteobacteria	*Rhodopseudomonas palustris TIE-1*
1	1	Proteobacteria	*Rhodospirillum centenum SW*
2	2	Proteobacteria	*Rhodospirillum rubrum ATCC 11170*
2	2	Proteobacteria	*Rhodospirillum rubrum F11*
1	1	Proteobacteria	*Rhodovulum sulfidophilum*
1	1	Proteobacteria	*Rickettsia africae ESF-5*
1	1	Proteobacteria	*Rickettsia akari str. Hartford*
1	1	Proteobacteria	*Rickettsia australis str. Cutlack*
1	1	Proteobacteria	*Rickettsia bellii OSU 85-389*
1	1	Proteobacteria	*Rickettsia bellii RML369-C*
1	1	Proteobacteria	*Rickettsia canadensis str. CA410*
1	1	Proteobacteria	*Rickettsia canadensis str. McKiel*
1	1	Proteobacteria	*Rickettsia conorii str. Malish 7*
1	1	Proteobacteria	*Rickettsia felis URRWXCal2*
1	1	Proteobacteria	*Rickettsia heilongjiangensis 054*
1	1	Proteobacteria	*Rickettsia helvetica C9P9*
1	1	Proteobacteria	*Rickettsia japonica YH*
1	1	Proteobacteria	*Rickettsia massiliae MTU5*
1	1	Proteobacteria	*Rickettsia monacensis*
1	1	Proteobacteria	*Rickettsia montanensis str. OSU 85-930*
0	1	Proteobacteria	*Rickettsia parkeri str. Portsmouth*
1	1	Proteobacteria	*Rickettsia philipii str. 364D*
1	1	Proteobacteria	*Rickettsia prowazekii*
1	1	Proteobacteria	*Rickettsia prowazekii str. Breinl*

1	1	Proteobacteria	*Rickettsia prowazekii str. BuV67-CWPP*
1	1	Proteobacteria	*Rickettsia prowazekii str. Chernikova*
1	1	Proteobacteria	*Rickettsia prowazekii str. GvV257*
1	1	Proteobacteria	*Rickettsia prowazekii str. Katsinyian*
1	1	Proteobacteria	*Rickettsia prowazekii str. NMRC Madrid E*
1	1	Proteobacteria	*Rickettsia prowazekii str. Rp22*
1	1	Proteobacteria	*Rickettsia prowazekii str. RpGvF24*
1	1	Proteobacteria	*Rickettsia rhipicephali*
1	1	Proteobacteria	*Rickettsia rhipicephali str. 3-7-female6-CWPP*
1	1	Proteobacteria	*Rickettsia rickettsii str. Arizona*
1	1	Proteobacteria	*Rickettsia rickettsii str. Brazil*
1	1	Proteobacteria	*Rickettsia rickettsii str. Colombia*
1	1	Proteobacteria	*Rickettsia rickettsii str. Hino*
1	1	Proteobacteria	*Rickettsia rickettsii str. Hlp#2*
1	1	Proteobacteria	*Rickettsia rickettsii str. Iowa*
1	1	Proteobacteria	*Rickettsia rickettsii str. Morgan*
1	1	Proteobacteria	*Rickettsia rickettsii str. R*
1	1	Proteobacteria	*Rickettsia rickettsii str. 'Sheila Smith'*
1	1	Proteobacteria	*Rickettsia sibirica 246*
1	1	Proteobacteria	*Rickettsia slovaca 13-B*
1	1	Proteobacteria	*Rickettsia slovaca str. D-CWPP*
1	1	Proteobacteria	*Rickettsia typhi str. B9991CWPP*
1	1	Proteobacteria	*Rickettsia typhi str. TH1527*
1	1	Proteobacteria	*Rickettsia typhi str. Wilmington*
1	1	Proteobacteria	*Rickettsiales bacterium Ac37b*
1	1	Proteobacteria	*Roseateles depolymerans*
1	1	Proteobacteria	*Roseibacterium elongatum DSM 19469*
2	2	Proteobacteria	*Roseobacter denitrificans OCh 114*
1	1	Proteobacteria	*Roseobacter litoralis Och 149*

1	1	Proteobacteria	*Rubrivivax gelatinosus IL144*
1	1	Proteobacteria	*Ruegeria pomeroyi DSS-3*
1	1	Proteobacteria	*Ruegeria sp. TM1040*
1	1	Proteobacteria	*Saccharophagus degradans 2-40*
1	1	Proteobacteria	*Salmonella bongori N268-08*
1	1	Proteobacteria	*Salmonella bongori NCTC 12419*
1	1	Proteobacteria	*Salmonella bongori serovar 48:z41:-- str. RKS3044*
1	1	Proteobacteria	*Salmonella enterica*
1	1	Proteobacteria	*Salmonella enterica subsp. arizonae serovar 62:z36:- str. RKS2983*
1	1	Proteobacteria	*Salmonella enterica subsp. arizonae serovar 62:z4,z23:-*
1	1	Proteobacteria	*Salmonella enterica subsp. enterica*
1	1	Proteobacteria	*Salmonella enterica subsp. enterica serovar 4,[5],12:i:- str. 08-1736*
1	1	Proteobacteria	*Salmonella enterica subsp. enterica serovar Abaetetuba str. ATCC 35640*
1	1	Proteobacteria	*Salmonella enterica subsp. enterica serovar Abony str. 0014*
1	1	Proteobacteria	*Salmonella enterica subsp. enterica serovar Agona str. 24249*
1	1	Proteobacteria	*Salmonella enterica subsp. enterica serovar Agona str. 460004 2-1*
1	1	Proteobacteria	*Salmonella enterica subsp. enterica serovar Agona str. SL483*
1	2	Proteobacteria	*Salmonella enterica subsp. enterica serovar Anatum*
1	2	Proteobacteria	*Salmonella enterica subsp. enterica serovar Anatum*
1	1	Proteobacteria	*Salmonella enterica subsp. enterica serovar Anatum str. ATCC BAA-1592*
1	1	Proteobacteria	*Salmonella enterica subsp. enterica serovar Anatum str. CDC 06-0532*

1	1	Proteobacteria	*Salmonella enterica subsp. enterica serovar Anatum str. USDA-ARS-USMARC-1175*
1	1	Proteobacteria	*Salmonella enterica subsp. enterica serovar Anatum str. USDA-ARS-USMARC-1735*
1	1	Proteobacteria	*Salmonella enterica subsp. enterica serovar Bareilly str. CFSAN000189*
1	1	Proteobacteria	*Salmonella enterica subsp. enterica serovar Bredeney str. CFSAN001080*
1	1	Proteobacteria	*Salmonella enterica subsp. enterica serovar Cerro str. CFSAN001588*
1	1	Proteobacteria	*Salmonella enterica subsp. enterica serovar Choleraesuis*
1	1	Proteobacteria	*Salmonella enterica subsp. enterica serovar Choleraesuis str. ATCC 10708*
1	1	Proteobacteria	*Salmonella enterica subsp. enterica serovar Choleraesuis str. SC-B67*
1	1	Proteobacteria	*Salmonella enterica subsp. enterica serovar Cubana str. CFSAN002050*
1	1	Proteobacteria	*Salmonella enterica subsp. enterica serovar Dublin str. CT_02021853*
1	1	Proteobacteria	*Salmonella enterica subsp. enterica serovar Enteritidis*
1	1	Proteobacteria	*Salmonella enterica subsp. enterica serovar Enteritidis*
1	1	Proteobacteria	*Salmonella enterica subsp. enterica serovar Enteritidis*
1	1	Proteobacteria	*Salmonella enterica subsp. enterica serovar Enteritidis*
1	1	Proteobacteria	*Salmonella enterica subsp. enterica serovar Enteritidis*
1	1	Proteobacteria	*Salmonella enterica subsp. enterica serovar Enteritidis*
1	1	Proteobacteria	*Salmonella enterica subsp. enterica serovar Enteritidis*
1	1	Proteobacteria	*Salmonella enterica subsp. enterica serovar Enteritidis*
1	1	Proteobacteria	*Salmonella enterica subsp. enterica serovar Enteritidis*
1	1	Proteobacteria	*Salmonella enterica subsp. enterica serovar Enteritidis*

1	1	Proteobacteria	*Salmonella enterica subsp. enterica serovar Enteritidis*
1	1	Proteobacteria	*Salmonella enterica subsp. enterica serovar Enteritidis*
1	1	Proteobacteria	*Salmonella enterica subsp. enterica serovar Enteritidis*
1	1	Proteobacteria	*Salmonella enterica subsp. enterica serovar Enteritidis*
1	1	Proteobacteria	*Salmonella enterica subsp. enterica serovar Enteritidis*
1	1	Proteobacteria	*Salmonella enterica subsp. enterica serovar Enteritidis*
1	1	Proteobacteria	*Salmonella enterica subsp. enterica serovar Enteritidis*
1	1	Proteobacteria	*Salmonella enterica subsp. enterica serovar Enteritidis str. 18569*
1	1	Proteobacteria	*Salmonella enterica subsp. enterica serovar Enteritidis str. 77-1427*
1	1	Proteobacteria	*Salmonella enterica subsp. enterica serovar Enteritidis str. CDC_2010K_0968*
1	1	Proteobacteria	*Salmonella enterica subsp. enterica serovar Enteritidis str. EC20090641*
1	1	Proteobacteria	*Salmonella enterica subsp. enterica serovar Enteritidis str. EC20090698*
1	1	Proteobacteria	*Salmonella enterica subsp. enterica serovar Enteritidis str. EC20100101*
1	1	Proteobacteria	*Salmonella enterica subsp. enterica serovar Enteritidis str. EC20100325*
1	1	Proteobacteria	*Salmonella enterica subsp. enterica serovar Enteritidis str. EC20110221*
1	1	Proteobacteria	*Salmonella enterica subsp. enterica serovar Enteritidis str. EC20110353*
1	1	Proteobacteria	*Salmonella enterica subsp. enterica serovar Enteritidis str. EC20110354*
1	1	Proteobacteria	*Salmonella enterica subsp. enterica serovar Enteritidis str. EC20110355*

1	1	Proteobacteria	*Salmonella enterica subsp. enterica serovar Enteritidis str. EC20110356*
1	1	Proteobacteria	*Salmonella enterica subsp. enterica serovar Enteritidis str. EC20110357*
1	1	Proteobacteria	*Salmonella enterica subsp. enterica serovar Enteritidis str. EC20110358*
1	1	Proteobacteria	*Salmonella enterica subsp. enterica serovar Enteritidis str. EC20110359*
1	1	Proteobacteria	*Salmonella enterica subsp. enterica serovar Enteritidis str. EC20110360*
1	1	Proteobacteria	*Salmonella enterica subsp. enterica serovar Enteritidis str. EC20110361*
1	1	Proteobacteria	*Salmonella enterica subsp. enterica serovar Enteritidis str. EC20111095*
1	1	Proteobacteria	*Salmonella enterica subsp. enterica serovar Enteritidis str. EC20111174*
1	1	Proteobacteria	*Salmonella enterica subsp. enterica serovar Enteritidis str. EC20111175*
1	1	Proteobacteria	*Salmonella enterica subsp. enterica serovar Enteritidis str. EC20120002*
1	1	Proteobacteria	*Salmonella enterica subsp. enterica serovar Enteritidis str. EC20120005*
1	1	Proteobacteria	*Salmonella enterica subsp. enterica serovar Enteritidis str. EC20120008*
1	1	Proteobacteria	*Salmonella enterica subsp. enterica serovar Enteritidis str. EC20120200*
1	1	Proteobacteria	*Salmonella enterica subsp. enterica serovar Enteritidis str. EC20120916*
1	1	Proteobacteria	*Salmonella enterica subsp. enterica serovar Enteritidis str.*

			EC20121175
1	1	Proteobacteria	*Salmonella enterica subsp. enterica serovar Enteritidis str. EC20121177*
1	1	Proteobacteria	*Salmonella enterica subsp. enterica serovar Enteritidis str. EC20121178*
1	1	Proteobacteria	*Salmonella enterica subsp. enterica serovar Enteritidis str. EC20121179*
1	1	Proteobacteria	*Salmonella enterica subsp. enterica serovar Enteritidis str. EC20121180*
1	1	Proteobacteria	*Salmonella enterica subsp. enterica serovar Enteritidis str. P125109*
1	1	Proteobacteria	*Salmonella enterica subsp. enterica serovar Enteritidis str. SA19960848*
1	1	Proteobacteria	*Salmonella enterica subsp. enterica serovar Enteritidis str. SA19970510*
1	1	Proteobacteria	*Salmonella enterica subsp. enterica serovar Enteritidis str. SA19970769*
1	1	Proteobacteria	*Salmonella enterica subsp. enterica serovar Enteritidis str. SA19980677*
1	1	Proteobacteria	*Salmonella enterica subsp. enterica serovar Enteritidis str. SA19981522*
1	1	Proteobacteria	*Salmonella enterica subsp. enterica serovar Enteritidis str. SA19981857*
1	1	Proteobacteria	*Salmonella enterica subsp. enterica serovar Enteritidis str. SA19992322*
1	1	Proteobacteria	*Salmonella enterica subsp. enterica serovar Enteritidis str. SA19994216*
1	1	Proteobacteria	*Salmonella enterica subsp. enterica serovar Enteritidis str. SA20082034*

1	1	Proteobacteria	*Salmonella enterica subsp. enterica serovar Enteritidis str. SA20094352*
1	1	Proteobacteria	*Salmonella enterica subsp. enterica serovar Gallinarum str. 287/91*
1	1	Proteobacteria	*Salmonella enterica subsp. enterica serovar Gallinarum/pullorum str. CDC1983-67*
1	1	Proteobacteria	*Salmonella enterica subsp. enterica serovar Gallinarum/pullorum str. RKS5078*
1	1	Proteobacteria	*Salmonella enterica subsp. enterica serovar Heidelberg*
1	1	Proteobacteria	*Salmonella enterica subsp. enterica serovar Heidelberg*
1	1	Proteobacteria	*Salmonella enterica subsp. enterica serovar Heidelberg*
1	1	Proteobacteria	*Salmonella enterica subsp. enterica serovar Heidelberg str. 41578*
1	1	Proteobacteria	*Salmonella enterica subsp. enterica serovar Heidelberg str. B182*
1	1	Proteobacteria	*Salmonella enterica subsp. enterica serovar Heidelberg str. CFSAN002064*
2	2	Proteobacteria	*Salmonella enterica subsp. enterica serovar Heidelberg str. CFSAN002069*
1	1	Proteobacteria	*Salmonella enterica subsp. enterica serovar Heidelberg str. SL476*
1	1	Proteobacteria	*Salmonella enterica subsp. enterica serovar Infantis*
1	1	Proteobacteria	*Salmonella enterica subsp. enterica serovar Javiana str. CFSAN001992*
1	1	Proteobacteria	*Salmonella enterica subsp. enterica serovar Montevideo str. 507440-20*
1	1	Proteobacteria	*Salmonella enterica subsp. enterica serovar Montevideo str. USDA-ARS-USMARC-1903*
1	1	Proteobacteria	*Salmonella enterica subsp. enterica serovar Montevideo str.*

			USDA-ARS-USMARC-1921
1	1	Proteobacteria	*Salmonella enterica subsp. enterica serovar Newport str. CDC 2010K-2159*
1	1	Proteobacteria	*Salmonella enterica subsp. enterica serovar Newport str. CVM 21538*
1	1	Proteobacteria	*Salmonella enterica subsp. enterica serovar Newport str. CVM 21550*
1	1	Proteobacteria	*Salmonella enterica subsp. enterica serovar Newport str. CVM 22425*
1	1	Proteobacteria	*Salmonella enterica subsp. enterica serovar Newport str. CVM 22462*
1	1	Proteobacteria	*Salmonella enterica subsp. enterica serovar Newport str. CVM 22513*
1	1	Proteobacteria	*Salmonella enterica subsp. enterica serovar Newport str. CVM N1543*
1	1	Proteobacteria	*Salmonella enterica subsp. enterica serovar Newport str. CVM N18486*
2	2	Proteobacteria	*Salmonella enterica subsp. enterica serovar Newport str. SL254*
1	1	Proteobacteria	*Salmonella enterica subsp. enterica serovar Newport str. USDA-ARS-USMARC-1927*
1	1	Proteobacteria	*Salmonella enterica subsp. enterica serovar Newport str. USMARC-S3124.1*
1	1	Proteobacteria	*Salmonella enterica subsp. enterica serovar Panama str. ATCC 7378*
1	1	Proteobacteria	*Salmonella enterica subsp. enterica serovar Paratyphi A*
1	1	Proteobacteria	*Salmonella enterica subsp. enterica serovar Paratyphi A*
1	1	Proteobacteria	*Salmonella enterica subsp. enterica serovar Paratyphi A str. AKU_12601*

1	1	Proteobacteria	*Salmonella enterica subsp. enterica serovar Paratyphi A str. ATCC 9150*
1	1	Proteobacteria	*Salmonella enterica subsp. enterica serovar Paratyphi B str. SPB7*
1	1	Proteobacteria	*Salmonella enterica subsp. enterica serovar Paratyphi C str. RKS4594*
1	1	Proteobacteria	*Salmonella enterica subsp. enterica serovar Pullorum str. ATCC 9120*
1	1	Proteobacteria	*Salmonella enterica subsp. enterica serovar Pullorum str. S06004*
1	1	Proteobacteria	*Salmonella enterica subsp. enterica serovar Schwarzengrund str. CVM19633*
1	1	Proteobacteria	*Salmonella enterica subsp. enterica serovar Senftenberg*
1	1	Proteobacteria	*Salmonella enterica subsp. enterica serovar Sloterdijk str. ATCC 15791*
1	1	Proteobacteria	*Salmonella enterica subsp. enterica serovar Tennessee str. TXSC_TXSC08-19*
1	1	Proteobacteria	*Salmonella enterica subsp. enterica serovar Thompson*
1	1	Proteobacteria	*Salmonella enterica subsp. enterica serovar Thompson*
1	1	Proteobacteria	*Salmonella enterica subsp. enterica serovar Thompson str. ATCC 8391*
1	1	Proteobacteria	*Salmonella enterica subsp. enterica serovar Thompson str. RM6836*
1	1	Proteobacteria	*Salmonella enterica subsp. enterica serovar Typhi*
1	1	Proteobacteria	*Salmonella enterica subsp. enterica serovar Typhi*
1	1	Proteobacteria	*Salmonella enterica subsp. enterica serovar Typhi str. CT18*
1	1	Proteobacteria	*Salmonella enterica subsp. enterica serovar Typhi str. P-stx-12*
1	1	Proteobacteria	*Salmonella enterica subsp. enterica serovar Typhi str. Ty2*

1	1	Proteobacteria	*Salmonella enterica subsp. enterica serovar Typhi str. Ty21a*
1	1	Proteobacteria	*Salmonella enterica subsp. enterica serovar Typhimurium*
1	1	Proteobacteria	*Salmonella enterica subsp. enterica serovar Typhimurium*
1	1	Proteobacteria	*Salmonella enterica subsp. enterica serovar Typhimurium*
1	1	Proteobacteria	*Salmonella enterica subsp. enterica serovar Typhimurium*
1	1	Proteobacteria	*Salmonella enterica subsp. enterica serovar Typhimurium*
1	1	Proteobacteria	*Salmonella enterica subsp. enterica serovar Typhimurium*
1	1	Proteobacteria	*Salmonella enterica subsp. enterica serovar Typhimurium*
1	1	Proteobacteria	*Salmonella enterica subsp. enterica serovar Typhimurium*
1	1	Proteobacteria	*Salmonella enterica subsp. enterica serovar Typhimurium*
1	1	Proteobacteria	*Salmonella enterica subsp. enterica serovar Typhimurium*
1	1	Proteobacteria	*Salmonella enterica subsp. enterica serovar Typhimurium str. 14028S*
1	1	Proteobacteria	*Salmonella enterica subsp. enterica serovar Typhimurium str. 798*
1	1	Proteobacteria	*Salmonella enterica subsp. enterica serovar Typhimurium str. CDC 2011K-0870*
1	1	Proteobacteria	*Salmonella enterica subsp. enterica serovar Typhimurium str. D23580*
1	1	Proteobacteria	*Salmonella enterica subsp. enterica serovar Typhimurium str. DT104*
1	1	Proteobacteria	*Salmonella enterica subsp. enterica serovar Typhimurium str. DT2*
1	1	Proteobacteria	*Salmonella enterica subsp. enterica serovar Typhimurium str. L-3553*
1	1	Proteobacteria	*Salmonella enterica subsp. enterica serovar Typhimurium str. LT2*
1	1	Proteobacteria	*Salmonella enterica subsp. enterica serovar Typhimurium*

			str. SL1344
1	1	Proteobacteria	*Salmonella enterica subsp. enterica serovar Typhimurium str. ST4/74*
1	1	Proteobacteria	*Salmonella enterica subsp. enterica serovar Typhimurium str. T000240*
1	1	Proteobacteria	*Salmonella enterica subsp. enterica serovar Typhimurium str. U288*
1	1	Proteobacteria	*Salmonella enterica subsp. enterica serovar Typhimurium str. UK-1*
1	1	Proteobacteria	*Salmonella enterica subsp. enterica serovar Typhimurium str. USDA-ARS-USMARC-1899*
1	1	Proteobacteria	*Salmonella enterica subsp. enterica serovar Typhimurium var. 5- str. CFSAN001921*
1	1	Proteobacteria	*Salmonella enterica subsp. enterica serovar Weltevreden*
1	1	Proteobacteria	*Salmonella enterica subsp. enterica serovar Weltevreden*
1	1	Proteobacteria	*Salmonella enterica subsp. enterica serovar Weltevreden*
1	1	Proteobacteria	*Salmonella enterica subsp. enterica serovar Weltevreden*
1	1	Proteobacteria	*Sandaracinus amylolyticus*
1	1	Proteobacteria	*secondary endosymbiont of Ctenarytaina eucalypti*
1	1	Proteobacteria	*secondary endosymbiont of Heteropsylla cubana*
1	1	Proteobacteria	*Sedimenticola sp. SIP-G1*
1	1	Proteobacteria	*Serratia fonticola*
1	1	Proteobacteria	*Serratia fonticola*
1	1	Proteobacteria	*Serratia liquefaciens*
1	1	Proteobacteria	*Serratia liquefaciens*
1	1	Proteobacteria	*Serratia liquefaciens ATCC 27592*
1	1	Proteobacteria	*Serratia marcescens*
1	1	Proteobacteria	*Serratia marcescens*
1	1	Proteobacteria	*Serratia marcescens*

1	1	Proteobacteria	*Serratia marcescens*
1	1	Proteobacteria	*Serratia marcescens FGI94*
1	1	Proteobacteria	*Serratia marcescens SM39*
1	1	Proteobacteria	*Serratia marcescens subsp. marcescens Db11*
1	1	Proteobacteria	*Serratia marcescens WW4*
1	1	Proteobacteria	*Serratia plymuthica 4Rx13*
1	1	Proteobacteria	*Serratia plymuthica AS9*
1	1	Proteobacteria	*Serratia plymuthica RVH1*
1	1	Proteobacteria	*Serratia plymuthica S13*
1	1	Proteobacteria	*Serratia proteamaculans 568*
1	1	Proteobacteria	*Serratia rubidaea*
1	1	Proteobacteria	*Serratia sp. AS12*
1	1	Proteobacteria	*Serratia sp. AS13*
1	1	Proteobacteria	*Serratia sp. FS14*
1	1	Proteobacteria	*Serratia sp. SCBI*
1	1	Proteobacteria	*Shewanella amazonensis SB2B*
1	1	Proteobacteria	*Shewanella baltica BA175*
1	1	Proteobacteria	*Shewanella baltica OS117*
1	1	Proteobacteria	*Shewanella baltica OS155*
1	1	Proteobacteria	*Shewanella baltica OS185*
1	1	Proteobacteria	*Shewanella baltica OS195*
1	1	Proteobacteria	*Shewanella baltica OS223*
1	1	Proteobacteria	*Shewanella baltica OS678*
1	1	Proteobacteria	*Shewanella denitrificans OS217*
1	1	Proteobacteria	*Shewanella frigidimarina NCIMB 400*
1	1	Proteobacteria	*Shewanella halifaxensis HAW-EB4*
1	1	Proteobacteria	*Shewanella loihica PV-4*
1	1	Proteobacteria	*Shewanella oneidensis MR-1*
1	1	Proteobacteria	*Shewanella pealeana ATCC 700345*

1	1	Proteobacteria	*Shewanella piezotolerans WP3*	
1	1	Proteobacteria	*Shewanella putrefaciens 200*	
1	1	Proteobacteria	*Shewanella putrefaciens CN-32*	
1	1	Proteobacteria	*Shewanella sediminis HAW-EB3*	
1	1	Proteobacteria	*Shewanella sp. ANA-3*	
1	1	Proteobacteria	*Shewanella sp. MR-4*	
1	1	Proteobacteria	*Shewanella sp. MR-7*	
1	1	Proteobacteria	*Shewanella sp. W3-18-1*	
1	1	Proteobacteria	*Shewanella violacea DSS12*	
1	1	Proteobacteria	*Shewanella woodyi ATCC 51908*	
1	1	Proteobacteria	*Shigella boydii*	
1	1	Proteobacteria	*Shigella boydii CDC 3083-94*	
1	1	Proteobacteria	*Shigella boydii Sb227*	
1	1	Proteobacteria	*Shigella dysenteriae Sd197*	
1	1	Proteobacteria	*Shigella flexneri*	
1	1	Proteobacteria	*Shigella flexneri 1a*	
1	1	Proteobacteria	*Shigella flexneri 2002017*	
1	1	Proteobacteria	*Shigella flexneri 2003036*	
1	1	Proteobacteria	*Shigella flexneri 2a*	
1	1	Proteobacteria	*Shigella flexneri 2a str. 2457T*	
1	1	Proteobacteria	*Shigella flexneri 2a str. 301*	
1	1	Proteobacteria	*Shigella flexneri 4c*	
1	1	Proteobacteria	*Shigella flexneri 5 str. 8401*	
1	1	Proteobacteria	*Shigella flexneri G1663*	
1	1	Proteobacteria	*Shigella flexneri Shi06HN006*	
1	1	Proteobacteria	*Shigella sonnei*	
1	1	Proteobacteria	*Shigella sonnei*	
1	1	Proteobacteria	*Shigella sonnei 53G*	
1	1	Proteobacteria	*Shigella sonnei Ss046*	

1	1	Proteobacteria	*Shigella sp. PAMC 28760*
1	1	Proteobacteria	*Shimwellia blattae DSM 4481 = NBRC 105725*
1	1	Proteobacteria	*Sideroxydans lithotrophicus ES-1*
2	2	Proteobacteria	*Simiduia agarivorans SA1 = DSM 21679*
4	3	Proteobacteria	*Sinorhizobium fredii NGR234*
5	3	Proteobacteria	*Sinorhizobium medicae WSM419*
6	4	Proteobacteria	*Sinorhizobium meliloti*
5	4	Proteobacteria	*Sinorhizobium meliloti 1021*
5	4	Proteobacteria	*Sinorhizobium meliloti 2011*
7	6	Proteobacteria	*Sinorhizobium meliloti AK83*
6	4	Proteobacteria	*Sinorhizobium meliloti BL225C*
6	5	Proteobacteria	*Sinorhizobium meliloti GR4*
6	3	Proteobacteria	*Sinorhizobium meliloti Rm41*
7	5	Proteobacteria	*Sinorhizobium meliloti SM11*
1	1	Proteobacteria	*Snodgrassella alvi wkB2*
1	1	Proteobacteria	*Sodalis glossinidius str. 'morsitans'*
1	1	Proteobacteria	*Sodalis praecaptivus*
2	2	Proteobacteria	*Sorangium cellulosum So ce56*
3	2	Proteobacteria	*Sorangium cellulosum So0157-2*
3	3	Proteobacteria	*Sphingobium baderi*
1	1	Proteobacteria	*Sphingobium chlorophenolicum L-1*
1	1	Proteobacteria	*Sphingobium japonicum UT26S*
2	2	Proteobacteria	*Sphingobium sp. SYK-6*
2	2	Proteobacteria	*Sphingobium sp. YBL2*
1	1	Proteobacteria	*Sphingomonas hengshuiensis*
3	3	Proteobacteria	*Sphingomonas sanxanigenens DSM 19645 = NX02*
1	1	Proteobacteria	*Sphingomonas sp. MM-1*
1	1	Proteobacteria	*Sphingomonas taxi*
1	1	Proteobacteria	*Sphingomonas wittichii RW1*

2	2	Proteobacteria	*Sphingopyxis alaskensis RB2256*
1	1	Proteobacteria	*Sphingopyxis fribergensis*
1	1	Proteobacteria	*Sphingopyxis macrogoltabida*
2	2	Proteobacteria	*Sphingopyxis macrogoltabida*
2	2	Proteobacteria	*Sphingopyxis sp. 113P3*
1	1	Proteobacteria	*Spiribacter salinus M19-40*
1	1	Proteobacteria	*Spiribacter sp. UAH-SP71*
1	1	Proteobacteria	*Spongiibacter sp. IMCC21906*
3	3	Proteobacteria	*Starkeya novella DSM 506*
1	1	Proteobacteria	*Stenotrophomonas acidaminiphila*
1	1	Proteobacteria	*Stenotrophomonas maltophilia*
1	1	Proteobacteria	*Stenotrophomonas maltophilia*
1	1	Proteobacteria	*Stenotrophomonas maltophilia*
1	1	Proteobacteria	*Stenotrophomonas maltophilia D457*
1	1	Proteobacteria	*Stenotrophomonas maltophilia JV3*
1	1	Proteobacteria	*Stenotrophomonas maltophilia K279a*
1	1	Proteobacteria	*Stenotrophomonas maltophilia R551-3*
2	1	Proteobacteria	*Stigmatella aurantiaca DW4/3-1*
1	1	Proteobacteria	*Sulfuricella denitrificans skB26*
1	1	Proteobacteria	*Sulfuricurvum kujiense DSM 16994*
1	1	Proteobacteria	*Sulfurimonas autotrophica DSM 16294*
1	1	Proteobacteria	*Sulfurimonas denitrificans DSM 1251*
1	1	Proteobacteria	*Sulfurimonas gotlandica GD1*
1	1	Proteobacteria	*Sulfuritalea hydrogenivorans sk43H*
1	1	Proteobacteria	*Sulfurospirillum barnesii SES-3*
1	1	Proteobacteria	*Sulfurospirillum cavolei*
1	1	Proteobacteria	*Sulfurospirillum deleyianum DSM 6946*
1	1	Proteobacteria	*Sulfurospirillum multivorans DSM 12446*
1	1	Proteobacteria	*Sulfurovum lithotrophicum*

1	1	Proteobacteria	*Sulfurovum sp. NBC37-1*
2	2	Proteobacteria	*Syntrophobacter fumaroxidans MPOB*
3	3	Proteobacteria	*Syntrophus aciditrophicus SB*
1	1	Proteobacteria	*Tatlockia micdadei*
1	1	Proteobacteria	*Taylorella asinigenitalis MCE3*
1	1	Proteobacteria	*Taylorella equigenitalis ATCC 35865*
1	1	Proteobacteria	*Taylorella equigenitalis MCE9*
1	1	Proteobacteria	*Teredinibacter turnerae T7901*
1	1	Proteobacteria	*Thalassolituus oleivorans MIL-1*
1	1	Proteobacteria	*Thalassolituus oleivorans R6-15*
1	1	Proteobacteria	*Thalassospira xiamenensis M-5 = DSM 17429*
1	1	Proteobacteria	*Thauera humireducens*
2	1	Proteobacteria	*Thauera sp. MZ1T*
1	1	Proteobacteria	*Thioalkalimicrobium aerophilum AL3*
1	1	Proteobacteria	*Thioalkalimicrobium cyclicum ALM1*
1	1	Proteobacteria	*Thioalkalivibrio nitratireducens DSM 14787*
1	1	Proteobacteria	*Thioalkalivibrio paradoxus ARh 1*
1	1	Proteobacteria	*Thioalkalivibrio sp. K90mix*
1	1	Proteobacteria	*Thioalkalivibrio sulfidiphilus HL-EbGr7*
1	1	Proteobacteria	*Thioalkalivibrio versutus*
1	1	Proteobacteria	*Thiobacillus denitrificans ATCC 25259*
2	2	Proteobacteria	*Thiocystis violascens DSM 198*
1	1	Proteobacteria	*Thioflavicoccus mobilis 8321*
1	1	Proteobacteria	*Thiolapillus brandeum*
1	1	Proteobacteria	*Thiomicrospira crunogena XCL-2*
1	1	Proteobacteria	*Thiomonas intermedia K12*
1	1	Proteobacteria	*Thioploca ingrica*
1	1	Proteobacteria	*Tistrella mobilis KA081020-065*
1	1	Proteobacteria	*Tolumonas auensis DSM 9187*

2	1	Proteobacteria	*Variovorax paradoxus B4*
1	1	Proteobacteria	*Variovorax paradoxus EPS*
1	1	Proteobacteria	*Variovorax paradoxus S110*
2	1	Proteobacteria	*Verminephrobacter eiseniae EF01-2*
2	2	Proteobacteria	*Vibrio alginolyticus NBRC 15630 = ATCC 17749*
1	1	Proteobacteria	*Vibrio anguillarum*
1	1	Proteobacteria	*Vibrio anguillarum 775*
2	2	Proteobacteria	*Vibrio antiquarius*
1	1	Proteobacteria	*Vibrio campbellii ATCC BAA-1116*
1	1	Proteobacteria	*Vibrio campbellii ATCC BAA-1116*
2	2	Proteobacteria	*Vibrio cholerae*
2	2	Proteobacteria	*Vibrio cholerae*
2	2	Proteobacteria	*Vibrio cholerae*
2	2	Proteobacteria	*Vibrio cholerae*
2	2	Proteobacteria	*Vibrio cholerae H1*
2	2	Proteobacteria	*Vibrio cholerae IEC224*
2	2	Proteobacteria	*Vibrio cholerae LMA3984-4*
2	2	Proteobacteria	*Vibrio cholerae M66-2*
2	2	Proteobacteria	*Vibrio cholerae MJ-1236*
2	2	Proteobacteria	*Vibrio cholerae MS6*
2	2	Proteobacteria	*Vibrio cholerae O1 biovar El Tor*
2	2	Proteobacteria	*Vibrio cholerae O1 biovar El Tor str. N16961*
2	2	Proteobacteria	*Vibrio cholerae O1 str. 2010EL-1786*
2	2	Proteobacteria	*Vibrio cholerae O1 str. KW3*
2	2	Proteobacteria	*Vibrio cholerae O395*
2	2	Proteobacteria	*Vibrio cholerae O395*
1	1	Proteobacteria	*Vibrio coralliilyticus*
1	1	Proteobacteria	*Vibrio coralliilyticus*
1	1	Proteobacteria	*Vibrio fischeri ES114*

2	2	Proteobacteria	*Vibrio fluvialis*
2	2	Proteobacteria	*Vibrio furnissii NCTC 11218*
1	1	Proteobacteria	*Vibrio harveyi*
1	1	Proteobacteria	*Vibrio mimicus*
1	1	Proteobacteria	*Vibrio nigripulchritudo*
2	2	Proteobacteria	*Vibrio parahaemolyticus*
2	2	Proteobacteria	*Vibrio parahaemolyticus*
2	2	Proteobacteria	*Vibrio parahaemolyticus*
2	2	Proteobacteria	*Vibrio parahaemolyticus*
2	2	Proteobacteria	*Vibrio parahaemolyticus BB22OP*
2	2	Proteobacteria	*Vibrio parahaemolyticus O1:K33 str. CDC_K4557*
2	2	Proteobacteria	*Vibrio parahaemolyticus O1:Kuk str. FDA_R31*
2	2	Proteobacteria	*Vibrio parahaemolyticus RIMD 2210633*
2	2	Proteobacteria	*Vibrio parahaemolyticus UCM-V493*
2	2	Proteobacteria	*Vibrio sp. EJY3*
1	1	Proteobacteria	*Vibrio tasmaniensis LGP32*
1	1	Proteobacteria	*Vibrio tubiashii ATCC 19109*
2	2	Proteobacteria	*Vibrio vulnificus*
2	2	Proteobacteria	*Vibrio vulnificus*
2	2	Proteobacteria	*Vibrio vulnificus*
2	3	Proteobacteria	*Vibrio vulnificus CMCP6*
2	2	Proteobacteria	*Vibrio vulnificus MO6-24/O*
2	2	Proteobacteria	*Vibrio vulnificus YJ016*
1	1	Proteobacteria	*Vulgatibacter incomptus*
1	1	Proteobacteria	*Wenzhouxiangella marina*
1	1	Proteobacteria	*Wigglesworthia glossinidia endosymbiont of Glossina brevipalpis*
1	1	Proteobacteria	*Wigglesworthia glossinidia endosymbiont of Glossina morsitans morsitans (Yale colony)*

1	1	Proteobacteria	*Wolbachia endosymbiont of Cimex lectularius*
1	1	Proteobacteria	*Wolbachia endosymbiont of Drosophila melanogaster*
1	1	Proteobacteria	*Wolbachia endosymbiont of Drosophila simulans wHa*
1	1	Proteobacteria	*Wolbachia endosymbiont of Drosophila simulans wNo*
1	1	Proteobacteria	*Wolbachia endosymbiont of Onchocerca ochengi*
1	1	Proteobacteria	*Wolbachia endosymbiont of Onchocerca volvulus str. Cameroon*
1	1	Proteobacteria	*Wolbachia endosymbiont strain TRS of Brugia malayi*
1	1	Proteobacteria	*Wolbachia sp. wRi*
2	2	Proteobacteria	*Xanthobacter autotrophicus Py2*
1	1	Proteobacteria	*Xanthomonas albilineans GPE PC73*
1	1	Proteobacteria	*Xanthomonas axonopodis pv. citri str. 306*
1	1	Proteobacteria	*Xanthomonas axonopodis pv. citrumelo F1*
1	1	Proteobacteria	*Xanthomonas axonopodis Xac29-1*
1	1	Proteobacteria	*Xanthomonas campestris*
1	1	Proteobacteria	*Xanthomonas campestris pv. campestris*
1	1	Proteobacteria	*Xanthomonas campestris pv. campestris*
1	1	Proteobacteria	*Xanthomonas campestris pv. campestris*
1	1	Proteobacteria	*Xanthomonas campestris pv. campestris str. 8004*
1	1	Proteobacteria	*Xanthomonas campestris pv. campestris str. ATCC 33913*
1	1	Proteobacteria	*Xanthomonas campestris pv. raphani 756C*
1	1	Proteobacteria	*Xanthomonas campestris pv. vesicatoria str. 85-10*
1	1	Proteobacteria	*Xanthomonas citri pv. citri*
1	1	Proteobacteria	*Xanthomonas citri subsp. citri*
1	1	Proteobacteria	*Xanthomonas citri subsp. citri*
1	1	Proteobacteria	*Xanthomonas citri subsp. citri*
1	1	Proteobacteria	*Xanthomonas citri subsp. citri*
1	1	Proteobacteria	*Xanthomonas citri subsp. citri*
1	1	Proteobacteria	*Xanthomonas citri subsp. citri*

1	1	Proteobacteria	*Xanthomonas citri subsp. citri*
1	1	Proteobacteria	*Xanthomonas citri subsp. citri*
1	1	Proteobacteria	*Xanthomonas citri subsp. citri*
1	1	Proteobacteria	*Xanthomonas citri subsp. citri*
1	1	Proteobacteria	*Xanthomonas citri subsp. citri*
1	1	Proteobacteria	*Xanthomonas citri subsp. citri*
1	1	Proteobacteria	*Xanthomonas citri subsp. citri*
1	1	Proteobacteria	*Xanthomonas citri subsp. citri*
1	1	Proteobacteria	*Xanthomonas citri subsp. citri*
1	1	Proteobacteria	*Xanthomonas citri subsp. citri*
1	1	Proteobacteria	*Xanthomonas citri subsp. citri*
1	1	Proteobacteria	*Xanthomonas citri subsp. citri A306*
1	1	Proteobacteria	*Xanthomonas citri subsp. citri Aw12879*
1	1	Proteobacteria	*Xanthomonas citri subsp. citri UI6*
1	1	Proteobacteria	*Xanthomonas fuscans subsp. fuscans*
1	1	Proteobacteria	*Xanthomonas oryzae pv. oryzae*
1	1	Proteobacteria	*Xanthomonas oryzae pv. oryzae*
1	1	Proteobacteria	*Xanthomonas oryzae pv. oryzae KACC 10331*
1	1	Proteobacteria	*Xanthomonas oryzae pv. oryzae MAFF 311018*
1	1	Proteobacteria	*Xanthomonas oryzae pv. oryzae PXO86*
1	1	Proteobacteria	*Xanthomonas oryzae pv. oryzae PXO99A*
1	1	Proteobacteria	*Xanthomonas oryzae pv. oryzicola*
1	1	Proteobacteria	*Xanthomonas oryzae pv. oryzicola*
1	1	Proteobacteria	*Xanthomonas oryzae pv. oryzicola*
1	1	Proteobacteria	*Xanthomonas oryzae pv. oryzicola*
1	1	Proteobacteria	*Xanthomonas oryzae pv. oryzicola*
1	1	Proteobacteria	*Xanthomonas oryzae pv. oryzicola*
1	1	Proteobacteria	*Xanthomonas oryzae pv. oryzicola*
1	1	Proteobacteria	*Xanthomonas oryzae pv. oryzicola*

1	1	Proteobacteria	*Xanthomonas oryzae pv. oryzicola*
1	1	Proteobacteria	*Xanthomonas oryzae pv. oryzicola*
1	1	Proteobacteria	*Xanthomonas oryzae pv. oryzicola BLS256*
1	1	Proteobacteria	*Xanthomonas sacchari*
1	1	Proteobacteria	*Xanthomonas translucens pv. undulosa*
1	1	Proteobacteria	*Xenorhabdus bovienii*
1	1	Proteobacteria	*Xenorhabdus bovienii SS-2004*
1	1	Proteobacteria	*Xenorhabdus doucetiae*
1	1	Proteobacteria	*Xenorhabdus nematophila AN6/1*
1	1	Proteobacteria	*Xenorhabdus poinarii G6*
1	1	Proteobacteria	*Xylella fastidiosa 9a5c*
1	1	Proteobacteria	*Xylella fastidiosa M12*
1	1	Proteobacteria	*Xylella fastidiosa M23*
1	1	Proteobacteria	*Xylella fastidiosa MUL0034*
1	1	Proteobacteria	*Xylella fastidiosa subsp. fastidiosa GB514*
1	1	Proteobacteria	*Xylella fastidiosa subsp. sandyi Ann-1*
1	1	Proteobacteria	*Xylella fastidiosa Temecula1*
1	1	Proteobacteria	*Yersinia aldovae 670-83*
1	1	Proteobacteria	*Yersinia aleksiciae*
1	1	Proteobacteria	*Yersinia enterocolitica*
1	1	Proteobacteria	*Yersinia enterocolitica*
1	1	Proteobacteria	*Yersinia enterocolitica*
1	1	Proteobacteria	*Yersinia enterocolitica*
1	1	Proteobacteria	*Yersinia enterocolitica*
1	1	Proteobacteria	*Yersinia enterocolitica (type O:5) str. YE53/03*
1	1	Proteobacteria	*Yersinia enterocolitica subsp. enterocolitica 8081*
1	1	Proteobacteria	*Yersinia enterocolitica subsp. palearctica 105.5R(r)*
1	1	Proteobacteria	*Yersinia enterocolitica subsp. palearctica Y11*
1	1	Proteobacteria	*Yersinia frederiksenii Y225*

1	1	Proteobacteria	*Yersinia intermedia*	
1	1	Proteobacteria	*Yersinia kristensenii*	
1	1	Proteobacteria	*Yersinia pestis*	
1	1	Proteobacteria	*Yersinia pestis*	
1	1	Proteobacteria	*Yersinia pestis*	
1	1	Proteobacteria	*Yersinia pestis*	
1	1	Proteobacteria	*Yersinia pestis*	
1	1	Proteobacteria	*Yersinia pestis*	
1	1	Proteobacteria	*Yersinia pestis 1412*	
1	1	Proteobacteria	*Yersinia pestis 1413*	
1	1	Proteobacteria	*Yersinia pestis 1522*	
1	1	Proteobacteria	*Yersinia pestis 2944*	
1	1	Proteobacteria	*Yersinia pestis 3067*	
1	1	Proteobacteria	*Yersinia pestis 3770*	
1	1	Proteobacteria	*Yersinia pestis 8787*	
1	1	Proteobacteria	*Yersinia pestis A1122*	
1	1	Proteobacteria	*Yersinia pestis A1122*	
1	1	Proteobacteria	*Yersinia pestis Angola*	
1	1	Proteobacteria	*Yersinia pestis Angola*	
1	1	Proteobacteria	*Yersinia pestis Antiqua*	
1	1	Proteobacteria	*Yersinia pestis Antiqua*	
1	1	Proteobacteria	*Yersinia pestis biovar Medievalis str. Harbin 35*	
1	1	Proteobacteria	*Yersinia pestis biovar Microtus str. 91001*	
1	1	Proteobacteria	*Yersinia pestis CO92*	
1	1	Proteobacteria	*Yersinia pestis CO92*	
1	1	Proteobacteria	*Yersinia pestis D106004*	
1	1	Proteobacteria	*Yersinia pestis D182038*	
1	1	Proteobacteria	*Yersinia pestis KIM10+*	
1	1	Proteobacteria	*Yersinia pestis Nepal516*	

1	1	Proteobacteria	*Yersinia pestis Pestoides F*
1	1	Proteobacteria	*Yersinia pestis Pestoides F*
1	1	Proteobacteria	*Yersinia pestis Pestoides G*
1	1	Proteobacteria	*Yersinia pestis str. Pestoides B*
1	1	Proteobacteria	*Yersinia pestis Z176003*
1	1	Proteobacteria	*Yersinia pseudotuberculosis*
1	1	Proteobacteria	*Yersinia pseudotuberculosis*
1	1	Proteobacteria	*Yersinia pseudotuberculosis*
1	1	Proteobacteria	*Yersinia pseudotuberculosis*
1	1	Proteobacteria	*Yersinia pseudotuberculosis IP 31758*
1	1	Proteobacteria	*Yersinia pseudotuberculosis IP 32953*
1	1	Proteobacteria	*Yersinia pseudotuberculosis IP 32953*
1	1	Proteobacteria	*Yersinia pseudotuberculosis PB1/+*
1	1	Proteobacteria	*Yersinia pseudotuberculosis PB1/+*
1	1	Proteobacteria	*Yersinia pseudotuberculosis str. PA3606*
1	1	Proteobacteria	*Yersinia pseudotuberculosis YPIII*
1	1	Proteobacteria	*Yersinia pseudotuberculosis YPIII*
1	1	Proteobacteria	*Yersinia rohdei*
1	1	Proteobacteria	*Yersinia ruckeri*
1	1	Proteobacteria	*Yersinia ruckeri*
1	1	Proteobacteria	*Yersinia similis*
2	2	Proteobacteria	*Zhongshania aliphaticivorans*
1	1	Proteobacteria	*Zymomonas mobilis subsp. mobilis ATCC 10988*
1	1	Proteobacteria	*Zymomonas mobilis subsp. mobilis ATCC 29191*
1	1	Proteobacteria	*Zymomonas mobilis subsp. mobilis NCIMB 11163*
1	1	Proteobacteria	*Zymomonas mobilis subsp. mobilis NRRL B-12526*
1	1	Proteobacteria	*Zymomonas mobilis subsp. mobilis str. CP4 = NRRL B-14023*
1	1	Proteobacteria	*Zymomonas mobilis subsp. mobilis str. CP4 = NRRL*

			B-14023
1	1	Proteobacteria	*Zymomonas mobilis subsp. mobilis ZM4 = ATCC 31821*
1	1	Proteobacteria	*Zymomonas mobilis subsp. pomaceae ATCC 29192*
1	1	PVC group	*Akkermansia muciniphila ATCC BAA-835*
1	1	PVC group	*Candidatus Xiphinematobacter sp. Idaho Grape*
2	1	PVC group	*Chlamydia abortus*
2	1	PVC group	*Chlamydia abortus S26/3*
1	1	PVC group	*Chlamydia avium 10DC88*
2	1	PVC group	*Chlamydia felis Fe/C-56*
1	1	PVC group	*Chlamydia muridarum*
1	1	PVC group	*Chlamydia muridarum*
1	1	PVC group	*Chlamydia muridarum*
1	1	PVC group	*Chlamydia muridarum str. Nigg*
1	1	PVC group	*Chlamydia muridarum str. Nigg 2 MCR*
1	1	PVC group	*Chlamydia muridarum str. Nigg CM972*
1	1	PVC group	*Chlamydia muridarum str. Nigg3 CMUT3-5*
1	1	PVC group	*Chlamydia pecorum P787*
1	1	PVC group	*Chlamydia pecorum PV3056/3*
1	1	PVC group	*Chlamydia pecorum W73*
2	1	PVC group	*Chlamydia pneumoniae*
2	1	PVC group	*Chlamydia pneumoniae*
2	1	PVC group	*Chlamydia pneumoniae*
2	1	PVC group	*Chlamydia pneumoniae*
2	1	PVC group	*Chlamydia pneumoniae*
2	1	PVC group	*Chlamydia pneumoniae*
2	1	PVC group	*Chlamydia psittaci 01DC11*
2	1	PVC group	*Chlamydia psittaci 02DC15*
2	1	PVC group	*Chlamydia psittaci 08DC60*
2	1	PVC group	*Chlamydia psittaci 6BC*

2	1	PVC group	*Chlamydia psittaci 6BC*
1	1	PVC group	*Chlamydia psittaci 84/55*
2	1	PVC group	*Chlamydia psittaci C19/98*
2	1	PVC group	*Chlamydia psittaci CP3*
2	1	PVC group	*Chlamydia psittaci GR9*
2	1	PVC group	*Chlamydia psittaci M56*
2	1	PVC group	*Chlamydia psittaci MN*
2	1	PVC group	*Chlamydia psittaci NJ1*
2	1	PVC group	*Chlamydia psittaci VS225*
2	1	PVC group	*Chlamydia psittaci WC*
2	1	PVC group	*Chlamydia psittaci WS/RT/E30*
1	1	PVC group	*Chlamydia trachomatis*
1	1	PVC group	*Chlamydia trachomatis*
1	1	PVC group	*Chlamydia trachomatis*
1	1	PVC group	*Chlamydia trachomatis*
1	1	PVC group	*Chlamydia trachomatis*
1	1	PVC group	*Chlamydia trachomatis*
1	1	PVC group	*Chlamydia trachomatis*
1	1	PVC group	*Chlamydia trachomatis*
1	1	PVC group	*Chlamydia trachomatis*
1	1	PVC group	*Chlamydia trachomatis 434/Bu*
1	1	PVC group	*Chlamydia trachomatis 6276*
1	1	PVC group	*Chlamydia trachomatis 6276s*
1	1	PVC group	*Chlamydia trachomatis 70*
1	1	PVC group	*Chlamydia trachomatis 70s*
1	1	PVC group	*Chlamydia trachomatis A/363*
1	1	PVC group	*Chlamydia trachomatis A/7249*
1	1	PVC group	*Chlamydia trachomatis A/HAR-13*
1	1	PVC group	*Chlamydia trachomatis A2497*

1	1	PVC group	*Chlamydia trachomatis A2497*
1	1	PVC group	*Chlamydia trachomatis B/Jali20/OT*
1	1	PVC group	*Chlamydia trachomatis B/TZ1A828/OT*
1	1	PVC group	*Chlamydia trachomatis C/TW-3*
1	1	PVC group	*Chlamydia trachomatis D(s)2923*
1	1	PVC group	*Chlamydia trachomatis D/CS637/11*
1	1	PVC group	*Chlamydia trachomatis D/UW-3/CX*
1	1	PVC group	*Chlamydia trachomatis D-EC*
1	1	PVC group	*Chlamydia trachomatis D-LC*
1	1	PVC group	*Chlamydia trachomatis E/11023*
1	1	PVC group	*Chlamydia trachomatis E/150*
1	1	PVC group	*Chlamydia trachomatis E/Bour*
1	1	PVC group	*Chlamydia trachomatis E/SW3*
1	1	PVC group	*Chlamydia trachomatis F/11-96*
1	1	PVC group	*Chlamydia trachomatis F/SW4*
1	1	PVC group	*Chlamydia trachomatis F/SW5*
1	1	PVC group	*Chlamydia trachomatis G/11074*
1	1	PVC group	*Chlamydia trachomatis G/11222*
1	1	PVC group	*Chlamydia trachomatis G/9301*
1	1	PVC group	*Chlamydia trachomatis G/9768*
1	1	PVC group	*Chlamydia trachomatis Ia/SotonIa1*
1	1	PVC group	*Chlamydia trachomatis Ia/SotonIa3*
1	1	PVC group	*Chlamydia trachomatis J/6276tet1*
1	1	PVC group	*Chlamydia trachomatis L1/115*
1	1	PVC group	*Chlamydia trachomatis L1/224*
1	1	PVC group	*Chlamydia trachomatis L1/440/LN*
1	1	PVC group	*Chlamydia trachomatis L2/25667R*
1	1	PVC group	*Chlamydia trachomatis L2/434/Bu(f)*
1	1	PVC group	*Chlamydia trachomatis L2/434/Bu(i)*

1	1	PVC group	*Chlamydia trachomatis L2b/795*
1	1	PVC group	*Chlamydia trachomatis L2b/8200/07*
1	1	PVC group	*Chlamydia trachomatis L2b/UCH-1/proctitis*
1	1	PVC group	*Chlamydia trachomatis L2b/UCH-2*
1	1	PVC group	*Chlamydia trachomatis L2c*
1	1	PVC group	*Chlamydia trachomatis L2tet1*
1	1	PVC group	*Chlamydia trachomatis L3/404/LN*
1	1	PVC group	*Chlamydia trachomatis RC-F(s)/342*
1	1	PVC group	*Chlamydia trachomatis RC-F(s)/852*
1	1	PVC group	*Chlamydia trachomatis RC-F/69*
1	1	PVC group	*Chlamydia trachomatis RC-J(s)/122*
1	1	PVC group	*Chlamydia trachomatis RC-J/943*
1	1	PVC group	*Chlamydia trachomatis RC-J/953*
1	1	PVC group	*Chlamydia trachomatis RC-J/966*
1	1	PVC group	*Chlamydia trachomatis RC-J/971*
1	1	PVC group	*Chlamydia trachomatis RC-L2(s)/3*
1	1	PVC group	*Chlamydia trachomatis RC-L2(s)/46*
1	1	PVC group	*Chlamydia trachomatis RC-L2/55*
1	1	PVC group	*Chlamydia trachomatis Sweden2*
2	1	PVC group	*Chlamydophila caviae GPIC*
1	1	PVC group	*Chlamydophila pecorum E58*
2	1	PVC group	*Chlamydophila pneumoniae CWL029*
2	1	PVC group	*Chlamydophila pneumoniae J138*
2	1	PVC group	*Chlamydophila pneumoniae LPCoLN*
2	1	PVC group	*Chlamydophila pneumoniae TW-183*
1	1	PVC group	*Coraliomargarita akajimensis DSM 45221*
3	2	PVC group	*Isosphaera pallida ATCC 43644*
1	1	PVC group	*Methylacidiphilum fumariolicum SolV*
1	1	PVC group	*Methylacidiphilum infernorum V4*

2	1	PVC group	*Opitutaceae bacterium TAV5*
2	1	PVC group	*Opitutus terrae PB90-1*
3	2	PVC group	*Parachlamydia acanthamoebae UV-7*
3	3	PVC group	*Phycisphaera mikurensis NBRC 102666*
3	4	PVC group	*Pirellula staleyi DSM 6068*
3	3	PVC group	*Planctopirus limnophila DSM 3776*
3	2	PVC group	*Protochlamydia naegleriophila*
3	3	PVC group	*Rubinisphaera brasiliensis DSM 5305*
3	2	PVC group	*Simkania negevensis Z*
3	2	PVC group	*Singulisphaera acidiphila DSM 18658*
1	1	PVC group	*Verrucomicrobia bacterium IMCC26134*
1	1	PVC group	*Verrucomicrobia bacterium L21-Fru-AB*
1	1	PVC group	*Verrucomicrobium spinosum DSM 4136 = JCM 18804*
3	2	PVC group	*Waddlia chondrophila WSU 86-1044*
1	1	Spirochaetes	*Borrelia afzelii HLJ01*
1	1	Spirochaetes	*Borrelia afzelii K78*
1	1	Spirochaetes	*Borrelia afzelii PKo*
1	1	Spirochaetes	*Borrelia afzelii Tom3107*
1	1	Spirochaetes	*Borrelia burgdorferi B31*
1	1	Spirochaetes	*Borrelia burgdorferi CA382*
1	1	Spirochaetes	*Borrelia burgdorferi JD1*
1	1	Spirochaetes	*Borrelia burgdorferi N40*
1	1	Spirochaetes	*Borrelia burgdorferi ZS7*
1	1	Spirochaetes	*Borrelia duttonii Ly*
1	1	Spirochaetes	*Borrelia garinii BgVir*
1	1	Spirochaetes	*Borrelia garinii NMJW1*
1	1	Spirochaetes	*Borrelia garinii SZ*
1	1	Spirochaetes	*Borrelia hermsii CC1*
1	1	Spirochaetes	*Borrelia miyamotoi*

1	1	Spirochaetes	*Borrelia miyamotoi LB-2001*
1	1	Spirochaetes	*Borrelia parkeri HR1*
1	1	Spirochaetes	*Borrelia recurrentis A1*
1	1	Spirochaetes	*Borrelia turicatae 91E135*
1	1	Spirochaetes	*Borreliella bavariensis PBi*
1	1	Spirochaetes	*Borreliella bissettii DN127*
1	1	Spirochaetes	*Borreliella valaisiana Tom4006*
1	1	Spirochaetes	*Borreliella valaisiana VS116*
1	1	Spirochaetes	*Brachyspira hyodysenteriae WA1*
1	1	Spirochaetes	*Brachyspira intermedia PWS/A*
1	1	Spirochaetes	*Brachyspira murdochii DSM 12563*
1	1	Spirochaetes	*Brachyspira pilosicoli 95/1000*
1	1	Spirochaetes	*Brachyspira pilosicoli B2904*
1	1	Spirochaetes	*Brachyspira pilosicoli P43/6/78*
1	1	Spirochaetes	*Leptospira biflexa serovar Patoc strain 'Patoc 1 (Ames)'*
1	1	Spirochaetes	*Leptospira biflexa serovar Patoc strain 'Patoc 1 (Paris)'*
1	1	Spirochaetes	*Leptospira borgpetersenii serovar Ballum*
1	1	Spirochaetes	*Leptospira borgpetersenii serovar Hardjo-bovis str. JB197*
1	1	Spirochaetes	*Leptospira borgpetersenii serovar Hardjo-bovis str. L550*
1	1	Spirochaetes	*Leptospira interrogans serovar Bratislava*
1	1	Spirochaetes	*Leptospira interrogans serovar Copenhageni str. Fiocruz L1-130*
1	1	Spirochaetes	*Leptospira interrogans serovar Hardjo str. Norma*
1	1	Spirochaetes	*Leptospira interrogans serovar Lai str. 56601*
1	1	Spirochaetes	*Leptospira interrogans serovar Lai str. IPAV*
1	1	Spirochaetes	*Leptospira interrogans serovar Linhai str. 56609*
1	1	Spirochaetes	*Leptospira interrogans serovar Manilae*
1	1	Spirochaetes	*Leptospira interrogans serovar Manilae*
1	1	Spirochaetes	*Leptospira santarosai serovar Shermani str. LT 821*

1	1	Spirochaetes	*Salinispira pacifica*
1	1	Spirochaetes	*Sphaerochaeta coccoides DSM 17374*
1	1	Spirochaetes	*Sphaerochaeta globosa str. Buddy*
1	1	Spirochaetes	*Sphaerochaeta pleomorpha str. Grapes*
1	1	Spirochaetes	*Spirochaeta africana DSM 8902*
1	1	Spirochaetes	*Spirochaeta smaragdinae DSM 11293*
1	1	Spirochaetes	*Spirochaeta thermophila DSM 6192*
1	1	Spirochaetes	*Spirochaeta thermophila DSM 6578*
1	1	Spirochaetes	*Treponema azotonutricium ZAS-9*
1	1	Spirochaetes	*Treponema brennaborense DSM 12168*
1	1	Spirochaetes	*Treponema caldarium DSM 7334*
1	1	Spirochaetes	*Treponema denticola ATCC 35405*
1	1	Spirochaetes	*Treponema pallidum subsp. pallidum str. Nichols*
1	1	Spirochaetes	*Treponema pallidum subsp. pallidum str. Sea 81-4*
1	1	Spirochaetes	*Treponema paraluiscuniculi Cuniculi A*
1	1	Spirochaetes	*Treponema pedis str. T A4*
1	1	Spirochaetes	*Treponema primitia ZAS-2*
1	1	Spirochaetes	*Treponema putidum*
1	1	Spirochaetes	*Treponema sp. OMZ 838*
1	1	Spirochaetes	*Treponema succinifaciens DSM 2489*
1	1	Spirochaetes	*Turneriella parva DSM 21527*
1	1	Synergistetes	*Aminobacterium colombiense DSM 12261*
1	1	Synergistetes	*Anaerobaculum mobile DSM 13181*
1	1	Synergistetes	*Thermanaerovibrio acidaminovorans DSM 6589*
1	1	Synergistetes	*Thermovirga lienii DSM 17291*
0	0	TACK group	*Acidianus hospitalis W1*
0	0	TACK group	*Acidilobus saccharovorans 345-15*
0	0	TACK group	*Caldisphaera lagunensis DSM 15908*
0	0	TACK group	*Caldivirga maquilingensis IC-167*

0	0	TACK group	*Candidatus Korarchaeum cryptofilum OPF8*
0	0	TACK group	*Candidatus Nitrosopelagicus brevis*
0	0	TACK group	*Candidatus Nitrosopumilus sp. AR2*
0	0	TACK group	*Candidatus Nitrosopumilus sp. NF5*
0	0	TACK group	*Candidatus Nitrosotenuis cloacae*
0	0	TACK group	*Desulfurococcus fermentans DSM 16532*
0	0	TACK group	*Desulfurococcus kamchatkensis 1221n*
0	0	TACK group	*Desulfurococcus mucosus DSM 2162*
0	0	TACK group	*Hyperthermus butylicus DSM 5456*
0	0	TACK group	*Ignicoccus hospitalis KIN4/I*
0	0	TACK group	*Metallosphaera cuprina Ar-4*
0	0	TACK group	*Metallosphaera sedula*
0	0	TACK group	*Metallosphaera sedula*
0	0	TACK group	*Metallosphaera sedula*
0	0	TACK group	*Metallosphaera sedula*
0	0	TACK group	*Metallosphaera sedula*
0	0	TACK group	*Metallosphaera sedula DSM 5348*
0	0	TACK group	*Pyrobaculum islandicum DSM 4184*
0	0	TACK group	*Pyrodictium delaneyi*
0	0	TACK group	*Pyrolobus fumarii 1A*
0	0	TACK group	*Staphylothermus hellenicus DSM 12710*
0	0	TACK group	*Staphylothermus marinus F1*
0	0	TACK group	*Sulfolobus acidocaldarius DSM 639*
0	0	TACK group	*Sulfolobus acidocaldarius N8*
0	0	TACK group	*Sulfolobus acidocaldarius Ron12/I*
0	0	TACK group	*Sulfolobus acidocaldarius SUSAZ*
0	0	TACK group	*Sulfolobus islandicus HVE10/4*
0	0	TACK group	*Sulfolobus islandicus L.S.2.15*
0	0	TACK group	*Sulfolobus islandicus LAL14/1*

0	0	TACK group	*Sulfolobus islandicus M.14.25*
0	0	TACK group	*Sulfolobus islandicus M.16.2*
0	0	TACK group	*Sulfolobus islandicus M.16.23*
0	0	TACK group	*Sulfolobus islandicus M.16.27*
0	0	TACK group	*Sulfolobus islandicus M.16.4*
0	0	TACK group	*Sulfolobus islandicus M.16.40*
0	0	TACK group	*Sulfolobus islandicus M.16.43*
0	0	TACK group	*Sulfolobus islandicus M.16.47*
0	0	TACK group	*Sulfolobus islandicus REY15A*
0	0	TACK group	*Sulfolobus islandicus Y.G.57.14*
0	0	TACK group	*Sulfolobus islandicus Y.N.15.51*
0	0	TACK group	*Sulfolobus solfataricus*
0	0	TACK group	*Sulfolobus solfataricus*
0	0	TACK group	*Sulfolobus solfataricus*
0	0	TACK group	*Sulfolobus solfataricus 98/2*
0	0	TACK group	*Sulfolobus solfataricus P2*
0	0	TACK group	*Sulfolobus tokodaii str. 7*
0	0	TACK group	*Thermofilum carboxyditrophus 1505*
0	0	TACK group	*Thermofilum pendens Hrk 5*
0	0	TACK group	*Thermofilum sp. 1807-2*
0	0	TACK group	*Thermofilum sp. 1910b*
0	0	TACK group	*Thermogladius cellulolyticus 1633*
0	0	TACK group	*Thermoproteus tenax Kra 1*
0	0	TACK group	*Thermoproteus uzoniensis 768-20*
0	0	TACK group	*Thermosphaera aggregans DSM 11486*
0	0	TACK group	*Vulcanisaeta distributa DSM 14429*
0	0	TACK group	*Vulcanisaeta moutnovskia 768-28*
1	1	Terrabacteria group	*[Bacillus thuringiensis] serovar konkukian str. 97-27*
1	1	Terrabacteria group	*[Bacillus] selenitireducens MLS10*

2	1	Terrabacteria group	*[Brevibacterium] flavum*
1	1	Terrabacteria group	*[Clostridium] acidurici 9a*
1	1	Terrabacteria group	*[Clostridium] cellulolyticum H10*
2	2	Terrabacteria group	*[Clostridium] cellulosi*
1	1	Terrabacteria group	*[Clostridium] clariflavum DSM 19732*
1	1	Terrabacteria group	*[Clostridium] saccharolyticum WM1*
2	2	Terrabacteria group	*[Clostridium] stercorarium subsp. stercorarium DSM 8532*
2	2	Terrabacteria group	*[Clostridium] stercorarium subsp. stercorarium DSM 8532*
1	1	Terrabacteria group	*[Clostridium] sticklandii*
1	1	Terrabacteria group	*[Eubacterium rectale] ATCC 33656*
1	1	Terrabacteria group	*[Eubacterium] eligens ATCC 27750*
3	2	Terrabacteria group	*Acaryochloris marina MBIC11017*
0	1	Terrabacteria group	*Acetobacterium woodii DSM 1030*
1	1	Terrabacteria group	*Acetohalobium arabaticum DSM 5501*
1	1	Terrabacteria group	*Acholeplasma brassicae*
1	1	Terrabacteria group	*Acholeplasma laidlawii PG-8A*
0	0	Terrabacteria group	*Acholeplasma oculi*
1	1	Terrabacteria group	*Acholeplasma palmae J233*
1	1	Terrabacteria group	*Acidaminococcus fermentans DSM 20731*
1	2	Terrabacteria group	*Acidaminococcus intestini RyC-MR95*
2	2	Terrabacteria group	*Acidimicrobium ferrooxidans DSM 10331*
2	2	Terrabacteria group	*Acidothermus cellulolyticus 11B*
1	2	Terrabacteria group	*Actinobacteria bacterium IMCC26256*
1	1	Terrabacteria group	*Actinomyces meyeri*
1	1	Terrabacteria group	*Actinomyces oris*
1	1	Terrabacteria group	*Actinomyces sp. oral taxon 414*
2	2	Terrabacteria group	*Actinoplanes friuliensis DSM 7358*
2	2	Terrabacteria group	*Actinoplanes missouriensis 431*
2	2	Terrabacteria group	*Actinoplanes sp. N902-109*

2	2	Terrabacteria group	*Actinoplanes sp. SE50/110*
2	2	Terrabacteria group	*Actinosynnema mirum DSM 43827*
1	1	Terrabacteria group	*Actinotignum schaalii*
1	1	Terrabacteria group	*Adlercreutzia equolifaciens DSM 19450*
1	1	Terrabacteria group	*Aerococcus christensenii*
1	1	Terrabacteria group	*Aerococcus urinae*
1	1	Terrabacteria group	*Aerococcus urinae ACS-120-V-Col10a*
1	1	Terrabacteria group	*Aerococcus urinaeequi*
1	1	Terrabacteria group	*Aerococcus urinaeequi*
1	1	Terrabacteria group	*Aerococcus viridans*
1	1	Terrabacteria group	*Alicyclobacillus acidocaldarius subsp. acidocaldarius DSM 446*
1	1	Terrabacteria group	*Alicyclobacillus acidocaldarius subsp. acidocaldarius Tc-4-1*
1	1	Terrabacteria group	*Alkaliphilus metalliredigens QYMF*
1	1	Terrabacteria group	*Alkaliphilus oremlandii OhILAs*
1	1	Terrabacteria group	*Ammonifex degensii KC4*
1	1	Terrabacteria group	*Amphibacillus xylanus NBRC 15112*
2	2	Terrabacteria group	*Amycolatopsis japonica*
4	2	Terrabacteria group	*Amycolatopsis mediterranei RB*
4	2	Terrabacteria group	*Amycolatopsis mediterranei S699*
4	2	Terrabacteria group	*Amycolatopsis mediterranei S699*
4	2	Terrabacteria group	*Amycolatopsis mediterranei U32*
4	2	Terrabacteria group	*Amycolatopsis methanolica 239*
2	2	Terrabacteria group	*Amycolatopsis orientalis HCCB10007*
3	1	Terrabacteria group	*Amycolicicoccus subflavus DQS3-9A1*
2	1	Terrabacteria group	*Anabaena cylindrica PCC 7122*
2	1	Terrabacteria group	*Anabaena sp. 90*
2	1	Terrabacteria group	*Anabaena sp. wa102*

1	1	Terrabacteria group	*Anaerococcus prevotii DSM 20548*
1	2	Terrabacteria group	*Anaerolinea thermophila UNI-1*
1	1	Terrabacteria group	*Aneurinibacillus sp. XH2*
1	1	Terrabacteria group	*Anoxybacillus flavithermus WK1*
1	1	Terrabacteria group	*Anoxybacillus gonensis*
1	1	Terrabacteria group	*Arcanobacterium haemolyticum DSM 20595*
2	1	Terrabacteria group	*Arsenicicoccus sp. oral taxon 190*
2	1	Terrabacteria group	*Arthrobacter alpinus*
2	1	Terrabacteria group	*Arthrobacter alpinus*
2	1	Terrabacteria group	*Arthrobacter sp. A3*
2	1	Terrabacteria group	*Arthrobacter sp. ATCC 21022*
2	1	Terrabacteria group	*Arthrobacter sp. ERGS1:01*
2	1	Terrabacteria group	*Arthrobacter sp. FB24*
2	1	Terrabacteria group	*Arthrobacter sp. IHBB 11108*
2	1	Terrabacteria group	*Arthrobacter sp. LS16*
2	1	Terrabacteria group	*Arthrobacter sp. PAMC 25486*
2	1	Terrabacteria group	*Arthrobacter sp. Rue61a*
2	1	Terrabacteria group	*Arthrobacter sp. YC-RL1*
1	1	Terrabacteria group	*Aster yellows witches'-broom phytoplasma AYWB*
1	1	Terrabacteria group	*Atopobium parvulum DSM 20469*
1	1	Terrabacteria group	*Bacillus amyloliquefaciens*
1	1	Terrabacteria group	*Bacillus amyloliquefaciens*
1	1	Terrabacteria group	*Bacillus amyloliquefaciens*
1	1	Terrabacteria group	*Bacillus amyloliquefaciens*
1	1	Terrabacteria group	*Bacillus amyloliquefaciens*
1	1	Terrabacteria group	*Bacillus amyloliquefaciens CC178*
1	1	Terrabacteria group	*Bacillus amyloliquefaciens DSM 7*
1	1	Terrabacteria group	*Bacillus amyloliquefaciens IT-45*
1	1	Terrabacteria group	*Bacillus amyloliquefaciens KHG19*

1	1	Terrabacteria group	*Bacillus amyloliquefaciens LFB112*
1	1	Terrabacteria group	*Bacillus amyloliquefaciens LL3*
1	1	Terrabacteria group	*Bacillus amyloliquefaciens TA208*
1	1	Terrabacteria group	*Bacillus amyloliquefaciens UMAF6614*
1	1	Terrabacteria group	*Bacillus amyloliquefaciens UMAF6639*
1	1	Terrabacteria group	*Bacillus amyloliquefaciens XH7*
1	1	Terrabacteria group	*Bacillus amyloliquefaciens Y2*
1	1	Terrabacteria group	*Bacillus anthracis*
1	1	Terrabacteria group	*Bacillus anthracis*
1	1	Terrabacteria group	*Bacillus anthracis*
1	1	Terrabacteria group	*Bacillus anthracis*
1	1	Terrabacteria group	*Bacillus anthracis*
1	1	Terrabacteria group	*Bacillus anthracis*
1	1	Terrabacteria group	*Bacillus anthracis*
1	1	Terrabacteria group	*Bacillus anthracis*
1	1	Terrabacteria group	*Bacillus anthracis*
1	1	Terrabacteria group	*Bacillus anthracis*
1	1	Terrabacteria group	*Bacillus anthracis*
1	1	Terrabacteria group	*Bacillus anthracis*
1	1	Terrabacteria group	*Bacillus anthracis*
1	1	Terrabacteria group	*Bacillus anthracis*
1	1	Terrabacteria group	*Bacillus anthracis*
1	1	Terrabacteria group	*Bacillus anthracis*
1	1	Terrabacteria group	*Bacillus anthracis str. A0248*
1	1	Terrabacteria group	*Bacillus anthracis str. A16*
1	1	Terrabacteria group	*Bacillus anthracis str. A16R*
1	1	Terrabacteria group	*Bacillus anthracis str. Ames*
1	1	Terrabacteria group	*Bacillus anthracis str. 'Ames Ancestor'*

1	1	Terrabacteria group	*Bacillus anthracis str. CDC 684*
1	1	Terrabacteria group	*Bacillus anthracis str. H9401*
1	1	Terrabacteria group	*Bacillus anthracis str. Sterne*
1	1	Terrabacteria group	*Bacillus anthracis str. Sterne*
1	1	Terrabacteria group	*Bacillus anthracis str. SVA11*
1	1	Terrabacteria group	*Bacillus anthracis str. Turkey32*
1	1	Terrabacteria group	*Bacillus anthracis str. V770-NP-1R*
1	1	Terrabacteria group	*Bacillus anthracis str. Vollum*
1	1	Terrabacteria group	*Bacillus atrophaeus*
1	1	Terrabacteria group	*Bacillus atrophaeus 1942*
1	1	Terrabacteria group	*Bacillus bombysepticus str. Wang*
1	1	Terrabacteria group	*Bacillus cellulosilyticus DSM 2522*
1	1	Terrabacteria group	*Bacillus cereus*
1	1	Terrabacteria group	*Bacillus cereus*
1	1	Terrabacteria group	*Bacillus cereus*
1	1	Terrabacteria group	*Bacillus cereus*
1	1	Terrabacteria group	*Bacillus cereus*
1	1	Terrabacteria group	*Bacillus cereus*
1	1	Terrabacteria group	*Bacillus cereus*
1	1	Terrabacteria group	*Bacillus cereus 03BB102*
1	1	Terrabacteria group	*Bacillus cereus 03BB102*
1	1	Terrabacteria group	*Bacillus cereus 03BB108*
1	1	Terrabacteria group	*Bacillus cereus AH187*
1	1	Terrabacteria group	*Bacillus cereus AH820*
0	1	Terrabacteria group	*Bacillus cereus ATCC 10987*
1	1	Terrabacteria group	*Bacillus cereus ATCC 14579*
1	1	Terrabacteria group	*Bacillus cereus ATCC 4342*
1	1	Terrabacteria group	*Bacillus cereus B4264*
1	1	Terrabacteria group	*Bacillus cereus biovar anthracis str. CI*

1	1	Terrabacteria group	*Bacillus cereus D17*
1	1	Terrabacteria group	*Bacillus cereus E33L*
1	1	Terrabacteria group	*Bacillus cereus E33L*
0	1	Terrabacteria group	*Bacillus cereus F837/76*
1	1	Terrabacteria group	*Bacillus cereus FRI-35*
1	1	Terrabacteria group	*Bacillus cereus G9241*
1	1	Terrabacteria group	*Bacillus cereus G9842*
1	1	Terrabacteria group	*Bacillus cereus NC7401*
1	1	Terrabacteria group	*Bacillus cereus Q1*
1	1	Terrabacteria group	*Bacillus clausii*
1	1	Terrabacteria group	*Bacillus clausii KSM-K16*
1	1	Terrabacteria group	*Bacillus coagulans*
1	1	Terrabacteria group	*Bacillus coagulans*
1	1	Terrabacteria group	*Bacillus coagulans 2-6*
1	1	Terrabacteria group	*Bacillus coagulans 36D1*
1	1	Terrabacteria group	*Bacillus coagulans DSM 1 = ATCC 7050*
1	1	Terrabacteria group	*Bacillus cytotoxicus NVH 391-98*
1	1	Terrabacteria group	*Bacillus endophyticus*
1	1	Terrabacteria group	*Bacillus halodurans C-125*
0	0	Terrabacteria group	*Bacillus infantis NRRL B-14911*
1	1	Terrabacteria group	*Bacillus lehensis G1*
1	1	Terrabacteria group	*Bacillus licheniformis*
1	1	Terrabacteria group	*Bacillus licheniformis DSM 13 = ATCC 14580*
1	1	Terrabacteria group	*Bacillus licheniformis DSM 13 = ATCC 14580*
1	1	Terrabacteria group	*Bacillus megaterium DSM 319*
1	1	Terrabacteria group	*Bacillus megaterium NBRC 15308 = ATCC 14581*
1	1	Terrabacteria group	*Bacillus megaterium Q3*
1	1	Terrabacteria group	*Bacillus megaterium QM B1551*
1	1	Terrabacteria group	*Bacillus megaterium WSH-002*

1	1	Terrabacteria group	*Bacillus methanolicus MGA3*
1	1	Terrabacteria group	*Bacillus mycoides*
1	1	Terrabacteria group	*Bacillus mycoides*
1	1	Terrabacteria group	*Bacillus paralicheniformis*
1	1	Terrabacteria group	*Bacillus paralicheniformis ATCC 9945a*
1	1	Terrabacteria group	*Bacillus pseudofirmus OF4*
1	1	Terrabacteria group	*Bacillus pumilus*
1	1	Terrabacteria group	*Bacillus pumilus*
1	1	Terrabacteria group	*Bacillus pumilus*
1	1	Terrabacteria group	*Bacillus pumilus*
1	1	Terrabacteria group	*Bacillus pumilus*
1	1	Terrabacteria group	*Bacillus pumilus*
1	1	Terrabacteria group	*Bacillus pumilus*
1	1	Terrabacteria group	*Bacillus pumilus*
1	1	Terrabacteria group	*Bacillus pumilus SAFR-032*
1	1	Terrabacteria group	*Bacillus simplex*
1	1	Terrabacteria group	*Bacillus smithii*
1	1	Terrabacteria group	*Bacillus sp.*
1	1	Terrabacteria group	*Bacillus sp. 1NLA3E*
1	1	Terrabacteria group	*Bacillus sp. BH072*
1	1	Terrabacteria group	*Bacillus sp. JS*
1	1	Terrabacteria group	*Bacillus sp. LM 4-2*
1	1	Terrabacteria group	*Bacillus sp. OxB-1*
1	1	Terrabacteria group	*Bacillus sp. Pc3*
1	1	Terrabacteria group	*Bacillus sp. SDLI1*
1	1	Terrabacteria group	*Bacillus sp. WP8*
1	1	Terrabacteria group	*Bacillus sp. X1(2014)*
1	1	Terrabacteria group	*Bacillus sp. YP1*
1	1	Terrabacteria group	*Bacillus subtilis*

1	1	Terrabacteria group	*Bacillus subtilis*
1	1	Terrabacteria group	*Bacillus subtilis*
1	1	Terrabacteria group	*Bacillus subtilis*
1	1	Terrabacteria group	*Bacillus subtilis*
1	1	Terrabacteria group	*Bacillus subtilis*
1	1	Terrabacteria group	*Bacillus subtilis*
1	1	Terrabacteria group	*Bacillus subtilis*
1	1	Terrabacteria group	*Bacillus subtilis*
1	1	Terrabacteria group	*Bacillus subtilis*
1	1	Terrabacteria group	*Bacillus subtilis BEST7003*
1	1	Terrabacteria group	*Bacillus subtilis BSn5*
1	1	Terrabacteria group	*Bacillus subtilis HJ5*
1	1	Terrabacteria group	*Bacillus subtilis KCTC 1028*
1	1	Terrabacteria group	*Bacillus subtilis PY79*
1	1	Terrabacteria group	*Bacillus subtilis QB928*
1	1	Terrabacteria group	*Bacillus subtilis subsp. globigii*
1	1	Terrabacteria group	*Bacillus subtilis subsp. inaquosorum*
1	1	Terrabacteria group	*Bacillus subtilis subsp. natto*
1	1	Terrabacteria group	*Bacillus subtilis subsp. natto BEST195*
1	1	Terrabacteria group	*Bacillus subtilis subsp. spizizenii*
1	1	Terrabacteria group	*Bacillus subtilis subsp. spizizenii str. W23*
1	1	Terrabacteria group	*Bacillus subtilis subsp. spizizenii TU-B-10*
1	1	Terrabacteria group	*Bacillus subtilis subsp. subtilis*
1	1	Terrabacteria group	*Bacillus subtilis subsp. subtilis*
1	1	Terrabacteria group	*Bacillus subtilis subsp. subtilis*
1	1	Terrabacteria group	*Bacillus subtilis subsp. subtilis*
1	1	Terrabacteria group	*Bacillus subtilis subsp. subtilis 6051-HGW*
1	1	Terrabacteria group	*Bacillus subtilis subsp. subtilis str. 168*
1	1	Terrabacteria group	*Bacillus subtilis subsp. subtilis str. 168*

1	1	Terrabacteria group	*Bacillus subtilis subsp. subtilis str. AG1839*
1	1	Terrabacteria group	*Bacillus subtilis subsp. subtilis str. BAB-1*
1	1	Terrabacteria group	*Bacillus subtilis subsp. subtilis str. BSP1*
1	1	Terrabacteria group	*Bacillus subtilis subsp. subtilis str. JH642 substr. AG174*
1	1	Terrabacteria group	*Bacillus subtilis subsp. subtilis str. OH 131.1*
1	1	Terrabacteria group	*Bacillus subtilis subsp. subtilis str. RO-NN-1*
1	1	Terrabacteria group	*Bacillus subtilis XF-1*
1	1	Terrabacteria group	*Bacillus thuringiensis*
1	1	Terrabacteria group	*Bacillus thuringiensis*
1	1	Terrabacteria group	*Bacillus thuringiensis*
1	1	Terrabacteria group	*Bacillus thuringiensis*
1	1	Terrabacteria group	*Bacillus thuringiensis*
1	1	Terrabacteria group	*Bacillus thuringiensis*
1	1	Terrabacteria group	*Bacillus thuringiensis*
1	1	Terrabacteria group	*Bacillus thuringiensis*
1	1	Terrabacteria group	*Bacillus thuringiensis*
1	1	Terrabacteria group	*Bacillus thuringiensis*
1	1	Terrabacteria group	*Bacillus thuringiensis BMB171*
1	1	Terrabacteria group	*Bacillus thuringiensis Bt407*
1	1	Terrabacteria group	*Bacillus thuringiensis HD1002*
1	1	Terrabacteria group	*Bacillus thuringiensis HD-771*
1	1	Terrabacteria group	*Bacillus thuringiensis HD-789*
1	1	Terrabacteria group	*Bacillus thuringiensis MC28*
1	1	Terrabacteria group	*Bacillus thuringiensis serovar chinensis CT-43*
1	1	Terrabacteria group	*Bacillus thuringiensis serovar finitimus YBT-020*
1	1	Terrabacteria group	*Bacillus thuringiensis serovar galleriae*
1	1	Terrabacteria group	*Bacillus thuringiensis serovar indiana*
1	1	Terrabacteria group	*Bacillus thuringiensis serovar kurstaki str. HD-1*
1	1	Terrabacteria group	*Bacillus thuringiensis serovar kurstaki str. HD73*

1	1	Terrabacteria group	*Bacillus thuringiensis serovar kurstaki str. YBT-1520*
1	1	Terrabacteria group	*Bacillus thuringiensis serovar kurstaki str. YBT-1520*
1	1	Terrabacteria group	*Bacillus thuringiensis serovar morrisoni*
1	1	Terrabacteria group	*Bacillus thuringiensis serovar thuringiensis str. IS5056*
1	1	Terrabacteria group	*Bacillus thuringiensis serovar tolworthi*
1	1	Terrabacteria group	*Bacillus thuringiensis str. Al Hakam*
1	1	Terrabacteria group	*Bacillus thuringiensis str. Al Hakam*
1	1	Terrabacteria group	*Bacillus thuringiensis YBT-1518*
1	1	Terrabacteria group	*Bacillus toyonensis BCT-7112*
1	1	Terrabacteria group	*Bacillus velezensis*
1	1	Terrabacteria group	*Bacillus velezensis*
1	1	Terrabacteria group	*Bacillus velezensis*
1	1	Terrabacteria group	*Bacillus velezensis*
1	1	Terrabacteria group	*Bacillus velezensis AS43.3*
1	1	Terrabacteria group	*Bacillus velezensis CAU B946*
1	1	Terrabacteria group	*Bacillus velezensis FZB42*
1	1	Terrabacteria group	*Bacillus velezensis NAU-B3*
1	1	Terrabacteria group	*Bacillus velezensis NJN-6*
1	1	Terrabacteria group	*Bacillus velezensis SQR9*
1	1	Terrabacteria group	*Bacillus velezensis TrigoCor1448*
1	1	Terrabacteria group	*Bacillus velezensis UCMB5033*
1	1	Terrabacteria group	*Bacillus velezensis UCMB5036*
1	1	Terrabacteria group	*Bacillus velezensis UCMB5113*
1	1	Terrabacteria group	*Bacillus velezensis YAU B9601-Y2*
1	1	Terrabacteria group	*Bacillus weihenstephanensis*
1	1	Terrabacteria group	*Bacillus weihenstephanensis KBAB4*
1	1	Terrabacteria group	*Beutenbergia cavernae DSM 12333*
1	1	Terrabacteria group	*Bifidobacterium actinocoloniiforme DSM 22766*
1	1	Terrabacteria group	*Bifidobacterium adolescentis*

1	1	Terrabacteria group	*Bifidobacterium adolescentis*
1	1	Terrabacteria group	*Bifidobacterium adolescentis ATCC 15703*
1	1	Terrabacteria group	*Bifidobacterium angulatum*
1	1	Terrabacteria group	*Bifidobacterium angulatum DSM 20098 = JCM 7096*
1	1	Terrabacteria group	*Bifidobacterium animalis*
1	1	Terrabacteria group	*Bifidobacterium animalis*
1	1	Terrabacteria group	*Bifidobacterium animalis subsp. animalis ATCC 25527*
1	1	Terrabacteria group	*Bifidobacterium animalis subsp. lactis*
1	1	Terrabacteria group	*Bifidobacterium animalis subsp. lactis AD011*
1	1	Terrabacteria group	*Bifidobacterium animalis subsp. lactis ATCC 27673*
1	1	Terrabacteria group	*Bifidobacterium animalis subsp. lactis B420*
1	1	Terrabacteria group	*Bifidobacterium animalis subsp. lactis BB-12*
1	1	Terrabacteria group	*Bifidobacterium animalis subsp. lactis Bi-07*
1	1	Terrabacteria group	*Bifidobacterium animalis subsp. lactis Bl-04*
1	1	Terrabacteria group	*Bifidobacterium animalis subsp. lactis Bl12*
1	1	Terrabacteria group	*Bifidobacterium animalis subsp. lactis BLC1*
1	1	Terrabacteria group	*Bifidobacterium animalis subsp. lactis CNCM I-2494*
1	1	Terrabacteria group	*Bifidobacterium animalis subsp. lactis DSM 10140*
1	1	Terrabacteria group	*Bifidobacterium animalis subsp. lactis KLDS2.0603*
1	1	Terrabacteria group	*Bifidobacterium animalis subsp. lactis V9*
1	1	Terrabacteria group	*Bifidobacterium asteroides PRL2011*
1	1	Terrabacteria group	*Bifidobacterium bifidum*
1	1	Terrabacteria group	*Bifidobacterium bifidum ATCC 29521 = JCM 1255 = DSM 20456*
1	1	Terrabacteria group	*Bifidobacterium bifidum BGN4*
1	1	Terrabacteria group	*Bifidobacterium bifidum PRL2010*
1	1	Terrabacteria group	*Bifidobacterium bifidum S17*
1	1	Terrabacteria group	*Bifidobacterium breve*
1	1	Terrabacteria group	*Bifidobacterium breve 12L*

1	1	Terrabacteria group	*Bifidobacterium breve 689b*
1	1	Terrabacteria group	*Bifidobacterium breve ACS-071-V-Sch8b*
1	1	Terrabacteria group	*Bifidobacterium breve DSM 20213 = JCM 1192*
1	1	Terrabacteria group	*Bifidobacterium breve JCM 7017*
1	1	Terrabacteria group	*Bifidobacterium breve JCM 7019*
1	1	Terrabacteria group	*Bifidobacterium breve NCFB 2258*
1	1	Terrabacteria group	*Bifidobacterium breve S27*
1	1	Terrabacteria group	*Bifidobacterium breve UCC2003*
1	1	Terrabacteria group	*Bifidobacterium catenulatum DSM 16992 = JCM 1194 = LMG 11043*
1	1	Terrabacteria group	*Bifidobacterium coryneforme*
1	1	Terrabacteria group	*Bifidobacterium dentium Bd1*
1	1	Terrabacteria group	*Bifidobacterium dentium JCM 1195 = DSM 20436*
1	1	Terrabacteria group	*Bifidobacterium indicum LMG 11587 = DSM 20214*
1	1	Terrabacteria group	*Bifidobacterium kashiwanohense JCM 15439 = DSM 21854*
1	1	Terrabacteria group	*Bifidobacterium kashiwanohense PV20-2*
1	1	Terrabacteria group	*Bifidobacterium longum*
1	1	Terrabacteria group	*Bifidobacterium longum*
1	1	Terrabacteria group	*Bifidobacterium longum*
1	1	Terrabacteria group	*Bifidobacterium longum DJO10A*
1	1	Terrabacteria group	*Bifidobacterium longum NCC2705*
1	1	Terrabacteria group	*Bifidobacterium longum subsp. infantis*
1	1	Terrabacteria group	*Bifidobacterium longum subsp. infantis 157F*
1	1	Terrabacteria group	*Bifidobacterium longum subsp. infantis ATCC 15697 = JCM 1222 = DSM 20088*
1	1	Terrabacteria group	*Bifidobacterium longum subsp. infantis ATCC 15697 = JCM 1222 = DSM 20088*
1	1	Terrabacteria group	*Bifidobacterium longum subsp. longum*
1	1	Terrabacteria group	*Bifidobacterium longum subsp. longum*

1	1	Terrabacteria group	*Bifidobacterium longum subsp. longum BBMN68*
1	1	Terrabacteria group	*Bifidobacterium longum subsp. longum GT15*
1	1	Terrabacteria group	*Bifidobacterium longum subsp. longum JCM 1217*
1	1	Terrabacteria group	*Bifidobacterium longum subsp. longum JDM301*
1	1	Terrabacteria group	*Bifidobacterium longum subsp. longum KACC 91563*
1	1	Terrabacteria group	*Bifidobacterium pseudocatenulatum DSM 20438 = JCM 1200 = LMG 10505*
1	1	Terrabacteria group	*Bifidobacterium pseudolongum PV8-2*
1	1	Terrabacteria group	*Bifidobacterium scardovii JCM 12489 = DSM 13734*
1	1	Terrabacteria group	*Bifidobacterium thermophilum RBL67*
2	2	Terrabacteria group	*Blastococcus saxobsidens DD2*
2	1	Terrabacteria group	*Brachybacterium faecium DSM 4810*
1	1	Terrabacteria group	*Brevibacillus brevis NBRC 100599*
1	1	Terrabacteria group	*Brevibacillus laterosporus LMG 15441*
1	1	Terrabacteria group	*Butyrivibrio proteoclasticus B316*
1	2	Terrabacteria group	*Caldanaerobacter subterraneus subsp. tengcongensis MB4*
1	1	Terrabacteria group	*Caldicellulosiruptor bescii DSM 6725*
1	1	Terrabacteria group	*Caldicellulosiruptor hydrothermalis 108*
1	1	Terrabacteria group	*Caldicellulosiruptor kristjanssonii I77R1B*
1	1	Terrabacteria group	*Caldicellulosiruptor kronotskyensis 2002*
1	1	Terrabacteria group	*Caldicellulosiruptor lactoaceticus 6A*
1	1	Terrabacteria group	*Caldicellulosiruptor obsidiansis OB47*
1	1	Terrabacteria group	*Caldicellulosiruptor owensensis OL*
1	1	Terrabacteria group	*Caldicellulosiruptor saccharolyticus DSM 8903*
1	2	Terrabacteria group	*Caldilinea aerophila DSM 14535 = NBRC 104270*
2	2	Terrabacteria group	*Calothrix sp. 336/3*
2	1	Terrabacteria group	*Calothrix sp. PCC 6303*
2	1	Terrabacteria group	*Calothrix sp. PCC 7507*
1	1	Terrabacteria group	*Candidatus Arthromitus sp. SFB-mouse-Japan*

1	1	Terrabacteria group	*Candidatus Arthromitus sp. SFB-mouse-NL*
1	1	Terrabacteria group	*Candidatus Arthromitus sp. SFB-mouse-Yit*
1	1	Terrabacteria group	*Candidatus Arthromitus sp. SFB-rat-Yit*
2	1	Terrabacteria group	*Candidatus Atelocyanobacterium thalassa isolate ALOHA*
1	1	Terrabacteria group	*Candidatus Desulforudis audaxviator MP104C*
1	1	Terrabacteria group	*Candidatus Phytoplasma australiense*
1	1	Terrabacteria group	*Candidatus Phytoplasma mali*
1	1	Terrabacteria group	*Carboxydothermus hydrogenoformans Z-2901*
1	1	Terrabacteria group	*Carnobacterium inhibens subsp. gilichinskyi*
1	1	Terrabacteria group	*Carnobacterium maltaromaticum LMA28*
1	1	Terrabacteria group	*Carnobacterium sp. 17-4*
1	1	Terrabacteria group	*Carnobacterium sp. CP1*
2	2	Terrabacteria group	*Catenulispora acidiphila DSM 44928*
1	2	Terrabacteria group	*Cellulomonas fimi ATCC 484*
1	2	Terrabacteria group	*Cellulomonas flavigena DSM 20109*
1	2	Terrabacteria group	*Cellulomonas gilvus ATCC 13127*
2	2	Terrabacteria group	*Chloroflexus aggregans DSM 9485*
2	2	Terrabacteria group	*Chloroflexus aurantiacus J-10-fl*
2	2	Terrabacteria group	*Chloroflexus sp. Y-400-fl*
2	1	Terrabacteria group	*Chroococcidiopsis thermalis PCC 7203*
1	1	Terrabacteria group	*Clavibacter michiganensis*
1	1	Terrabacteria group	*Clavibacter michiganensis subsp. insidiosus*
1	1	Terrabacteria group	*Clavibacter michiganensis subsp. michiganensis NCPPB 382*
1	1	Terrabacteria group	*Clavibacter michiganensis subsp. nebraskensis NCPPB 2581*
1	1	Terrabacteria group	*Clavibacter michiganensis subsp. sepedonicus*
1	1	Terrabacteria group	*Clostridium aceticum*
1	1	Terrabacteria group	*Clostridium acetobutylicum ATCC 824*

1	1	Terrabacteria group	*Clostridium acetobutylicum DSM 1731*
1	1	Terrabacteria group	*Clostridium acetobutylicum EA 2018*
1	1	Terrabacteria group	*Clostridium autoethanogenum DSM 10061*
1	1	Terrabacteria group	*Clostridium autoethanogenum DSM 10061*
2	2	Terrabacteria group	*Clostridium baratii str. Sullivan*
1	1	Terrabacteria group	*Clostridium beijerinckii*
1	1	Terrabacteria group	*Clostridium beijerinckii ATCC 35702*
1	1	Terrabacteria group	*Clostridium beijerinckii NCIMB 8052*
1	1	Terrabacteria group	*Clostridium bornimense*
1	1	Terrabacteria group	*Clostridium botulinum*
1	1	Terrabacteria group	*Clostridium botulinum*
1	1	Terrabacteria group	*Clostridium botulinum*
1	1	Terrabacteria group	*Clostridium botulinum 202F*
1	1	Terrabacteria group	*Clostridium botulinum A str. ATCC 19397*
1	1	Terrabacteria group	*Clostridium botulinum A str. ATCC 3502*
1	1	Terrabacteria group	*Clostridium botulinum A str. Hall*
1	1	Terrabacteria group	*Clostridium botulinum A2 str. Kyoto*
1	1	Terrabacteria group	*Clostridium botulinum A3 str. Loch Maree*
1	1	Terrabacteria group	*Clostridium botulinum B str. Eklund 17B (NRP)*
1	1	Terrabacteria group	*Clostridium botulinum B1 str. Okra*
1	1	Terrabacteria group	*Clostridium botulinum Ba4 str. 657*
1	1	Terrabacteria group	*Clostridium botulinum BKT015925*
1	1	Terrabacteria group	*Clostridium botulinum CDC_1436*
1	1	Terrabacteria group	*Clostridium botulinum E3 str. Alaska E43*
1	1	Terrabacteria group	*Clostridium botulinum F str. 230613*
1	1	Terrabacteria group	*Clostridium botulinum F str. Langeland*
1	1	Terrabacteria group	*Clostridium botulinum H04402 065*
1	1	Terrabacteria group	*Clostridium butyricum*
1	1	Terrabacteria group	*Clostridium butyricum*

1	1	Terrabacteria group	*Clostridium carboxidivorans P7*
1	1	Terrabacteria group	*Clostridium cellulovorans 743B*
1	1	Terrabacteria group	*Clostridium kluyveri DSM 555*
1	1	Terrabacteria group	*Clostridium kluyveri NBRC 12016*
1	1	Terrabacteria group	*Clostridium lentocellum DSM 5427*
1	1	Terrabacteria group	*Clostridium ljungdahlii DSM 13528*
1	1	Terrabacteria group	*Clostridium novyi NT*
1	1	Terrabacteria group	*Clostridium pasteurianum BC1*
1	1	Terrabacteria group	*Clostridium pasteurianum DSM 525 = ATCC 6013*
1	1	Terrabacteria group	*Clostridium pasteurianum DSM 525 = ATCC 6013*
1	1	Terrabacteria group	*Clostridium pasteurianum NRRL B-598*
1	1	Terrabacteria group	*Clostridium perfringens*
1	1	Terrabacteria group	*Clostridium perfringens*
1	1	Terrabacteria group	*Clostridium perfringens*
1	1	Terrabacteria group	*Clostridium perfringens ATCC 13124*
1	1	Terrabacteria group	*Clostridium perfringens str. 13*
1	1	Terrabacteria group	*Clostridium saccharobutylicum DSM 13864*
1	2	Terrabacteria group	*Clostridium saccharoperbutylacetonicum N1-4(HMT)*
1	1	Terrabacteria group	*Clostridium scatologenes*
1	1	Terrabacteria group	*Clostridium sp. BNL1100*
1	1	Terrabacteria group	*Clostridium sp. SY8519*
1	1	Terrabacteria group	*Clostridium sporogenes*
1	1	Terrabacteria group	*Clostridium sporogenes*
1	1	Terrabacteria group	*Clostridium tetani 12124569*
1	1	Terrabacteria group	*Clostridium tetani E88*
1	2	Terrabacteria group	*Conexibacter woesei DSM 14684*
1	1	Terrabacteria group	*Coprothermobacter proteolyticus DSM 5265*
1	1	Terrabacteria group	*Coriobacteriaceae bacterium 68-1-3*
1	1	Terrabacteria group	*Coriobacterium glomerans PW2*

2	1	Terrabacteria group	*Corynebacteriales bacterium X1036*
2	1	Terrabacteria group	*Corynebacteriales bacterium X1698*
2	1	Terrabacteria group	*Corynebacterium argentoratense DSM 44202*
2	1	Terrabacteria group	*Corynebacterium atypicum*
2	1	Terrabacteria group	*Corynebacterium aurimucosum ATCC 700975*
2	1	Terrabacteria group	*Corynebacterium callunae DSM 20147*
2	1	Terrabacteria group	*Corynebacterium camporealensis*
2	1	Terrabacteria group	*Corynebacterium casei LMG S-19264*
2	1	Terrabacteria group	*Corynebacterium deserti GIMN1.010*
2	1	Terrabacteria group	*Corynebacterium diphtheriae*
2	1	Terrabacteria group	*Corynebacterium diphtheriae 241*
2	1	Terrabacteria group	*Corynebacterium diphtheriae 31A*
2	1	Terrabacteria group	*Corynebacterium diphtheriae BH8*
2	1	Terrabacteria group	*Corynebacterium diphtheriae C7 (beta)*
2	1	Terrabacteria group	*Corynebacterium diphtheriae CDCE 8392*
2	1	Terrabacteria group	*Corynebacterium diphtheriae HC01*
2	1	Terrabacteria group	*Corynebacterium diphtheriae HC02*
2	1	Terrabacteria group	*Corynebacterium diphtheriae HC03*
3	1	Terrabacteria group	*Corynebacterium diphtheriae HC04*
2	1	Terrabacteria group	*Corynebacterium diphtheriae INCA 402*
2	1	Terrabacteria group	*Corynebacterium diphtheriae PW8*
2	1	Terrabacteria group	*Corynebacterium diphtheriae VA01*
2	1	Terrabacteria group	*Corynebacterium doosanense CAU 212 = DSM 45436*
2	1	Terrabacteria group	*Corynebacterium efficiens YS-314*
2	1	Terrabacteria group	*Corynebacterium epidermidicanis*
2	1	Terrabacteria group	*Corynebacterium falsenii DSM 44353*
2	1	Terrabacteria group	*Corynebacterium glutamicum*
2	1	Terrabacteria group	*Corynebacterium glutamicum*
2	1	Terrabacteria group	*Corynebacterium glutamicum*

2	1	Terrabacteria group	*Corynebacterium glutamicum*
2	1	Terrabacteria group	*Corynebacterium glutamicum*
2	1	Terrabacteria group	*Corynebacterium glutamicum ATCC 13032*
2	1	Terrabacteria group	*Corynebacterium glutamicum K051*
1	1	Terrabacteria group	*Corynebacterium glutamicum MB001*
2	1	Terrabacteria group	*Corynebacterium glutamicum R*
2	1	Terrabacteria group	*Corynebacterium glutamicum SCgG1*
2	1	Terrabacteria group	*Corynebacterium glutamicum SCgG2*
2	1	Terrabacteria group	*Corynebacterium glyciniphilum AJ 3170*
2	1	Terrabacteria group	*Corynebacterium halotolerans YIM 70093 = DSM 44683*
2	1	Terrabacteria group	*Corynebacterium humireducens NBRC 106098 = DSM 45392*
2	1	Terrabacteria group	*Corynebacterium imitans*
2	1	Terrabacteria group	*Corynebacterium jeikeium K411*
2	1	Terrabacteria group	*Corynebacterium kroppenstedtii DSM 44385*
2	1	Terrabacteria group	*Corynebacterium lactis RW2-5*
2	1	Terrabacteria group	*Corynebacterium marinum DSM 44953*
2	1	Terrabacteria group	*Corynebacterium maris DSM 45190*
2	1	Terrabacteria group	*Corynebacterium mustelae*
1	1	Terrabacteria group	*Corynebacterium pseudotuberculosis*
2	1	Terrabacteria group	*Corynebacterium pseudotuberculosis*
2	1	Terrabacteria group	*Corynebacterium pseudotuberculosis*
2	1	Terrabacteria group	*Corynebacterium pseudotuberculosis*
2	1	Terrabacteria group	*Corynebacterium pseudotuberculosis*
2	1	Terrabacteria group	*Corynebacterium pseudotuberculosis*
2	1	Terrabacteria group	*Corynebacterium pseudotuberculosis*
2	1	Terrabacteria group	*Corynebacterium pseudotuberculosis*
2	1	Terrabacteria group	*Corynebacterium pseudotuberculosis*
2	1	Terrabacteria group	*Corynebacterium pseudotuberculosis*

2	1	Terrabacteria group	*Corynebacterium pseudotuberculosis*
2	1	Terrabacteria group	*Corynebacterium pseudotuberculosis*
2	1	Terrabacteria group	*Corynebacterium pseudotuberculosis*
2	1	Terrabacteria group	*Corynebacterium pseudotuberculosis*
2	1	Terrabacteria group	*Corynebacterium pseudotuberculosis*
2	1	Terrabacteria group	*Corynebacterium pseudotuberculosis*
2	1	Terrabacteria group	*Corynebacterium pseudotuberculosis*
2	1	Terrabacteria group	*Corynebacterium pseudotuberculosis*
2	1	Terrabacteria group	*Corynebacterium pseudotuberculosis*
2	1	Terrabacteria group	*Corynebacterium pseudotuberculosis*
2	1	Terrabacteria group	*Corynebacterium pseudotuberculosis 1/06-A*
2	1	Terrabacteria group	*Corynebacterium pseudotuberculosis 1002*
2	1	Terrabacteria group	*Corynebacterium pseudotuberculosis 258*
2	1	Terrabacteria group	*Corynebacterium pseudotuberculosis 267*
2	1	Terrabacteria group	*Corynebacterium pseudotuberculosis 3/99-5*
2	1	Terrabacteria group	*Corynebacterium pseudotuberculosis 31*
2	1	Terrabacteria group	*Corynebacterium pseudotuberculosis 316*
2	1	Terrabacteria group	*Corynebacterium pseudotuberculosis 42/02-A*
2	1	Terrabacteria group	*Corynebacterium pseudotuberculosis C231*
2	1	Terrabacteria group	*Corynebacterium pseudotuberculosis CIP 52.97*
2	1	Terrabacteria group	*Corynebacterium pseudotuberculosis Cp162*
2	1	Terrabacteria group	*Corynebacterium pseudotuberculosis FRC41*
2	1	Terrabacteria group	*Corynebacterium pseudotuberculosis I19*
2	1	Terrabacteria group	*Corynebacterium pseudotuberculosis P54B96*
2	1	Terrabacteria group	*Corynebacterium pseudotuberculosis PAT10*
2	1	Terrabacteria group	*Corynebacterium resistens DSM 45100*
2	1	Terrabacteria group	*Corynebacterium simulans*
2	1	Terrabacteria group	*Corynebacterium simulans*
2	1	Terrabacteria group	*Corynebacterium singulare*

2	1	Terrabacteria group	*Corynebacterium sp. ATCC 6931*
2	1	Terrabacteria group	*Corynebacterium terpenotabidum Y-11*
2	1	Terrabacteria group	*Corynebacterium testudinoris*
2	1	Terrabacteria group	*Corynebacterium ulcerans*
2	1	Terrabacteria group	*Corynebacterium ulcerans*
2	1	Terrabacteria group	*Corynebacterium ulcerans*
2	1	Terrabacteria group	*Corynebacterium ulcerans*
2	1	Terrabacteria group	*Corynebacterium ulcerans*
2	1	Terrabacteria group	*Corynebacterium ulcerans 0102*
2	1	Terrabacteria group	*Corynebacterium ulcerans 809*
2	1	Terrabacteria group	*Corynebacterium ulcerans BR-AD22*
2	1	Terrabacteria group	*Corynebacterium ulcerans FRC11*
2	1	Terrabacteria group	*Corynebacterium ulcerans FRC58*
2	1	Terrabacteria group	*Corynebacterium urealyticum DSM 7109*
2	1	Terrabacteria group	*Corynebacterium urealyticum DSM 7111*
2	1	Terrabacteria group	*Corynebacterium ureicelerivorans*
2	1	Terrabacteria group	*Corynebacterium uterequi*
2	1	Terrabacteria group	*Corynebacterium variabile DSM 44702*
2	1	Terrabacteria group	*Corynebacterium vitaeruminis DSM 20294*
2	1	Terrabacteria group	*Crinalium epipsammum PCC 9333*
1	1	Terrabacteria group	*Cryptobacterium curtum DSM 15641*
2	1	Terrabacteria group	*Cyanobacterium aponinum PCC 10605*
2	1	Terrabacteria group	*cyanobacterium endosymbiont of Epithemia turgida isolate EtSB Lake Yunoko*
2	1	Terrabacteria group	*Cyanobium gracile PCC 6307*
2	1	Terrabacteria group	*Cyanothece sp. ATCC 51142*
2	1	Terrabacteria group	*Cyanothece sp. PCC 7424*
2	1	Terrabacteria group	*Cyanothece sp. PCC 7425*
2	1	Terrabacteria group	*Cyanothece sp. PCC 7822*

2	1	Terrabacteria group	*Cyanothece sp. PCC 8801*
2	1	Terrabacteria group	*Cyanothece sp. PCC 8802*
2	1	Terrabacteria group	*Dactylococcopsis salina PCC 8305*
2	1	Terrabacteria group	*Dehalobacter restrictus DSM 9455*
1	1	Terrabacteria group	*Dehalobacter sp. CF*
1	1	Terrabacteria group	*Dehalobacter sp. DCA*
1	1	Terrabacteria group	*Dehalococcoides mccartyi 195*
1	1	Terrabacteria group	*Dehalococcoides mccartyi BAV1*
1	1	Terrabacteria group	*Dehalococcoides mccartyi BTF08*
1	1	Terrabacteria group	*Dehalococcoides mccartyi CBDB1*
1	1	Terrabacteria group	*Dehalococcoides mccartyi CG1*
1	1	Terrabacteria group	*Dehalococcoides mccartyi CG4*
1	1	Terrabacteria group	*Dehalococcoides mccartyi CG5*
1	1	Terrabacteria group	*Dehalococcoides mccartyi DCMB5*
1	1	Terrabacteria group	*Dehalococcoides mccartyi GT*
1	1	Terrabacteria group	*Dehalococcoides mccartyi GY50*
1	1	Terrabacteria group	*Dehalococcoides mccartyi IBARAKI*
1	1	Terrabacteria group	*Dehalococcoides mccartyi VS*
1	1	Terrabacteria group	*Dehalococcoides sp. UCH007*
1	1	Terrabacteria group	*Dehalogenimonas lykanthroporepellens BL-DC-9*
1	1	Terrabacteria group	*Dehalogenimonas sp. WBC-2*
1	1	Terrabacteria group	*Deinococcus actinosclerus*
1	1	Terrabacteria group	*Deinococcus deserti VCD115*
1	1	Terrabacteria group	*Deinococcus geothermalis DSM 11300*
1	1	Terrabacteria group	*Deinococcus gobiensis I-0*
1	1	Terrabacteria group	*Deinococcus maricopensis DSM 21211*
1	1	Terrabacteria group	*Deinococcus peraridilitoris DSM 19664*
1	1	Terrabacteria group	*Deinococcus proteolyticus MRP*
1	1	Terrabacteria group	*Deinococcus radiodurans R1*

1	1	Terrabacteria group	*'Deinococcus soli' Cha et al. 2014*
1	1	Terrabacteria group	*Deinococcus swuensis*
2	1	Terrabacteria group	*Dermacoccus nishinomiyaensis*
3	1	Terrabacteria group	*Desulfitobacterium dehalogenans ATCC 51507*
1	1	Terrabacteria group	*Desulfitobacterium dichloroeliminans LMG P-21439*
5	1	Terrabacteria group	*Desulfitobacterium hafniense DCB-2*
1	1	Terrabacteria group	*Desulfitobacterium hafniense Y51*
1	1	Terrabacteria group	*Desulfitobacterium metallireducens DSM 15288*
1	1	Terrabacteria group	*Desulfosporosinus acidiphilus SJ4*
1	1	Terrabacteria group	*Desulfosporosinus meridiei DSM 13257*
1	1	Terrabacteria group	*Desulfosporosinus orientis DSM 765*
1	1	Terrabacteria group	*Desulfotomaculum acetoxidans DSM 771*
1	1	Terrabacteria group	*Desulfotomaculum gibsoniae DSM 7213*
1	1	Terrabacteria group	*Desulfotomaculum nigrificans CO-1-SRB*
1	1	Terrabacteria group	*Desulfotomaculum reducens MI-1*
1	1	Terrabacteria group	*Desulfotomaculum ruminis DSM 2154*
2	1	Terrabacteria group	*Eggerthella lenta DSM 2243*
1	1	Terrabacteria group	*Eggerthella sp. YY7918*
1	1	Terrabacteria group	*Enterococcus casseliflavus EC20*
1	1	Terrabacteria group	*Enterococcus durans*
1	1	Terrabacteria group	*Enterococcus durans*
1	1	Terrabacteria group	*Enterococcus faecalis*
1	1	Terrabacteria group	*Enterococcus faecalis ATCC 29212*
1	1	Terrabacteria group	*Enterococcus faecalis D32*
1	1	Terrabacteria group	*Enterococcus faecalis DENG1*
1	1	Terrabacteria group	*Enterococcus faecalis OG1RF*
1	1	Terrabacteria group	*Enterococcus faecalis str. Symbioflor 1*
1	1	Terrabacteria group	*Enterococcus faecalis V583*
1	1	Terrabacteria group	*Enterococcus faecium*

1	1	Terrabacteria group	*Enterococcus faecium*
1	1	Terrabacteria group	*Enterococcus faecium*
1	1	Terrabacteria group	*Enterococcus faecium*
1	1	Terrabacteria group	*Enterococcus faecium*
1	1	Terrabacteria group	*Enterococcus faecium Aus0004*
1	1	Terrabacteria group	*Enterococcus faecium Aus0085*
1	1	Terrabacteria group	*Enterococcus faecium DO*
1	1	Terrabacteria group	*Enterococcus faecium NRRL B-2354*
1	1	Terrabacteria group	*Enterococcus faecium T110*
1	1	Terrabacteria group	*Enterococcus gallinarum*
1	1	Terrabacteria group	*Enterococcus hirae ATCC 9790*
1	1	Terrabacteria group	*Enterococcus mundtii QU 25*
1	1	Terrabacteria group	*Erysipelothrix rhusiopathiae*
1	1	Terrabacteria group	*Erysipelothrix rhusiopathiae str. Fujisawa*
1	1	Terrabacteria group	*Erysipelothrix rhusiopathiae SY1027*
1	1	Terrabacteria group	*Ethanoligenens harbinense YUAN-3*
1	1	Terrabacteria group	*Eubacterium acidaminophilum DSM 3953*
1	1	Terrabacteria group	*Eubacterium limosum*
1	1	Terrabacteria group	*Eubacterium limosum KIST612*
1	1	Terrabacteria group	*Eubacterium sulci ATCC 35585*
1	1	Terrabacteria group	*Exiguobacterium antarcticum B7*
1	1	Terrabacteria group	*Exiguobacterium sibiricum 255-15*
1	1	Terrabacteria group	*Exiguobacterium sp. AT1b*
1	1	Terrabacteria group	*Exiguobacterium sp. MH3*
1	1	Terrabacteria group	*Filifactor alocis ATCC 35896*
2	2	Terrabacteria group	*Fimbriimonas ginsengisoli Gsoil 348*
1	1	Terrabacteria group	*Finegoldia magna ATCC 29328*
3	2	Terrabacteria group	*Fischerella sp. NIES-3754*
4	2	Terrabacteria group	*Frankia alni ACN14a*

4	2	Terrabacteria group	*Frankia sp. CcI3*
4	2	Terrabacteria group	*Frankia sp. EAN1pec*
2	2	Terrabacteria group	*Frankia sp. EuI1c*
2	2	Terrabacteria group	*Frankia symbiont of Datisca glomerata*
1	1	Terrabacteria group	*Gardnerella vaginalis 409-05*
1	1	Terrabacteria group	*Gardnerella vaginalis ATCC 14018 = JCM 11026*
1	1	Terrabacteria group	*Gardnerella vaginalis ATCC 14019*
1	1	Terrabacteria group	*Gardnerella vaginalis HMP9231*
2	1	Terrabacteria group	*Geitlerinema sp. PCC 7407*
1	1	Terrabacteria group	*Gemella sp. oral taxon 928*
1	1	Terrabacteria group	*Geobacillus kaustophilus HTA426*
1	1	Terrabacteria group	*Geobacillus sp. 12AMOR1*
1	1	Terrabacteria group	*Geobacillus sp. C56-T3*
1	1	Terrabacteria group	*Geobacillus sp. GHH01*
1	1	Terrabacteria group	*Geobacillus sp. JF8*
1	1	Terrabacteria group	*Geobacillus sp. LC300*
1	1	Terrabacteria group	*Geobacillus sp. WCH70*
1	1	Terrabacteria group	*Geobacillus sp. Y4.1MC1*
1	1	Terrabacteria group	*Geobacillus sp. Y412MC52*
1	1	Terrabacteria group	*Geobacillus sp. Y412MC61*
1	1	Terrabacteria group	*Geobacillus stearothermophilus 10*
1	1	Terrabacteria group	*Geobacillus thermodenitrificans NG80-2*
1	1	Terrabacteria group	*Geobacillus thermoglucosidasius*
1	1	Terrabacteria group	*Geobacillus thermoglucosidasius C56-YS93*
1	1	Terrabacteria group	*Geobacillus thermoleovorans CCB_US3_UF5*
2	2	Terrabacteria group	*Geodermatophilus obscurus DSM 43160*
2	2	Terrabacteria group	*Gloeobacter kilaueensis JS1*
2	2	Terrabacteria group	*Gloeobacter violaceus PCC 7421*
2	1	Terrabacteria group	*Gloeocapsa sp. PCC 7428*

2	1	Terrabacteria group	*Glutamicibacter arilaitensis*
2	1	Terrabacteria group	*Glutamicibacter arilaitensis Re117*
3	1	Terrabacteria group	*Gordonia bronchialis DSM 43247*
3	1	Terrabacteria group	*Gordonia polyisoprenivorans VH2*
3	1	Terrabacteria group	*Gordonia sp. KTR9*
1	1	Terrabacteria group	*Gordonia sp. QH-11*
1	1	Terrabacteria group	*Halanaerobium hydrogeniformans*
1	1	Terrabacteria group	*Halanaerobium praevalens DSM 2228*
1	1	Terrabacteria group	*Halobacillus halophilus DSM 2266*
1	1	Terrabacteria group	*Halobacteroides halobius DSM 5150*
2	1	Terrabacteria group	*Halothece sp. PCC 7418*
1	1	Terrabacteria group	*Halothermothrix orenii H 168*
1	1	Terrabacteria group	*Heliobacterium modesticaldum Ice1*
1	2	Terrabacteria group	*Ilumatobacter coccineus YM16-304*
1	1	Terrabacteria group	*Intestinimonas butyriciproducens*
2	2	Terrabacteria group	*Intrasporangium calvum DSM 43043*
1	2	Terrabacteria group	*Isoptericola variabilis 225*
1	1	Terrabacteria group	*Jeotgalibacillus sp. D5*
1	1	Terrabacteria group	*Jeotgalicoccus sp. 13MG44_air*
1	2	Terrabacteria group	*Jonesia denitrificans DSM 20603*
3	2	Terrabacteria group	*Kibdelosporangium phytohabitans*
2	2	Terrabacteria group	*Kineococcus radiotolerans SRS30216 = ATCC BAA-149*
2	2	Terrabacteria group	*Kitasatospora setae KM-6054*
2	1	Terrabacteria group	*Kocuria flava*
2	1	Terrabacteria group	*Kocuria palustris*
2	1	Terrabacteria group	*Kocuria rhizophila DC2201*
2	2	Terrabacteria group	*Kribbella flavida DSM 17836*
2	2	Terrabacteria group	*Kutzneria albida DSM 43870*
1	1	Terrabacteria group	*Kyrpidia tusciae DSM 2912*

2	1	Terrabacteria group	*Kytococcus sedentarius DSM 20547*
1	1	Terrabacteria group	*Lachnoclostridium phytofermentans ISDg*
1	1	Terrabacteria group	*Lactobacillus acetotolerans*
1	1	Terrabacteria group	*Lactobacillus acidophilus*
1	1	Terrabacteria group	*Lactobacillus acidophilus La-14*
1	1	Terrabacteria group	*Lactobacillus acidophilus NCFM*
1	1	Terrabacteria group	*Lactobacillus amylovorus GRL1118*
1	1	Terrabacteria group	*Lactobacillus brevis ATCC 367*
1	1	Terrabacteria group	*Lactobacillus brevis KB290*
1	1	Terrabacteria group	*Lactobacillus buchneri CD034*
1	1	Terrabacteria group	*Lactobacillus buchneri NRRL B-30929*
1	1	Terrabacteria group	*Lactobacillus casei 12A*
1	1	Terrabacteria group	*Lactobacillus casei BD-II*
1	1	Terrabacteria group	*Lactobacillus casei BL23*
1	1	Terrabacteria group	*Lactobacillus casei LC2W*
1	1	Terrabacteria group	*Lactobacillus casei LOCK919*
1	1	Terrabacteria group	*Lactobacillus casei str. Zhang*
1	1	Terrabacteria group	*Lactobacillus casei subsp. casei ATCC 393*
1	1	Terrabacteria group	*Lactobacillus casei W56*
1	1	Terrabacteria group	*Lactobacillus delbrueckii subsp. bulgaricus*
1	1	Terrabacteria group	*Lactobacillus delbrueckii subsp. bulgaricus 2038*
1	1	Terrabacteria group	*Lactobacillus delbrueckii subsp. bulgaricus ATCC 11842 = JCM 1002*
1	1	Terrabacteria group	*Lactobacillus delbrueckii subsp. bulgaricus ATCC BAA-365*
1	1	Terrabacteria group	*Lactobacillus delbrueckii subsp. bulgaricus ND02*
1	1	Terrabacteria group	*Lactobacillus fermentum 3872*
1	1	Terrabacteria group	*Lactobacillus fermentum CECT 5716*
1	1	Terrabacteria group	*Lactobacillus fermentum F-6*
1	1	Terrabacteria group	*Lactobacillus fermentum IFO 3956*

1	1	Terrabacteria group	*Lactobacillus gallinarum*
1	1	Terrabacteria group	*Lactobacillus gasseri 130918*
1	1	Terrabacteria group	*Lactobacillus gasseri ATCC 33323 = JCM 1131*
1	1	Terrabacteria group	*Lactobacillus ginsenosidimutans*
1	1	Terrabacteria group	*Lactobacillus heilongjiangensis*
1	1	Terrabacteria group	*Lactobacillus helveticus*
1	1	Terrabacteria group	*Lactobacillus helveticus*
1	1	Terrabacteria group	*Lactobacillus helveticus*
1	1	Terrabacteria group	*Lactobacillus helveticus CNRZ32*
1	1	Terrabacteria group	*Lactobacillus helveticus DPC 4571*
1	1	Terrabacteria group	*Lactobacillus helveticus H10*
1	1	Terrabacteria group	*Lactobacillus helveticus H9*
1	1	Terrabacteria group	*Lactobacillus helveticus R0052*
1	1	Terrabacteria group	*Lactobacillus hokkaidonensis JCM 18461*
1	1	Terrabacteria group	*Lactobacillus johnsonii DPC 6026*
1	1	Terrabacteria group	*Lactobacillus johnsonii FI9785*
1	1	Terrabacteria group	*Lactobacillus johnsonii N6.2*
1	1	Terrabacteria group	*Lactobacillus johnsonii NCC 533*
1	1	Terrabacteria group	*Lactobacillus kefiranofaciens ZW3*
1	1	Terrabacteria group	*Lactobacillus koreensis*
1	1	Terrabacteria group	*Lactobacillus kunkeei*
1	1	Terrabacteria group	*Lactobacillus mucosae LM1*
2	2	Terrabacteria group	*Lactobacillus oris*
1	1	Terrabacteria group	*Lactobacillus paracasei*
1	1	Terrabacteria group	*Lactobacillus paracasei*
1	1	Terrabacteria group	*Lactobacillus paracasei*
1	1	Terrabacteria group	*Lactobacillus paracasei ATCC 334*
1	1	Terrabacteria group	*Lactobacillus paracasei N1115*
1	1	Terrabacteria group	*Lactobacillus paracasei subsp. paracasei 8700:2*

1	1	Terrabacteria group	*Lactobacillus paracasei subsp. paracasei JCM 8130*
1	1	Terrabacteria group	*Lactobacillus paraplantarum*
1	1	Terrabacteria group	*Lactobacillus plantarum*
1	1	Terrabacteria group	*Lactobacillus plantarum*
1	1	Terrabacteria group	*Lactobacillus plantarum*
1	1	Terrabacteria group	*Lactobacillus plantarum*
1	1	Terrabacteria group	*Lactobacillus plantarum*
1	1	Terrabacteria group	*Lactobacillus plantarum*
1	1	Terrabacteria group	*Lactobacillus plantarum*
1	1	Terrabacteria group	*Lactobacillus plantarum 16*
1	1	Terrabacteria group	*Lactobacillus plantarum DOMLa*
1	1	Terrabacteria group	*Lactobacillus plantarum JDM1*
1	1	Terrabacteria group	*Lactobacillus plantarum subsp. plantarum P-8*
1	1	Terrabacteria group	*Lactobacillus plantarum subsp. plantarum ST-III*
1	1	Terrabacteria group	*Lactobacillus plantarum WCFS1*
1	1	Terrabacteria group	*Lactobacillus plantarum ZJ316*
1	1	Terrabacteria group	*Lactobacillus reuteri*
1	1	Terrabacteria group	*Lactobacillus reuteri DSM 20016*
1	1	Terrabacteria group	*Lactobacillus reuteri I5007*
1	1	Terrabacteria group	*Lactobacillus reuteri JCM 1112*
1	1	Terrabacteria group	*Lactobacillus reuteri SD2112*
1	1	Terrabacteria group	*Lactobacillus reuteri TD1*
1	1	Terrabacteria group	*Lactobacillus rhamnosus*
1	1	Terrabacteria group	*Lactobacillus rhamnosus ATCC 8530*
1	1	Terrabacteria group	*Lactobacillus rhamnosus GG*
1	1	Terrabacteria group	*Lactobacillus rhamnosus GG*
1	1	Terrabacteria group	*Lactobacillus rhamnosus Lc 705*
1	1	Terrabacteria group	*Lactobacillus rhamnosus LOCK900*
1	1	Terrabacteria group	*Lactobacillus rhamnosus LOCK908*

1	1	Terrabacteria group	*Lactobacillus ruminis ATCC 27782*
1	1	Terrabacteria group	*Lactobacillus sakei subsp. sakei 23K*
1	1	Terrabacteria group	*Lactobacillus salivarius*
1	1	Terrabacteria group	*Lactobacillus salivarius CECT 5713*
1	1	Terrabacteria group	*Lactobacillus salivarius str. Ren*
1	1	Terrabacteria group	*Lactobacillus salivarius UCC118*
1	1	Terrabacteria group	*Lactobacillus sanfranciscensis TMW 1.1304*
1	1	Terrabacteria group	*Lactobacillus sp. wkB8*
1	1	Terrabacteria group	*Lactococcus garvieae ATCC 49156*
1	1	Terrabacteria group	*Lactococcus garvieae Lg2*
1	1	Terrabacteria group	*Lactococcus lactis*
1	1	Terrabacteria group	*Lactococcus lactis subsp. cremoris A76*
1	1	Terrabacteria group	*Lactococcus lactis subsp. cremoris KW2*
1	1	Terrabacteria group	*Lactococcus lactis subsp. cremoris MG1363*
1	1	Terrabacteria group	*Lactococcus lactis subsp. cremoris NZ9000*
1	1	Terrabacteria group	*Lactococcus lactis subsp. cremoris SK11*
1	1	Terrabacteria group	*Lactococcus lactis subsp. cremoris UC509.9*
1	1	Terrabacteria group	*Lactococcus lactis subsp. lactis*
1	1	Terrabacteria group	*Lactococcus lactis subsp. lactis CV56*
1	1	Terrabacteria group	*Lactococcus lactis subsp. lactis Il1403*
1	1	Terrabacteria group	*Lactococcus lactis subsp. lactis IO-1*
1	1	Terrabacteria group	*Lactococcus lactis subsp. lactis KF147*
1	1	Terrabacteria group	*Lactococcus lactis subsp. lactis KLDS 4.0325*
1	1	Terrabacteria group	*Lactococcus lactis subsp. lactis NCDO 2118*
1	1	Terrabacteria group	*Lactococcus piscium MKFS47*
1	1	Terrabacteria group	*Leifsonia xyli subsp. cynodontis DSM 46306*
1	1	Terrabacteria group	*Leifsonia xyli subsp. xyli str. CTCB07*
2	1	Terrabacteria group	*Leptolyngbya sp. PCC 7376*
1	1	Terrabacteria group	*Leuconostoc carnosum JB16*

1	1	Terrabacteria group	*Leuconostoc citreum KM20*
1	1	Terrabacteria group	*Leuconostoc gelidum JB7*
1	1	Terrabacteria group	*Leuconostoc gelidum subsp. gasicomitatum KG16-1*
1	1	Terrabacteria group	*Leuconostoc gelidum subsp. gasicomitatum LMG 18811*
1	1	Terrabacteria group	*Leuconostoc kimchii IMSNU 11154*
1	1	Terrabacteria group	*Leuconostoc mesenteroides KFRI-MG*
1	1	Terrabacteria group	*Leuconostoc mesenteroides subsp. dextranicum*
1	1	Terrabacteria group	*Leuconostoc mesenteroides subsp. mesenteroides*
1	1	Terrabacteria group	*Leuconostoc mesenteroides subsp. mesenteroides ATCC 8293*
1	1	Terrabacteria group	*Leuconostoc mesenteroides subsp. mesenteroides J18*
1	1	Terrabacteria group	*Leuconostoc sp. C2*
1	1	Terrabacteria group	*Listeria ivanovii subsp. ivanovii*
1	1	Terrabacteria group	*Listeria ivanovii subsp. ivanovii PAM 55*
1	1	Terrabacteria group	*Listeria ivanovii subsp. londoniensis*
1	1	Terrabacteria group	*Listeria ivanovii subsp. londoniensis*
1	1	Terrabacteria group	*Listeria ivanovii WSLC3009*
1	1	Terrabacteria group	*Listeria monocytogenes*
1	1	Terrabacteria group	*Listeria monocytogenes*
1	1	Terrabacteria group	*Listeria monocytogenes*
1	1	Terrabacteria group	*Listeria monocytogenes*
1	1	Terrabacteria group	*Listeria monocytogenes*
1	1	Terrabacteria group	*Listeria monocytogenes*
1	1	Terrabacteria group	*Listeria monocytogenes*
1	1	Terrabacteria group	*Listeria monocytogenes*
1	1	Terrabacteria group	*Listeria monocytogenes*
1	1	Terrabacteria group	*Listeria monocytogenes*
1	1	Terrabacteria group	*Listeria monocytogenes*
1	1	Terrabacteria group	*Listeria monocytogenes*

1	1	Terrabacteria group	*Listeria monocytogenes*
1	1	Terrabacteria group	*Listeria monocytogenes*
1	1	Terrabacteria group	*Listeria monocytogenes*
1	1	Terrabacteria group	*Listeria monocytogenes*
1	1	Terrabacteria group	*Listeria monocytogenes*
1	1	Terrabacteria group	*Listeria monocytogenes*
1	1	Terrabacteria group	*Listeria monocytogenes*
1	1	Terrabacteria group	*Listeria monocytogenes*
1	1	Terrabacteria group	*Listeria monocytogenes*
1	1	Terrabacteria group	*Listeria monocytogenes*
1	1	Terrabacteria group	*Listeria monocytogenes*
1	1	Terrabacteria group	*Listeria monocytogenes*
1	1	Terrabacteria group	*Listeria monocytogenes*
1	1	Terrabacteria group	*Listeria monocytogenes*
1	1	Terrabacteria group	*Listeria monocytogenes*
1	1	Terrabacteria group	*Listeria monocytogenes*
1	1	Terrabacteria group	*Listeria monocytogenes*
1	1	Terrabacteria group	*Listeria monocytogenes*
1	1	Terrabacteria group	*Listeria monocytogenes*
1	1	Terrabacteria group	*Listeria monocytogenes*
1	1	Terrabacteria group	*Listeria monocytogenes*
1	1	Terrabacteria group	*Listeria monocytogenes*
1	1	Terrabacteria group	*Listeria monocytogenes*
1	1	Terrabacteria group	*Listeria monocytogenes*
1	1	Terrabacteria group	*Listeria monocytogenes*
1	1	Terrabacteria group	*Listeria monocytogenes 07PF0776*
1	1	Terrabacteria group	*Listeria monocytogenes 08-5578*
1	1	Terrabacteria group	*Listeria monocytogenes 08-5923*
1	1	Terrabacteria group	*Listeria monocytogenes 10403S*

1	1	Terrabacteria group	*Listeria monocytogenes 6179*
1	1	Terrabacteria group	*Listeria monocytogenes ATCC 19117*
1	1	Terrabacteria group	*Listeria monocytogenes ATCC 19117*
1	1	Terrabacteria group	*Listeria monocytogenes EGD*
1	1	Terrabacteria group	*Listeria monocytogenes Finland 1998*
1	1	Terrabacteria group	*Listeria monocytogenes FSL R2-561*
1	1	Terrabacteria group	*Listeria monocytogenes HCC23*
1	1	Terrabacteria group	*Listeria monocytogenes J0161*
1	1	Terrabacteria group	*Listeria monocytogenes J1-220*
1	1	Terrabacteria group	*Listeria monocytogenes J1816*
1	1	Terrabacteria group	*Listeria monocytogenes L312*
1	1	Terrabacteria group	*Listeria monocytogenes L99*
1	1	Terrabacteria group	*Listeria monocytogenes M7*
1	1	Terrabacteria group	*Listeria monocytogenes R479a*
1	1	Terrabacteria group	*Listeria monocytogenes serotype 4b str. CLIP 80459*
1	1	Terrabacteria group	*Listeria monocytogenes serotype 4b str. F2365*
1	1	Terrabacteria group	*Listeria monocytogenes serotype 4b str. LL195*
1	1	Terrabacteria group	*Listeria monocytogenes SLCC2372*
1	1	Terrabacteria group	*Listeria monocytogenes SLCC2376*
1	1	Terrabacteria group	*Listeria monocytogenes SLCC2378*
1	1	Terrabacteria group	*Listeria monocytogenes SLCC2479*
1	1	Terrabacteria group	*Listeria monocytogenes SLCC2540*
1	1	Terrabacteria group	*Listeria monocytogenes SLCC2755*
1	1	Terrabacteria group	*Listeria monocytogenes SLCC5850*
1	1	Terrabacteria group	*Listeria monocytogenes SLCC7179*
1	1	Terrabacteria group	*Listeria monocytogenes WSLC1001*
1	1	Terrabacteria group	*Listeria monocytogenes WSLC1042*
1	1	Terrabacteria group	*Listeria seeligeri serovar 1/2b str. SLCC3954*
1	1	Terrabacteria group	*Listeria welshimeri serovar 6b str. SLCC5334*

2	2	Terrabacteria group	*Luteipulveratus mongoliensis*
1	1	Terrabacteria group	*Lysinibacillus fusiformis*
1	1	Terrabacteria group	*Lysinibacillus sphaericus*
1	1	Terrabacteria group	*Lysinibacillus sphaericus*
1	1	Terrabacteria group	*Lysinibacillus varians*
1	1	Terrabacteria group	*Macrococcus caseolyticus JCSC5402*
1	1	Terrabacteria group	*Mageeibacillus indolicus UPII9-5*
1	1	Terrabacteria group	*Mahella australiensis 50-1 BON*
1	1	Terrabacteria group	*Marinithermus hydrothermalis DSM 14884*
1	1	Terrabacteria group	*Megasphaera elsdenii 14-14*
1	1	Terrabacteria group	*Meiothermus ruber DSM 1279*
1	1	Terrabacteria group	*Meiothermus silvanus DSM 9946*
1	1	Terrabacteria group	*Melissococcus plutonius DAT561*
0	0	Terrabacteria group	*Mesoplasma florum L1*
0	0	Terrabacteria group	*Mesoplasma florum W37*
1	1	Terrabacteria group	*Microbacterium sp. CGR1*
1	1	Terrabacteria group	*Microbacterium sp. No. 7*
1	1	Terrabacteria group	*Microbacterium sp. PAMC 28756*
1	1	Terrabacteria group	*Microbacterium testaceum StLB037*
2	1	Terrabacteria group	*Micrococcus luteus NCTC 2665*
2	1	Terrabacteria group	*Microcoleus sp. PCC 7113*
2	1	Terrabacteria group	*Microcystis aeruginosa NIES-2549*
2	1	Terrabacteria group	*Microcystis aeruginosa NIES-843*
2	1	Terrabacteria group	*Microcystis panniformis FACHB-1757*
2	2	Terrabacteria group	*Microlunatus phosphovorus NM-1*
2	2	Terrabacteria group	*Micromonospora aurantiaca ATCC 27029*
2	2	Terrabacteria group	*Micromonospora sp. L5*
1	1	Terrabacteria group	*Mobiluncus curtisii ATCC 43063*
2	2	Terrabacteria group	*Modestobacter marinus*

0	0	Terrabacteria group	*Mollicutes bacterium HR1*
2	2	Terrabacteria group	*Moorella thermoacetica*
2	2	Terrabacteria group	*Moorella thermoacetica*
2	2	Terrabacteria group	*Moorella thermoacetica ATCC 39073*
2	1	Terrabacteria group	*Mycobacterium abscessus*
2	1	Terrabacteria group	*Mycobacterium abscessus*
2	1	Terrabacteria group	*Mycobacterium abscessus subsp. abscessus*
2	1	Terrabacteria group	*Mycobacterium abscessus subsp. abscessus*
2	1	Terrabacteria group	*Mycobacterium abscessus subsp. bolletii*
2	1	Terrabacteria group	*Mycobacterium abscessus subsp. bolletii*
2	1	Terrabacteria group	*Mycobacterium abscessus subsp. bolletii*
2	1	Terrabacteria group	*Mycobacterium abscessus subsp. bolletii 103*
2	1	Terrabacteria group	*Mycobacterium abscessus subsp. bolletii 50594*
2	1	Terrabacteria group	*Mycobacterium abscessus subsp. bolletii CCUG 48898 = JCM 15300*
2	1	Terrabacteria group	*Mycobacterium abscessus subsp. bolletii str. GO 06*
2	1	Terrabacteria group	*Mycobacterium abscessus UC22*
2	1	Terrabacteria group	*Mycobacterium africanum GM041182*
2	1	Terrabacteria group	*Mycobacterium avium 104*
2	1	Terrabacteria group	*Mycobacterium avium subsp. avium*
2	1	Terrabacteria group	*Mycobacterium avium subsp. avium 2285 (R)*
2	1	Terrabacteria group	*Mycobacterium avium subsp. avium 2285 (S)*
2	1	Terrabacteria group	*Mycobacterium avium subsp. hominissuis TH135*
2	1	Terrabacteria group	*Mycobacterium avium subsp. paratuberculosis*
2	1	Terrabacteria group	*Mycobacterium avium subsp. paratuberculosis*
2	1	Terrabacteria group	*Mycobacterium avium subsp. paratuberculosis K-10*
2	1	Terrabacteria group	*Mycobacterium avium subsp. paratuberculosis MAP4*
2	1	Terrabacteria group	*Mycobacterium bovis*
2	1	Terrabacteria group	*Mycobacterium bovis*

2	1	Terrabacteria group	*Mycobacterium bovis*
2	1	Terrabacteria group	*Mycobacterium bovis BCG*
2	1	Terrabacteria group	*Mycobacterium bovis BCG*
2	1	Terrabacteria group	*Mycobacterium bovis BCG str. ATCC 35743*
2	1	Terrabacteria group	*Mycobacterium bovis BCG str. Korea 1168P*
2	1	Terrabacteria group	*Mycobacterium bovis BCG str. Mexico*
2	1	Terrabacteria group	*Mycobacterium bovis BCG str. Moreau RDJ*
2	1	Terrabacteria group	*Mycobacterium bovis BCG str. Pasteur 1173P2*
2	1	Terrabacteria group	*Mycobacterium bovis BCG str. Tokyo 172*
2	1	Terrabacteria group	*Mycobacterium bovis BCG str. Tokyo 172*
2	1	Terrabacteria group	*Mycobacterium canettii CIPT 140010059*
3	1	Terrabacteria group	*Mycobacterium chubuense NBB4*
2	1	Terrabacteria group	*Mycobacterium fortuitum*
2	1	Terrabacteria group	*Mycobacterium gilvum PYR-GCK*
2	1	Terrabacteria group	*Mycobacterium gilvum Spyr1*
3	1	Terrabacteria group	*Mycobacterium goodii*
2	1	Terrabacteria group	*Mycobacterium haemophilum DSM 44634*
2	1	Terrabacteria group	*Mycobacterium immunogenum*
2	1	Terrabacteria group	*Mycobacterium indicus pranii MTCC 9506*
2	1	Terrabacteria group	*Mycobacterium intracellulare 1956*
2	1	Terrabacteria group	*Mycobacterium intracellulare ATCC 13950*
2	1	Terrabacteria group	*Mycobacterium intracellulare MOTT-02*
2	1	Terrabacteria group	*Mycobacterium intracellulare MOTT-64*
2	1	Terrabacteria group	*Mycobacterium kansasii 824*
2	1	Terrabacteria group	*Mycobacterium kansasii ATCC 12478*
2	1	Terrabacteria group	*Mycobacterium leprae Br4923*
2	1	Terrabacteria group	*Mycobacterium liflandii 128FXT*
2	1	Terrabacteria group	*Mycobacterium marinum E11*
2	1	Terrabacteria group	*Mycobacterium marinum M*

2	1	Terrabacteria group	*Mycobacterium neoaurum VKM Ac-1815D*
2	1	Terrabacteria group	*Mycobacterium phlei*
2	1	Terrabacteria group	*Mycobacterium rhodesiae NBB3*
2	1	Terrabacteria group	*Mycobacterium sinense*
3	1	Terrabacteria group	*Mycobacterium smegmatis*
3	1	Terrabacteria group	*Mycobacterium smegmatis*
3	1	Terrabacteria group	*Mycobacterium smegmatis*
3	1	Terrabacteria group	*Mycobacterium smegmatis str. MC2 155*
3	1	Terrabacteria group	*Mycobacterium smegmatis str. MC2 155*
3	1	Terrabacteria group	*Mycobacterium smegmatis str. MC2 155*
2	1	Terrabacteria group	*Mycobacterium sp. EPa45*
2	1	Terrabacteria group	*Mycobacterium sp. JLS*
3	1	Terrabacteria group	*Mycobacterium sp. JS623*
2	1	Terrabacteria group	*Mycobacterium sp. KMS*
2	1	Terrabacteria group	*Mycobacterium sp. MCS*
2	1	Terrabacteria group	*Mycobacterium sp. MOTT36Y*
2	1	Terrabacteria group	*Mycobacterium sp. NRRL B-3805*
2	1	Terrabacteria group	*Mycobacterium sp. VKM Ac-1817D*
2	1	Terrabacteria group	*Mycobacterium tuberculosis*
2	1	Terrabacteria group	*Mycobacterium tuberculosis*
2	1	Terrabacteria group	*Mycobacterium tuberculosis*
2	1	Terrabacteria group	*Mycobacterium tuberculosis*
2	1	Terrabacteria group	*Mycobacterium tuberculosis*
2	1	Terrabacteria group	*Mycobacterium tuberculosis*
2	1	Terrabacteria group	*Mycobacterium tuberculosis*
2	1	Terrabacteria group	*Mycobacterium tuberculosis*
2	1	Terrabacteria group	*Mycobacterium tuberculosis*
2	1	Terrabacteria group	*Mycobacterium tuberculosis*
2	1	Terrabacteria group	*Mycobacterium tuberculosis*

2	1	Terrabacteria group	*Mycobacterium tuberculosis 49-02*
2	1	Terrabacteria group	*Mycobacterium tuberculosis 7199-99*
2	1	Terrabacteria group	*Mycobacterium tuberculosis BT1*
2	1	Terrabacteria group	*Mycobacterium tuberculosis BT2*
2	1	Terrabacteria group	*Mycobacterium tuberculosis CCDC5079*
1	1	Terrabacteria group	*Mycobacterium tuberculosis CCDC5180*
2	1	Terrabacteria group	*Mycobacterium tuberculosis CCDC5180*
2	1	Terrabacteria group	*Mycobacterium tuberculosis CDC1551*
2	1	Terrabacteria group	*Mycobacterium tuberculosis CTRI-2*
2	1	Terrabacteria group	*Mycobacterium tuberculosis EAI5*
2	1	Terrabacteria group	*Mycobacterium tuberculosis EAI5/NITR206*
2	1	Terrabacteria group	*Mycobacterium tuberculosis F11*
2	1	Terrabacteria group	*Mycobacterium tuberculosis H37Ra*
2	1	Terrabacteria group	*Mycobacterium tuberculosis H37Rv*
2	1	Terrabacteria group	*Mycobacterium tuberculosis H37Rv*
2	1	Terrabacteria group	*Mycobacterium tuberculosis H37Rv*
2	1	Terrabacteria group	*Mycobacterium tuberculosis H37RvSiena*
2	1	Terrabacteria group	*Mycobacterium tuberculosis HKBS1*
2	1	Terrabacteria group	*Mycobacterium tuberculosis K*
2	1	Terrabacteria group	*Mycobacterium tuberculosis KZN 1435*
2	1	Terrabacteria group	*Mycobacterium tuberculosis KZN 4207*
2	1	Terrabacteria group	*Mycobacterium tuberculosis KZN 605*
2	1	Terrabacteria group	*Mycobacterium tuberculosis str. Beijing/NITR203*
2	1	Terrabacteria group	*Mycobacterium tuberculosis str. Erdman = ATCC 35801*
2	1	Terrabacteria group	*Mycobacterium tuberculosis str. Haarlem*
2	1	Terrabacteria group	*Mycobacterium tuberculosis str. Kurono*
2	1	Terrabacteria group	*Mycobacterium tuberculosis W-148*
2	1	Terrabacteria group	*Mycobacterium vanbaalenii PYR-1*
2	1	Terrabacteria group	*Mycobacterium yongonense 05-1390*

0	0	Terrabacteria group	*Mycoplasma agalactiae*
0	0	Terrabacteria group	*Mycoplasma agalactiae PG2*
0	0	Terrabacteria group	*Mycoplasma arginini*
0	0	Terrabacteria group	*Mycoplasma arthritidis 158L3-1*
0	0	Terrabacteria group	*Mycoplasma bovis*
0	0	Terrabacteria group	*Mycoplasma bovis CQ-W70*
0	0	Terrabacteria group	*Mycoplasma bovis HB0801*
0	0	Terrabacteria group	*Mycoplasma bovis Hubei-1*
0	0	Terrabacteria group	*Mycoplasma bovis PG45*
0	0	Terrabacteria group	*Mycoplasma bovoculi M165/69*
0	0	Terrabacteria group	*Mycoplasma californicum*
0	0	Terrabacteria group	*Mycoplasma californicum HAZ160_1*
0	0	Terrabacteria group	*Mycoplasma canadense*
0	0	Terrabacteria group	*Mycoplasma canis*
0	0	Terrabacteria group	*Mycoplasma canis PG 14*
0	0	Terrabacteria group	*Mycoplasma capricolum subsp. capricolum ATCC 27343*
0	0	Terrabacteria group	*Mycoplasma capricolum subsp. capripneumoniae*
0	0	Terrabacteria group	*Mycoplasma capricolum subsp. capripneumoniae*
0	0	Terrabacteria group	*Mycoplasma capricolum subsp. capripneumoniae*
0	0	Terrabacteria group	*Mycoplasma capricolum subsp. capripneumoniae 87001*
0	0	Terrabacteria group	*Mycoplasma crocodyli MP145*
0	0	Terrabacteria group	*Mycoplasma cynos C142*
0	0	Terrabacteria group	*Mycoplasma dispar*
0	0	Terrabacteria group	*Mycoplasma fermentans JER*
0	0	Terrabacteria group	*Mycoplasma fermentans M64*
0	0	Terrabacteria group	*Mycoplasma flocculare ATCC 27399*
1	1	Terrabacteria group	*Mycoplasma gallisepticum CA06_2006.052-5-2P*
1	1	Terrabacteria group	*Mycoplasma gallisepticum NC06_2006.080-5-2P*
1	1	Terrabacteria group	*Mycoplasma gallisepticum NC08_2008.031-4-3P*

1	1	Terrabacteria group	*Mycoplasma gallisepticum NC95_13295-2-2P*
1	1	Terrabacteria group	*Mycoplasma gallisepticum NC96_1596-4-2P*
1	1	Terrabacteria group	*Mycoplasma gallisepticum NY01_2001.047-5-1P*
1	1	Terrabacteria group	*Mycoplasma gallisepticum str. F*
1	1	Terrabacteria group	*Mycoplasma gallisepticum str. R(high)*
1	1	Terrabacteria group	*Mycoplasma gallisepticum str. R(low)*
1	1	Terrabacteria group	*Mycoplasma gallisepticum VA94_7994-1-7P*
1	1	Terrabacteria group	*Mycoplasma gallisepticum WI01_2001.043-13-2P*
1	1	Terrabacteria group	*Mycoplasma genitalium G37*
1	1	Terrabacteria group	*Mycoplasma genitalium M2288*
1	1	Terrabacteria group	*Mycoplasma genitalium M2321*
1	1	Terrabacteria group	*Mycoplasma genitalium M6282*
1	1	Terrabacteria group	*Mycoplasma genitalium M6320*
0	0	Terrabacteria group	*Mycoplasma haemocanis str. Illinois*
0	0	Terrabacteria group	*Mycoplasma haemofelis Ohio2*
0	0	Terrabacteria group	*Mycoplasma haemofelis str. Langford 1*
0	0	Terrabacteria group	*Mycoplasma hominis*
0	0	Terrabacteria group	*Mycoplasma hominis*
0	0	Terrabacteria group	*Mycoplasma hominis ATCC 23114*
0	0	Terrabacteria group	*Mycoplasma hominis ATCC 27545*
0	0	Terrabacteria group	*Mycoplasma hyopneumoniae 168*
0	0	Terrabacteria group	*Mycoplasma hyopneumoniae 168-L*
0	0	Terrabacteria group	*Mycoplasma hyopneumoniae 232*
0	0	Terrabacteria group	*Mycoplasma hyopneumoniae 7422*
0	0	Terrabacteria group	*Mycoplasma hyopneumoniae 7448*
0	0	Terrabacteria group	*Mycoplasma hyopneumoniae J*
0	0	Terrabacteria group	*Mycoplasma hyorhinis DBS 1050*
0	0	Terrabacteria group	*Mycoplasma hyorhinis GDL-1*
0	0	Terrabacteria group	*Mycoplasma hyorhinis HUB-1*

0	0	Terrabacteria group	*Mycoplasma hyorhinis MCLD*
0	0	Terrabacteria group	*Mycoplasma hyorhinis SK76*
0	0	Terrabacteria group	*Mycoplasma leachii PG50*
0	0	Terrabacteria group	*Mycoplasma mobile 163K*
0	0	Terrabacteria group	*Mycoplasma mycoides subsp. capri*
0	0	Terrabacteria group	*Mycoplasma mycoides subsp. capri LC str. 95010*
0	0	Terrabacteria group	*Mycoplasma mycoides subsp. capri str. GM12*
0	0	Terrabacteria group	*Mycoplasma mycoides subsp. capri str. GM12*
0	0	Terrabacteria group	*Mycoplasma mycoides subsp. mycoides*
0	0	Terrabacteria group	*Mycoplasma mycoides subsp. mycoides*
0	0	Terrabacteria group	*Mycoplasma mycoides subsp. mycoides*
0	0	Terrabacteria group	*Mycoplasma mycoides subsp. mycoides*
0	0	Terrabacteria group	*Mycoplasma mycoides subsp. mycoides*
0	0	Terrabacteria group	*Mycoplasma mycoides subsp. mycoides SC str. Gladysdale*
0	0	Terrabacteria group	*Mycoplasma mycoides subsp. mycoides SC str. PG1*
0	0	Terrabacteria group	*Mycoplasma ovis str. Michigan*
0	0	Terrabacteria group	*Mycoplasma parvum str. Indiana*
1	1	Terrabacteria group	*Mycoplasma penetrans HF-2*
1	1	Terrabacteria group	*Mycoplasma pneumoniae*
1	1	Terrabacteria group	*Mycoplasma pneumoniae*
1	1	Terrabacteria group	*Mycoplasma pneumoniae*
1	1	Terrabacteria group	*Mycoplasma pneumoniae 19294*
1	1	Terrabacteria group	*Mycoplasma pneumoniae 309*
1	1	Terrabacteria group	*Mycoplasma pneumoniae 39443*
1	1	Terrabacteria group	*Mycoplasma pneumoniae 51494*
1	1	Terrabacteria group	*Mycoplasma pneumoniae 54089*
1	1	Terrabacteria group	*Mycoplasma pneumoniae 54524*
1	1	Terrabacteria group	*Mycoplasma pneumoniae 85084*
1	1	Terrabacteria group	*Mycoplasma pneumoniae 85138*

1	1	Terrabacteria group	*Mycoplasma pneumoniae FH*
1	1	Terrabacteria group	*Mycoplasma pneumoniae FH*
1	1	Terrabacteria group	*Mycoplasma pneumoniae M1139*
1	1	Terrabacteria group	*Mycoplasma pneumoniae M129*
1	1	Terrabacteria group	*Mycoplasma pneumoniae M129-B7*
1	1	Terrabacteria group	*Mycoplasma pneumoniae M2592*
1	1	Terrabacteria group	*Mycoplasma pneumoniae M29*
1	1	Terrabacteria group	*Mycoplasma pneumoniae MAC*
1	1	Terrabacteria group	*Mycoplasma pneumoniae PI 1428*
1	1	Terrabacteria group	*Mycoplasma pneumoniae PO1*
0	0	Terrabacteria group	*Mycoplasma putrefaciens KS1*
0	0	Terrabacteria group	*Mycoplasma putrefaciens Mput9231*
0	0	Terrabacteria group	*Mycoplasma suis KI3806*
0	0	Terrabacteria group	*Mycoplasma suis str. Illinois*
0	0	Terrabacteria group	*Mycoplasma synoviae 53*
0	0	Terrabacteria group	*Mycoplasma synoviae ATCC 25204*
0	0	Terrabacteria group	*Mycoplasma wenyonii str. Massachusetts*
0	0	Terrabacteria group	*Mycoplasma yeatsii GM274B*
2	2	Terrabacteria group	*Nakamurella multipartita DSM 44233*
1	1	Terrabacteria group	*Natranaerobius thermophilus JW/NM-WN-LF*
2	1	Terrabacteria group	*Nocardia brasiliensis ATCC 700358*
2	1	Terrabacteria group	*Nocardia cyriacigeorgica GUH-2*
3	1	Terrabacteria group	*Nocardia farcinica*
2	1	Terrabacteria group	*Nocardia farcinica IFM 10152*
2	2	Terrabacteria group	*Nocardia nova SH22a*
2	2	Terrabacteria group	*Nocardioides sp. JS614*
2	2	Terrabacteria group	*Nocardiopsis alba ATCC BAA-2165*
2	2	Terrabacteria group	*Nocardiopsis dassonvillei subsp. dassonvillei DSM 43111*
2	1	Terrabacteria group	*'Nostoc azollae' 0708*

3	2	Terrabacteria group	*Nostoc punctiforme PCC 73102*
2	1	Terrabacteria group	*Nostoc sp. PCC 7107*
2	1	Terrabacteria group	*Nostoc sp. PCC 7120*
2	1	Terrabacteria group	*Nostoc sp. PCC 7524*
1	1	Terrabacteria group	*Oceanithermus profundus DSM 14977*
1	1	Terrabacteria group	*Oceanobacillus iheyensis HTE831*
1	1	Terrabacteria group	*Oenococcus kitaharae DSM 17330*
1	1	Terrabacteria group	*Oenococcus oeni PSU-1*
1	1	Terrabacteria group	*Olsenella sp. oral taxon 807*
1	1	Terrabacteria group	*Olsenella uli DSM 7084*
1	1	Terrabacteria group	*Onion yellows phytoplasma OY-M*
2	1	Terrabacteria group	*Oscillatoria acuminata PCC 6304*
2	1	Terrabacteria group	*Oscillatoria nigro-viridis PCC 7112*
1	1	Terrabacteria group	*Oscillibacter valericigenes Sjm18-20*
2	1	Terrabacteria group	*Paenarthrobacter aurescens TC1*
1	1	Terrabacteria group	*Paenibacillus beijingensis*
1	1	Terrabacteria group	*Paenibacillus borealis*
1	1	Terrabacteria group	*Paenibacillus bovis*
1	1	Terrabacteria group	*Paenibacillus durus*
1	1	Terrabacteria group	*Paenibacillus durus ATCC 35681*
1	1	Terrabacteria group	*Paenibacillus graminis*
1	1	Terrabacteria group	*Paenibacillus larvae subsp. larvae DSM 25430*
1	2	Terrabacteria group	*Paenibacillus mucilaginosus 3016*
2	2	Terrabacteria group	*Paenibacillus mucilaginosus K02*
2	2	Terrabacteria group	*Paenibacillus mucilaginosus KNP414*
2	2	Terrabacteria group	*Paenibacillus naphthalenovorans*
1	1	Terrabacteria group	*Paenibacillus odorifer*
1	1	Terrabacteria group	*Paenibacillus peoriae*
1	1	Terrabacteria group	*Paenibacillus polymyxa*

1	1	Terrabacteria group	*Paenibacillus polymyxa CR1*
1	1	Terrabacteria group	*Paenibacillus polymyxa E681*
1	1	Terrabacteria group	*Paenibacillus polymyxa M1*
1	1	Terrabacteria group	*Paenibacillus polymyxa SC2*
1	1	Terrabacteria group	*Paenibacillus polymyxa SQR-21*
1	1	Terrabacteria group	*Paenibacillus riograndensis SBR5*
1	1	Terrabacteria group	*Paenibacillus sabinae T27*
1	1	Terrabacteria group	*Paenibacillus sp. 32O-W*
1	1	Terrabacteria group	*Paenibacillus sp. FSL H7-0357*
1	1	Terrabacteria group	*Paenibacillus sp. FSL H7-0737*
1	1	Terrabacteria group	*Paenibacillus sp. FSL P4-0081*
1	1	Terrabacteria group	*Paenibacillus sp. FSL R5-0345*
1	1	Terrabacteria group	*Paenibacillus sp. FSL R5-0912*
1	1	Terrabacteria group	*Paenibacillus sp. FSL R7-0273*
1	1	Terrabacteria group	*Paenibacillus sp. FSL R7-0331*
1	1	Terrabacteria group	*Paenibacillus sp. IHBB 10380*
1	1	Terrabacteria group	*Paenibacillus sp. JDR-2*
1	1	Terrabacteria group	*Paenibacillus sp. Y412MC10*
1	1	Terrabacteria group	*Paenibacillus stellifer*
1	1	Terrabacteria group	*Paenibacillus terrae HPL-003*
1	1	Terrabacteria group	*Parascardovia denticolens DSM 10105 = JCM 12538*
1	1	Terrabacteria group	*Parvimonas micra*
1	1	Terrabacteria group	*Pediococcus claussenii ATCC BAA-344*
1	1	Terrabacteria group	*Pediococcus pentosaceus ATCC 25745*
1	1	Terrabacteria group	*Pediococcus pentosaceus SL4*
1	1	Terrabacteria group	*Pelosinus fermentans JBW45*
1	1	Terrabacteria group	*Pelosinus sp. UFO1*
1	1	Terrabacteria group	*Peptoclostridium difficile*
1	1	Terrabacteria group	*Peptoclostridium difficile*

1	1	Terrabacteria group	*Peptoclostridium difficile*
1	1	Terrabacteria group	*Peptoclostridium difficile 630*
1	1	Terrabacteria group	*Peptoclostridium difficile 630*
1	1	Terrabacteria group	*Peptoclostridium difficile CD196*
1	1	Terrabacteria group	*Peptoclostridium difficile M120*
1	1	Terrabacteria group	*Peptoniphilus sp. 1-1*
2	1	Terrabacteria group	*Pimelobacter simplex*
1	1	Terrabacteria group	*Planococcus kocurii*
1	1	Terrabacteria group	*Planococcus sp. PAMC 21323*
2	1	Terrabacteria group	*Pleurocapsa sp. PCC 7327*
2	1	Terrabacteria group	*Prochlorococcus marinus str. AS9601*
2	1	Terrabacteria group	*Prochlorococcus marinus str. MIT 9215*
2	1	Terrabacteria group	*Prochlorococcus marinus str. MIT 9301*
2	1	Terrabacteria group	*Prochlorococcus marinus str. MIT 9312*
2	1	Terrabacteria group	*Prochlorococcus marinus str. MIT 9313*
2	1	Terrabacteria group	*Prochlorococcus marinus str. MIT 9515*
2	1	Terrabacteria group	*Prochlorococcus marinus str. NATL1A*
2	1	Terrabacteria group	*Prochlorococcus marinus str. NATL2A*
2	1	Terrabacteria group	*Prochlorococcus marinus subsp. marinus str. CCMP1375*
2	1	Terrabacteria group	*Prochlorococcus marinus subsp. pastoris str. CCMP1986*
2	1	Terrabacteria group	*Prochlorococcus sp. MIT 0604*
2	1	Terrabacteria group	*Prochlorococcus sp. MIT 0801*
2	2	Terrabacteria group	*Propionibacterium acidipropionici*
2	2	Terrabacteria group	*Propionibacterium acidipropionici*
2	2	Terrabacteria group	*Propionibacterium acidipropionici ATCC 4875*
2	1	Terrabacteria group	*Propionibacterium acnes*
2	1	Terrabacteria group	*Propionibacterium acnes*
2	1	Terrabacteria group	*Propionibacterium acnes*
2	1	Terrabacteria group	*Propionibacterium acnes*

2	1	Terrabacteria group	*Propionibacterium acnes*
2	1	Terrabacteria group	*Propionibacterium acnes 266*
2	1	Terrabacteria group	*Propionibacterium acnes 6609*
2	1	Terrabacteria group	*Propionibacterium acnes ATCC 11828*
2	1	Terrabacteria group	*Propionibacterium acnes C1*
2	1	Terrabacteria group	*Propionibacterium acnes hdn-1*
2	1	Terrabacteria group	*Propionibacterium acnes HL096PA1*
2	1	Terrabacteria group	*Propionibacterium acnes KPA171202*
2	1	Terrabacteria group	*Propionibacterium acnes SK137*
2	1	Terrabacteria group	*Propionibacterium acnes TypeIA2 P.acn17*
2	1	Terrabacteria group	*Propionibacterium acnes TypeIA2 P.acn31*
2	1	Terrabacteria group	*Propionibacterium acnes TypeIA2 P.acn33*
2	1	Terrabacteria group	*Propionibacterium avidum 44067*
2	2	Terrabacteria group	*Propionibacterium freudenreichii subsp. freudenreichii*
2	2	Terrabacteria group	*Propionibacterium freudenreichii subsp. shermanii CIRM-BIA1*
2	1	Terrabacteria group	*Propionibacterium propionicum F0230a*
2	2	Terrabacteria group	*Pseudanabaena sp. PCC 7367*
2	1	Terrabacteria group	*Pseudarthrobacter chlorophenolicus A6*
2	1	Terrabacteria group	*Pseudarthrobacter phenanthrenivorans Sphe3*
2	1	Terrabacteria group	*Pseudarthrobacter sulfonivorans*
3	2	Terrabacteria group	*Pseudonocardia dioxanivorans CB1190*
3	2	Terrabacteria group	*Pseudonocardia sp. AL041005-10*
4	2	Terrabacteria group	*Pseudonocardia sp. EC080610-09*
4	2	Terrabacteria group	*Pseudonocardia sp. EC080619-01*
3	2	Terrabacteria group	*Pseudonocardia sp. EC080625-04*
4	2	Terrabacteria group	*Pseudonocardia sp. HH130629-09*
1	1	Terrabacteria group	*Rathayibacter toxicus*
1	1	Terrabacteria group	*Rathayibacter toxicus*

2	1	Terrabacteria group	*Renibacterium salmoninarum ATCC 33209*
3	1	Terrabacteria group	*Rhodococcus aetherivorans*
2	1	Terrabacteria group	*Rhodococcus equi 103S*
2	1	Terrabacteria group	*Rhodococcus erythropolis*
2	1	Terrabacteria group	*Rhodococcus erythropolis CCM2595*
2	1	Terrabacteria group	*Rhodococcus erythropolis PR4*
2	1	Terrabacteria group	*Rhodococcus erythropolis R138*
3	1	Terrabacteria group	*Rhodococcus jostii RHA1*
2	1	Terrabacteria group	*Rhodococcus opacus B4*
3	1	Terrabacteria group	*Rhodococcus opacus PD630*
3	1	Terrabacteria group	*Rhodococcus pyridinivorans SB3094*
2	1	Terrabacteria group	*Rhodococcus sp. B7740*
1	1	Terrabacteria group	*Rhodoluna lacicola*
2	1	Terrabacteria group	*Rivularia sp. PCC 7116*
1	1	Terrabacteria group	*Roseburia hominis A2-183*
2	2	Terrabacteria group	*Roseiflexus castenholzii DSM 13941*
2	4	Terrabacteria group	*Roseiflexus sp. RS-1*
2	1	Terrabacteria group	*Rothia dentocariosa ATCC 17931*
2	1	Terrabacteria group	*Rothia mucilaginosa*
2	1	Terrabacteria group	*Rothia mucilaginosa DY-18*
1	1	Terrabacteria group	*Rubrobacter radiotolerans*
1	1	Terrabacteria group	*Rubrobacter xylanophilus DSM 9941*
1	1	Terrabacteria group	*Ruminiclostridium thermocellum AD2*
1	1	Terrabacteria group	*Ruminiclostridium thermocellum ATCC 27405*
1	1	Terrabacteria group	*Ruminiclostridium thermocellum DSM 1313*
1	1	Terrabacteria group	*Ruminococcus albus 7 = DSM 20455*
1	1	Terrabacteria group	*Ruminococcus bicirculans*
2	2	Terrabacteria group	*Saccharomonospora viridis DSM 43017*
2	2	Terrabacteria group	*Saccharopolyspora erythraea NRRL 2338*

2	2	Terrabacteria group	*Saccharothrix espanaensis DSM 44229*
1	1	Terrabacteria group	*Salinicoccus halodurans*
2	2	Terrabacteria group	*Salinispora arenicola CNS-205*
2	2	Terrabacteria group	*Salinispora tropica CNB-440*
1	1	Terrabacteria group	*Sanguibacter keddieii DSM 10542*
1	1	Terrabacteria group	*Scardovia inopinata JCM 12537*
2	1	Terrabacteria group	*Segniliparus rotundus DSM 44985*
1	1	Terrabacteria group	*Selenomonas ruminantium subsp. lactilytica TAM6421*
1	1	Terrabacteria group	*Selenomonas sp. oral taxon 136*
1	1	Terrabacteria group	*Selenomonas sp. oral taxon 478*
1	1	Terrabacteria group	*Selenomonas sputigena ATCC 35185*
2	1	Terrabacteria group	*Slackia heliotrinireducens DSM 20476*
1	1	Terrabacteria group	*Solibacillus silvestris*
1	1	Terrabacteria group	*Solibacillus silvestris StLB046*
1	1	Terrabacteria group	*Sphaerobacter thermophilus DSM 20745*
0	0	Terrabacteria group	*Spiroplasma apis B31*
0	0	Terrabacteria group	*Spiroplasma atrichopogonis*
0	0	Terrabacteria group	*Spiroplasma cantharicola*
0	0	Terrabacteria group	*Spiroplasma chrysopicola DF-1*
0	0	Terrabacteria group	*Spiroplasma culicicola AES-1*
0	0	Terrabacteria group	*Spiroplasma diminutum CUAS-1*
0	0	Terrabacteria group	*Spiroplasma eriocheiris CCTCC M 207170*
1	1	Terrabacteria group	*Spiroplasma kunkelii CR2-3x*
0	0	Terrabacteria group	*Spiroplasma mirum ATCC 29335*
0	0	Terrabacteria group	*Spiroplasma mirum ATCC 29335*
0	0	Terrabacteria group	*Spiroplasma sabaudiense Ar-1343*
0	0	Terrabacteria group	*Spiroplasma syrphidicola EA-1*
0	0	Terrabacteria group	*Spiroplasma taiwanense CT-1*
2	2	Terrabacteria group	*Stackebrandtia nassauensis DSM 44728*

2	1	Terrabacteria group	*Stanieria cyanosphaera PCC 7437*
1	1	Terrabacteria group	*Staphylococcus agnetis*
1	1	Terrabacteria group	*Staphylococcus argenteus*
0	0	Terrabacteria group	*Staphylococcus aureus*
1	1	Terrabacteria group	*Staphylococcus aureus*
1	1	Terrabacteria group	*Staphylococcus aureus*
1	1	Terrabacteria group	*Staphylococcus aureus*
1	1	Terrabacteria group	*Staphylococcus aureus*
1	1	Terrabacteria group	*Staphylococcus aureus*
1	1	Terrabacteria group	*Staphylococcus aureus*
1	1	Terrabacteria group	*Staphylococcus aureus*
1	1	Terrabacteria group	*Staphylococcus aureus*
1	1	Terrabacteria group	*Staphylococcus aureus*
1	1	Terrabacteria group	*Staphylococcus aureus*
1	1	Terrabacteria group	*Staphylococcus aureus*
1	1	Terrabacteria group	*Staphylococcus aureus*
1	1	Terrabacteria group	*Staphylococcus aureus*
1	1	Terrabacteria group	*Staphylococcus aureus*
1	1	Terrabacteria group	*Staphylococcus aureus*
1	1	Terrabacteria group	*Staphylococcus aureus*
1	1	Terrabacteria group	*Staphylococcus aureus*
1	1	Terrabacteria group	*Staphylococcus aureus*
1	1	Terrabacteria group	*Staphylococcus aureus*
1	1	Terrabacteria group	*Staphylococcus aureus*
1	1	Terrabacteria group	*Staphylococcus aureus*
1	1	Terrabacteria group	*Staphylococcus aureus*
1	1	Terrabacteria group	*Staphylococcus aureus*
1	1	Terrabacteria group	*Staphylococcus aureus*
1	1	Terrabacteria group	*Staphylococcus aureus*
1	1	Terrabacteria group	*Staphylococcus aureus*

1	1	Terrabacteria group	*Staphylococcus aureus*
1	1	Terrabacteria group	*Staphylococcus aureus*
1	1	Terrabacteria group	*Staphylococcus aureus*
1	1	Terrabacteria group	*Staphylococcus aureus*
1	1	Terrabacteria group	*Staphylococcus aureus*
1	1	Terrabacteria group	*Staphylococcus aureus*
1	1	Terrabacteria group	*Staphylococcus aureus*
1	1	Terrabacteria group	*Staphylococcus aureus*
1	1	Terrabacteria group	*Staphylococcus aureus*
1	1	Terrabacteria group	*Staphylococcus aureus 04-02981*
1	1	Terrabacteria group	*Staphylococcus aureus 08BA02176*
1	1	Terrabacteria group	*Staphylococcus aureus Bmb9393*
1	1	Terrabacteria group	*Staphylococcus aureus CA-347*
1	1	Terrabacteria group	*Staphylococcus aureus RF122*
1	1	Terrabacteria group	*Staphylococcus aureus subsp. aureus*
1	1	Terrabacteria group	*Staphylococcus aureus subsp. aureus*
1	1	Terrabacteria group	*Staphylococcus aureus subsp. aureus*
1	1	Terrabacteria group	*Staphylococcus aureus subsp. aureus*
1	1	Terrabacteria group	*Staphylococcus aureus subsp. aureus*
1	1	Terrabacteria group	*Staphylococcus aureus subsp. aureus*
1	1	Terrabacteria group	*Staphylococcus aureus subsp. aureus*
1	1	Terrabacteria group	*Staphylococcus aureus subsp. aureus*
1	1	Terrabacteria group	*Staphylococcus aureus subsp. aureus*
1	1	Terrabacteria group	*Staphylococcus aureus subsp. aureus*
1	1	Terrabacteria group	*Staphylococcus aureus subsp. aureus*
1	1	Terrabacteria group	*Staphylococcus aureus subsp. aureus*
1	1	Terrabacteria group	*Staphylococcus aureus subsp. aureus*
1	1	Terrabacteria group	*Staphylococcus aureus subsp. aureus*
1	1	Terrabacteria group	*Staphylococcus aureus subsp. aureus*

1	1	Terrabacteria group	*Staphylococcus aureus subsp. aureus 11819-97*
1	1	Terrabacteria group	*Staphylococcus aureus subsp. aureus 55/2053*
1	1	Terrabacteria group	*Staphylococcus aureus subsp. aureus 6850*
1	1	Terrabacteria group	*Staphylococcus aureus subsp. aureus CN1*
1	1	Terrabacteria group	*Staphylococcus aureus subsp. aureus COL*
1	1	Terrabacteria group	*Staphylococcus aureus subsp. aureus DSM 20231*
1	1	Terrabacteria group	*Staphylococcus aureus subsp. aureus ECT-R 2*
1	1	Terrabacteria group	*Staphylococcus aureus subsp. aureus ED133*
1	1	Terrabacteria group	*Staphylococcus aureus subsp. aureus ED98*
1	1	Terrabacteria group	*Staphylococcus aureus subsp. aureus HO 5096 0412*
1	1	Terrabacteria group	*Staphylococcus aureus subsp. aureus JH1*
1	1	Terrabacteria group	*Staphylococcus aureus subsp. aureus JH9*
1	1	Terrabacteria group	*Staphylococcus aureus subsp. aureus JKD6159*
1	1	Terrabacteria group	*Staphylococcus aureus subsp. aureus LGA251*
1	1	Terrabacteria group	*Staphylococcus aureus subsp. aureus M013*
1	1	Terrabacteria group	*Staphylococcus aureus subsp. aureus MRSA252*
1	1	Terrabacteria group	*Staphylococcus aureus subsp. aureus MSSA476*
1	1	Terrabacteria group	*Staphylococcus aureus subsp. aureus Mu3*
1	1	Terrabacteria group	*Staphylococcus aureus subsp. aureus Mu50*
1	1	Terrabacteria group	*Staphylococcus aureus subsp. aureus MW2*
1	1	Terrabacteria group	*Staphylococcus aureus subsp. aureus N315*
1	1	Terrabacteria group	*Staphylococcus aureus subsp. aureus NCTC 8325*
1	1	Terrabacteria group	*Staphylococcus aureus subsp. aureus SA268*
1	1	Terrabacteria group	*Staphylococcus aureus subsp. aureus SA40*
1	1	Terrabacteria group	*Staphylococcus aureus subsp. aureus SA957*
1	1	Terrabacteria group	*Staphylococcus aureus subsp. aureus ST228*
1	1	Terrabacteria group	*Staphylococcus aureus subsp. aureus ST228*
1	1	Terrabacteria group	*Staphylococcus aureus subsp. aureus ST228*
1	1	Terrabacteria group	*Staphylococcus aureus subsp. aureus ST228*

1	1	Terrabacteria group	*Staphylococcus aureus subsp. aureus ST228*
1	1	Terrabacteria group	*Staphylococcus aureus subsp. aureus ST228*
1	1	Terrabacteria group	*Staphylococcus aureus subsp. aureus ST228*
1	1	Terrabacteria group	*Staphylococcus aureus subsp. aureus ST228*
1	1	Terrabacteria group	*Staphylococcus aureus subsp. aureus ST398*
1	1	Terrabacteria group	*Staphylococcus aureus subsp. aureus ST772-MRSA-V*
1	1	Terrabacteria group	*Staphylococcus aureus subsp. aureus str. JKD6008*
1	1	Terrabacteria group	*Staphylococcus aureus subsp. aureus str. Newman*
1	1	Terrabacteria group	*Staphylococcus aureus subsp. aureus T0131*
1	1	Terrabacteria group	*Staphylococcus aureus subsp. aureus Tager 104*
1	1	Terrabacteria group	*Staphylococcus aureus subsp. aureus TCH60*
1	1	Terrabacteria group	*Staphylococcus aureus subsp. aureus TW20*
1	1	Terrabacteria group	*Staphylococcus aureus subsp. aureus USA300_FPR3757*
1	1	Terrabacteria group	*Staphylococcus aureus subsp. aureus USA300_TCH1516*
1	1	Terrabacteria group	*Staphylococcus aureus subsp. aureus VC40*
1	1	Terrabacteria group	*Staphylococcus aureus subsp. aureus Z172*
1	1	Terrabacteria group	*Staphylococcus aureus USA300-ISMMS1*
1	1	Terrabacteria group	*Staphylococcus capitis subsp. capitis*
1	1	Terrabacteria group	*Staphylococcus carnosus subsp. carnosus TM300*
1	1	Terrabacteria group	*Staphylococcus epidermidis*
1	1	Terrabacteria group	*Staphylococcus epidermidis ATCC 12228*
1	1	Terrabacteria group	*Staphylococcus epidermidis PM221*
1	1	Terrabacteria group	*Staphylococcus epidermidis RP62A*
1	1	Terrabacteria group	*Staphylococcus equorum*
1	1	Terrabacteria group	*Staphylococcus haemolyticus JCSC1435*
1	1	Terrabacteria group	*Staphylococcus hyicus*
1	1	Terrabacteria group	*Staphylococcus lugdunensis*
1	1	Terrabacteria group	*Staphylococcus lugdunensis*
1	1	Terrabacteria group	*Staphylococcus lugdunensis HKU09-01*

1	1	Terrabacteria group	*Staphylococcus lugdunensis N920143*
1	1	Terrabacteria group	*Staphylococcus pasteuri SP1*
1	1	Terrabacteria group	*Staphylococcus pseudintermedius E140*
1	1	Terrabacteria group	*Staphylococcus pseudintermedius ED99*
1	1	Terrabacteria group	*Staphylococcus pseudintermedius HKU10-03*
1	1	Terrabacteria group	*Staphylococcus saprophyticus*
1	1	Terrabacteria group	*Staphylococcus saprophyticus*
1	1	Terrabacteria group	*Staphylococcus saprophyticus subsp. saprophyticus ATCC 15305*
1	1	Terrabacteria group	*Staphylococcus schleiferi*
1	1	Terrabacteria group	*Staphylococcus schleiferi*
1	1	Terrabacteria group	*Staphylococcus schleiferi*
1	1	Terrabacteria group	*Staphylococcus schleiferi*
1	1	Terrabacteria group	*Staphylococcus schleiferi*
1	1	Terrabacteria group	*Staphylococcus simulans*
1	1	Terrabacteria group	*Staphylococcus warneri SG1*
1	1	Terrabacteria group	*Staphylococcus xylosus*
1	1	Terrabacteria group	*Staphylococcus xylosus*
1	1	Terrabacteria group	*Staphylococcus xylosus*
1	1	Terrabacteria group	*Strawberry lethal yellows phytoplasma (CPA) str. NZSb11*
1	1	Terrabacteria group	*Streptococcus agalactiae*
1	1	Terrabacteria group	*Streptococcus agalactiae*
1	1	Terrabacteria group	*Streptococcus agalactiae*
1	1	Terrabacteria group	*Streptococcus agalactiae*
1	1	Terrabacteria group	*Streptococcus agalactiae*
1	1	Terrabacteria group	*Streptococcus agalactiae*
1	1	Terrabacteria group	*Streptococcus agalactiae*
1	1	Terrabacteria group	*Streptococcus agalactiae*
1	1	Terrabacteria group	*Streptococcus agalactiae*

1	1	Terrabacteria group	*Streptococcus agalactiae*
1	1	Terrabacteria group	*Streptococcus agalactiae*
1	1	Terrabacteria group	*Streptococcus agalactiae*
1	1	Terrabacteria group	*Streptococcus agalactiae*
1	1	Terrabacteria group	*Streptococcus agalactiae*
1	1	Terrabacteria group	*Streptococcus agalactiae 09mas018883*
1	1	Terrabacteria group	*Streptococcus agalactiae 2603V/R*
1	1	Terrabacteria group	*Streptococcus agalactiae A909*
1	1	Terrabacteria group	*Streptococcus agalactiae CNCTC 10/84*
1	1	Terrabacteria group	*Streptococcus agalactiae COH1*
1	1	Terrabacteria group	*Streptococcus agalactiae GD201008-001*
1	1	Terrabacteria group	*Streptococcus agalactiae ILRI005*
1	1	Terrabacteria group	*Streptococcus agalactiae SA20-06*
1	1	Terrabacteria group	*Streptococcus anginosus*
1	1	Terrabacteria group	*Streptococcus anginosus*
1	1	Terrabacteria group	*Streptococcus anginosus C1051*
1	1	Terrabacteria group	*Streptococcus anginosus C238*
1	1	Terrabacteria group	*Streptococcus anginosus subsp. whileyi MAS624*
1	1	Terrabacteria group	*Streptococcus constellatus subsp. pharyngis C1050*
1	1	Terrabacteria group	*Streptococcus constellatus subsp. pharyngis C232*
1	1	Terrabacteria group	*Streptococcus constellatus subsp. pharyngis C818*
1	1	Terrabacteria group	*Streptococcus dysgalactiae subsp. equisimilis 167*
1	1	Terrabacteria group	*Streptococcus dysgalactiae subsp. equisimilis AC-2713*
1	1	Terrabacteria group	*Streptococcus dysgalactiae subsp. equisimilis ATCC 12394*
1	1	Terrabacteria group	*Streptococcus dysgalactiae subsp. equisimilis GGS_124*
1	1	Terrabacteria group	*Streptococcus dysgalactiae subsp. equisimilis RE378*
1	1	Terrabacteria group	*Streptococcus equi subsp. equi 4047*
1	1	Terrabacteria group	*Streptococcus equi subsp. zooepidemicus*
1	1	Terrabacteria group	*Streptococcus equi subsp. zooepidemicus ATCC 35246*

1	1	Terrabacteria group	*Streptococcus equi subsp. zooepidemicus CY*
1	1	Terrabacteria group	*Streptococcus equi subsp. zooepidemicus MGCS10565*
1	1	Terrabacteria group	*Streptococcus gallolyticus*
1	1	Terrabacteria group	*Streptococcus gallolyticus subsp. gallolyticus ATCC 43143*
1	1	Terrabacteria group	*Streptococcus gallolyticus subsp. gallolyticus ATCC BAA-2069*
1	1	Terrabacteria group	*Streptococcus gallolyticus UCN34*
1	1	Terrabacteria group	*Streptococcus gordonii*
1	1	Terrabacteria group	*Streptococcus gordonii str. Challis substr. CH1*
1	1	Terrabacteria group	*Streptococcus infantarius*
1	1	Terrabacteria group	*Streptococcus infantarius subsp. infantarius CJ18*
1	1	Terrabacteria group	*Streptococcus iniae*
1	1	Terrabacteria group	*Streptococcus intermedius B196*
1	1	Terrabacteria group	*Streptococcus intermedius C270*
1	1	Terrabacteria group	*Streptococcus intermedius JTH08*
1	1	Terrabacteria group	*Streptococcus lutetiensis 033*
1	1	Terrabacteria group	*Streptococcus macedonicus ACA-DC 198*
1	1	Terrabacteria group	*Streptococcus mitis*
1	1	Terrabacteria group	*Streptococcus mitis*
1	1	Terrabacteria group	*Streptococcus mitis B6*
1	1	Terrabacteria group	*Streptococcus mutans*
1	1	Terrabacteria group	*Streptococcus mutans GS-5*
1	1	Terrabacteria group	*Streptococcus mutans LJ23*
1	1	Terrabacteria group	*Streptococcus mutans NN2025*
1	1	Terrabacteria group	*Streptococcus mutans UA159*
1	1	Terrabacteria group	*Streptococcus mutans UA159-FR*
1	1	Terrabacteria group	*Streptococcus oligofermentans AS 1.3089*
1	1	Terrabacteria group	*Streptococcus oralis Uo5*
1	1	Terrabacteria group	*Streptococcus parasanguinis ATCC 15912*

1	1	Terrabacteria group	*Streptococcus parasanguinis FW213*
1	1	Terrabacteria group	*Streptococcus parauberis KCTC 11537*
1	1	Terrabacteria group	*Streptococcus parauberis NCFD 2020*
1	1	Terrabacteria group	*Streptococcus pasteurianus ATCC 43144*
1	1	Terrabacteria group	*Streptococcus pneumoniae*
1	1	Terrabacteria group	*Streptococcus pneumoniae*
1	1	Terrabacteria group	*Streptococcus pneumoniae*
1	1	Terrabacteria group	*Streptococcus pneumoniae 670-6B*
1	1	Terrabacteria group	*Streptococcus pneumoniae 70585*
1	1	Terrabacteria group	*Streptococcus pneumoniae AP200*
1	1	Terrabacteria group	*Streptococcus pneumoniae ATCC 700669*
1	1	Terrabacteria group	*Streptococcus pneumoniae CGSP14*
1	1	Terrabacteria group	*Streptococcus pneumoniae D39*
1	1	Terrabacteria group	*Streptococcus pneumoniae G54*
1	1	Terrabacteria group	*Streptococcus pneumoniae gamPNI0373*
1	1	Terrabacteria group	*Streptococcus pneumoniae Hungary19A-6*
1	1	Terrabacteria group	*Streptococcus pneumoniae INV104*
1	1	Terrabacteria group	*Streptococcus pneumoniae INV200*
1	1	Terrabacteria group	*Streptococcus pneumoniae JJA*
1	1	Terrabacteria group	*Streptococcus pneumoniae OXC141*
1	1	Terrabacteria group	*Streptococcus pneumoniae P1031*
1	1	Terrabacteria group	*Streptococcus pneumoniae PCS8235*
1	1	Terrabacteria group	*Streptococcus pneumoniae R6*
1	1	Terrabacteria group	*Streptococcus pneumoniae SPN032672*
1	1	Terrabacteria group	*Streptococcus pneumoniae SPN033038*
1	1	Terrabacteria group	*Streptococcus pneumoniae SPN034156*
1	1	Terrabacteria group	*Streptococcus pneumoniae SPN034183*
1	1	Terrabacteria group	*Streptococcus pneumoniae SPN994038*
1	1	Terrabacteria group	*Streptococcus pneumoniae SPN994039*

1	1	Terrabacteria group	*Streptococcus pneumoniae SPNA45*
1	1	Terrabacteria group	*Streptococcus pneumoniae ST556*
1	1	Terrabacteria group	*Streptococcus pneumoniae Taiwan19F-14*
1	1	Terrabacteria group	*Streptococcus pneumoniae TCH8431/19A*
1	1	Terrabacteria group	*Streptococcus pneumoniae TIGR4*
1	1	Terrabacteria group	*Streptococcus pneumoniae TIGR4*
1	1	Terrabacteria group	*Streptococcus pseudopneumoniae IS7493*
1	1	Terrabacteria group	*Streptococcus pyogenes*
1	1	Terrabacteria group	*Streptococcus pyogenes*
1	1	Terrabacteria group	*Streptococcus pyogenes*
1	1	Terrabacteria group	*Streptococcus pyogenes*
1	1	Terrabacteria group	*Streptococcus pyogenes*
1	1	Terrabacteria group	*Streptococcus pyogenes*
1	1	Terrabacteria group	*Streptococcus pyogenes*
1	1	Terrabacteria group	*Streptococcus pyogenes*
1	1	Terrabacteria group	*Streptococcus pyogenes*
1	1	Terrabacteria group	*Streptococcus pyogenes*
1	1	Terrabacteria group	*Streptococcus pyogenes*
1	1	Terrabacteria group	*Streptococcus pyogenes*
1	1	Terrabacteria group	*Streptococcus pyogenes*
1	1	Terrabacteria group	*Streptococcus pyogenes*
1	1	Terrabacteria group	*Streptococcus pyogenes*
1	1	Terrabacteria group	*Streptococcus pyogenes*
1	1	Terrabacteria group	*Streptococcus pyogenes*
1	1	Terrabacteria group	*Streptococcus pyogenes*
1	1	Terrabacteria group	*Streptococcus pyogenes*
1	1	Terrabacteria group	*Streptococcus pyogenes*
1	1	Terrabacteria group	*Streptococcus pyogenes*
1	1	Terrabacteria group	*Streptococcus pyogenes A20*

1	1	Terrabacteria group	*Streptococcus pyogenes Alab49*
1	1	Terrabacteria group	*Streptococcus pyogenes HKU QMH11M0907901*
1	1	Terrabacteria group	*Streptococcus pyogenes HSC5*
1	1	Terrabacteria group	*Streptococcus pyogenes JRS4*
1	1	Terrabacteria group	*Streptococcus pyogenes JRS4*
1	1	Terrabacteria group	*Streptococcus pyogenes M1 476*
1	1	Terrabacteria group	*Streptococcus pyogenes M1 GAS*
1	1	Terrabacteria group	*Streptococcus pyogenes MGAS10270*
1	1	Terrabacteria group	*Streptococcus pyogenes MGAS10394*
1	1	Terrabacteria group	*Streptococcus pyogenes MGAS10750*
1	1	Terrabacteria group	*Streptococcus pyogenes MGAS15252*
1	1	Terrabacteria group	*Streptococcus pyogenes MGAS1882*
1	1	Terrabacteria group	*Streptococcus pyogenes MGAS2096*
1	1	Terrabacteria group	*Streptococcus pyogenes MGAS315*
1	1	Terrabacteria group	*Streptococcus pyogenes MGAS5005*
1	1	Terrabacteria group	*Streptococcus pyogenes MGAS6180*
1	1	Terrabacteria group	*Streptococcus pyogenes MGAS8232*
1	1	Terrabacteria group	*Streptococcus pyogenes MGAS9429*
1	1	Terrabacteria group	*Streptococcus pyogenes NZ131*
1	1	Terrabacteria group	*Streptococcus pyogenes SSI-1*
1	1	Terrabacteria group	*Streptococcus pyogenes STAB902*
1	1	Terrabacteria group	*Streptococcus pyogenes str. Manfredo*
1	0	Terrabacteria group	*Streptococcus salivarius*
1	1	Terrabacteria group	*Streptococcus salivarius*
1	1	Terrabacteria group	*Streptococcus salivarius*
1	1	Terrabacteria group	*Streptococcus salivarius CCHSS3*
1	1	Terrabacteria group	*Streptococcus salivarius JIM8777*
1	1	Terrabacteria group	*Streptococcus sanguinis SK36*
1	1	Terrabacteria group	*Streptococcus sp. A12*

1	1	Terrabacteria group	*Streptococcus sp. HTS9*
1	1	Terrabacteria group	*Streptococcus sp. I-G2*
1	1	Terrabacteria group	*Streptococcus sp. I-P16*
1	1	Terrabacteria group	*Streptococcus sp. oral taxon 431*
1	1	Terrabacteria group	*Streptococcus sp. VT 162*
1	1	Terrabacteria group	*Streptococcus suis*
1	1	Terrabacteria group	*Streptococcus suis*
1	1	Terrabacteria group	*Streptococcus suis*
1	1	Terrabacteria group	*Streptococcus suis 05HAS68*
1	1	Terrabacteria group	*Streptococcus suis 6407*
1	1	Terrabacteria group	*Streptococcus suis A7*
1	1	Terrabacteria group	*Streptococcus suis BM407*
1	1	Terrabacteria group	*Streptococcus suis D12*
1	1	Terrabacteria group	*Streptococcus suis D9*
1	1	Terrabacteria group	*Streptococcus suis GZ1*
1	1	Terrabacteria group	*Streptococcus suis JS14*
1	1	Terrabacteria group	*Streptococcus suis P1/7*
1	1	Terrabacteria group	*Streptococcus suis S735*
1	1	Terrabacteria group	*Streptococcus suis SC070731*
1	1	Terrabacteria group	*Streptococcus suis SC84*
1	1	Terrabacteria group	*Streptococcus suis SS12*
1	1	Terrabacteria group	*Streptococcus suis ST1*
1	1	Terrabacteria group	*Streptococcus suis ST3*
1	1	Terrabacteria group	*Streptococcus suis T15*
1	1	Terrabacteria group	*Streptococcus suis TL13*
1	1	Terrabacteria group	*Streptococcus suis YB51*
1	1	Terrabacteria group	*Streptococcus thermophilus*
1	1	Terrabacteria group	*Streptococcus thermophilus*
1	1	Terrabacteria group	*Streptococcus thermophilus*

1	1	Terrabacteria group	*Streptococcus thermophilus ASCC 1275*
1	1	Terrabacteria group	*Streptococcus thermophilus CNRZ1066*
1	1	Terrabacteria group	*Streptococcus thermophilus JIM 8232*
1	1	Terrabacteria group	*Streptococcus thermophilus LMD-9*
1	1	Terrabacteria group	*Streptococcus thermophilus LMG 18311*
1	1	Terrabacteria group	*Streptococcus thermophilus MN-ZLW-002*
1	1	Terrabacteria group	*Streptococcus thermophilus ND03*
1	1	Terrabacteria group	*Streptococcus uberis 0140J*
2	2	Terrabacteria group	*Streptomyces albulus*
2	2	Terrabacteria group	*Streptomyces albulus ZPM*
2	2	Terrabacteria group	*Streptomyces albus*
2	2	Terrabacteria group	*Streptomyces albus*
2	2	Terrabacteria group	*Streptomyces albus J1074*
2	2	Terrabacteria group	*Streptomyces ambofaciens ATCC 23877*
2	2	Terrabacteria group	*Streptomyces bingchenggensis BCW-1*
2	2	Terrabacteria group	*Streptomyces cattleya NRRL 8057 = DSM 46488*
2	2	Terrabacteria group	*Streptomyces cattleya NRRL 8057 = DSM 46488*
2	2	Terrabacteria group	*Streptomyces collinus Tu 365*
2	2	Terrabacteria group	*Streptomyces cyaneogriseus subsp. noncyanogenus*
2	2	Terrabacteria group	*Streptomyces fulvissimus DSM 40593*
2	2	Terrabacteria group	*Streptomyces glaucescens*
2	2	Terrabacteria group	*Streptomyces globisporus C-1027*
2	2	Terrabacteria group	*Streptomyces griseus subsp. griseus NBRC 13350*
2	2	Terrabacteria group	*Streptomyces hygroscopicus subsp. jinggangensis 5008*
2	2	Terrabacteria group	*Streptomyces hygroscopicus subsp. jinggangensis TL01*
2	2	Terrabacteria group	*Streptomyces hygroscopicus subsp. limoneus*
2	2	Terrabacteria group	*Streptomyces leeuwenhoekii*
2	2	Terrabacteria group	*Streptomyces lividans TK24*
2	2	Terrabacteria group	*Streptomyces lydicus A02*

2	2	Terrabacteria group	*Streptomyces pratensis ATCC 33331*
2	2	Terrabacteria group	*Streptomyces pristinaespiralis*
2	2	Terrabacteria group	*Streptomyces reticuli*
2	2	Terrabacteria group	*Streptomyces scabiei 87.22*
2	2	Terrabacteria group	*Streptomyces sp. 4F*
2	2	Terrabacteria group	*Streptomyces sp. 769*
2	2	Terrabacteria group	*Streptomyces sp. CCM_MD2014*
2	2	Terrabacteria group	*Streptomyces sp. CdTB01*
2	2	Terrabacteria group	*Streptomyces sp. CFMR 7*
2	2	Terrabacteria group	*Streptomyces sp. CNQ-509*
2	2	Terrabacteria group	*Streptomyces sp. Mg1*
2	2	Terrabacteria group	*Streptomyces sp. PAMC 26508*
2	2	Terrabacteria group	*Streptomyces sp. SirexAA-E*
2	2	Terrabacteria group	*Streptomyces venezuelae*
2	2	Terrabacteria group	*Streptomyces venezuelae*
2	2	Terrabacteria group	*Streptomyces vietnamensis*
2	2	Terrabacteria group	*Streptomyces violaceusniger Tu 4113*
2	2	Terrabacteria group	*Streptomyces xiamenensis*
3	2	Terrabacteria group	*Streptosporangium roseum DSM 43021*
1	1	Terrabacteria group	*Symbiobacterium thermophilum IAM 14863*
2	1	Terrabacteria group	*Synechococcus elongatus PCC 6301*
2	1	Terrabacteria group	*Synechococcus elongatus PCC 7942*
2	1	Terrabacteria group	*Synechococcus sp. CC9311*
2	1	Terrabacteria group	*Synechococcus sp. CC9605*
2	1	Terrabacteria group	*Synechococcus sp. CC9902*
2	1	Terrabacteria group	*Synechococcus sp. JA-2-3B'a(2-13)*
2	1	Terrabacteria group	*Synechococcus sp. JA-3-3Ab*
2	1	Terrabacteria group	*Synechococcus sp. KORDI-100*
2	1	Terrabacteria group	*Synechococcus sp. KORDI-49*

2	1	Terrabacteria group	*Synechococcus sp. KORDI-52*
2	1	Terrabacteria group	*Synechococcus sp. PCC 6312*
2	1	Terrabacteria group	*Synechococcus sp. PCC 7002*
2	1	Terrabacteria group	*Synechococcus sp. PCC 73109*
2	2	Terrabacteria group	*Synechococcus sp. PCC 7502*
1	1	Terrabacteria group	*Synechococcus sp. RCC307*
2	1	Terrabacteria group	*Synechococcus sp. UTEX 2973*
2	1	Terrabacteria group	*Synechococcus sp. WH 7803*
2	1	Terrabacteria group	*Synechococcus sp. WH 8103*
2	1	Terrabacteria group	*Synechococcus sp. WH 8109*
2	1	Terrabacteria group	*Synechocystis sp. PCC 6714*
2	1	Terrabacteria group	*Synechocystis sp. PCC 6803*
2	1	Terrabacteria group	*Synechocystis sp. PCC 6803*
2	1	Terrabacteria group	*Synechocystis sp. PCC 6803*
2	1	Terrabacteria group	*Synechocystis sp. PCC 6803*
2	1	Terrabacteria group	*Synechocystis sp. PCC 6803 substr. GT-I*
2	1	Terrabacteria group	*Synechocystis sp. PCC 6803 substr. PCC-N*
2	1	Terrabacteria group	*Synechocystis sp. PCC 6803 substr. PCC-P*
1	1	Terrabacteria group	*Syntrophobotulus glycolicus DSM 8271*
1	1	Terrabacteria group	*Syntrophomonas wolfei subsp. wolfei str. Goettingen G311*
1	1	Terrabacteria group	*Syntrophothermus lipocalidus DSM 12680*
1	1	Terrabacteria group	*Tepidanaerobacter acetatoxydans Re1*
1	1	Terrabacteria group	*Tepidanaerobacter acetatoxydans Re1*
1	1	Terrabacteria group	*Terribacillus aidingensis*
1	1	Terrabacteria group	*Tetragenococcus halophilus NBRC 12172*
2	1	Terrabacteria group	*Thermacetogenium phaeum DSM 12270*
1	1	Terrabacteria group	*Thermaerobacter marianensis DSM 12885*
1	1	Terrabacteria group	*Thermincola potens JR*
1	2	Terrabacteria group	*Thermoanaerobacter brockii subsp. finnii Ako-1*

1	2	Terrabacteria group	*Thermoanaerobacter italicus Ab9*
1	2	Terrabacteria group	*Thermoanaerobacter kivui*
1	2	Terrabacteria group	*Thermoanaerobacter mathranii subsp. mathranii str. A3*
1	2	Terrabacteria group	*Thermoanaerobacter pseudethanolicus ATCC 33223*
1	2	Terrabacteria group	*Thermoanaerobacter sp. X513*
1	2	Terrabacteria group	*Thermoanaerobacter sp. X514*
1	2	Terrabacteria group	*Thermoanaerobacter wiegelii Rt8.B1*
1	2	Terrabacteria group	*Thermoanaerobacterium thermosaccharolyticum DSM 571*
1	2	Terrabacteria group	*Thermoanaerobacterium thermosaccharolyticum M0795*
1	2	Terrabacteria group	*Thermoanaerobacterium xylanolyticum LX-11*
1	1	Terrabacteria group	*Thermobacillus composti KWC4*
2	1	Terrabacteria group	*Thermobifida fusca YX*
2	2	Terrabacteria group	*Thermobispora bispora DSM 43833*
1	2	Terrabacteria group	*Thermodesulfobium narugense DSM 14796*
1	1	Terrabacteria group	*Thermomicrobium roseum DSM 5159*
2	2	Terrabacteria group	*Thermomonospora curvata DSM 43183*
1	1	Terrabacteria group	*Thermosediminibacter oceani DSM 16646*
2	1	Terrabacteria group	*Thermosynechococcus elongatus BP-1*
1	1	Terrabacteria group	*Thermus aquaticus Y51MC23*
1	1	Terrabacteria group	*Thermus oshimai JL-2*
1	1	Terrabacteria group	*Thermus parvatiensis*
1	1	Terrabacteria group	*Thermus scotoductus SA-01*
1	1	Terrabacteria group	*Thermus sp. CCB_US3_UF1*
1	1	Terrabacteria group	*Thermus thermophilus HB27*
1	1	Terrabacteria group	*Thermus thermophilus HB8*
1	1	Terrabacteria group	*Thermus thermophilus JL-18*
1	1	Terrabacteria group	*Thermus thermophilus SG0.5JP17-16*
2	1	Terrabacteria group	*Trichodesmium erythraeum IMS101*
1	1	Terrabacteria group	*Tropheryma whipplei str. Twist*

1	1	Terrabacteria group	*Truepera radiovictrix DSM 17093*
1	1	Terrabacteria group	*Trueperella pyogenes*
1	1	Terrabacteria group	*Trueperella pyogenes*
1	1	Terrabacteria group	*Trueperella pyogenes TP8*
2	1	Terrabacteria group	*Tsukamurella paurometabola DSM 20162*
0	0	Terrabacteria group	*Ureaplasma parvum serovar 3*
0	0	Terrabacteria group	*Ureaplasma parvum serovar 3 str. ATCC 27815*
0	0	Terrabacteria group	*Ureaplasma parvum serovar 3 str. ATCC 700970*
0	0	Terrabacteria group	*Ureaplasma urealyticum serovar 10 str. ATCC 33699*
1	1	Terrabacteria group	*Veillonella parvula DSM 2008*
2	2	Terrabacteria group	*Verrucosispora maris AB-18-032*
1	1	Terrabacteria group	*Virgibacillus sp. SK37*
1	1	Terrabacteria group	*Weissella ceti*
1	1	Terrabacteria group	*Weissella ceti*
1	1	Terrabacteria group	*Weissella ceti*
1	1	Terrabacteria group	*Weissella cibaria*
1	1	Terrabacteria group	*Weissella koreensis KACC 15510*
1	2	Terrabacteria group	*Xylanimonas cellulosilytica DSM 15894*
1	1	Thermodesulfobacteria	*Thermodesulfatator indicus DSM 15286*
1	1	Thermodesulfobacteria	*Thermodesulfobacterium commune DSM 2178*
1	1	Thermodesulfobacteria	*Thermodesulfobacterium geofontis OPF15*
1	1	Thermotogae	*Defluviitoga tunisiensis*
1	1	Thermotogae	*Fervidobacterium nodosum Rt17-B1*
1	1	Thermotogae	*Fervidobacterium pennivorans DSM 9078*
1	1	Thermotogae	*Kosmotoga olearia TBF 19.5.1*
1	1	Thermotogae	*Kosmotoga pacifica*

1	1	Thermotogae	*Marinitoga piezophila KA3*
1	1	Thermotogae	*Mesotoga prima MesG1.Ag.4.2*
1	1	Thermotogae	*Petrotoga mobilis SJ95*
1	1	Thermotogae	*Pseudothermotoga elfii DSM 9442 = NBRC 107921*
1	1	Thermotogae	*Pseudothermotoga hypogea DSM 11164 = NBRC 106472*
1	1	Thermotogae	*Pseudothermotoga lettingae TMO*
1	1	Thermotogae	*Pseudothermotoga thermarum DSM 5069*
1	1	Thermotogae	*Thermosipho africanus TCF52B*
1	1	Thermotogae	*Thermosipho melanesiensis BI429*
1	1	Thermotogae	*Thermotoga caldifontis AZM44c09*
1	1	Thermotogae	*Thermotoga maritima*
1	1	Thermotogae	*Thermotoga maritima*
1	1	Thermotogae	*Thermotoga maritima MSB8*
1	1	Thermotogae	*Thermotoga maritima MSB8*
1	1	Thermotogae	*Thermotoga maritima MSB8*
1	1	Thermotogae	*Thermotoga maritima MSB8*
1	1	Thermotogae	*Thermotoga naphthophila RKU-10*
0	0	Thermotogae	*Thermotoga neapolitana DSM 4359*
1	1	Thermotogae	*Thermotoga petrophila RKU-1*
1	1	Thermotogae	*Thermotoga profunda AZM34c06*
1	1	Thermotogae	*Thermotoga sp. 2812B*
1	1	Thermotogae	*Thermotoga sp. Cell2*
1	1	Thermotogae	*Thermotoga sp. RQ2*
1	1	Thermotogae	*Thermotoga sp. RQ7*
0	0	unclassified Archaea	*halophilic archaeon DL31*
1	3	unclassified Bacteria	*bacterium L21-Spi-D4*
0	0	unclassified Bacteria	*candidate division SR1 bacterium RAAC1_SR1_1*
0	0	unclassified Bacteria	*Candidatus Saccharibacteria bacterium RAAC3_TM7_1*
0	0	unclassified Bacteria	*Candidatus Saccharibacteria oral taxon TM7x*

1	1	unclassified Bacteria	*Thermobaculum terrenum ATCC BAA-798*
