# Supplementary material for: Myxococcus xanthus DK1622 Coordinates Expressions of the Duplicate groEL and Single groES Genes for Synergistic Functions of GroELs and GroES
Source: Front Microbiol. 2017 Apr 27;8:733. doi: 10.3389/fmicb.2017.00733 (PMC5406781; doi:10.3389/fmicb.2017.00733)
Supplement: Supplementary Table 4 — The occurrence of groEL and groES genes in 24 sequenced myxobacterial genomes. [file Table4.pdf]

**Supplementary Table S4.** The occurrence of *groEL* and *groES* genes in 24 sequenced myxobacterial genomes.

| strain | locus_tag | name | strand | accession |
|---|---|---|---|---|
| *Anaeromyxobacter dehalogenans* 2CP-1 | A2CP1_RS07860 | *groEL2* | + | WP_012632845.1 |
| *Anaeromyxobacter dehalogenans* 2CP-1 | A2CP1_RS18815 | *groES* | + | WP_011422641.1 |
| *Anaeromyxobacter dehalogenans* 2CP-1 | A2CP1_RS18820 | *groEL1* | + | WP_015934824.1 |
| *Anaeromyxobacter dehalogenans* 2CP-C | ADEH_RS12380 | *groEL2* | - | WP_011421446.1 |
| *Anaeromyxobacter dehalogenans* 2CP-C | ADEH_RS18565 | *groES* | + | WP_011422641.1 |
| *Anaeromyxobacter dehalogenans* 2CP-C | ADEH_RS18570 | *groEL1* | + | WP_011422642.1 |
| *Anaeromyxobacter* sp. Fw109-5 | ANAE109_RS07500 | *groEL2* | + | WP_011985785.1 |
| *Anaeromyxobacter* sp. Fw109-5 | ANAE109_RS19060 | *groES* | + | WP_012098520.1 |
| *Anaeromyxobacter* sp. Fw109-5 | ANAE109_RS19065 | *groEL1* | + | WP_012098521.1 |
| *Anaeromyxobacter* sp. K | ANAEK_RS07370 | *groEL2* | + | WP_012525521.1 |
| *Anaeromyxobacter* sp. K | ANAEK_RS18410 | *groES* | + | WP_011422641.1 |
| *Anaeromyxobacter* sp. K | ANAEK_RS18415 | *groEL1* | + | WP_012527640.1 |
| *Archangium gephyra* DSM 2261 | AA314_RS19345 | *groEL1* | - | WP_047856671.1 |
| *Archangium gephyra* DSM 2261 | AA314_RS19350 | *groES* | - | WP_043396073.1 |
| *Archangium gephyra* DSM 2261 | AA314_RS21800 | *groEL2* | + | WP_047857048.1 |
| *Chondromyces apiculatus* DSM 436 | CAP_RS19785 | *groEL* | - | WP_044243374.1 |
| *Chondromyces apiculatus* DSM 436 | CAP_RS19790 | *groES* | - | WP_044243376.1 |
| *Chondromyces apiculatus* DSM 436 | CAP_RS00265 | *groEL* | - | WP_044234642.1 |
| *Chondromyces apiculatus* DSM 436 | CAP_RS00270 | *groES* | - | WP_044234644.1 |
| *Chondromyces crocatus* Cm c5 | CMC5_RS18270 | *groES* | + | WP_050431631.1 |
| *Chondromyces crocatus* Cm c5 | CMC5_RS18275 | *groEL* | + | WP_050431632.1 |
| *Chondromyces crocatus* Cm c5 | CMC5_RS36310 | *groES* | + | WP_050434698.1 |
| *Chondromyces crocatus* Cm c5 | CMC5_RS36315 | *groEL* | + | WP_050434699.1 |
| *Corallococcus coralloides* DSM 2259 | COCOR_RS12630 | *groEL1* | - | WP_014395361.1 |
| *Corallococcus coralloides* DSM 2259 | COCOR_RS12635 | *groES* | - | WP_014395362.1 |
| *Corallococcus coralloides* DSM 2259 | COCOR_RS16530 | *groEL2* | + | WP_014396128.1 |
| *Cystobacter fuscus* DSM 2262 | D187_RS22520 | *groEL1* | - | WP_043431189.1 |
| *Cystobacter fuscus* DSM 2262 | D187_RS22525 | *groES* | - | WP_002624038.1 |
| *Cystobacter fuscus* DSM 2262 | D187_RS38105 | *groEL* | - | WP_002630326.1 |

| | | | | |
|---|---|---|---|---|
| *Cystobacter fuscus* DSM 2262 | D187_RS38365 | *groEL2* | + | WP_002630378.1 |
| *Cystobacter violaceus* Cb vi76 | Q664_RS39225 | *groEL2* | + | WP_043408220.1 |
| *Cystobacter violaceus* Cb vi76 | Q664_RS17205 | *groEL1* | - | WP_043396071.1 |
| *Cystobacter violaceus* Cb vi76 | Q664_RS17210 | *groES* | - | WP_043396073.1 |
| *Haliangium ochraceum* DSM 14365 | HOCH_RS26645 | *groES* | + | WP_012830442.1 |
| *Haliangium ochraceum* DSM 14365 | HOCH_RS26650 | *groEL* | + | WP_012830443.1 |
| *Haliangium ochraceum* DSM 14365 | HOCH_RS32730 | *groEL* | - | WP_012831647.1 |
| *Haliangium ochraceum* DSM 14365 | HOCH_RS32735 | *groES* | - | WP_012831648.1 |
| *Hyalangium minutum* DSM 14724 | DB31_RS11645 | *groEL2* | + | WP_044186442.1 |
| *Hyalangium minutum* DSM 14724 | DB31_RS22055 | *groES* | + | WP_044191117.1 |
| *Hyalangium minutum* DSM 14724 | DB31_RS22060 | *groEL1* | + | WP_044191118.1 |
| *Myxococcus fulvus* 124B02 | MFUL124B02_RS27270 | *groEL2* | - | WP_046714602.1 |
| *Myxococcus fulvus* 124B02 | MFUL124B02_RS29475 | *groES* | + | WP_013941457.1 |
| *Myxococcus fulvus* 124B02 | MFUL124B02_RS29480 | *groEL1* | + | WP_046714963.1 |
| *Myxococcus fulvus* HW-1 | LILAB_RS29980 | *groEL2* | - | WP_013941070.1 |
| *Myxococcus fulvus* HW-1 | LILAB_RS31935 | *groES* | + | WP_013941457.1 |
| *Myxococcus fulvus* HW-1 | LILAB_RS31940 | *groEL1* | + | WP_013941458.1 |
| *Myxococcus stipitatus* DSM 14675 | MYSTI_RS24550 | *groEL2* | - | WP_015350497.1 |
| *Myxococcus stipitatus* DSM 14675 | MYSTI_RS26680 | *groES* | + | WP_015350918.1 |
| *Myxococcus stipitatus* DSM 14675 | MYSTI_RS26685 | *groEL1* | + | WP_015350919.1 |
| *Myxococcus xanthus* DK 1622 | MXAN_RS21695 | *groEL2* | - | WP_011554465.1 |
| *Myxococcus xanthus* DK 1622 | MXAN_RS23760 | *groES* | + | WP_002640434.1 |
| *Myxococcus xanthus* DK 1622 | MXAN_RS23765 | *groEL1* | + | WP_011554876.1 |
| *Myxococcus xanthus* DZ2 | MXDZ_RS0219785 | *groEL2* | + | WP_011554465.1 |
| *Myxococcus xanthus* DZ2 | MXDZ_RS0233565 | *groES* | + | WP_002640434.1 |
| *Myxococcus xanthus* DZ2 | MXDZ_RS0233570 | *groEL1* | + | WP_011554876.1 |
| *Myxococcus xanthus* DZF1 | MXF1_RS0137875 | *groEL2* | + | WP_011554465.1 |
| *Myxococcus xanthus* DZF1 | MXF1_RS0133640 | *groES* | + | WP_002640434.1 |
| *Myxococcus xanthus* DZF1 | MXF1_RS0133645 | *groEL1* | + | WP_011554876.1 |
| *Plesiocystis pacifica* SIR-1 | PPSIR1_RS03005 | *groEL* | - | WP_006969504.1 |
| *Plesiocystis pacifica* SIR-1 | PPSIR1_RS03010 | *groES* | - | WP_006969505.1 |
| *Sandaracinus amylolyticus* DSM 53668 | DB32_RS16520 | *groEL* | - | WP_053233422.1 |
| *Sandaracinus amylolyticus* DSM 53668 | DB32_RS16525 | *groES* | - | WP_053233423.1 |

| | | | | |
|---|---|---|---|---|
| *Sorangium cellulosum* So ce56 | SCE_RS14260 | *groES* | + | WP_012235412.1 |
| *Sorangium cellulosum* So ce56 | SCE_RS14265 | *groEL* | + | WP_012235413.1 |
| *Sorangium cellulosum* So ce56 | SCE_RS30385 | *groES* | + | WP_012238539.1 |
| *Sorangium cellulosum* So ce56 | SCE_RS30390 | *groEL* | + | WP_012238540.1 |
| *Sorangium cellulosum* So0157-2 | SCE1572_RS17550 | *groES* | + | WP_020735364.1 |
| *Sorangium cellulosum* So0157-2 | SCE1572_RS17555 | *groEL* | + | WP_020735365.1 |
| *Sorangium cellulosum* So0157-2 | SCE1572_RS36395 | *groES* | + | WP_020738934.1 |
| *Sorangium cellulosum* So0157-2 | SCE1572_RS36400 | *groEL* | + | WP_020738935.1 |
| *Sorangium cellulosum* So0157-2 | SCE1572_RS37890 | *groEL* | + | WP_020739228.1 |
| *Stigmatella aurantiaca* DW4/3-1 | STAUR_RS23745 | *groEL2* | - | WP_002615259.1 |
| *Stigmatella aurantiaca* DW4/3-1 | STAUR_RS27970 | *groES* | + | WP_002615502.1 |
| *Stigmatella aurantiaca* DW4/3-1 | STAUR_RS27975 | *groEL1* | + | WP_013376916.1 |
| *Vulgatibacter incomptus* DSM 27710 | AKJ08_RS08640 | *groES* | + | WP_050725696.1 |
| *Vulgatibacter incomptus* DSM 27710 | AKJ08_RS08645 | *groEL* | + | WP_050725697.1 |
